# Productive Homogeneous Hydrogenation of Fatty Esters with Carboxylate SNS Ruthenium Catalysts

**DOI:** 10.1002/chem.202501898

**Published:** 2025-07-16

**Authors:** Maurizio Ballico, Damian Grainger, Tasnim Al‐Kassous, Joseph Burke, Lucas Carreras, Julian Zuber, Walter Baratta

**Affiliations:** ^1^ Dipartimento di Scienze AgroAlimentari Ambientali e Animali (DI4A) Università di Udine Via Cotonificio 108 Udine 33100 Italy; ^2^ Johnson Matthey 28 Cambridge Science Park Milton Road Cambridge CB4 0FP UK; ^3^ Catalysis Research Center Technical University of Munich Ernst‐Otto‐Fischer‐Straße 1 85748 Garching b. München Germany

**Keywords:** carboxylates, fatty esters, hydrogenation, reduction, ruthenium

## Abstract

The carboxylate pincer complexes *trans*‐[Ru(η^1^‐OCOR)_2_(SNS)(PPh_3_)] (R = CH_3_ **1**, *^t^* Bu **2**; SNS = bis(2‐(ethylthio)ethylamine) are obtained from [Ru(η^2^‐OCOR)_2_(PPh_3_)_2_] (R = CH_3_, *^t^* Bu) and HN(CH_2_CH_2_SEt)_2_ in acetone by elimination of PPh_3_. Substitution of PPh_3_ in **1** with PPh_2_Me gives *trans*‐[Ru(η^1^‐OAc)_2_(SNS)(PPh_2_Me)] (**3**) in toluene at 105°C. The cationic complexes *cis*‐[Ru(η^1^‐OAc)(SNS)(dppb)]OAc (**4**) and [Ru(η^1^‐OAc)(CO)(SNS)(PPh_3_)]OAc (**5**) are synthesized from [Ru(η^2^‐OAc)_2_(dppb)] and [Ru(η^2^‐OAc)_2_(CO)(PPh_3_)_2_], respectively, by treatment with the SNS ligand in toluene. Hydride *trans*‐[RuH(η^1^‐OAc)(SNS)(PPh_3_)] (**6**) is obtained by reaction of **1** with NaO*i*Pr in a mixture of *i*PrOH/toluene. The carboxylate complexes **1**–**6** catalyze the homogeneous hydrogenation of methyl decanoate to 1‐decanol with NaOMe under mild reaction conditions (T = 40°C, H_2_ pressure 27.5 bar) and extremely low catalyst loading (S/C 10,000–100,000). Complexes **1** and **2** display remarkably high catalytic activity for the reduction of fatty acid methyl and ethyl esters at S/C of 50,000–100,000, with high selectivity for the reduction of the carboxylate group in the presence of C ═ C functionalities. An easy scale‐up of this process (100 mmol of substrate) has been demonstrated. Mechanistic studies show that **1** reacts with H_2_ (5 bar), affording the monohydride **6,** that by further reaction with H_2_ and an alkoxide gives the dihydride *cis*‐[RuH_2_(SNS)(PPh_3_)], which reduces methyl benzoate to benzyl alcohol.

## Graphical abstract

Carboxylate ruthenium complexes, prepared from easily accessible ruthenium precursors and bis(2‐(ethylthio)ethyl)amine (SNS ligand), efficiently catalyze the hydrogenation (HY) of methyl decanoate (model for the FAME reduction) to 1‐decanol with H_2_ under mild conditions and without solvents. The acetate *trans*‐[Ru(η^1^‐OAc)_2_(SNS)(PPh_3_)] derivative catalyzes the HY of a number of long‐chain and aromatic methyl‐ and ethyl esters at extremely high S/C (up to 100, 000).

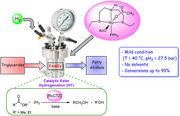

## 1 Introduction

The reduction of esters to alcohols is a fundamental process of great concern for both academia and industry, on account of the increasing interest in the valorization of sustainable raw materials.^[^ ^1^ ^]^ Bio‐based resources obtained from seeds and vegetable oils can be transformed into fatty alcohols (FAls) and other bulk chemicals. Traditional methods for ester reduction require stoichiometric amounts of metal hydrides (e.g., LiAlH_4_, NaBH_4_, and DIBAL‐H), entailing cumbersome workup and production of large amounts of waste.^[^ ^2^ ^]^ Conversely, catalytic hydrogenation (HY) with molecular hydrogen is a highly atom‐economic and environmentally more sustainable process, which can be carried out in the industry using heterogeneous catalysts. Large‐scale industrial processes are based on Cu‐Cr and Ni catalysts, although the use of catalysts with Ru, Rh, Pd, and Re has also been demonstrated.^[^ ^3^ ^]^ Such processes require high pressures and temperatures (>200°C, >200 atm) and lead to the formation of side products with reduction of C ═ C bonds besides the target HY of the ester functionality. Homogeneous catalysts for HY reactions have been deeply investigated in the past decades, showing the ability to operate more selectively and under mild reaction conditions.^[^ ^4^ ^]^ Since the 1980s, a number of homogeneous catalysts for ester HY have been reported.^[^ ^5^ ^]^ Important developments have taken place since 2006, when Milstein et al. described the catalytic ester HY with PNN‐Ru‐based homogeneous catalysts.^[^ ^6^ ^]^ Subsequently, researchers at Firmenich^[^ ^7^ ^]^ and Takasago,^[^ ^8^ ^]^ prepared highly active Ru‐based catalysts for HY of a wide range of nonactivated esters under relatively mild reaction conditions (S/C > 100 with respect to substrate, T ≤ 115 °C and H_2_ pressure ≤ 50 bar). Since then, several homogeneous catalysts have been synthesized to increase the productivity of ester HY, mainly derived from phosphine‐based bifunctional and pincer ligands (PNP, PNN, CNN, NNN, PNS) and employing Ru, but also Os, Ir, Mn, and the earth‐abundant Co and Fe metals.^[^ ^9^ ^]^ The achievement of high conversion to product at low catalyst loadings (high S/C) with efficient volume‐time yields (high turnover frequency (TOF), with high concentration) is critical for the industrial application of homogeneous HY technology. Gusev and co‐workers reported a family of Ru and Os‐PNN pyridine aminophosphine (PNN) pincer catalysts with turnover numbers (TONs) of ≈ 18, 000 in the HY of methyl benzoate at 100°C and 50 bar H_2_.^[^ ^10^ ^]^ Later, the same group also described a series of ruthenium complexes with an aliphatic SNS pincer ligand, including *trans*‐[RuCl_2_(SNS)(PPh_3_)] that allows a TON of ≈ 60, 000 in ethyl acetate HY at 40°C and 50 bar H_2_.^[^ ^11^ ^]^ It is worth noting that this complex also catalyzes the acceptorless dehydrogenative coupling of ethanol to ethyl acetate. In the last few years, mono‐, bi‐, and tridentate ruthenium(II) *N*‐heterocyclic carbene complexes (NHCs) have also been demonstrated to catalyze the HY of esters, generating TONs up to 53, 900.^[^ ^12^ ^]^ However, most of the Ru‐NHC‐based catalysts require relatively harsh reaction conditions in terms of temperature and pressure. More recently, Mn‐ and Mo‐based bis(NHC) complexes have been reported for the homogeneous HY of esters.^[^ ^13^ ^]^

It is worth noting that FAls or long‐chain alcohols, namely octyl, cetyl, and stearyl alcohols are relevant compounds for industry and they are produced on a multi‐million‐ton scale each year.^[^ ^14^ ^]^ FAls find their application as intermediates in the production of fragrances, pharmaceuticals, emulsifiers, lubricants, and detergents. They are obtained either from fossil petrochemical resources through *Aufbau‐Reaction* and/or *Oxo processes* or, more often, by HY of fatty acids and their methyl esters (FAMEs). FAMEs, which constitute biodiesel, in turn are prepared by transesterification with methanol of triglycerides from seed or animal oils/fats.^[^ ^15^ ^]^ Most of the current industrial processes to make the derived FAls involve the HY of FAMEs through heterogeneous catalysts at high temperature (250–300°C) and H_2_ pressure (150–200 bar). Because of to the harsh reaction conditions associated with the existing processes, substantial effort has been put into finding homogeneous catalysts that can convert FAMEs under milder conditions. Ru‐MACHO has been successfully used in the HY of methyl oleate and other FAMEs (also as mixtures) under relatively mild conditions (S/C 1000, 20 bar H_2_ and 110°C).^[^ ^16^ ^]^ Guan et al. have demonstrated that ruthenium‐ and iron‐based pincer complexes with PNN, PNP, and SNS ligands hydrogenate FAMEs under neat conditions (S/C up to 3330).^[^ ^17^ ^]^ The osmium complexes [OsHX(CO)(NH(CH_2_P*^i^* Pr_2_)_2_)] (X = Cl, H) were evaluated in the HY of hexyl octanoate and *cis*‐3‐hexenyl hexanoate to alcohols as model substrates for triglycerides (at S/C 1000) even if at high temperature and pressure (220°C, 55 bar of H_2_).^[^ ^18^ ^]^ Gusev further demonstrated the application of ruthenium catalysts to the selective reduction of fatty esters even in the presence of terminal C ═ C bonds (S/C 2000, T = 40–100 °C, 5–10 bar of H_2_). Very recently a new efficient method, based on the in‐situ direct alcoholysis of natural oils to less sterically hindered FAMEs, separation of glycerol and HY to FAls with a quinaldine‐based PNN‐Ru pincer complex, using a two‐phase solvent system, has been proposed.^[^ ^19^ ^]^

Herein we report a straightforward preparation of neutral and cationic ruthenium carboxylate complexes containing mono‐, di‐phosphine and Gusev's SNS pincer‐type ligand, using easily accessible ruthenium precursors. These catalysts have been found highly active in the homogeneous HY of methyl and ethyl esters to alcohols. In particular, FAME substrates are easily reduced under low H_2_ pressure and temperature (27.5 bar, 40°C), with high chemoselectivity, low catalyst loadings (S/C 50, 000–100, 000) and without the use of solvents.

## 2 Results and Discussion

### 2.1 Synthesis of SNS Carboxylate Ruthenium Complexes

**Scheme 1:**
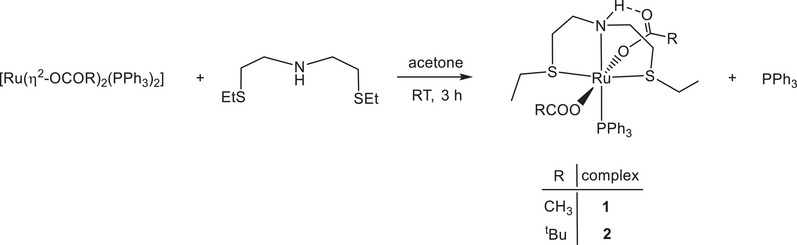
Synthesis of SNS carboxylate complexes **1** and **2**.

Treatment of [Ru(η^2^‐OAc)_2_(PPh_3_)_2_] with bis(2‐(ethylthio)ethyl)amine (SNS) (1 equiv) in acetone at room temperature for 3 hours, afforded the neutral derivative *trans*‐[Ru(η^1^‐OAc)_2_(SNS)(PPh_3_)] (**1**), as a light‐yellow precipitate in 88% yield by elimination of triphenylphosphine (Scheme 1).

**Figure 1:**
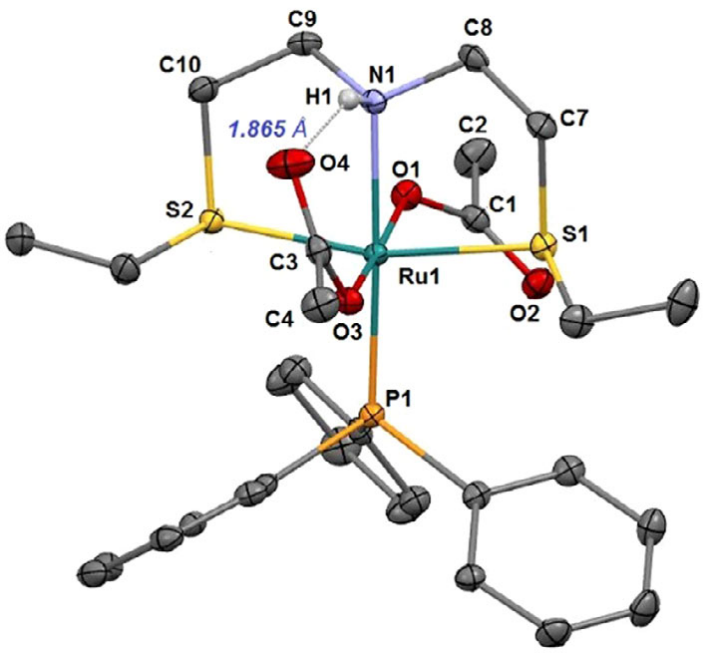
ORTEP representation of compound **1** in the solid state (CCDC 2 449 278). Ellipsoids are shown at the 50% probability level. Hydrogen atoms (except for the amino proton) are omitted for clarity. The dotted line indicates a possible intramolecular hydrogen bond. Selected bond lengths [Å] and angles [°]: Ru1–O1 2.101(2), Ru1–O3 2.123(2), Ru1–N1 2.154(2), Ru1–P1 2.2975(8), Ru1–S1 2.3419(8), Ru1–S2 2.3440(8), S1‐C7 1.838(3), S2‐C10 1.838(3), N1–C8 1.473(4), N1–C9 1.482(4), O1‐Ru1‐O3 174.77(8), O1‐Ru1‐N1 83.22(9), O3‐Ru1‐N1 91.66(9), O1‐Ru1‐P1 95.07(6), O3‐Ru1‐P1 90.10(6), N1‐Ru1‐P1 176.87(7), O1‐Ru1‐S1 90.44(6), O3‐Ru1‐S1 88.02(6), N1‐Ru1‐S1 84.36(7), P1‐Ru1‐S1 98.29(3), O1‐Ru1‐S2 83.75(6), O3‐Ru1‐S2 96.64(6), N1‐Ru1‐S2 82.42(7), P1‐Ru1‐S2 94.80(3), S1‐Ru1‐S2 166.10(3), C8‐N1‐C9 112.2(2).

The thermally stable compound **1** was isolated as a mixture of three stereoisomers (95, 3, and 2%, respectively), as inferred from the ^31^P{^1^H} NMR spectrum, which shows a singlet at δ = 48.8 ppm for the main species and two close singlets at δ = 47.5 and 47.3 ppm (toluene‐*d* ^8^) (Figure S1). These isomers are in slow interconversion in solution at RT, on account of the different configuration at the two sulfur atoms, as reported for the derivatives *trans*‐[RuXCl(SNS)(PPh_3_)] (X = Cl, H).^[^ ^11^, ^20^ ^]^ Thus the three isomers of **1** have the phosphine PPh_3_ *trans* to the N atom, as inferred from 2D ^1^H‐^31^P HMBC NMR experiments, with a *mer* SNS and two *trans* acetate ligands. The main isomer shows an intramolecular hydrogen bond between the NH moiety and one acetate, with δ_H_ = 10.74 ppm, whereas the two OAc methyl groups are at δ = 1.88 and 1.70 ppm. In addition, the signal at δ_C_ = 182.4 ppm is for the CO interacting with the NH moiety, whereas the other acetate resonance is at δ = 177.8 ppm. The presence of the acetate ligands makes **1** highly soluble in several organic solvents, including toluene, CH_2_Cl_2_, CH_3_OH, and diethyl ether. The molecular structure of **1** established by NMR analysis has been corroborated through a single crystal X‐ray diffraction experiment in the solid state, showing a ruthenium center in a distorted octahedral environment (Figure 1).

The Ru‐O distance in **1** is practically the same for the two acetate groups (2.101 and 2.123 Å), whereas the Ru‐P and the Ru‐N distances were found to be 2.2975(8) and 2.154(2) Å, respectively, which is slightly lower than those observed for *trans*‐[RuCl_2_(SNS)(PPh_3_)] (2.3097(6) and 2.164(2) Å).^[^ ^11^ ^]^ The meridionally coordinated SNS‐ligand gives rise to very similar Ru‐S (2.3419(8) and 2.3440(8) Å) bond lengths, while the S1‐Ru‐S2 angle is 166.10(3)° with the two thioether ethyl groups in a mutual *syn* arrangement. The solid‐state study of **1** confirmed the presence, already observed in solution, of an intramolecular hydrogen bond interaction of one acetate with the N‐H proton (the H1···O4 distance is 1.865 Å), leading to a six‐membered cycle (N1···O4 = 2.772(2) Å). The N‐H bond is almost parallel to the Ru‐O3 bond (H1‐N1‐Ru1‐O3 dihedral angle of about ‐10.60°, with an H1···O3 distance of 2.776(2) Å).

The pivalate derivative *trans*‐[Ru(η^1^‐OPiv)_2_(SNS)(PPh_3_)] (**2**) was synthesized following the procedure used for **1**, by reaction of [Ru(η^2^‐OPiv)_2_(PPh_3_)_2_] with the SNS ligand (Scheme 1) and has been isolated in 88% yield. The ^31^P{^1^H} spectrum of **2** reveals the presence of two stereoisomers in 93 and 7%, showing two singlets at δ = 49.8 and 47.3 ppm, respectively. The ^1^H NMR resonances at δ = 1.20 and 0.95 ppm in toluene‐*d* ^8^ are for the two pivalate ligands of the main isomer, whereas the NH signal at δ = 11.50 ppm is in agreement with the hydrogen bond interaction of the NH moiety with one pivalate.

**Scheme 2:**
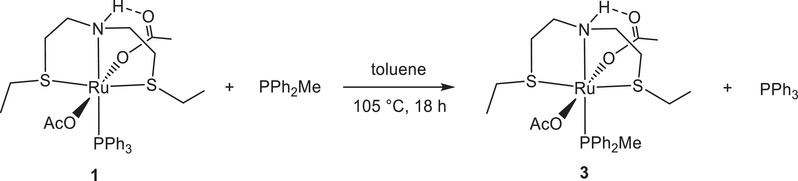
Synthesis of the PPh_2_Me complex **3**.

Treatment of **1** with methyldiphenylphosphine in toluene at 105°C for 18 hours, affords *trans*‐[Ru(η^1^‐OAc)_2_(SNS)(PPh_2_Me)] (**3**), isolated in 91% yield, by substitution of PPh_3_ with the more basic and less bulky PPh_2_Me phosphine (Scheme 2).

**Figure 2:**
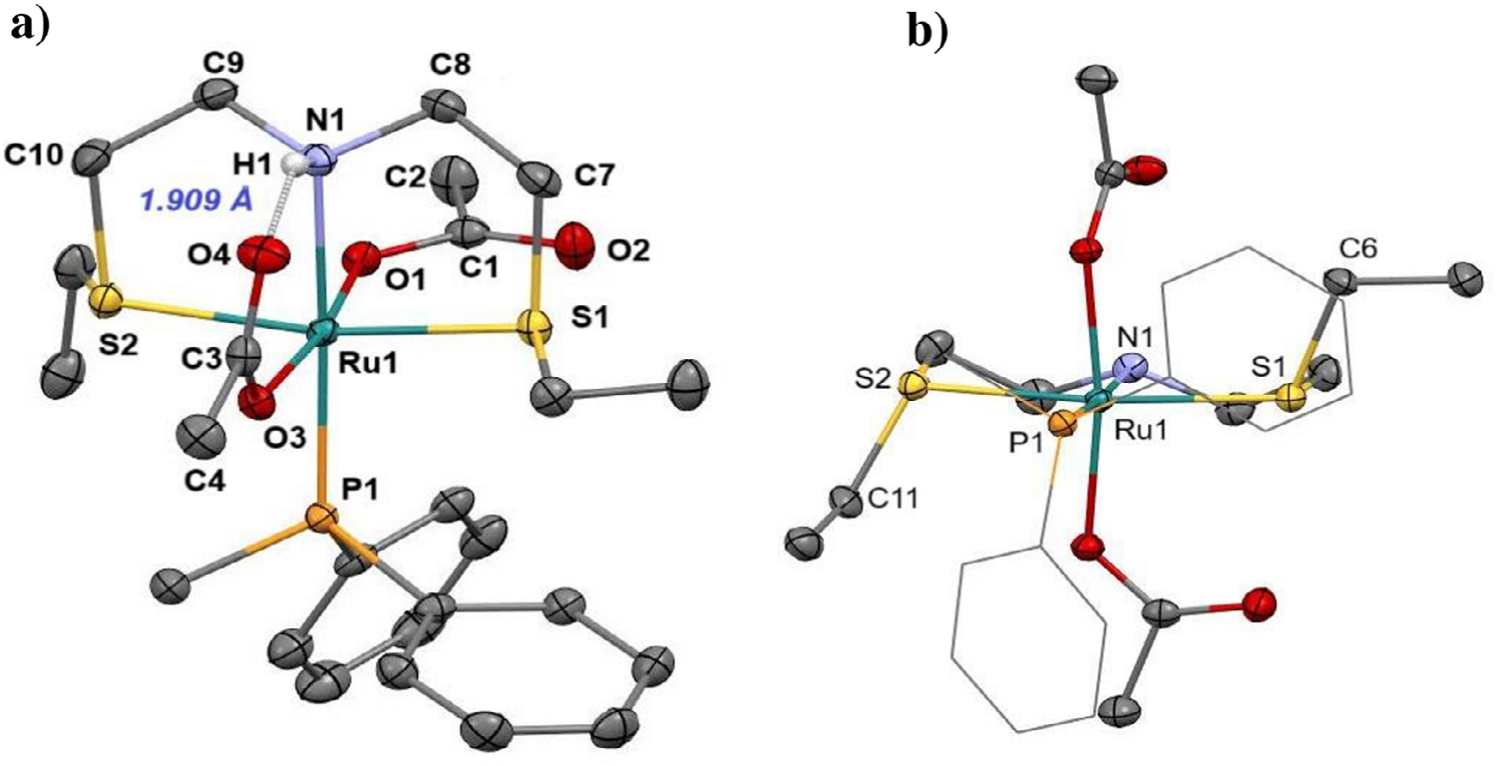
**a**) ORTEP representation of compound **3** in the solid state (CCDC 2 449 279). Ellipsoids are shown at the 50% probability level. Hydrogen atoms (except for the amino proton) are omitted for clarity. The dotted line indicates a possible intramolecular hydrogen bond. **b**) Different orientation of the molecular structure of complex **3**, in this representation hydrogen atoms are omitted, and phosphine methyl and phenyl groups are simplified as wireframes for clarity. Selected bond lengths [Å] and angles [°]: Ru1–O1 2.1049(13), Ru1–O3 2.1173(13), Ru1–N1 2.1473(15), Ru1–P1 2.2804(5), Ru1–S1 2.3457(5), Ru1–S2 2.3527(5), S1‐C7 1.846(2), S2‐C10 1.829(2), N1–C8 1.469(2), N1–C9 1.474(3), O1‐Ru1‐O3 171.88(5), O1‐Ru1‐N1 85.93(6), O3‐Ru1‐N1 93.65(6), O1‐Ru1‐P1 97.72(4), O3‐Ru1‐P1 82.56(4), N1‐Ru1‐P1 176.14(5), O1‐Ru1‐S1 95.81(4), O3‐Ru1‐S1 92.21(4), N1‐Ru1‐S1 83.96(4), P1‐Ru1‐S1 96.879(18), O1‐Ru1‐S2 86.75(4), O3‐Ru1‐S2 85.14(4), N1‐Ru1‐S2 84.02(4), P1‐Ru1‐S2 94.897(18), S1‐Ru1‐S2 167.492(18), C8‐N1‐C9 113.72(15).

Similarly to **1**, the derivative **3** was isolated as a mixture of three isomers in 83, 13, and 4%, as revealed by the ^31^P{^1^H} NMR spectrum (peaks at δ_P_ = 33.5, 33.0, and 32.4 ppm, respectively), due to the different configurations at the S atoms. A hydrogen bond interaction involving the NH moiety and one acetate is evidenced by the low‐field ^1^H NMR resonance at δ = 10.34 ppm for the main isomer, and with the signal at δ_C _= 182.6 ppm for the interacting carboxylate, compared to the other O*CO*CH_3_ group (δ_C_ = 177.7 ppm). It is worth noting that the one‐pot synthesis starting from [Ru(η^2^‐OAc)_2_(PPh_3_)_2_] and PPh_2_Me (1 equiv) in toluene at RT leads to [Ru(η^2^‐OAc)_2_(PPh_3_)(PPh_2_Me)] as predominant species. Addition of the SNS ligand at RT affords the cationic intermediate *cis*‐[Ru(η^1^‐OAc)_2_(PPh_3_)(PPh_2_Me)(SNS)]OAc (doublets at δ_P_ = 28.2 and 22.6 ppm with ^2^ *J*(P,P) of 37.2 Hz), which by heating at 60°C in 2 hours gives **3** in the presence of **1** (9:1 molar ratio). The molecular structure of **3**, obtained by SC‐XRD, bearing the less sterically hindered phosphine PPh_2_Me (Figure 2) resembles that of **1** with a similar Ru‐N, ‐P, ‐S, and ‐O connectivity, but with the sulfur‐bound ethyl groups displaying an *anti*‐arrangement with respect to the central equatorial plane of the SNS ligand (Figure 2b).^[^ ^20^ ^]^ Also in this case, an intramolecular hydrogen bond interaction involving the N‐H proton with one acetate (H1···O4 distance of 1.909 Å) is observed in the solid‐state study. The obtained six‐membered cycle (N1···O4 = 2.816(3) Å) shows that the N‐H bond is almost parallel to the Ru‐O3 bond (H1‐N1‐Ru1‐O3 dihedral angle of about 5.49°, with an H1···O3 distance of 2.821(2) Å).

**Scheme 3:**
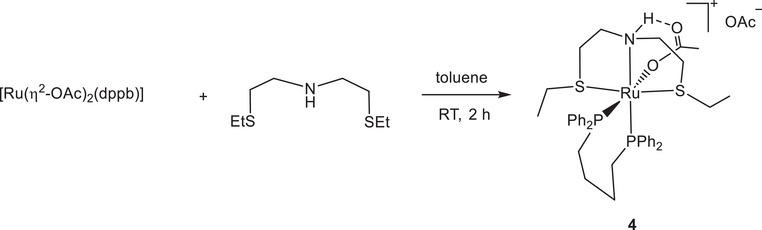
Synthesis of the cationic diphosphine complex **4**.

The cationic complex *cis*‐[Ru(η^1^‐OAc)(SNS)(dppb)]OAc (**4**) was obtained in 73% yield by reaction of [Ru(η^2^‐OAc)_2_(dppb)] (dppb = 1,4‐bis(diphenylphosphino)butane) with the SNS ligand in toluene at RT in 2 hours, by displacement of an acetate (Scheme 3).

The ^31^P{^1^H} NMR spectrum of **4** shows two doublets at δ = 37.7 and 36.5 ppm (^2^ *J*(P,P) of 38.1 Hz) for the phosphorous *trans* to N and O atoms, respectively, as inferred by a ^1^H‐^31^P HMBC 2D NMR experiment. Differently from complexes **1**–**3**, only a single stereoisomer is observed. ^1^H NMR resonances at δ = 2.01 and 1.84 ppm are for the free and coordinated acetate methyls, respectively, whereas the downfield shifted signal at δ = 10.43 ppm is for the NH moiety that interacts with the internal acetate. In the ^13^C{^1^H} NMR spectrum, the singlets at δ = 182.7 and 178.4 ppm are for the coordinated and free acetate carbonyls.

**Scheme 4:**
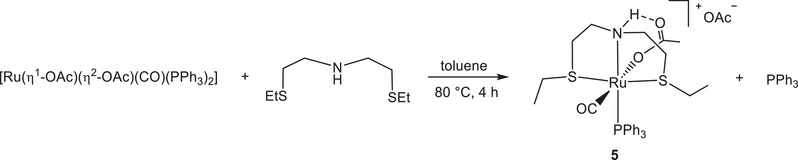
Synthesis of the cationic monocarbonyl complex **5**.

The cationic monocarbonyl derivative [Ru(η^1^‐OAc)(CO)(SNS)(PPh_3_)]OAc (**5**) was obtained in 76% yield by treatment of [Ru(η^1^‐OAc)(η^2^‐OAc)(CO)(PPh_3_)_2_] with the SNS ligand in toluene at 80°C in 4 hours, by displacement of OAc and PPh_3_ ligands (Scheme 4).

The ^31^P{^1^H} NMR spectrum of **5** in CD_3_OD shows three singlets at δ = 35.8, 35.7, and 31.8 ppm, in about 6.6:1.4:1 molar ratio, respectively, indicating the formation of three stereoisomers. A different order of distribution of the isomers (δ_P_ = 40.8, 37.1, and 33.2 ppm; 1.5:6.6:1 molar ratio) has been observed in toluene‐*d* ^8^, with the main isomer showing a low‐field NH resonance at δ_H_ = 11.28 ppm, as for **1**–**4**. The ^13^C{^1^H} NMR signal of the Ru‐CO appears as a doublet at δ = 201.4 ppm with a ^2^ *J*(C,P) of 10.3 Hz, indicating a *cis* arrangement with respect to PPh_3_.^[^ ^21^ ^]^ The presence of a weak NOE effect between the *ortho*‐phenyl phosphine protons at δ = 7.58 ppm and the methyl group of the acetate ligand bound to the ruthenium at δ = 1.63 ppm, shows that, in the main isomer, the CO is *trans* to the OAc, while the NH is *trans* to the phosphine, as determined by 2D ^1^H‐^1^H NOESY NMR experiments (Figure S45). This arrangement deduced from NMR studies is also supported by a single crystal X‐ray diffraction experiment, even if the poor quality of the crystal and the present disorder do not allow the accurate molecular structure of **5** (Figure S124).

**Scheme 5:**
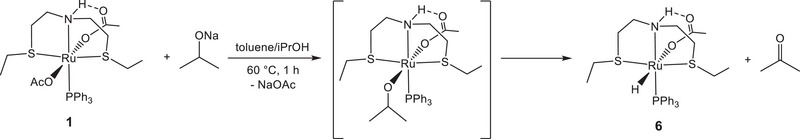
Synthesis of the monohydride complex **6**.

The ruthenium monohydride *trans*‐[RuH(η^1^‐OAc)(SNS)(PPh_3_)] (**6**) was obtained in 95% yield by reaction of **1** with NaO*^i^* Pr (1 equiv) in a toluene/*i*PrOH mixture (1:1 in volume) at 60°C for 1 hour, by the elimination of acetone (Scheme 5).

This reaction occurs through the formation of the monoalkoxide intermediate *trans*‐[Ru(O*^i^* Pr)(η^1^‐OAc)(SNS)(PPh_3_)], via acetate substitution, followed by β‐H elimination. Hydride **6** can be easily obtained by removing acetone through evaporating the isopropanol/toluene mixture, according to the equilibrium reaction.^[^ ^22^ ^]^ Compound **6** was isolated as a mixture of three stereoisomers in 1:0.6:0.2 molar ratio, (δ_P_ = 60.6, 62.5, and 65.7 ppm, respectively), due to the different configurations of the sulfur atoms, as for *trans*‐[RuHCl(SNS)(PPh_3_)].^[^ ^20^ ^]^ The hydride ligand of the three isomers appears as doublets at δ_H_ = ‐21.54, ‐21.94 and ‐21.82 (major isomer) ppm with ^2^ *J*(H,P) = 23.2, 25.9, and 26.2 Hz, respectively, *cis* to PPh_3_ and *trans* to the acetate ligands. 2D ^1^H‐^1^H NOESY NMR experiments in toluene‐*d* ^8^ show a NOE interaction between Ru‐H and the *ortho*‐phenyl protons of PPh_3_ (about δ = 7.97 ppm), but no exchange with the NH proton. In addition, the presence of cross‐peaks for the exchange correlations between the ^1^H NMR resonances confirms that the three isomers are in equilibrium (Figure S53). A hydrogen bond between the NH and carbonyl of the acetate is present, as inferred from the upfield shifted NH at δ_H_ = 10.70 and 10.06 ppm (minor species), and δ_H_ = 10.18 ppm (major isomer). Complex **6** is highly soluble in aromatic solvents, slowly reacts in CHCl_3_ affording chlorinated species and is oxygen and moisture sensitive. Attempts to prepare **6** from [RuH(η^2^‐OAc)(PPh_3_)_3_]^[^ ^23^ ^]^ and the SNS ligand in toluene or *n*‐heptane at 80–100 °C failed, leading to a mixture of **1** and the dihydride *cis*‐[RuH_2_(SNS)(PPh_3_)]^[^ ^11^ ^]^ (δ_P_ = 82.0 ppm), as observed by NMR measurements.

### 2.2 HY of Esters Catalyzed by Carboxylate SNS Ruthenium Complexes

**Scheme 6:**
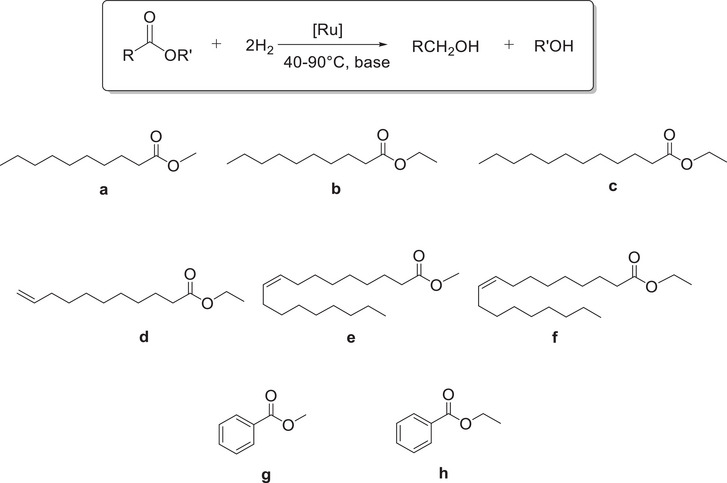
Reduction of methyl and ethyl esters via HY catalyzed by complexes **1**–**6**.

The catalytic activity of the carboxylate complexes **1**–**6** has been investigated on methyl and ethyl fatty esters HY under mild H_2_ pressure (27.5 bar) with substrate/catalyst ratios (S/C) of 10, 000–100, 000, in the presence and absence of solvent, and at temperatures in the range of 40 to 90°C (Scheme 6).

It is worth noting that methyl esters are the standard substrates in industry, although ethyl esters display generally a better conversion with respect to methyl esters, on account of the inhibiting effect of the methanol by‐product.^[^ ^3^, ^12^ ^]^ The HY of methyl decanoate **a** into 1‐decanol and MeOH is a good model for the reduction of fatty acid methyl esters (FAME) and it has been carried out with catalysts **1**–**6** without the addition of solvent and in the presence of NaOMe 50 mol% at 40°C (Table 1).

**Table 1:** Catalytic HY of methyl decanoate **a** with **1**–**6** and *trans*‐[RuCl_2_(SNS)(PPh_3_)] (S/C = 10, 000–100,000) NaOMe 50 mol% at 27.5 bar of H_2_ at 40°C, neat, 16 hours.

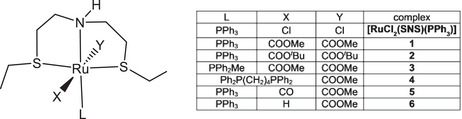			
Entry	Complex	S/C	Conv.^[^ ^a^ ^]^ [%]
1	**1**	10, 000	100
2	**1**	50, 000	100
3	**1**	100, 000	64
4	**2**	10, 000	100
5	**2**	50, 000	90
6	**3**	10, 000	75
7	**4**	10, 000	65
8	**5**	10, 000	69
9	**6**	10, 000	97
10	**6**	50, 000	75
11	[RuCl_2_(SNS)(PPh_3_)]	10, 000	100
12	[RuCl_2_(SNS)(PPh_3_)]	50, 000	79
13	[RuCl_2_(SNS)(PPh_3_)]	100, 000	2

^[a]^ Determined by GC analyses.

At S/C = 10, 000 full conversion is achieved after 16 hours with the PPh_3_ complexes **1**, **2**, **6,** and *trans*‐[RuCl_2_(SNS)(PPh_3_)] (entries 1, 4, 9, and 11 of Table 1). Conversely, the PPh_2_Me **3**, and the cationic derivatives **4** and **5** lead to 65–75% conversion (entries 6, 7, and 8). When the catalyst loading was further reduced (S/C 50, 000), high conversion is observed for the carboxylate **1**, **2** (100, 90% conv., entries 2, 5), whereas the Gusev catalyst *trans*‐[RuCl_2_(SNS)(PPh_3_)] affords 78% conv. (entry 12). Conversely, **3** and **5** at S/C 50, 000 lead to partial formation of 1‐decanol at 40°C (31 and 28%, entries 7 and 11, Table S1). Notably, the presence of the electron‐donating PPh_2_Me in **3** leads to a more stable complex compared to the PPh_3_ derivative **1**, thus preventing the release of phosphine that has been observed when **1** reacts with strong bases and H_2_. In addition, complex **5** containing CO displays similar activity at 90°C, indicating slow deactivation (entry 12, Table S1). The superior activity of the acetate **1** with respect to the chloride *trans*‐[RuCl_2_(SNS)(PPh_3_)] is evidenced at S/C 100, 000, with 64 versus 2% conversion, respectively, (entries 3 and 13). By increasing the temperature at 90°C incomplete HY is observed, suggesting that the active dihydride species undergoes a deactivation at high temperature (see Table S1).

The monohydride **6** leads to quantitative formation of 1‐decanol at S/C = 10, 000, whereas at higher S/C ratios incomplete conversion is observed (75% at S/C = 50, 000, entry 10). The minor activity of this hydride ruthenium complex compared to the dicarboxylates **1** and **2**, which are much more effective catalysts at low loadings, can be ascribed to the higher air and moisture sensitivity of **6**.

Catalysts **1** and **2** have been tested in the HY of methyl and ethyl esters, using NaOMe and NaOEt, respectively, to avoid transesterification (Scheme 6 and Table 2).

**Table 2:** Catalytic HY of esters with **1**, **2,** and *trans*‐[RuCl_2_(SNS)(PPh_3_)], with base 50 mol% at 27.5 bar of H_2_ and in the absence of solvent after 16 hours.

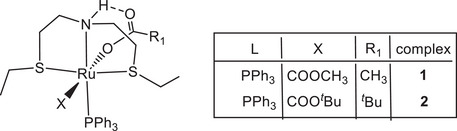						
Entry	Complex	Substrate	S/C	base	T [°C)]	Conv.^[^ ^b^ ^]^ [%]
1	**1** ^[^ ^a^ ^]^	**b**	50, 000	NaOEt	40	97
2	**1**		100, 000	NaOEt	40	99
3	**2** ^[^ ^a^ ^]^		50, 000	NaOEt	40	95
4	[RuCl_2_(SNS)(PPh_3_)]		100, 000	NaOEt	40	93
5	**1**	**c**	44, 000	NaOEt	40	100
6	**1**		100, 000	NaOEt	40	98
7	**1**	**d**	10, 000	NaOEt	40	99
8	**1**		50, 000	NaOEt	40	84
9	**1**	**e** ^[^ ^c^ ^]^	25, 000	NaOMe	60	100
10	**1**		50, 000	NaOMe	50	99
11	**1**		100, 000	NaOMe	50	74
12	[RuCl_2_(SNS)(PPh_3_)]		100, 000	NaOMe	50	49
13	**1**	**f** ^[^ ^c^ ^]^	50, 000	NaOEt	40	97
14	**1**		100, 000	NaOEt	40	96
15	**1**		100, 000	NaOEt	50	91
16	[RuCl_2_(SNS)(PPh_3_)]		100, 000	NaOEt	50	55
17	**1**	**g**	100, 000	NaOMe	40	84
18	[RuCl_2_(SNS)(PPh_3_)]		100, 000	NaOMe	40	77
19	**1**	**h**	50, 000	NaOEt	40	98
20	**1**		100, 000	NaOEt	40	100
21	**2**		100, 000	NaOEt	40	94

^[a]^ Reaction in toluene.

^[b]^ Determined by GC analyses.

^[c]^ Substrates **e** and **f** were of technical grade and contains methyl or ethyl oleate (>76‐80%) but also other polyunsaturated (methyl or ethyl linoleate and linolenate, etc.) and saturated esters (methyl or ethyl palmitate and stearate): the reported conversions are referred to the HY of the real methyl or ethyl oleate present in the starting material, although the reduction of the other esters to the respective alcohols (linoleyl alcohol, palmityl alcohol, etc.) has been also observed (see Supporting Information).

With these catalysts the HY of ethyl decanoate **b** occurs more easily compared to methyl substrate **a**. Thus, complexes **1** and **2** reduce quantitatively **b** to 1‐decanol at 40°C at S/C 50, 000 in toluene, with **1** that affords complete reduction also at S/C 100, 000 in neat conditions in 16 hours (entries 1–3 of Table 2), with 93% conv. for the chloride analogue (entry 4). Ethyl dodecanoate **c** is completely hydrogenated to the corresponding alcohol by **1** at S/C of 44, 000, and 98% conv. was observed at S/C of 100, 000 without solvent at 40 °C (entries 5, 6). The unsaturated ethyl 10‐undecenoate **d** is selectively converted to 10‐undecen‐1‐ol at S/C 10, 000 without remarkable HY or isomerization of the C ═ C bond (with the presence of less than 1% of 1‐undecanol),^[^ ^18^ ^]^ whereas at S/C 50, 000 the unsaturated alcohol is obtained in 84% conversion (entries 7, 8). Methyl oleate **e** is quantitatively reduced with **1** to oleyl alcohol ((*Z*)‐Octadec‐9‐en‐1‐ol) with preservation of the C ═ C bond in the range of S/C 25, 000–50, 000 at 50–60 °C (entries 9–10). At S/C 100, 000 the catalyst **1** provides 74% conversion, whereas *trans*‐[RuCl_2_(SNS)(PPh_3_)] gives 49% conv. (entries 11–12). Ethyl oleate **f** is more easily reduced and affords the oleyl alcohol at a lower temperature (40°C) at S/C 50, 000–100, 000 with **1** and the related chloride (entries 13, 14, and 16). Interestingly, **1** is active also at 50°C leading to 91% conv., while *trans*‐[RuCl_2_(SNS)(PPh_3_)] gives 55% of the hydrogenated product (entries 15 and 16). Finally, **1** affords 84% conv. of methyl benzoate **g** at S/C 100, 000, while the chloride derivative gives 77% conv. in the same conditions (entries 17 and 18). The ethyl substrate **h** was completely hydrogenated to benzylic alcohol at S/C 50, 000 and 100, 000 at 40°C by **1** in the absence of solvents (entries 19–20), whereas the pivalate **2** shows a 94% conv. (entry 21 and see also Table S2). The preparation of 1‐decanol by HY of **b** on a 100 mmol scale at S/**1** = 50, 000 and without solvents under mild conditions (45°C, 28 bar H_2_ pressure, 50 mol% NaOEt), has also been easily carried out in high yield (95%) and purity (99%), demonstrating the easy scale‐up of this process, which is of crucial importance for pharmaceutical and large‐scale industrial productions. Analogously, oleyl alcohol was also prepared from technical grade **f** on a 70 mmol scale using similar reaction conditions.

These results indicate that at a high S/C ratio (50, 000–100, 000) the FAMEs are better hydrogenated using the carboxylate Ru‐SNS catalysts, compared to the chloride, indicating that the carboxylate gives access to a more cost‐efficient catalyst for the HY of FAMEs.

### 2.3 Hydrogen Uptake During Catalysis and NMR Studies

**Figure 3:**
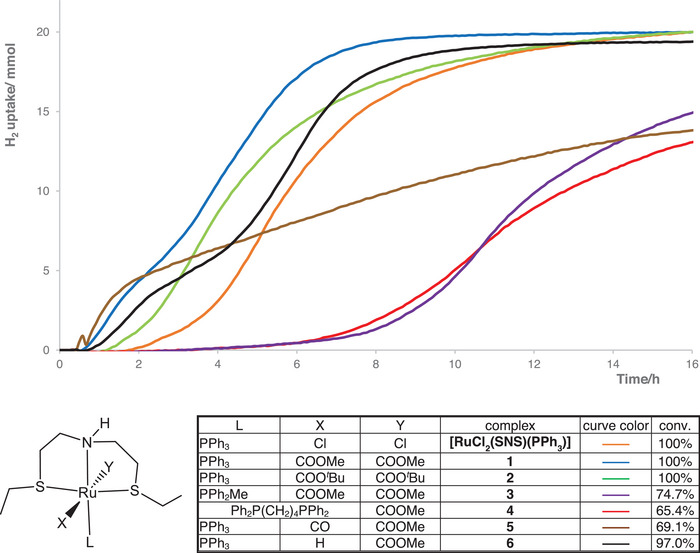
Curves of the hydrogen uptake for the HY of **a** with **1–6** and *trans*‐[RuCl_2_(SNS)(PPh_3_)] at 40°C, 27.5 bar of H_2_ and 50 mol% NaOMe (S/C 10, 000).

To shed light on the steps involved in the HY of esters, hydrogen uptake measurements and NMR studies have been carried out with complexes **1**–**6**. The curves of the hydrogen consumption for the HY of **a** with **1**–**6** at S/C 10, 000 are reported in Figure 3, and these data are compared with those obtained with *trans*‐[RuCl_2_(SNS)(PPh_3_)].

The carboxylate **1**, **2** containing PPh_3_ shows a short induction period, whereas the PPh_2_Me and the dppb derivatives **3** and **4** require more than 6 hours to become active in the HY, resulting in incomplete conversion of the substrate after 16 hours. The cationic monocarbonyl complex **5** shows a rapid activation, followed by a slowdown in H_2_ uptake, possibly due to the formation of several hydride species by displacement of PPh_3_ and CO (see NMR studies). As expected, the monohydride **6** shows a rapid activation (Figure S78). Complex *trans*‐[RuCl_2_(SNS)(PPh_3_)] requires an induction period of about 2 hours, indicating that the carboxylates **1**, **2** are more active, affording quantitative HY in shorter reaction times, at different temperatures, catalyst loadings, and with several substrates. As previously described, methyl esters require longer reaction times with respect to the analogous ethyl derivatives, while for some substrates (i.e., methyl oleate), higher temperatures (50 or 60°C) are needed to achieve quantitative conversion (see Supporting Information for details Figures S77‐S99).

**Scheme 7:**
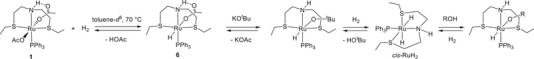
Equilibrium between complex **1**, *trans*‐[RuH(X)(SNS)(PPh_3_)] (X = OAc, O*^t^* Bu) and *cis*‐[RuH_2_(SNS)(PPh_3_)] species.

NMR experiments have been carried out to investigate the species formed during the HY of the carboxylate complexes. Reaction of **1** in toluene‐*d* ^8^ with H_2_ (5 bar) without base at 70°C affords the monohydride **6** in small amount, without the formation of dihydride derivatives (Scheme 7).

Treatment of **1** with KO*^t^* Bu (3 equiv) under H_2_ at 50°C after 2 hours gives a mixture of the mono‐ and dihydride compounds *trans*‐[RuH(X)(SNS)(PPh_3_)] (X = OAc, O*^t^* Bu) and *cis*‐[RuH_2_(SNS)(PPh_3_)], respectively (Scheme 7). At higher temperatures (70°C) the amount of the dihydride species increases, without disappearance of the monohydride derivatives (Figures S55‐S61). The monohydride complexes show singlets at δ_P_ = 60–70 ppm and doublets in the range δ_H_ = ‐10 to ‐22 ppm, whereas the dihydride *cis*‐[RuH_2_(SNS)(PPh_3_)] gives a signal at δ_P_ = 81.2 and a doublet at δ_H_ = ‐12.1 ppm with ^2^ *J*(H,P) = 27.4 Hz. The formation of several RuH species is also due to the presence of stereoisomers, differing for the configuration at the sulfur atoms. Addition of methyl benzoate (5 equiv) to the mixture of RuH/RuH_2_ species under H_2_ atmosphere (5 bar) and in the presence of KO*^t^* Bu (3 equiv) leads to the complete formation of benzylic alcohol and MeOH at 70°C in 5 hours (Figures S62‐S64), confirming that *cis*‐[RuH_2_(SNS)(PPh_3_)] is the catalytically active species in the ester HY and that a strong base is necessary to generate the dihydride.^[^ ^11^ ^]^ No appreciable reduction of methyl benzoate has been observed without a strong base. KO*^t^* Bu has been used as the base of choice on account of its relative stability and because its NMR signals do not overlap with those of the species under examination. Reactions of the monohydride **6** with H_2_ in toluene‐*d* ^8^ with KO*^t^* Bu are fully consistent with the results observed for **1**, with formation of the dihydride in equilibrium with **6** only in the presence of base, as inferred from 2D ^1^H‐^1^H NOESY NMR experiments (Figures S60‐S61). Interestingly, the addition of an excess of 2‐propanol (10 equiv) to the NMR tube containing *cis*‐[RuH_2_(SNS)(PPh_3_)] results in the quantitative conversion to a monohydride alkoxide species, under H_2_ atmosphere (5 atm) (Figure S65), thus limiting the formation of the catalytically active species (Scheme 7). This point is particularly crucial since the products of ester HY are primary alcohols, and therefore an excess of base is required to hinder the dihydride protonation, allowing quantitative HY. NMR experiments performed with *trans*‐[RuCl_2_(SNS)(PPh_3_)], which is poorly soluble in toluene, indicate that higher temperature (80–90°C) and longer reaction time (overnight) are required for the formation of mono‐and dihydride species (Figure S66).

The effect of the nature of the base in the reaction of **1** with H_2_ has also been investigated. The use of the non‐nucleophilic base 1,8‐diazabicyclo[5.4.0]undec‐7‐ene (DBU) (3 equiv.) with **1** and H_2_ (5 bar) in toluene‐*d* ^8^ affords quantitatively **6** at 100°C in 15 days (Figures S67‐S68). Conversely, addition of diisobutylaluminium hydride (DIBAL‐H) (1 equiv as dimer) to **1**, gives dihydride species (4 hours at 70°C) with deprotonation of the NH group (Figures S69‐S70).^[^ ^11^, ^24^ ^]^ This mixture also proved to be capable of reducing methyl benzoate (5 equiv with respect to **1**) to benzylic alcohol and MeOH under H_2_ atmosphere. Control experiments show that under these conditions and without **1**, no conversion to alcohol has been observed with DIBAL‐H.^[^ ^25^ ^]^ Our study has shown that DIBAL‐H can be used as a co‐catalyst for the conversion of the acetate SNS ruthenium derivatives to RuH_2_ active species for ester HY. With an excess of DIBAL‐H (3 equiv. as dimer with respect to **1**) at 70°C a deprotonated dihydride complex is formed, namely *cis*‐[RuH_2_(SNS)(PPh_3_)]^−^ [AlH_2_(*^i^* Bu)_2_]^+^ (δ_H_ = ‐12.76 ppm) in the presence of a small amount of a monohydride derivative *cis*‐[RuH(SNS)(PPh_3_)_2_] (δ_P_ = 57.0 and 17.9 ppm with ^2^ *J*(P,P) = 29.2 Hz and δ_H_ = ‐10.07 ppm with ^2^ *J*(H,P)_trans_ = 109.1 Hz, ^2^ *J*(H,P)_cis_ = 24.5 Hz), possibly formed by reaction of the ruthenium hydride **6** with PPh_3_ produced by partial decomposition of **1** (Figures S71‐S72).

Finally, NMR experiments performed with the monocarbonyl **5** have shown that, in the presence of H_2_ and KO*^t^* Bu (3 equiv), a mixture of mono‐ and dihydride ruthenium species containing PPh_3_ and CO are generated at 70 °C in 2 hours. Thus, in addition to **6** and *cis*‐[RuH_2_(SNS)(PPh_3_)], the corresponding CO monohydride [RuHX(CO)(SNS)] (X = OAc, O*^t^* Bu) (δ_H_ = ‐15/‐18 ppm) and dihydride [RuH_2_(CO)(SNS)] complexes (δ_H_ = ‐10.33 ppm) have been identified by ^1^H‐^31^P HMBC and 2D ^13^C NMR analyses (Figures S73‐S76). This indicates that the reaction of **5** with H_2_ and a base leads to RuH and RuH_2_ species by displacement of CO and PPh_3_ ligands.

### 2.4 Proposed Mechanism

Based on these results, a mechanism for the HY of esters with **1**–**3** and **6** is proposed in agreement with the studies of Gusev.^[^ ^11^ ^]^ Reaction of the carboxylate complexes with H_2_ in the presence of a strong base leads to the catalytically active dihydride complex, in equilibrium with monohydride species, which reacts with the ester substrate to generate a hemiacetaloxide complex.

**Scheme 8:**
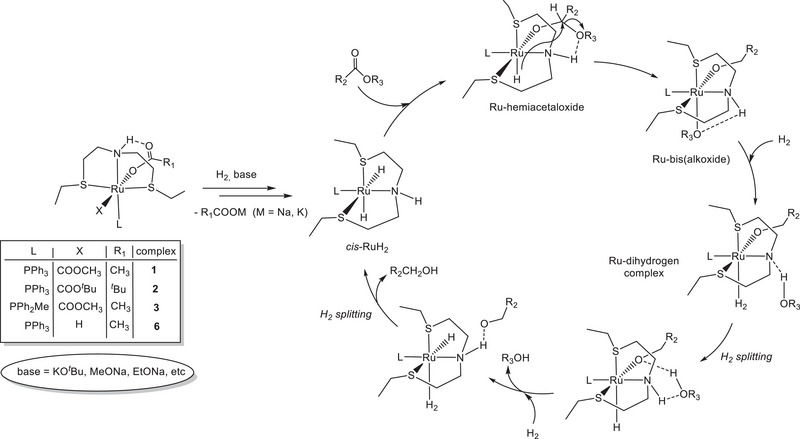
Proposed mechanism for HY of esters catalyzed by the ruthenium carboxylate SNS complexes.

Intramolecular hydride attack leads to a Ru‐bis(alkoxide) containing an NH function, that reacts with H_2_ leading to a RuH(alkoxide) via heterolytic H_2_ splitting and elimination of an alcohol molecule. Further reaction with a second H_2_ molecule affords the RuH_2_ species, which closes the cycle with extrusion of the second alcohol product (Scheme 8). It is worth noting that the H_2_ activation is mediated by the presence of the NH group^[^ ^26^ ^]^ that increases the reactivity of the Ru‐alkoxide through a hydrogen bond interaction, in line also with our data on the carboxylate **1**–**6**. The NMR experiments have shown that esters are reduced by RuH_2_ and that this species is protonated by alcohols to RuH(alkoxide), suggesting that the lower activity of methyl esters is due to the higher acidity of methanol by‐product, with respect to ethanol. In addition, the use of strong bases in excess (MeONa, NaOEt, KO*^t^* Bu) has a beneficial effect on the ester HY, by reducing traces of H_2_O and carboxylic acids present in commercial esters and lowering the acidity of the alcohol products.^[^ ^27^ ^]^ Attempts to detect Ru‐hemiacetaloxide and Ru‐bis(alkoxide) in solution failed. The NMR analysis of the HY reaction mixtures has provided no evidence of the formation of hemiacetal or aldehydes during the process, ruling out the release of these intermediates, as proposed by Milstein^[^ ^28^ ^]^ and Saudan.^[^ ^7b^ ^]^ Conversely, the isolation of Ru‐hemiacetaloxide at low temperature has been described by Bergens,^[^ ^29^ ^]^ using lactones, and observed by Pidko^[^ ^12d^ ^]^ and Gusev,^[^ ^30^ ^]^ employing ESI‐MS techniques, whereas the synthesis of fluoral hemiacetals from polyfluorinated esters has been reported by Dub.^[^ ^27^ ^]^ More recently, computational studies on ester HY with Gusev's SNS complex suggest a direct hydride transfer mechanism involving the five‐coordinated *mer*‐[RuH(SNS)(PPh_3_)], but we have no evidence of this possible pathway.^[^ ^31^ ^]^ The NMR and H_2_ uptake experiments indicate that the generation of the active dihydride species occurs much more rapidly with the carboxylate complexes compared to the chloride *trans*‐[RuCl_2_(SNS)(PPh_3_)]. The long induction time observed for the PPh_2_Me derivative **3**, compared to **1**, is likely due to the higher basicity of the phosphine, which can retard the formation of RuH_2_ species. Also for complex **4**, bearing the diphosphine dppb, the HY starts slowly since the formation of the RuH_2_ may require the displacement of a P atom, taking also into account the stronger basic character of the resulting PPh_2_R moiety. In addition, the complete dissociation of the phosphines may result in the deactivation of these systems. Conversely, the monocarbonyl catalyst **5** shows high activity similar to **1** in the first 2 hours, followed by a decrease of rate. This is likely due to the formation of the highly active dihydride [RuH_2_(PPh_3_)(SNS)], as well as the more robust and less active [RuH_2_(CO)(SNS)] carbonyl species, according to the catalytic pathway described for **1** and the NMR studies on the reaction of **5** with H_2_ in the presence of a base.

## 3 Conclusions

In summary, we have reported the straightforward preparation of new carboxylate SNS ruthenium complexes starting from ruthenium carboxylate phosphine precursors and the HN(CH_2_CH_2_SEt)_2_ ligand (SNS). These derivatives catalyze efficiently the HY of methyl decanoate, used as a model for the FAME reduction, to 1‐decanol with H_2_ under mild reaction conditions (40°C and H_2_ pressure of 27.5 bar), without the use of solvents. The acetate *trans*‐[Ru(η^1^‐OAc)_2_(SNS)(PPh_3_)] has shown remarkably high activity for the HY of several long‐chain and aromatic methyl‐ and ethyl esters to alcohols at extremely high S/C (up to 100, 000), with high selectivity for the reduction of ester carbonyl group versus C ═ C in unsaturated esters. An easy scale‐up of this process (100 mmol of substrate) has been demonstrated for industrial applications. NMR studies have suggested that the dihydride *cis*‐[RuH_2_(SNS)(PPh_3_)] is the active species, which forms by reaction of the carboxylate complexes with hydrogen in basic conditions. Further studies are ongoing to shed new light on the mechanism of the ester HY promoted by the carboxylate derivatives and to apply these catalysts in other C‐H forming reactions.

## 4 Experimental Section

### General methods

All reactions were carried out under an argon atmosphere using standard Schlenk techniques. The solvents were carefully dried by standard methods and distilled under argon before use. The ruthenium complexes [Ru(η^2^‐OAc)_2_(PPh_3_)_2_],^[^ ^32^ ^]^ [Ru(η^2^‐OAc)_2_(dppb)],^[^ ^33^ ^]^ [Ru(η^2^‐OPiv)_2_(PPh_3_)_2_],^[^ ^34^ ^]^ [RuH(η^2^‐OAc)(PPh_3_)_3_],^[^ ^23^ ^]^ [Ru(η^1^‐OAc)(η^2^‐OAc)(CO)(PPh_3_)_2_],^[^ ^35^ ^]^ and the SNS^[^ ^11^, ^36^ ^]^ ligand were prepared according to literature procedures, whereas all other chemicals were purchased from Merck and Strem and used without further purification. NMR measurements were recorded on Bruker Avance III HD NMR 400 spectrometer. Chemical shifts (ppm) are relative to TMS for ^1^H and ^13^C{^1^H}, whereas H_3_PO_4_ was used for ^31^P{^1^H}. Elemental analyses (C, H, N, S) were carried out with a Carlo Erba 1106 analyzer, whereas GC analyses of catalytic products were performed with a Varian CP‐3900 gas chromatograph system equipped with an Agilent HP‐88, 60 m x 0.25 mm x 0.2 µm column using nitrogen (1.2 mL/minute) as the carrier gas and a flame ionization detector (FID).

### Synthesis of *Trans*‐[Ru(η^1^‐OAc)_2_(SNS)(PPh_3_)] (1)

[Ru(η^2^‐OAc)_2_(PPh_3_)_2_] (200 mg, 0.269 mmol) was suspended in acetone (5 mL) and the SNS ligand (54.6 mg, 0.282 mmol, 1.05 equiv) was added. The mixture was stirred for 3 hours at room temperature. The obtained light‐yellow precipitate was filtered, washed with *n*‐heptane (4×3 mL), diethyl ether (2×2 mL), *n*‐pentane (2×3 mL) and dried under reduced pressure. The compound was isolated as a mixture of a major isomer (95%) and two minor species (5%), differing by the configuration of the S atoms. Yield: 159.7 mg (88%); ^1^H NMR (400.1 MHz, toluene‐*d* ^8^, 25 °C): δ = 10.74 (br s, 0.95H; NH major isomer), 10.58 (br s, 0.03H; NH minor species), 9.78 (br s, 0.02H; NH minor species), 8.12 (t, ^3^ *J*(H,H) = 9.4 Hz; Ph minor species), 7.92 (t, ^3^ *J*(H,H) = 8.5 Hz, 5H; Ph major isomer), 7.71 (t, ^3^ *J*(H,H) = 8.1 Hz; Ph minor species), 7.43–7.30 (br m; Ph minor species), 7.20 (br t, ^3^ *J*(H,H) = 6.9 Hz, 5H; Ph major isomer), 7.16–6.99 (m, 5H; Ph major isomer), 6.95–6.80 (br m; Ph minor species), 2.96–2.87 (m, 2H; NCH_2_ major isomer), 2.86–2.71 (m, 2H; NCH_2_ major isomer), 2.68–2.60 (m, 2H; CH_2_ major isomer), 2.50 (pseudo t, ^3^ *J*(H,H) = 10.5 Hz, 2H; CH_2_ major isomer), 2.38–2.25 (m; CH_2_ minor species), 2.18–2.01 (m, 2H; C*H*_2_CH_3_ major isomer), 1.88 (s, 3H; OCOCH_3_ major isomer), 1.70 (br s, 3H; OCOCH_3_ major isomer), 1.47–1.25 (m, 2H; C*H*_2_CH_3_ major isomer), 1.27 (br s; OCOCH_3_ minor species), 0.94 (t, ^3^ *J*(H,H) = 7.0 Hz; CH_2_C*H*_3_ minor species), 0.87 ppm (t, ^3^ *J*(H,H) = 7.4 Hz, 6H; CH_2_C*H*_3_ major isomer); ^13^C{^1^H} NMR of the major isomer (100.6 MHz, toluene‐*d* ^8^, 25 °C): δ = 182.4 (br s; O*C*OCH_3_), 177.8 (s; O*C*OCH_3_), 137.2–124.6 (m; aromatic carbon atoms), 48.9 (s; N*C*H_2_), 40.3 (s; S*C*H_2_), 30.5 (s; S*C*H_2_CH_3_), 24.7 (s; OCO*C*H_3_), 24.6 (s; OCO*C*H_3_), 12.8 ppm (s; CH_2_ *C*H_3_); ^31^P{^1^H} NMR (162.0 MHz, toluene‐*d* ^8^, 25 °C): δ = 48.8 (s; major isomer, 95%), 47.5 (br s; minor species, 3%), 47.3 ppm (br s; minor species, 2%); elemental analysis calcd (%) for C_30_H_40_NO_4_PRuS_2_ (674.82): C 53.40, H 5.97, N 2.08, S 9.50; found: C 53.33, H 5.95, N 2.10, S 9.45.

### Synthesis of *Trans*‐[Ru(η^1^‐OPiv)_2_(SNS)(PPh_3_)] (2)

Complex **2** was prepared by following the procedure used for the synthesis of **1**, with [Ru(η^2^‐OPiv)_2_(PPh_3_)_2_] (200 mg, 0.242 mmol) in place of [Ru(η^2^‐OAc)_2_(PPh_3_)_2_] and the SNS ligand (49.0 mg, 0.254 mmol, 1.05 equiv), leading to the product as a light‐yellow solid. The compound was isolated as a mixture of a major isomer (93%) and a minor species (7%) differing by the configuration of the S atoms. Yield: 161.3 mg (88%); ^1^H NMR (400.1 MHz, toluene‐*d* ^8^, 25 °C): δ = 11.50 (br s, 1H; NH major isomer), 11.16 (br s, 1H; NH minor species), 7.94 (t, ^3^ *J*(H,H) = 2.1 Hz, 5H; Ph major isomer), 7.43–7.26 (br m; Ph protons minor species), 7.20 (br t, ^3^ *J*(H,H) = 1.7 Hz, 5H; Ph major isomer), 7.16–6.96 (m, 5H; Ph major isomer), 6.94–6.80 (br m; Ph minor species), 3.05–2.85 (m, 2H; NCH_2_ major isomer), 2.84–2.70 (m, 2H; NCH_2_ major isomer), 2.69–2.45 (m, 4H; CH_2_ major isomer), 2.34–2.00 (m, 2H; C*H*_2_CH_3_ major isomer), 1.68–1.43 (m, 2H; C*H*_2_CH_3_ major isomer), 1.20 (br s, 9H; OCOC(CH_3_)_3_), 0.95 (br s, 9H; OCOC(CH_3_)_3_), 0.89 (t, ^3^ *J*(H,H) = 1.9 Hz, 6H; CH_2_C*H*_3_ major isomer); ^13^C{^1^H} NMR of the major isomer (100.6 MHz, toluene‐*d* ^8^, 25 °C): δ = 184.3 (br s; O*C*OC(CH_3_)_3_), 138.1–124.3 (m; aromatic carbon atoms), 48.6 (s; N*C*H_2_), 40.2 (s; S*C*H_2_), 30.5 (s; S*C*H_2_CH_3_), 37.5 (br s; OCO*C*(CH_3_)_3_), 28.5 (br s; OCOC(*C*H_3_)_3_), 12.6 ppm (s; CH_2_ *C*H_3_); ^31^P{^1^H} NMR (162.0 MHz, toluene‐*d* ^8^, 25 °C): δ = 49.8 (br s; minor species, 7%), 47.3 ppm (s; major isomer, 93%); elemental analysis calcd (%) for C_36_H_52_NO_4_PRuS_2_ (758.98): C 56.97, H 6.91, N 1.85, S 8.45; found: C 56.92, H 6.96, N 1.80, S 8.46.

### Synthesis of *Trans*‐[Ru(η^1^‐OAc)_2_(SNS)(PPh_2_Me)] (3)

Complex **1** (200 mg, 0.296 mmol) was dissolved in degassed toluene (5 mL), PPh_2_Me (82.8 µL, 89 mg, 0.445 mmol, 1.5 equiv), was added and the mixture was stirred at 105°C for 18 hours. The resulting light orange solution was concentrated to about 1 mL under reduced pressure. Addition of *n*‐heptane (10 mL) afforded the precipitation of the product as a pale‐yellow solid that was filtered, washed with a mixture of diethyl ether/*n*‐heptane (5/1 (v/v); 2×2 mL), *n*‐heptane (4×3 mL) and *n*‐pentane (4×2 mL) and dried under reduced pressure. The compound was isolated as a mixture of 3 isomers differing by the configuration of the S atoms. Yield: 165.0 mg (91%); ^1^H NMR (400.1 MHz, toluene‐*d* ^8^, 25 °C): δ = 10.51 (br s, 1H; NH minor isomer), 10.34 (br s, 1H; NH major species), 8.07–6.78 (m, 10H; Ph protons), 3.15–2.80 (m, 3H; NCH_2_), 2.80–2.58 (m, 2H; CH_2_), 2.58–2.35 (m, 2H; CH_2_), 2.34–2.00 (m, 3H; NCH_2_), 2.07 (br d, ^2^ *J*(H,P) = 6.1 Hz, 3H; PCH_3_), 1.92 (s, 3H; OCOCH_3_), 1.90 (s, 3H; OCOCH_3_), 1.72–1.54 (s, 1H; C*H*_2_CH_3_), 1.42–1.21 (m, 1H; C*H*_2_CH_3_), 0.86 (t, ^3^ *J*(H,H) = 6.5 Hz, 3H; CH_2_C*H*_3_), 0.77 ppm (br t, ^3^ *J*(H,H) = 6.7 Hz, 3H; CH_2_C*H*_3_); ^13^C{^1^H} NMR (100.6 MHz, toluene‐*d* ^8^, 25 °C): δ = 182.6 (s; O*C*OCH_3_), 177.7 (br s; O*C*OCH_3_), 138.5–123.2 (m; aromatic carbon atoms), 48.8 (s; N*C*H_2_), 47.2 (s; N*C*H_2_), 40.9 (s; S*C*H_2_), 40.5 (s; S*C*H_2_ minor species), 37.2 (s; S*C*H_2_), 31.7 (s; S*C*H_2_CH_3_), 30.8 (s; S*C*H_2_ minor species), 24.9 (s; S*C*H_2_CH_3_), 24.7 (s; OCO*C*H_3_), 24.3 (s; OCO*C*H_3_), 13.1 (d, ^1^ *J*(C,P) = 31.2 Hz; P*C*H_3_), 12.9 (s; CH_2_ *C*H_3_), 12.3 ppm (s; CH_2_ *C*H_3_); ^31^P{^1^H} NMR (162.0 MHz, toluene‐*d* ^8^, 25 °C): δ = 33.5 (s; 4%), 33.0 (s; 13%), 32.4 ppm (s; 83%); elemental analysis calcd (%) for C_25_H_38_NO_4_PRuS_2_ (612.75): C 49.00, H 6.25, N 2.29, S 10.46; found: C 48.92, H 6.27, N 2.23, S 10.50.

### Synthesis of *Cis*‐[Ru(η^1^‐OAc)(SNS)(dppb)]OAc (4)

[Ru(η^2^‐OAc)_2_(dppb)] (200 mg, 0.310 mmol) and the SNS ligand (66 mg, 0.341 mmol, 1.1 equiv) were dissolved in degassed toluene (5 mL), and the mixture was stirred at RT for 2 hours. The resulting light orange solution was concentrated to about 1 mL under reduced pressure. Addition of *n*‐heptane (10 mL) afforded the precipitation of the product as a yellowish‐white solid that was filtered, washed with a mixture of diethyl ether/*n*‐heptane (5/1 (v/v); 2×2 mL), *n*‐heptane (4×5 mL) and *n*‐pentane (4×5 mL) and dried under reduced pressure. Yield: 189.9 mg (73%); ^1^H NMR (400.1 MHz, toluene‐*d* ^8^, 25 °C): δ = 10.43 (br s, 1H; NH), 8.02–7.86 (m, 1H; Ph), 7.85–7.67 (m, 4H; Ph), 7.65–7.51 (m, 1H; Ph), 7.47–7.27 (m, 2H; Ph), 7.27–6.79 (m, 12H; Ph), 3.94–3.74 (m, 1H; PCH_2_), 2.89–2.74 (m, 1H; PCH_2_), 2.69–2.56 (m, 3H; CH_2_), 2.56–2.41 (m, 3H; CH_2_), 2.38–1.90 (m, 8H; CH_2_), 2.01 (s, 3H; OCOCH_3_), 1.84 (s, 3H; OCOCH_3_), 1.70–1.52 (m, 2H; CH_2_), 1.32 (s, 1H; CH_2_), 1.11 (t, ^3^ *J*(H,H) = 7.2 Hz, 3H; CH_2_C*H*_3_), 1.05 (t, ^3^ *J*(H,H) = 7.2 Hz, 3H; CH_2_C*H*_3_), 0.76–0.57 ppm (br m, 1H; CH_2_); ^13^C{^1^H} NMR (100.6 MHz, toluene‐*d* ^8^, 25 °C): δ = 182.7 (br s; O*C*OCH_3_), 178.4 (s; O*C*OCH_3_), 139.6 (d, ^1^ *J*(C,P) = 14.7 Hz; *ipso*‐Ph), 137.7–124.0 (m; aromatic carbon atoms), 48.7 (s; N*C*H_2_), 40.5 (s; S*C*H_2_), 32.1 (s; S*C*H_2_CH_3_), 28.0 (d, ^1^ *J*(C,P) = 12.5 Hz; P*C*H_2_), 26.1 (d, ^1^ *J*(C,P) = 13.2 Hz; P*C*H_2_), 24.8 (s; OCO*C*H_3_), 24.5 (s; OCO*C*H_3_), 23.8 (s; CH_2_), 21.2 (s; CH_2_), 12.9 ppm (s; CH_2_ *C*H_3_); ^31^P{^1^H} NMR (162.0 MHz, toluene‐*d* ^8^, 25 °C): δ = 37.7 (d, ^2^ *J*(P,P) = 38.1 Hz), 36.5 ppm (d, ^2^ *J*(P,P) = 38.1 Hz); elemental analysis calcd (%) for C_40_H_53_NO_4_P_2_RuS_2_ (839.00): C 57.26, H 6.37, N 1.67, S 7.64; found: C 57.30, H 6.34, N 1.72, S 7.60.

### Synthesis of [Ru(η^1^‐OAc)(CO)(SNS)(PPh_3_)]OAc (5)

[Ru(η^1^‐OAc)(η^2^‐OAc)(CO)(PPh_3_)_2_] (150 mg, 0.194 mmol) and the SNS ligand (41.3 mg, 0.214 mmol, 1.1 equiv) were dissolved in degassed toluene (5 mL), and the mixture was stirred at 80°C for 4 hours. The resulting orange solution was concentrated to about 1 mL under reduced pressure. Addition of *n*‐heptane (10 mL) afforded the precipitation of the product as a yellowish‐white solid that was filtered, washed with a mixture of diethyl ether/*n*‐heptane (4/1 (v/v); 2×2 mL), *n*‐heptane (3×5 mL) and *n*‐pentane (3×5 mL) and dried under reduced pressure. The compound was isolated as a mixture of 3 isomers in 1/0.22/0.15 ratio. Yield: 103.8 mg (76%); ^1^H NMR (400.1 MHz, CD_3_OD, 25 °C): δ = 7.90–7.23 (m, 15H; Ph protons), 3.76–3.61 (m, 2H; NCH_2_ major isomer), 3.54–3.43 (m, 2H; NCH_2_ minor species), 3.27–2.13 (m, 2H; NCH_2_ major isomer), 3.06–2.98 (m, 2H; NCH_2_ minor species), 2.86 (pseudo t, ^3^ *J*(H,H) = 12.4 Hz, 2H; C*H*_2_CH_3_ major isomer), 2.76–2.64 (m, 2H; CH_2_ major isomer), 2.63–2.53 (m, 2H; CH_2_ major isomer), 1.92 (s, 3H; OCOCH_3_ major and minor species), 1.70 (br s, 3H; OCOCH_3_ minor species), 1.63 (br s, 3H; OCOCH_3_ major isomer), 1.55–1.42 (m, 2H; C*H*_2_CH_3_ major isomer), 1.41–1.32 (m, 2H; C*H*_2_CH_3_ minor species), 1.14 (br s, 3H; OCOCH_3_ minor species), 1.16–1.02 (m, 6H; CH_2_C*H*_3_ major isomer), 0.92 ppm (t, ^3^ *J*(H,H) = 6.9 Hz; CH_2_C*H*_3_ minor species); ^1^H NMR (400.1 MHz, toluene‐*d* ^8^, 25 °C): δ = 11.28 (br s, 1H; NH major isomer), 9.15 (br s, 1H; NH minor species), 8.27–6.77 (m, 15H; Ph protons), 4.12–3.88 (m, 2H; NCH_2_ minor species), 3.78 (ddd, ^2^ *J*(H,H) = 15.2 Hz, ^3^ *J*(H,P) = 11.0 Hz, ^3^ *J*(H,H) = 4.1 Hz, 2H; NCH_2_ major isomer), 3.67–3.55 (m, 2H; NCH_2_ minor species), 3.41–3.11 (m, 2H; NCH_2_ minor species), 3.09–2.95 (m, 4H; NCH_2_ and C*H*_2_CH_3_ major isomer), 2.95–2.83 (m, 2H; CH_2_ minor species), 2.80–2.71 (m, 2H; CH_2_ major isomer), 2.70–2.53 (m, 2H; CH_2_ major isomer), 2.49 (*pseudo* q, *J*(H,H) = 7.4 Hz, 2H; CH_2_ minor species), 2.35 (s, 3H; OCOCH_3_), 2.06 (s, 3H; OCOCH_3_ minor species), 1.92 (s, 3H; OCOCH_3_ minor species), 1.76 (s, 3H; OCOCH_3_ major isomer), 1.43–1.16 (m, 2H; C*H*_2_CH_3_ major isomer), 0.94 (t, ^3^ *J*(H,H) = 6.8 Hz; CH_2_C*H*_3_ minor species), 0.77 (t, ^3^ *J*(H,H) = 7.3 Hz, 6H; CH_2_C*H*_3_ major isomer), 0.59 ppm (t, ^3^ *J*(H,H) = 7.3 Hz; CH_2_C*H*_3_ minor species); ^13^C{^1^H} NMR (100.6 MHz, CD_3_OD, 25 °C): δ = 201.4 (d, ^2^ *J*(C,P) = 10.3 Hz; CO major isomer), 197.2 (s; CO minor species), 180.6 (br s; O*C*OCH_3_ major isomer), 178.7 (s; O*C*OCH_3_ major isomer), 177.7 (s; O*C*OCH_3_ minor species), 133.9–128.1 (m; aromatic carbon atoms), 53.2 (s; N*C*H_2_ minor species), 52.7 (s; N*C*H_2_ major isomer), 48.5 (s; N*C*H_2_ minor species), 41.0 (s; S*C*H_2_ minor species), 39.6 (s; S*C*H_2_ minor species), 36.1 (s; S*C*H_2_ major isomer), 35.4 (s; S*C*H_2_CH_3_ minor species), 31.3 (s; S*C*H_2_CH_3_ major isomer), 31.1 (s; S*C*H_2_CH_3_ minor species), 23.8 (s; OCO*C*H_3_ minor species), 23.4 (s; OCO*C*H_3_ major isomer), 22.7 (s; OCO*C*H_3_ major isomer), 21.6 (s; OCO*C*H_3_ minor species), 12.6 ppm (s; CH_2_ *C*H_3_ major isomer), 11.9 ppm (s; CH_2_ *C*H_3_ minor species); ^13^C{^1^H} NMR (100.6 MHz, toluene‐*d* ^8^, 25 °C): δ = 200.8 (s; CO minor species), 197.5 (s; CO major isomer), 176.4 (br s; O*C*OCH_3_ major isomer), 175.7 (s; O*C*OCH_3_ major isomer), 175.0 (s; O*C*OCH_3_ minor species), 137.3–124.2 (m; aromatic carbon atoms), 53.5 (s; N*C*H_2_ minor species), 52.7 (s; N*C*H_2_ minor species), 49.1 (s; N*C*H_2_ major isomer), 40.3 (s; S*C*H_2_ major isomer), 35.1 (s; S*C*H_2_CH_3_ major isomer), 25.0 (s; OCO*C*H_3_ major isomer), 24.3 (s; OCO*C*H_3_ major isomer), 23.1 (s; OCO*C*H_3_ minor species), 14.0  (s; SCH_2_ *C*H_3_ major isomer), 13.5 ppm (s; SCH_2_ *C*H_3_ minor species); ^31^P{^1^H} NMR (162.0 MHz, CD_3_OD, 25 °C): δ = 35.8 (s; major isomer, 73%), 35.7 (br s; minor species, 11%), 31.8 ppm (br s; minor species, 16%); ^31^P{^1^H} NMR (162.0 MHz, toluene‐*d* ^8^, 25 °C): δ = 40.8 (s; minor species, 16%), 37.1 (br s; major isomer, 73%), 33.2 ppm (br s; minor species, 11%); elemental analysis calcd (%) for C_31_H_40_NO_5_PRuS_2_ (702.83): C 52.98, H 5.74, N 1.99, S 9.12; found: C 53.04, H 5.73, N 1.88, S 9.20.

### Synthesis of *Trans*‐[RuH(η^1^‐OAc)(SNS)(PPh_3_)] (6)

Complex **1** (200 mg, 0.296 mmol) was dissolved in degassed toluene (5 mL), and a 0.1 M solution of NaO*^i^* Pr (4.45 mL, 0.445 mmol, 1.5 equiv) in 2‐propanol was added. The mixture was heated for 1 hour at 60°C, and the final solution was concentrated under reduced pressure to almost 3 mL, stirred at room temperature for 30 minutes, and, after addition of toluene (5 mL), filtered on Celite (fine frit). The filtrate was concentrated under reduced pressure (∼ 1 mL), and the addition of *n*‐heptane (10 mL) led to the precipitation of a pale‐yellow product. The solid was filtered, washed with *n*‐heptane (2×2 mL) and *n*‐pentane (2×2 mL), and dried under reduced pressure. The compound was isolated as a mixture of 3 isomers differing by the relative orientation of the SEt groups with respect to the SNS ligand plane. Yield: 173.6 mg (95%); ^1^H NMR (400.1 MHz, toluene‐*d* ^8^, 25 °C): δ = 10.70 (br s, 1H; NH minor isomer), 10.18 (br s, 1H; NH major isomer), 10.06 (br s, 1H; NH minor species), 8.12–7.79 (m, 5H; Ph), 7.41–6.80 (m, 10H; Ph), 3.33–3.08 (m; CH_2_ minor species), 2.89–2.80 (m, 2H; NCH_2_ major isomer), 2.79–2.71 (m, 2H; NCH_2_ major isomer), 2.70–2.54 (m; CH_2_ minor species), 2.53–2.39 (m, 4H; NCH_2_ and C*H*_2_CH_3_ major isomer), 2.29 (br s, 3H; OCOCH_3_ minor species), 2.24 (br s, 3H; OCOCH_3_ minor species), 2.22 (br s, 3H; OCOCH_3_ major isomer), 2.18–2.05 (m; NCH_2_ minor and major isomers), 2.04–1.92 (m; CH_2_ minor species), 1.85–1.72 (m; CH_2_ minor species), 1.61–1.48 (m, 2H; C*H*_2_CH_3_ major isomer), 1.11–0.97 (m; C*H*_2_CH_3_ minor species), 0.95 (t, ^3^ *J*(H,H) = 7.0 Hz; CH_2_C*H*_3_ minor species), 0.76 (t, ^3^ *J*(H,H) = 7.3 Hz, 6H; CH_2_C*H*_3_ major isomer), 0.71–0.62 ppm (m; CH_2_C*H*_3_ minor species), ‐21.54 (d, ^2^ *J*(H,P) = 23.2 Hz, 1H; Ru‐H minor species), ‐21.82 (d, ^2^ *J*(H,P) = 26.2 Hz, 1H; Ru‐H major isomer), ‐21.94 ppm (br d, ^2^ *J*(H,P) = 25.9 Hz, 1H; Ru‐H minor species); ^13^C{^1^H} NMR (100.6 MHz, toluene‐*d* ^8^, 25 °C): δ = 180.7 (s; O*C*OCH_3_ minor species), 180.6 (s; O*C*OCH_3_ minor species), 180.3 (s; O*C*OCH_3_ major isomer), 140.6–124.6 (m; aromatic carbon atoms), 52.8 (s; N*C*H_2_ minor species), 52.3 (s; N*C*H_2_ major isomer), 49.9 (s; N*C*H_2_ minor species), 39.8 (d, ^3^ *J*(C,P) = 3.1 Hz; S*C*H_2_ major isomer), 35.9 (s; S*C*H_2_ minor species), 35.3 (s; S*C*H_2_ minor species), 33.2 (s; S*C*H_2_CH_3_ minor species), 31.6 (s; S*C*H_2_CH_3_ major isomer), 25.7 (s; OCO*C*H_3_ minor species), 25.3 (s; OCO*C*H_3_ major isomer), 13.8 (s; CH_2_ *C*H_3_ minor species), 13.6 (s; CH_2_ *C*H_3_ minor species), 13.0 ppm (s; CH_2_ *C*H_3_ major isomer); ^31^P{^1^H} NMR (162.0 MHz, toluene‐*d* ^8^, 25 °C): δ = 65.7 (s; 11%), 62.5 (s; 33%), 60.6 ppm (s; 67%); elemental analysis calcd (%) for C_28_H_38_NO_2_PRuS_2_ (616.78): C 54.53, H 6.21, N 2.27, S 10.40; found: C 54.60, H 6.15, N 2.26, S 10.45.

### Typical procedure for HY of methyl decanoate

The HY reactions for the reduction of methyl decanoate were performed in an 8 vessels Endeavor Biotage apparatus employing the ruthenium SNS carboxylates **1**–**6** and *trans*‐[RuCl_2_(SNS)(PPh_3_)]. The catalysts were added to the reactor from appropriate volumes of stock solutions of the complexes prepared by dissolving the required mass of the catalyst in CH_2_Cl_2_ (1.0–4.0 mL), aliquots of which were dispensed into 8 different vials. The solvent was then removed under the flow of N_2_. To each of these containers, NaOMe or NaOEt (50 mol% with respect to the substrate) was added, followed by the substrate (10‐20 mmol, 2.1–4.3 mL). For the experiments carried out with solvent, toluene was added (one volume with respect to the substrate, 2.1–4.3 mL). The vials were then sealed inside of the instrument, where the vessels were purged 5 times with N_2_ before running an automated purging cycle with H_2_, then the vessels were charged with H_2_ (27.5 bar), heated to 40°C or 90°C and stirred (600 rpm) for the required time (16 hours). The reactions were then cooled to room temperature, depressurized, and purged with nitrogen five times. Two work‐up procedures were used, prior to analysis: a) adding EtOH (4 mL), followed by 1.0 M HCl (3 mL) or b) adding MTBE (3 mL), followed by 1.0 M HCl (3 mL) and extraction of the product in the organic phase. The obtained solutions were sampled, diluted with EtOH or MTBE (1 mL), depending on the work‐up used, and analyzed by GC.

### Typical procedure for HY of methyl and ethyl esters

The HY reductions of a series of methyl and ethyl esters were performed in an 8 vessels Endeavor Biotage apparatus employing the catalysts **1**, **2,** and *trans*‐[RuCl_2_(SNS)(PPh_3_)], following the procedure used for methyl decanoate.

### Synthesis of 1‐decanol by large‐scale HY of ethyl decanoate

The HY of ethyl decanoate on a laboratory scale (107.6 mmol, 21.55 g, 25 mL) was performed in a 50 mL Parr reactor. The substrate was added to the vessel, followed by NaOEt (53.79 mmol, 3.66 g) and catalyst **1** (1.45 mg, 2.16 µmol) from a 5.84 mg/mL solution in EtOH (250 µL, 1 vol%). The S/C molar ratio was 50, 000, whereas the base concentration was 50 mol% with respect to the substrate. A Parr High Pressure Burette system (650 mL) was connected to the reactor with a high‐pressure hose, fitted with valves so that it can be sealed off from both the hydrogen supply and the autoclave. The line was then purged several times with nitrogen, and three times with hydrogen, followed by slowly filling the Parr reactor to the desired pressure (28‐29 bar) with H_2_, and disconnected to the hydrogen source. The reaction was then set to stir at 1300 rpm, and the reactor slowly heated to 45°C (within about 20 minutes) using a model 4838 Parr Temperature Controller. The reaction mixture was stirred at this temperature for 16 hours. At the end of the reaction, the reactor was cooled to room temperature, the excess hydrogen was vented, and the product was purged with nitrogen five times. The final waxy compound was treated with 1.0 M HCl (50 mL), extracted with MTBE (40 mL) and dried with anhydrous Na_2_SO_4_. The product was isolated and separated from the remaining catalyst by carrying out a short filtration over a plug of silica gel and evaporation of the volatiles, leading to 1‐decanol in 95% yield (16.16 g) that was analyzed by NMR and GC (99% purity).

### Synthesis of oleyl alcohol by large‐scale HY of ethyl oleate

Oleyl alcohol was prepared on a larger scale by following the procedure used for the synthesis of 1‐decanol, with ethyl oleate (69.40 mmol, 21.50 g, 25 mL) in a 50 mL Parr reactor, employing the catalyst **1** (0.94 mg, 1.39 µmol) from a 3.75 mg/mL solution in EtOH (250 µL, 1 vol%). The S/C molar ratio was 50, 000. A similar result was also obtained with an S/C ratio of 100, 000 using the appropriate amounts of catalyst **1**. The stirring rate of the reaction mixture was 1400 rpm, and the reactor was slowly heated to 45°C. The hydrogen pressure was 28 bar, and the reaction time was 16 hours. After work‐up, in the same conditions previously described for 1‐decanol, oleyl alcohol was recovered in 92% yield (17.14 g) and analyzed by NMR and GC (99.9% purity). The final product contains oleyl alcohol (>78%) and other alcohols (linoleyl alcohol, palmityl alcohol, etc) because the starting material was of technical grade and other esters were present together with ethyl oleate.

### X‐ray crystallography

Single crystals of complex **1** were obtained by slow evaporation of acetone/diethyl ether solutions, whereas **3** crystallizes from toluene/diethyl ether. Crystals of **5** were formed by slow evaporation of a CH_2_Cl_2_, diethyl ether and *n*‐pentane mixture solution. X‐ray diffraction data were collected at 100 K on an X‐ray single crystal Bruker D8 VENTURE Duo three‐angle diffractometer with either a TXS rotating anode or with an IMS microsource with MoK_α_ radiation (*λ* = 0.71 073 Å) using the APEX4 software.^[^ ^37^ ^]^ The diffractometer was equipped with a Helios optic monochromator, a Bruker PHOTON II detector, and an Oxford Cryostream low‐temperature device. Deposition Numbers 2 449 278 (for **1**) and 2 449 279 (for **3**) contain the supplementary crystallographic data for this paper. These data are provided free of charge by the joint Cambridge Crystallographic Data Centre and Fachinformationszentrum Karlsruhe Access Structures service http://www.ccdc.cam.ac.uk/structures.^[^ ^38^ ^]^ For additional details on the collection and refining of data, see the Supporting Information. NMR data of the isolated complexes, X‐ray crystallographic details of complexes **1** and **3**, and further data for the HY of esters catalyzed by the ruthenium derivatives are available in Supplementary Information.

## Conflict of Interest

The authors declare no conflict of interest.

## Supporting information



Supporting Information

## Data Availability

The data that support the findings of this study are available from the corresponding author upon reasonable request.
