# Supplementary material for: Productive Homogeneous Hydrogenation of Fatty Esters with Carboxylate SNS Ruthenium Catalysts
Source: Chemistry. 2025 Jul 16;31(44):e202501898. doi: 10.1002/chem.202501898 (PMC12336773; doi:10.1002/chem.202501898)
Supplement: Supplementary file 1 — Supporting Information [file CHEM-31-e202501898-s001.pdf]

Chemistry—A European Journal

Supporting Information

Table of Contents:

Figure S1. $^{31}P\{^{1}H\}$ NMR spectrum of *trans*-[Ru(η^1-OAc)$_2$(SNS)(PPh$_3$)] (**1**) Pag. S14

Figure S2. ^{1}H NMR spectrum of *trans*-[Ru(η^1-OAc)$_2$(SNS)(PPh$_3$)] (**1**) Pag. S15

Figure S3. $^{13}C\{^{1}H\}$ DEPTQ NMR spectrum of *trans*-[Ru(η^1-OAc)$_2$(SNS)(PPh$_3$)] (**1**) Pag. S16

Figure S4. ^{1}H-^{1}H COSY 2D NMR spectrum of *trans*-[Ru(η^1-OAc)$_2$(SNS)(PPh$_3$)] (**1**) Pag. S17

Figure S5. ^{1}H-^{13}C HSQC 2D NMR spectrum of *trans*-[Ru(η^1-OAc)$_2$(SNS)(PPh$_3$)] (**1**) Pag. S18

Figure S6. ^{1}H-^{13}C HMBC 2D NMR spectrum of *trans*-[Ru(η^1-OAc)$_2$(SNS)(PPh$_3$)] (**1**) Pag. S19

Figure S7. ^{1}H-^{31}P HMBC 2D NMR spectrum of *trans*-[Ru(η^1-OAc)$_2$(SNS)(PPh$_3$)] (**1**) Pag. S20

Figure S8. ^{1}H-^{1}H NOESY 2D NMR spectrum of *trans*-[Ru(η^1-OAc)$_2$(SNS)(PPh$_3$)] (**1**) Pag. S21

Figure S9. ^{1}H-^{15}N HSQC 2D NMR spectrum of *trans*-[Ru(η^1-OAc)$_2$(SNS)(PPh$_3$)] (**1**) Pag. S22

Figure S10. $^{31}P\{^{1}H\}$ NMR spectrum of *trans*-[Ru(η^1-Piv)$_2$(SNS)(PPh$_3$)] (**2**) Pag. S23

Figure S11. ^{1}H NMR spectrum of *trans*-[Ru(η^1-Piv)$_2$(SNS)(PPh$_3$)] (**2**) Pag. S24

Figure S12. $^{13}C\{^{1}H\}$ DEPTQ NMR spectrum of *trans*-[Ru(η^1-Piv)$_2$(SNS)(PPh$_3$)] (**2**) Pag. S25

Figure S13. ^{1}H-^{1}H COSY 2D NMR spectrum of *trans*-[Ru(η^1-Piv)$_2$(SNS)(PPh$_3$)] (**2**) Pag. S26

Figure S14. ^{1}H-^{13}C HSQC 2D NMR spectrum of *trans*-[Ru(η^1-Piv)$_2$(SNS)(PPh$_3$)] (**2**) Pag. S27

Figure S15. ^{1}H-^{13}C HMBC 2D NMR spectrum of *trans*-[Ru(η^1-Piv)$_2$(SNS)(PPh$_3$)] (**2**) Pag. S28

Figure S16. ^{1}H-^{31}P HMBC 2D NMR spectrum of *trans*-[Ru(η^1-Piv)$_2$(SNS)(PPh$_3$)] (**2**) Pag. S29

Figure S17. ^{1}H-^{1}H NOESY 2D NMR spectrum of *trans*-[Ru(η^1-Piv)$_2$(SNS)(PPh$_3$)] (**2**) Pag. S30

Figure S18. $^{31}P\{^{1}H\}$ NMR spectrum of *trans*-[Ru(η^1-OAc)$_2$(SNS)(PPh$_2$Me)] (**3**) Pag. S31

Figure S19. ^{1}H NMR spectrum of *trans*-[Ru(η^1-OAc)$_2$(SNS)(PPh$_2$Me)] (**3**) Pag. S32

Figure S20. $^{13}C\{^{1}H\}$ DEPTQ NMR spectrum of *trans*-[Ru(η^1-OAc)$_2$(SNS)(PPh$_2$Me)] (**3**) Pag. S33

Figure S21. ^{1}H-^{1}H COSY 2D NMR spectrum of *trans*-[Ru(η^1-OAc)$_2$(SNS)(PPh$_2$Me)] (**3**) Pag. S34

Figure S22. ^1H-^{13}C HSQC 2D NMR spectrum of *trans*-[Ru(η^1-OAc)$_2$(SNS)(PPh$_2$Me)] (**3**)

Pag. S35

Figure S23. ^1H-^{13}C HMBC 2D NMR spectrum of *trans*-[Ru(η^1-OAc)$_2$(SNS)(PPh$_2$Me)] (**3**)

Pag. S36

Figure S24. ^1H-^{31}P HMBC 2D NMR spectrum of *trans*-[Ru(η^1-OAc)$_2$(SNS)(PPh$_2$Me)] (**3**)

Pag. S37

Figure S25. ^1H-^1H NOESY 2D NMR spectrum of *trans*-[Ru(η^1-OAc)$_2$(SNS)(PPh$_2$Me)] (**3**)

Pag. S38

Figure S26. ^1H-^{15}N HSQC 2D NMR spectrum of *trans*-[Ru(η^1-OAc)$_2$(SNS)(PPh$_2$Me)] (**3**)

Pag. S39

Figure S27. $^{31}P\{^1H\}$ NMR spectrum of *cis*-[Ru(η^1-OAc)(SNS)(dppb)] (**4**) Pag. S40

Figure S28. ^1H NMR spectrum of *cis*-[Ru(η^1-OAc)(SNS)(dppb)] (**4**) Pag. S41

Figure S29. $^{13}C\{^1H\}$ DEPTQ NMR spectrum of *cis*-[Ru(η^1-OAc)(SNS)(dppb)] (**4**) Pag. S42

Figure S30. ^1H-^1H COSY 2D NMR spectrum of *cis*-[Ru(η^1-OAc)(SNS)(dppb)] (**4**) Pag. S43

Figure S31. ^1H-^{13}C HSQC 2D NMR spectrum of *cis*-[Ru(η^1-OAc)(SNS)(dppb)] (**4**) Pag. S44

Figure S32. ^1H-^{13}C HMBC 2D NMR spectrum of *cis*-[Ru(η^1-OAc)(SNS)(dppb)] (**4**) Pag. S45

Figure S33. ^1H-^{31}P HMBC 2D NMR spectrum of *cis*-[Ru(η^1-OAc)(SNS)(dppb)] (**4**) Pag. S46

Figure S34. ^1H-^1H NOESY 2D NMR spectrum of *cis*-[Ru(η^1-OAc)(SNS)(dppb)] (**4**) Pag. S47

Figure S35. $^{31}P\{^1H\}$ NMR spectrum of [Ru(η^1-OAc)(CO)(SNS)(PPh$_3$)]OAc (**5**) in CD_3OD

Pag. S48

Figure S36. $^{31}P\{^1H\}$ NMR spectrum of [Ru(η^1-OAc)(CO)(SNS)(PPh$_3$)]OAc (**5**) in toluene-d^8

Pag. S49

Figure S37. ^1H NMR spectrum of [Ru(η^1-OAc)(CO)(SNS)(PPh$_3$)]OAc (**5**) in CD_3OD

Pag. S50

Figure S38. ^1H NMR spectrum of [Ru(η^1-OAc)(CO)(SNS)(PPh$_3$)]OAc (**5**) in toluene-d^8

Pag. S51

Figure S39. $^{13}C\{^1H\}$ DEPTQ NMR spectrum of [Ru(η^1-OAc)(CO)(SNS)(PPh_3)]OAc (**5**) in CD_3OD Pag. S52

Figure S40. $^{13}C\{^1H\}$ DEPTQ NMR spectrum of [Ru(η^1-OAc)(CO)(SNS)(PPh_3)]OAc (**5**) in toluene-*d*^{8} Pag. S53

Figure S41. ^1H-^1H COSY 2D NMR spectrum of [Ru(η^1-OAc)(CO)(SNS)(PPh_3)]OAc (**5**) Pag. S54

Figure S42. ^1H-^{13}C HSQC 2D NMR spectrum of [Ru(η^1-OAc)(CO)(SNS)(PPh_3)]OAc (**5**) Pag. S55

Figure S43. ^1H-^{13}C HMBC 2D NMR spectrum of [Ru(η^1-OAc)(CO)(SNS)(PPh_3)]OAc (**5**) Pag. S56

Figure S44. ^1H-^{31}P HMBC 2D NMR spectrum of [Ru(η^1-OAc)(CO)(SNS)(PPh_3)]OAc (**5**) Pag. S57

Figure S45. ^1H-^1H NOESY 2D NMR spectrum of [Ru(η^1-OAc)(CO)(SNS)(PPh_3)]OAc (**5**) Pag. S58

Figure S46. $^{31}P\{^1H\}$ NMR spectrum of *trans*-[RuH(η^1-OAc)(SNS)(PPh_3)] (**6**) Pag. S59

Figure S47. ^1H NMR spectrum of *trans*-[RuH(η^1-OAc)(SNS)(PPh_3)] (**6**) Pag. S60

Figure S48. $^{13}C\{^1H\}$ DEPTQ NMR spectrum of *trans*-[RuH(η^1-OAc)(SNS)(PPh_3)] (**6**) Pag. S61

Figure S49. ^1H-^1H COSY 2D NMR spectrum of *trans*-[RuH(η^1-OAc)(SNS)(PPh_3)] (**6**) Pag. S62

Figure S50. ^1H-^{13}C HSQC 2D NMR spectrum of *trans*-[RuH(η^1-OAc)(SNS)(PPh_3)] (**6**) Pag. S63

Figure S51. ^1H-^{13}C HMBC 2D NMR spectrum of *trans*-[RuH(η^1-OAc)(SNS)(PPh_3)] (**6**) Pag. S64

Figure S52. ^1H-^{31}P HMBC 2D NMR spectrum of *trans*-[RuH(η^1-OAc)(SNS)(PPh_3)] (**6**) Pag. S65

Figure S53. ^{1}H-^{1}H NOESY 2D NMR spectrum of *trans*-[RuH(η^1-OAc)(SNS)(PPh_3)] (**6**)

Pag. S66

Figure S54. ^{1}H-^{15}N HSQC 2D NMR spectrum of *trans*-[RuH(η^1-OAc)(SNS)(PPh_3)] (**6**)

Pag. S67

Table S1. Further data regarding the catalytic HY of methyl decanoate **c** (2-20 mmol) with complexes **1-6** (S/C = 1000) and with different bases (50 mol%) at 28 bar of H_2 in presence of different solvents

Pag. S68

Figure S55. ^{31}P{^{1}H} NMR spectrum of the mixture of ruthenium hydrides obtained by reaction of *trans*-[Ru(η^1-OAc)$_2$(SNS)(PPh_3)] (**1**) with H_2 (5 bar) and KO^{t}Bu (3 equiv) after heating at 70 °C for 5 h Pag. S69

Figure S56. Effect of heating on the formation of the mixture of ruthenium hydrides by reaction of *trans*-[Ru(η^1-OAc)$_2$(SNS)(PPh_3)] (**1**) with H_2 (5 bar) and KO^{t}Bu (3 equiv) in the ^{31}P{^{1}H} NMR spectra Pag. S70

Figure S57. ^{1}H NMR spectrum of the mixture of ruthenium hydrides obtained by reaction of *trans*-[Ru(η^2-OAc)$_2$(SNS)(PPh_3)] (**1**) with H_2 (5 bar) and KO^{t}Bu (3 equiv) after heating at 70 °C for 12 h

Pag. S71

Figure S58. ^{1}H-^{31}P HMBC 2D NMR spectrum of the mixture of ruthenium hydrides obtained by reaction of *trans*-[Ru(η^1-OAc)$_2$(SNS)(PPh_3)] (**1**) with H_2 (5 bar) and KO^{t}Bu (3 equiv) after heating at 70 °C for 12 h Pag. S72

Figure S59. ^{1}H-^{1}H COSY 2D NMR spectrum of the mixture of ruthenium hydrides obtained by reaction of *trans*-[Ru(η^1-OAc)$_2$(SNS)(PPh_3)] (**1**) with H_2 (5 bar) and KO^{t}Bu (3 equiv) after heating at 70 °C for 12 h Pag. S73

Figure S60. ^1H-^1H NOESY 2D NMR spectrum of the mixture of ruthenium hydrides obtained by reaction of *trans*-[Ru(η^1-OAc)$_2$(SNS)(PPh$_3$)] (**1**) with H_2 (5 bar) and KO^{t}Bu (3 equiv) after heating at 70 °C for 12 h Pag. S74

Figure S61. ^1H-^1H NOESY 2D NMR spectrum of the mixture of ruthenium hydrides obtained by reaction of *trans*-[Ru(η^1-OAc)$_2$(SNS)(PPh$_3$)] (**1**) with H_2 (5 bar) and KO^{t}Bu (3 equiv) after heating at 70 °C for 12 h (region 0.0-4.0 ppm) Pag. S75

Figure S62. ^1H NMR spectrum after the addition of PhCOOMe (5 equiv) to the mixture of ruthenium hydrides (obtained by reaction of *trans*-[Ru(η^1-OAc)$_2$(SNS)(PPh$_3$)] (**1**) with H_2 and KO^{t}Bu), at RT in H_2 (5 bar) Pag. S76

Figure S63. ^1H NMR spectrum after the addition of PhCOOMe (5 equiv) to the mixture of ruthenium hydrides (obtained by reaction of *trans*-[Ru(η^1-OAc)$_2$(SNS)(PPh$_3$)] (**1**) with H_2 and KO^{t}Bu), after heating at 70 °C for 5 h Pag. S77

Figure S64. Effect of heating (70 °C for 5 h) on the ^1H NMR spectrum of the mixture of ruthenium hydrides (obtained by reaction of *trans*-[Ru(η^1-OAc)$_2$(SNS)(PPh$_3$)] (**1**) with H_2 and KO^{t}Bu) after the addition of PhCOOMe (5 equiv) in H_2 (5 bar) Pag. S78

Figure S65. Effect of addition of 2-propanol (10 equiv) to the mixture of ruthenium hydrides (obtained by reaction of *trans*-[Ru(η^1-OAc)$_2$(SNS)(PPh$_3$)] (**1**) with H_2 and KO^{t}Bu) on the $^{31}P\{^1H\}$ NMR spectra Pag. S79

Figure S66. Effect of heating in the formation of the mixture of ruthenium hydrides by reaction of *trans*-[RuCl$_2$(SNS)(PPh$_3$)] with H_2 (5 bar) and KO^{t}Bu (3 equiv) in the $^{31}P\{^1H\}$ NMR spectra Pag. S80

Figure S67. $^{31}P\{^1H\}$ NMR spectrum of the mixture of ruthenium monohydrides obtained by reaction of *trans*-[Ru(η^1-OAc)$_2$(SNS)(PPh$_3$)] (**1**) with H_2 (5 bar) and DBU (3 equiv) after heating at 100 °C for 15 days Pag. S81

Figure S68. ^1H NMR spectrum of the mixture of ruthenium monohydrides obtained by reaction of *trans*-[Ru(η^1-OAc)$_2$(SNS)(PPh$_3$)] (**1**) with H_2 (5 bar) and DBU (3 equiv) after heating at 100 °C for 15 days Pag. S82

Figure S69. $^{31}P\{^1H\}$ NMR spectrum of the mixture of ruthenium hydrides obtained by reaction of *trans*-[Ru(η^1-OAc)$_2$(SNS)(PPh$_3$)] (**1**) with H_2 (5 bar) and DIBAL-H (2 equiv or 1 equiv as dimer) after heating at 70 °C for 4 h Pag. S83

Figure S70. ^1H NMR spectrum of the mixture of ruthenium hydrides obtained by reaction of *trans*-[Ru(η^1-OAc)$_2$(SNS)(PPh$_3$)] (**1**) with H_2 (5 bar) and DIBAL-H (2 equiv or 1 equiv as dimer) after heating at 70 °C for 4 h Pag. S84

Figure S71. $^{31}P\{^1H\}$ NMR spectrum of the mixture of ruthenium hydrides obtained by reaction of *trans*-[Ru(η^1-OAc)$_2$(SNS)(PPh$_3$)] (**1**) with H_2 (5 bar) and DIBAL-H (6 equiv or 3 equiv as dimer) after heating at 70 °C for 1 h Pag. S85

Figure S72. ^1H NMR spectrum of the mixture of ruthenium hydrides obtained by reaction of *trans*-[Ru(η^1-OAc)$_2$(SNS)(PPh$_3$)] (**1**) with H_2 (5 bar) and DIBAL-H (6 equiv or 3 equiv as dimer) after heating at 70 °C for 1 h Pag. S86

Figure S73. $^{31}P\{^1H\}$ NMR spectrum of the mixture of ruthenium hydrides obtained by reaction of [Ru(η^1-OAc)(CO)(SNS)(PPh$_3$)]OAc (**5**) with H_2 (5 bar) and KO^{t}Bu (3 equiv) after heating at 70 °C for 2 h Pag. S87

Figure S74. ^{1}H NMR spectrum of the mixture of ruthenium hydrides obtained by reaction of [Ru(η^1-OAc)(CO)(SNS)(PPh_3)]OAc (**5**) with H_2 (5 bar) and KO^{t}Bu (3 equiv) after heating at 70 °C for 2 h Pag. S88

Figure S75. ^{1}H-^{13}C HMBC 2D NMR spectrum of the mixture of ruthenium hydrides obtained by reaction of [Ru(η^1-OAc)(CO)(SNS)(PPh_3)]OAc (**5**) with H_2 (5 bar) and KO^{t}Bu (3 equiv) after heating at 70 °C for 2 h Pag. S89

Figure S76. ^{1}H-^{31}P HMBC 2D NMR spectrum of the mixture of ruthenium hydrides obtained by reaction of [Ru(η^1-OAc)(CO)(SNS)(PPh_3)]OAc (**5**) with H_2 (5 bar) and KO^{t}Bu (3 equiv) after heating at 70 °C for 2 h Pag. S90

Figure S77. Hydrogen uptake curve of complexes **1-6** and the *trans*-[RuCl$_2$(SNS)(PPh_3)] derivative for the solvent-free reduction of methyl decanoate **a** at 90 °C (S/C 10000) Pag. S91

Figure S78. Hydrogen uptake curve of complexes **1**, **2**, **4-6** and the *trans*-[RuCl$_2$(SNS)(PPh_3)] derivative for the solvent-free reduction of methyl decanoate **a** at 40 °C (S/C 50000) Pag. S91

Figure S79. Hydrogen uptake curve of complexes **1-3**, **5** and the *trans*-[RuCl$_2$(SNS)(PPh_3)] derivative for the solvent-free reduction of methyl decanoate **a** at 90 °C (S/C 50000) Pag. S92

Figure S80. Hydrogen uptake curve of complex **2** and the *trans*-[RuCl$_2$(SNS)(PPh_3)] derivative for the reduction of methyl decanoate **a** at 40 °C (S/C 50000) and toluene as solvent Pag. S92

Figure S81. Hydrogen uptake curve of complex **1** and the *trans*-[RuCl$_2$(SNS)(PPh_3)] derivative for the solvent-free reduction of methyl decanoate **a** at 40 °C (S/C 100000) Pag. S93

Figure S82. Hydrogen uptake curve of the *trans*-[RuCl$_2$(SNS)(PPh_3)] derivative for the solvent-free reduction of methyl decanoate **a** at 40 °C, using different bases (S/C 100000) Pag. S93

Figure S83. Hydrogen uptake curve of complexes **1**, **2** and the *trans*-[$RuCl_2$(SNS)(PPh_3)] derivative for the solvent-free reduction of ethyl decanoate **b** at 40 °C (S/C 50000) Pag. S94

Figure S84. Hydrogen uptake curve of complex **1** and the *trans*-[$RuCl_2$(SNS)(PPh_3)] derivative for the solvent-free reduction of ethyl decanoate **b** at 40 °C (S/C 100000) Pag. S94

Figure S85. Hydrogen uptake curve of complex **1** and the *trans*-[$RuCl_2$(SNS)(PPh_3)] derivative for the solvent-free reduction of ethyl dodecanoate **c** at 40 °C (S/C 44000) Pag. S95

Figure S86. Hydrogen uptake curve of complex **1** and the *trans*-[$RuCl_2$(SNS)(PPh_3)] derivative for the solvent-free reduction of ethyl dodecanoate **c** at 40 °C (S/C 100000) Pag. S95

Figure S87. Hydrogen uptake curve of complex **1** and the *trans*-[$RuCl_2$(SNS)(PPh_3)] derivative for the solvent-free reduction of ethyl 10-undecenoate **d** at 40 °C (S/C 10000) Pag. S96

Figure S88. Hydrogen uptake curve of complex **1** and the *trans*-[$RuCl_2$(SNS)(PPh_3)] derivative for the solvent-free reduction of ethyl 10-undecenoate **d** at 30 °C (S/C 10000) Pag. S96

Figure S89. Hydrogen uptake curve of complex **1** and the *trans*-[$RuCl_2$(SNS)(PPh_3)] derivative for the solvent-free reduction of ethyl 10-undecenoate **d** at 40 °C (S/C 50000) Pag. S97

Figure S90. Hydrogen uptake curve of complex **1** and the *trans*-[$RuCl_2$(SNS)(PPh_3)] derivative for the solvent-free reduction of ethyl 10-undecenoate **d** at 40 °C (S/C 100000) Pag. S97

Figure S91. Hydrogen uptake curve of complex **1** and the *trans*-[$RuCl_2$(SNS)(PPh_3)] derivative for the solvent-free reduction of methyl oleate **e** at 60 °C (S/C 25000) Pag. S98

Figure S92. Hydrogen uptake curve of complex **1** and the *trans*-[$RuCl_2$(SNS)(PPh_3)] derivative for the solvent-free reduction of methyl oleate **e** at 50 °C (S/C 50000) Pag. S98

Figure S93. Hydrogen uptake curve of complex **1** and the *trans*-[$RuCl_2$(SNS)(PPh_3)] derivative for the solvent-free reduction of methyl oleate **e** at 50 °C (S/C 100000) Pag. S99

Figure S94. Hydrogen uptake curve of complex **1** and the *trans*-[$RuCl_2$(SNS)(PPh_3)] derivative for the solvent-free reduction of ethyl oleate **f** at 40 °C (S/C 50000) Pag. S99

Figure S95. Hydrogen uptake curve of complex **1** and the *trans*-[$RuCl_2$(SNS)(PPh_3)] derivative for the solvent-free reduction of ethyl oleate **f** at 40 °C (S/C 100000) Pag. S100

Figure S96. Hydrogen uptake curve of complex **1** and the *trans*-[$RuCl_2$(SNS)(PPh_3)] derivative for the solvent-free reduction of ethyl oleate **f** at 50 °C (S/C 100000) Pag. S100

Figure S97. Hydrogen uptake curve of complex **1** and the *trans*-[$RuCl_2$(SNS)(PPh_3)] derivative for the solvent-free reduction of methyl benzoate **g** at 40 °C (S/C 100000) Pag. S101

Figure S98. Hydrogen uptake curve of complex **1** and the *trans*-[$RuCl_2$(SNS)(PPh_3)] derivative for the solvent-free reduction of ethyl benzoate **h** at 40 °C (S/C 50000) Pag. S101

Figure S99. Hydrogen uptake curve of complexes **1**, **2** and the *trans*-[$RuCl_2$(SNS)(PPh_3)] derivative for the solvent-free reduction of ethyl benzoate **h** at 40 °C (S/C 100000) Pag. S102

GC analytical method for methyl and ethyl decanoate hydrogenation Pag. S103

Figure S100. Representative example of GC chromatogram of the catalytic HY of methyl decanoate **a** using RuSNS complexes, containing all the species involved in the reaction Pag. S104

Figure S101. GC trace of products of methyl decanoate **a** hydrogenation catalysed by complex **1** (S/C 50000), showing full conversion to 1-decanol Pag. S104

Figure S102. Representative example of GC chromatogram of the catalytic HY of ethyl decanoate **b** using RuSNS complexes, containing all the species involved in the reaction Pag. S105

Figure S103. GC trace of products of ethyl decanoate **b** hydrogenation catalysed by complex **1** (S/C 50000), showing full conversion to 1-decanol Pag. S105

GC analytical method for ethyl dodecanoate hydrogenations Pag. S106

Figure S104. Representative example of GC chromatogram of the catalytic HY of ethyl dodecanoate **c** using RuSNS complexes, containing all the species involved in the reaction Pag. S107

Figure S105. GC trace of products of ethyl dodecanoate **c** hydrogenation catalysed by complex **1** (S/C 50000), showing full conversion to 1-dodecanol Pag. S107

GC analytical method for ethyl 10-undecenoate hydrogenations Pag. S108

Figure S106. Representative example of GC chromatogram of the catalytic HY of ethyl 10-undecenoate **d** using RuSNS complexes, containing all the species involved in the reaction

Pag. S109

Figure S107. GC trace of products of ethyl 10-undecenoate **d** hydrogenation catalysed by complex **1** (S/C 10000), showing full conversion to 10-undecen-1-ol Pag. S109

GC analytical method for methyl and ethyl oleate hydrogenations Pag. S110

Figure S108. Representative GC trace of starting material reference for technical grade methyl oleate **e**, showing composition (%) as a ratio of peak areas Pag. S111

Figure S109. Representative GC trace of hydrogenation products of methyl oleate **e** catalysed by complex **1** (S/C 25000) Pag. S111

Figure S110. GC trace of starting material reference for technical grade ethyl oleate **f**, showing composition (%) as a ratio of peak areas Pag. S112

Figure S111. GC trace of hydrogenation products of ethyl oleate **f** catalyzed by complex **1** (S/C 50000) Pag. S112

GC analytical method for methyl and ethyl benzoate hydrogenations Pag. S113

Figure S112. Representative example of GC chromatogram of the catalytic HY of methyl benzoate **g** using RuSNS complexes containing all the species involved in the reaction Pag. S114

Figure S113. GC trace of products of methyl benzoate **g** hydrogenation catalysed by complex **1** (S/C 10000), showing full conversion to benzyl alcohol Pag. S114

Figure S114. Representative example of GC chromatogram of the catalytic HY of ethyl benzoate **h** using RuSNS complexes containing all the species involved in the reaction Pag. S115

Figure S115. GC trace of products of ethyl benzoate **h** hydrogenation catalysed by complex **1** (S/C 100000), showing full conversion to benzyl alcohol Pag. S115

Synthesis of 1-decanol by large scale hydrogenation of ethyl decanoate Pag. S116

Figure S116. GC trace of products of ethyl decanoate **b** hydrogenation in multigram scale, promoted by complex **1** at S/C 50000 in neat conditions Pag. S117

Figure S117. ^{1}H NMR spectrum of 1-decanol obtained by catalytic hydrogenation of ethyl decanoate **b** in multigram scale, promoted by complex **1** at S/C 50000 in neat conditions Pag. S118

Figure S118. ^{13}C{^{1}H} NMR spectrum of 1-decanol obtained by catalytic hydrogenation of ethyl decanoate **b** in multigram scale, promoted by complex **1** at S/C 50000 in neat conditions

Pag. S119

Synthesis of oleyl alcohol by large scale hydrogenation of ethyl oleate Pag. S120

Figure S119. GC trace of products of ethyl oleate **f** hydrogenation in multigram scale, promoted by complex **1** at S/C 50000 in neat conditions Pag. S121

Figure S120. ^{1}H NMR spectrum of ethyl oleate **f** hydrogenation products in multigram scale, promoted by complex **1** at S/C 50000 in neat conditions Pag. S122

Figure S121. ^{13}C{^{1}H} NMR spectrum of ethyl oleate **f** hydrogenation products in multigram scale, promoted by complex **1** at S/C 50000 in neat conditions Pag. S123

Single crystal X-ray structure determination of Compounds 1 and 3. General data.

Pag. S124

Figure S122. Molecular structure of complex **1** Pag. S125

Single crystal X-ray structure determination of complex 1. Detailed crystallographic data.

Pag. S126

Figure S123. Molecular structure of complex **3** Pag. S128

Single crystal X-ray structure determination of complex 3. Detailed crystallographic data.

Pag. S129

Figure S124. Molecular structure of complex **5** Pag. S131

References Pag. S132

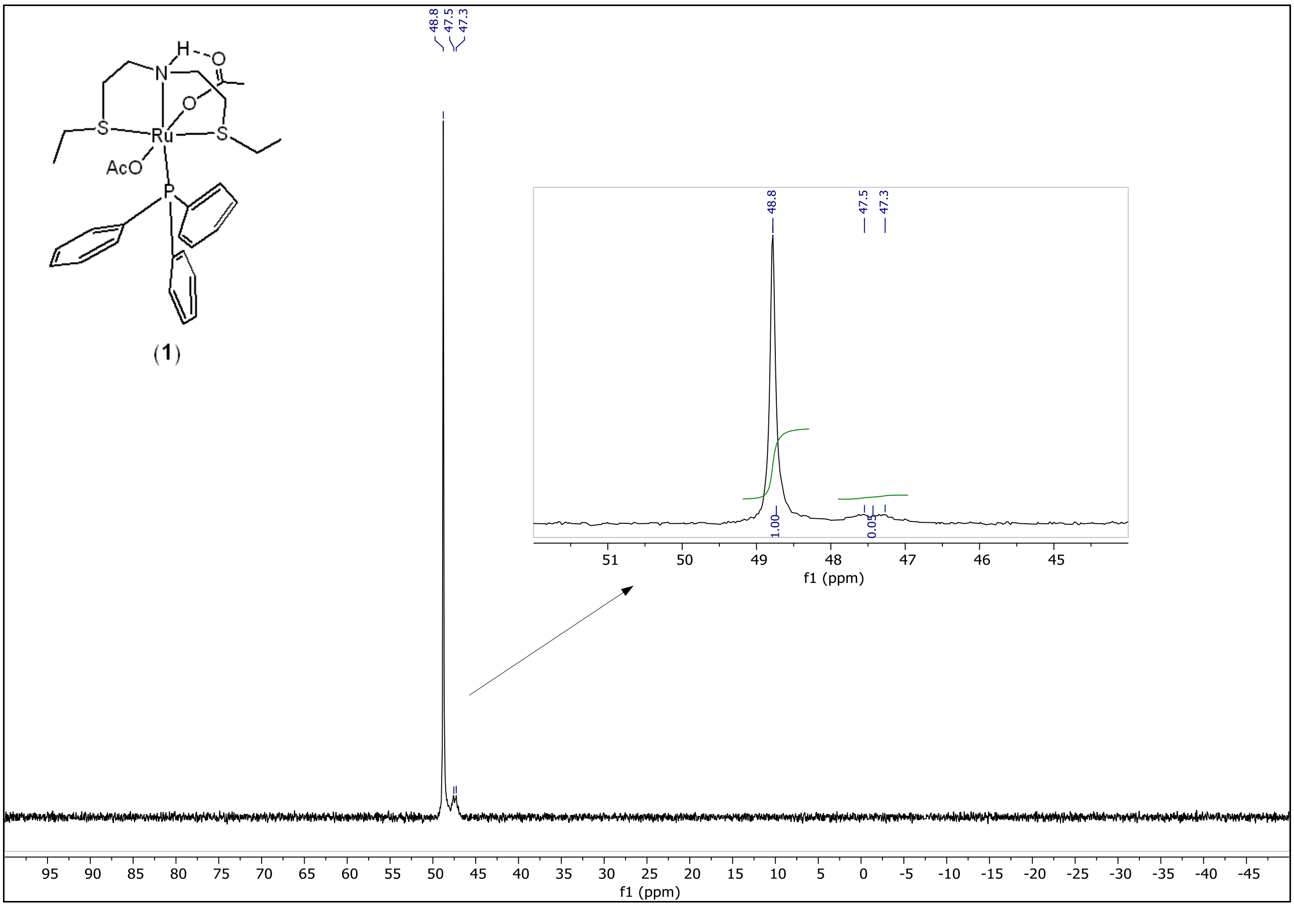

Figure S1. $^{31}P\{^{1}H\}$ NMR spectrum (162.0 MHz) of *trans*-[Ru(η^1-OAc)$_2$(SNS)(PPh$_3$)] (**1**) in toluene-d^8 at 25 °C.

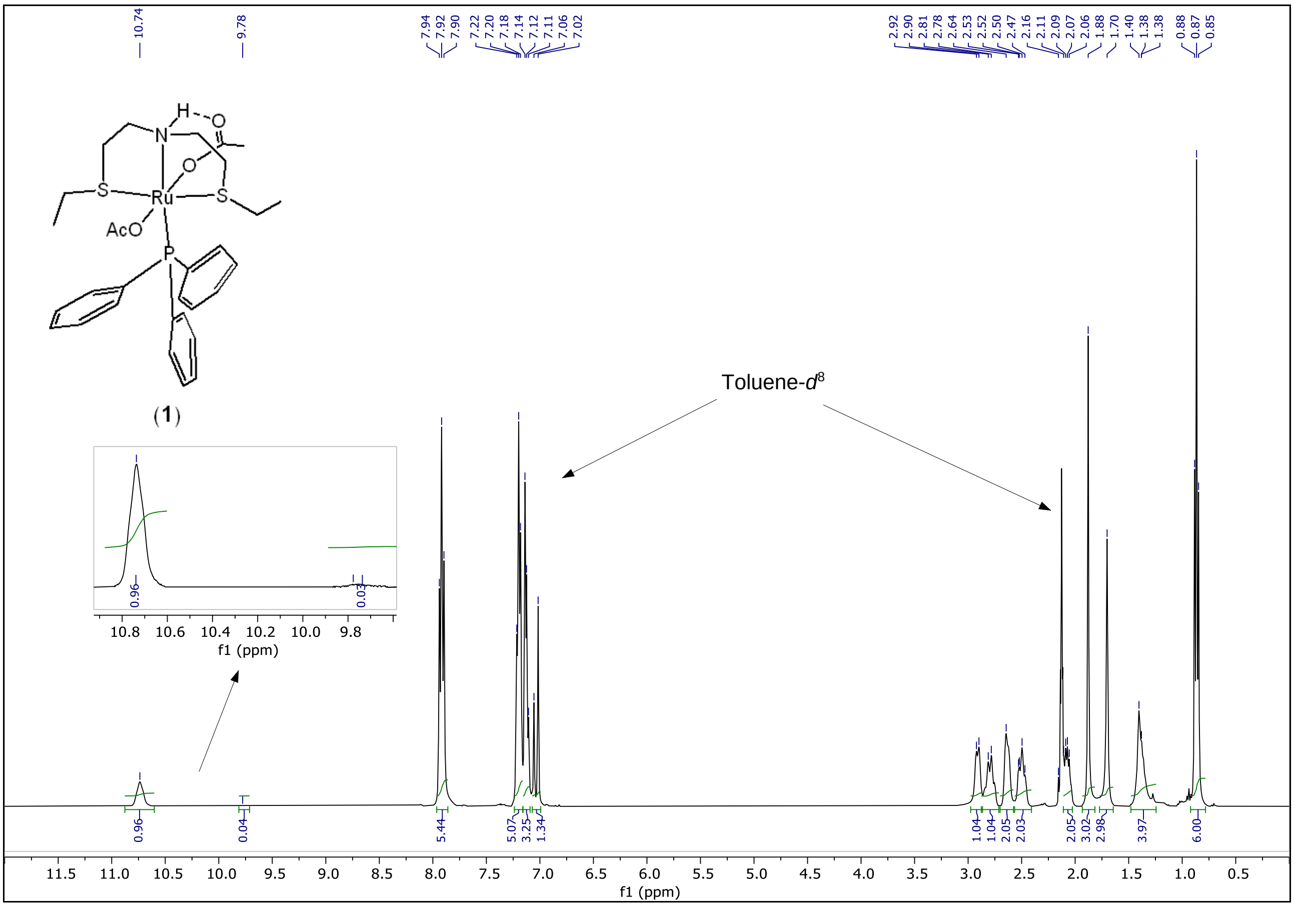

Figure S2. ^{1}H NMR spectrum (400.1 MHz) of *trans*-[Ru(η^1-OAc)$_2$(SNS)(PPh_3)] (**1**) in toluene-d^8 at 25 °C.

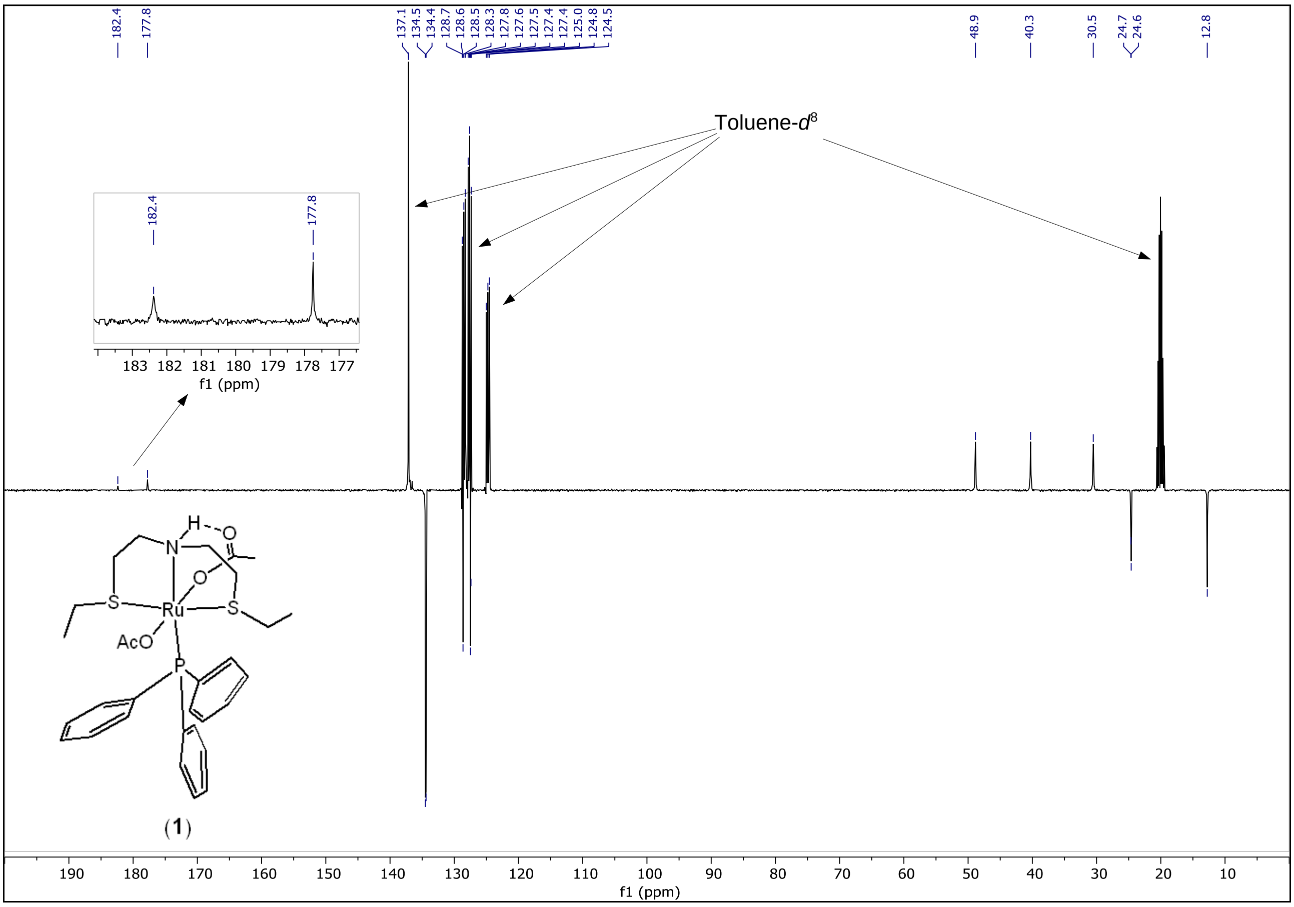

Figure S3. $^{13}C\{^{1}H\}$ DEPTQ NMR spectrum (100.6 MHz) of *trans*-[Ru(η^1-OAc)$_2$(SNS)(PPh$_3$)] (**1**) in toluene-d^8 at 25 °C.

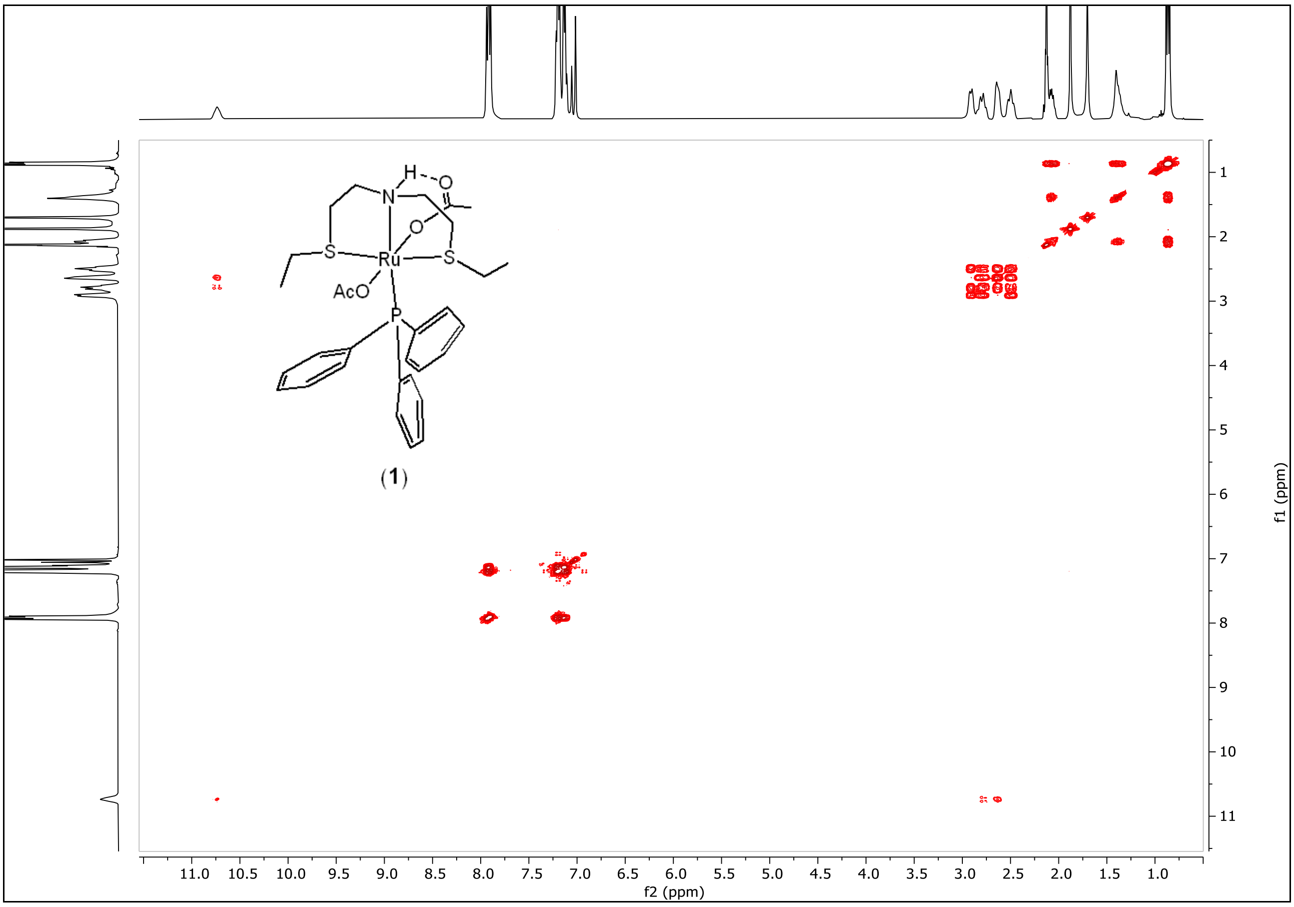

Figure S4. ^{1}H-^{1}H COSY 2D NMR spectrum of *trans*-[Ru(η^{1}-OAc)$_2$(SNS)(PPh$_3$)] (**1**) in toluene-d^{8} at 25 °C.

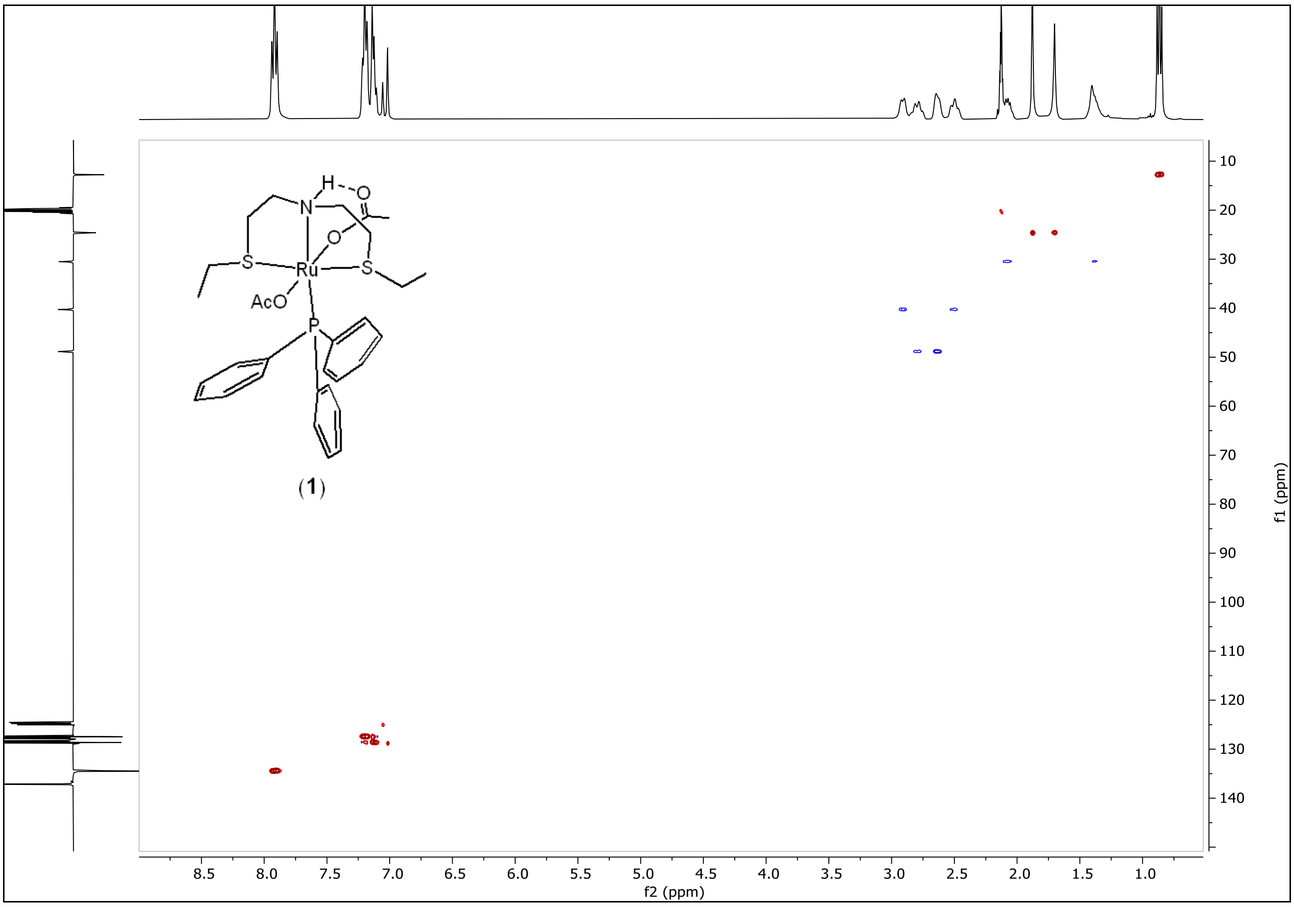

Figure S5. ^{1}H-^{13}C HSQC 2D NMR spectrum of *trans*-[Ru(η^{1}-OAc)$_2$(SNS)(PPh$_3$)] (**1**) in toluene-d^8 at 25 °C.

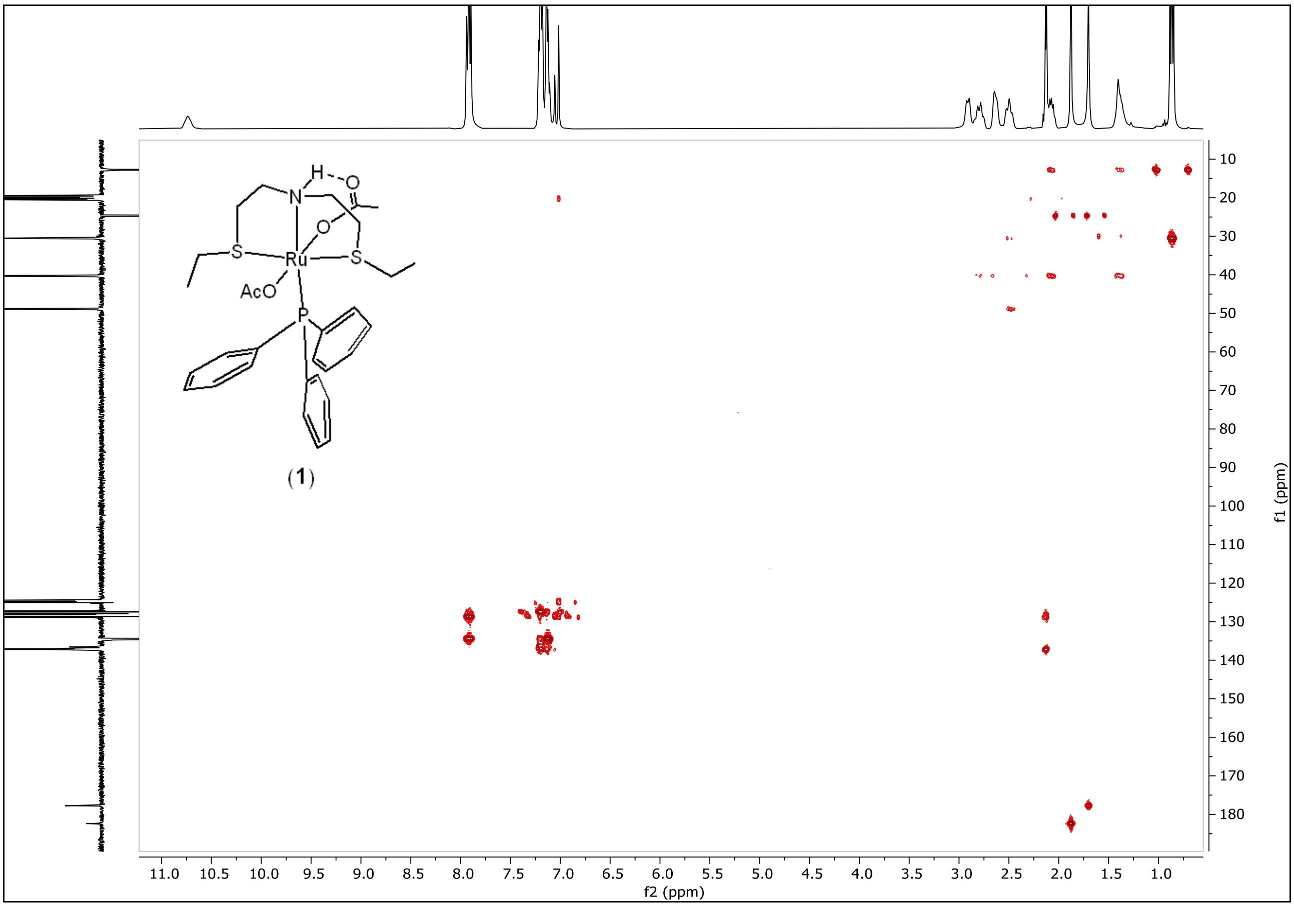

Figure S6. ^{1}H-^{13}C HMBC 2D NMR spectrum of *trans*-[Ru(η^1-OAc)$_2$(SNS)(PPh$_3$)] (**1**) in toluene-d^8 at 25 °C.

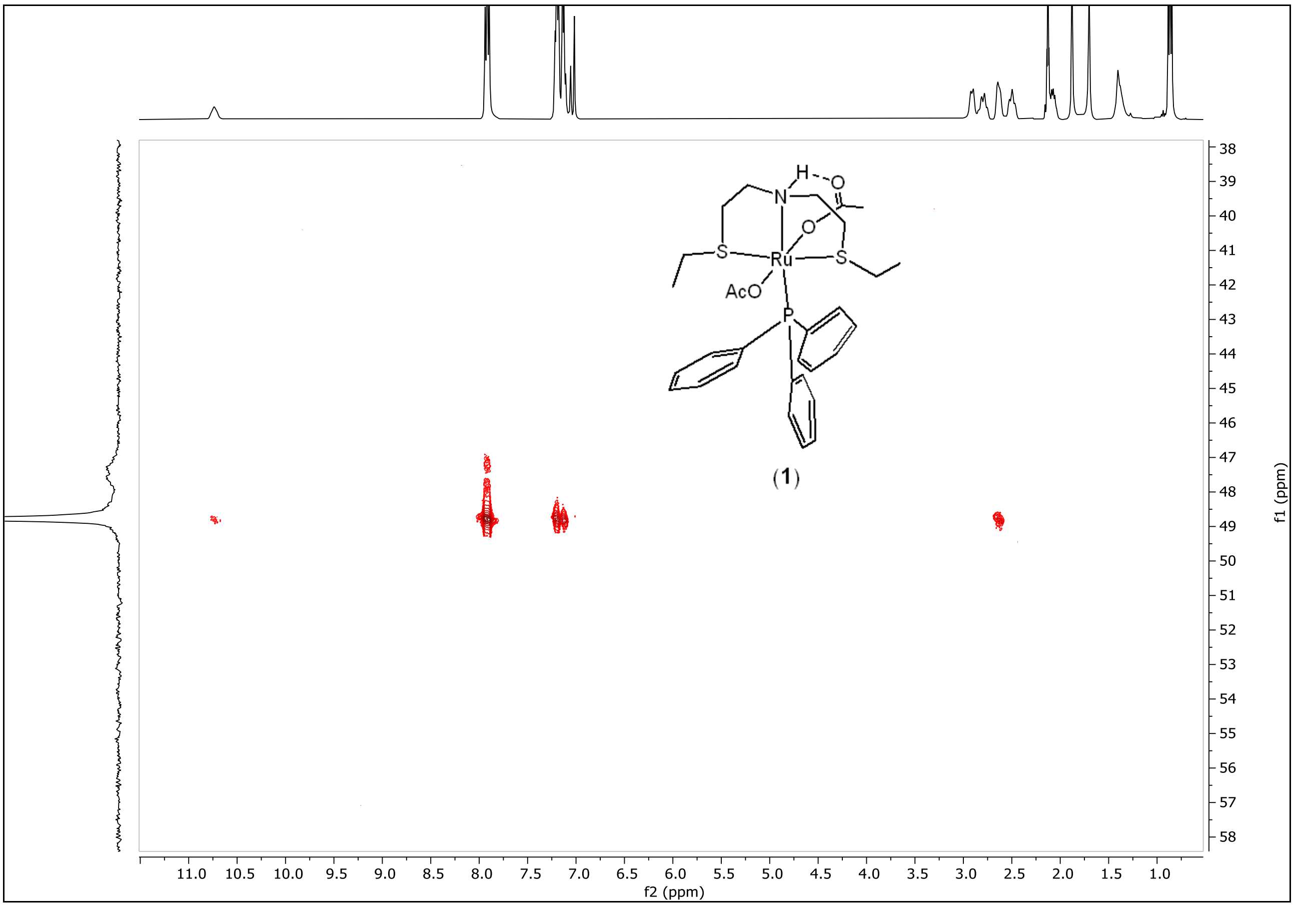

Figure S7. ^{1}H-^{31}P HMBC 2D NMR spectrum of *trans*-[Ru(η^{1}-OAc)$_2$(SNS)(PPh$_3$)] (**1**) in toluene-d^{8} at 25 °C.

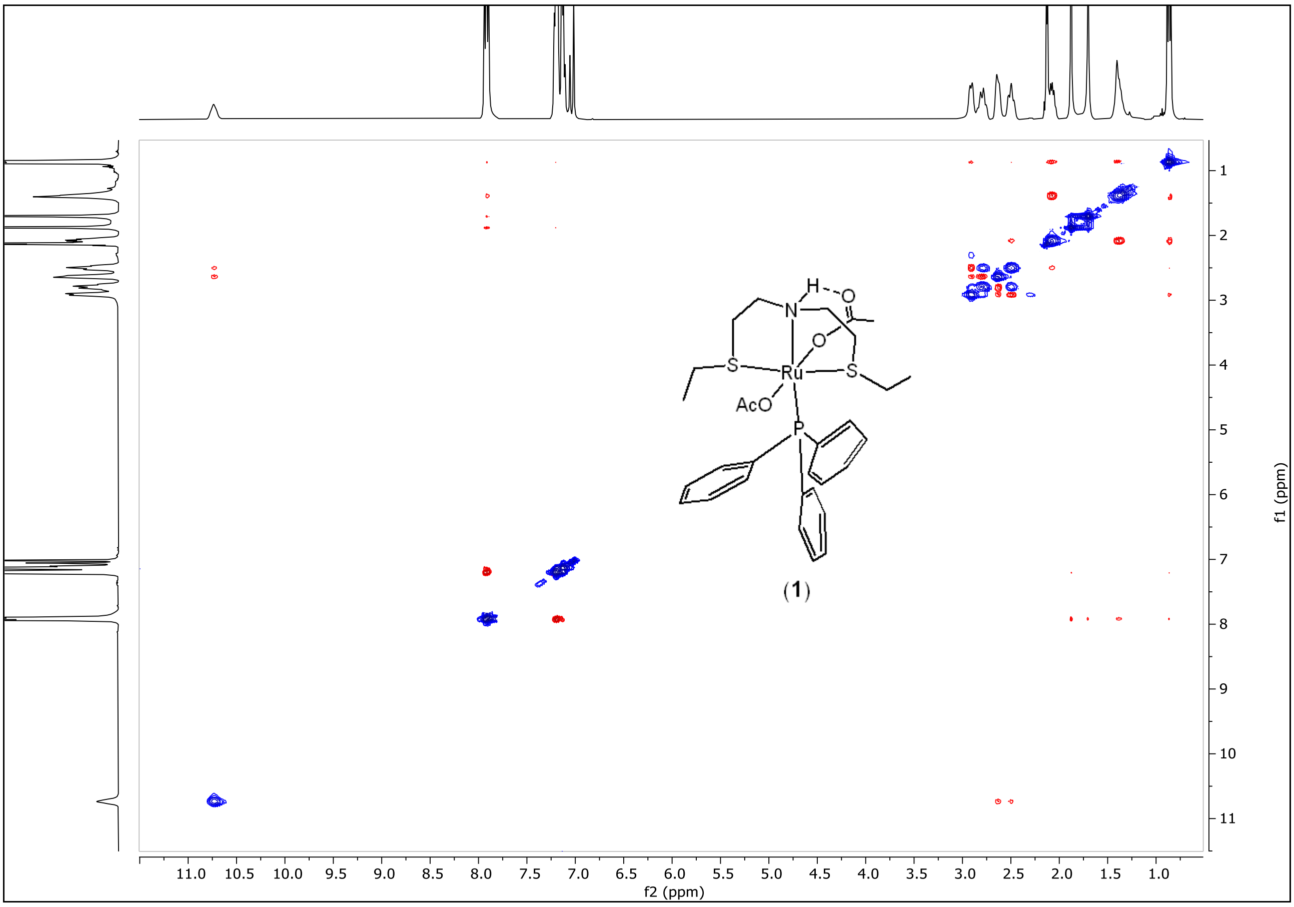

Figure S8. ^{1}H-^{1}H NOESY 2D NMR spectrum of *trans*-[Ru(η^{1}-OAc)$_2$(SNS)(PPh$_3$)] (**1**) in toluene-d^{8} at 25 °C.

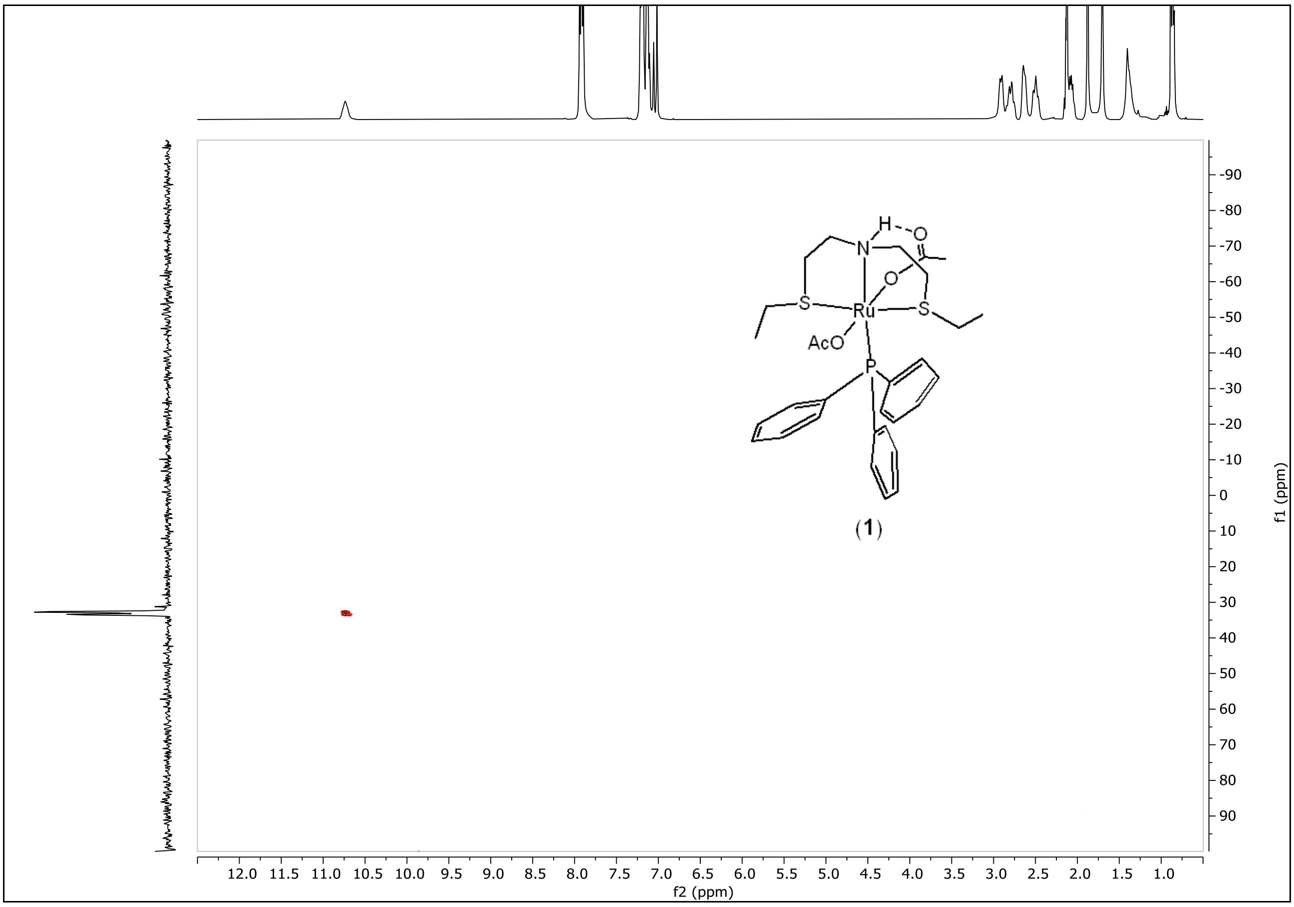

Figure S9. ^{1}H-^{15}N HSQC 2D NMR spectrum of *trans*-[Ru(η^1-OAc)$_2$(SNS)(PPh$_3$)] (**1**) in toluene-d^8 at 25 °C.

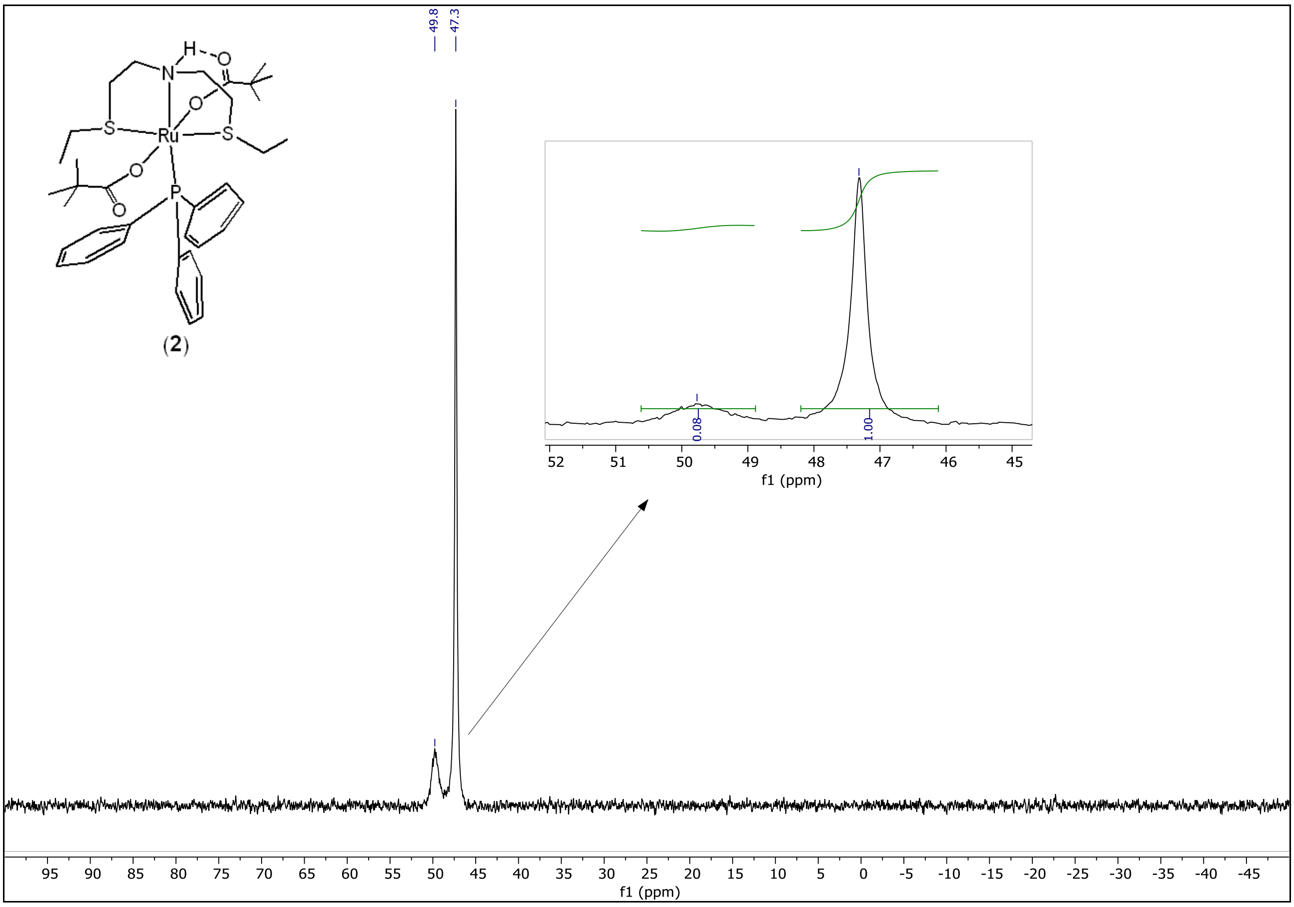

Figure S10. $^{31}P\{^{1}H\}$ NMR spectrum (162.0 MHz) of *trans*-[Ru(η^1-Piv)$_2$(SNS)(PPh$_3$)] (**2**) in toluene-*d*^{8} at 25 °C.

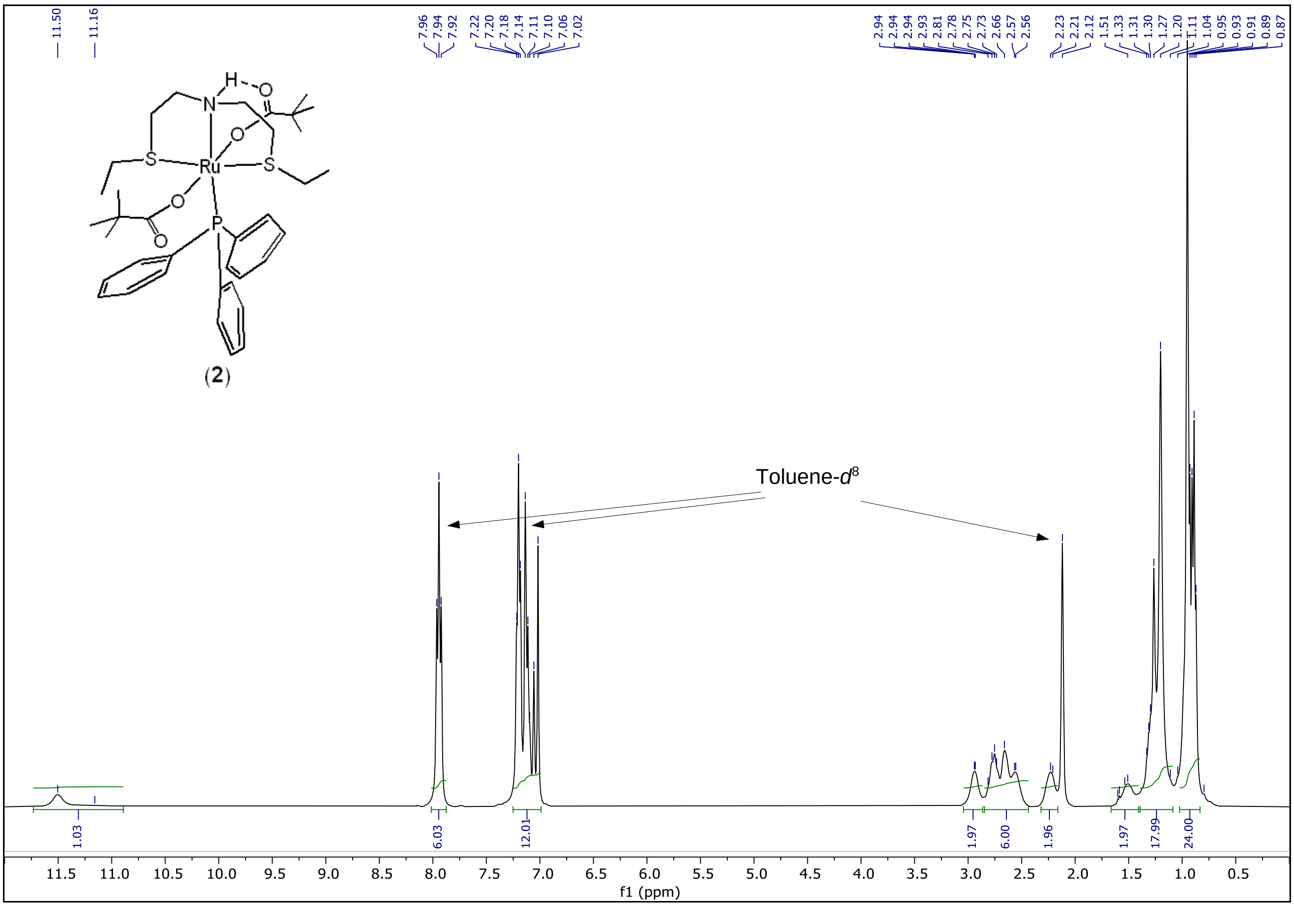

Figure S11. ^{1}H NMR spectrum (400.1 MHz) of *trans*-[Ru(η^{1}-Piv)$_2$(SNS)(PPh$_3$)] (**2**) in toluene-*d*^{8} at 25 °C.

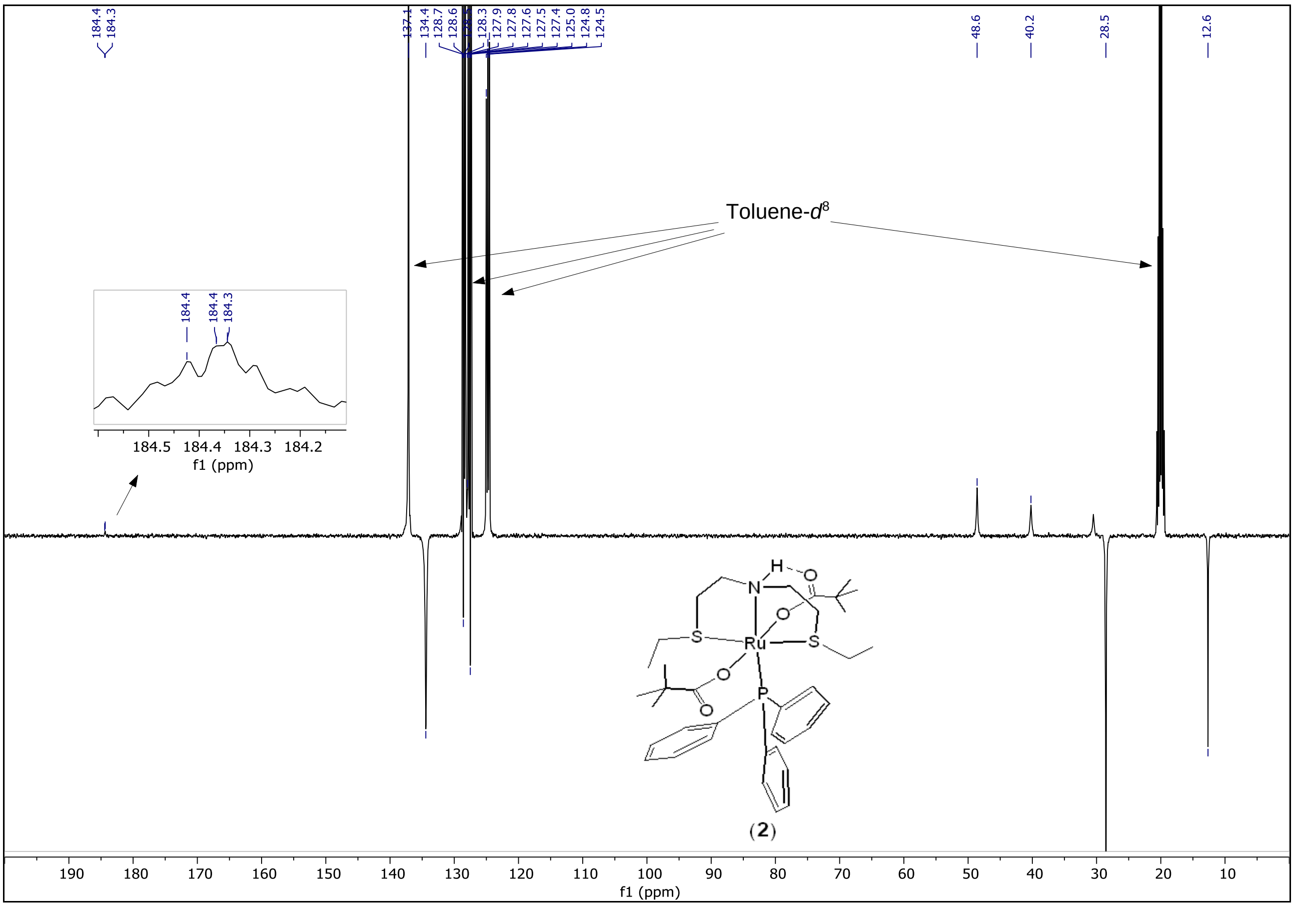

Figure S12. $^{13}C\{^{1}H\}$ NMR spectrum (100.6 MHz) of *trans*-[Ru(η^1-Piv)$_2$(SNS)(PPh$_3$)] (**2**) in toluene-d^8 at 25 °C.

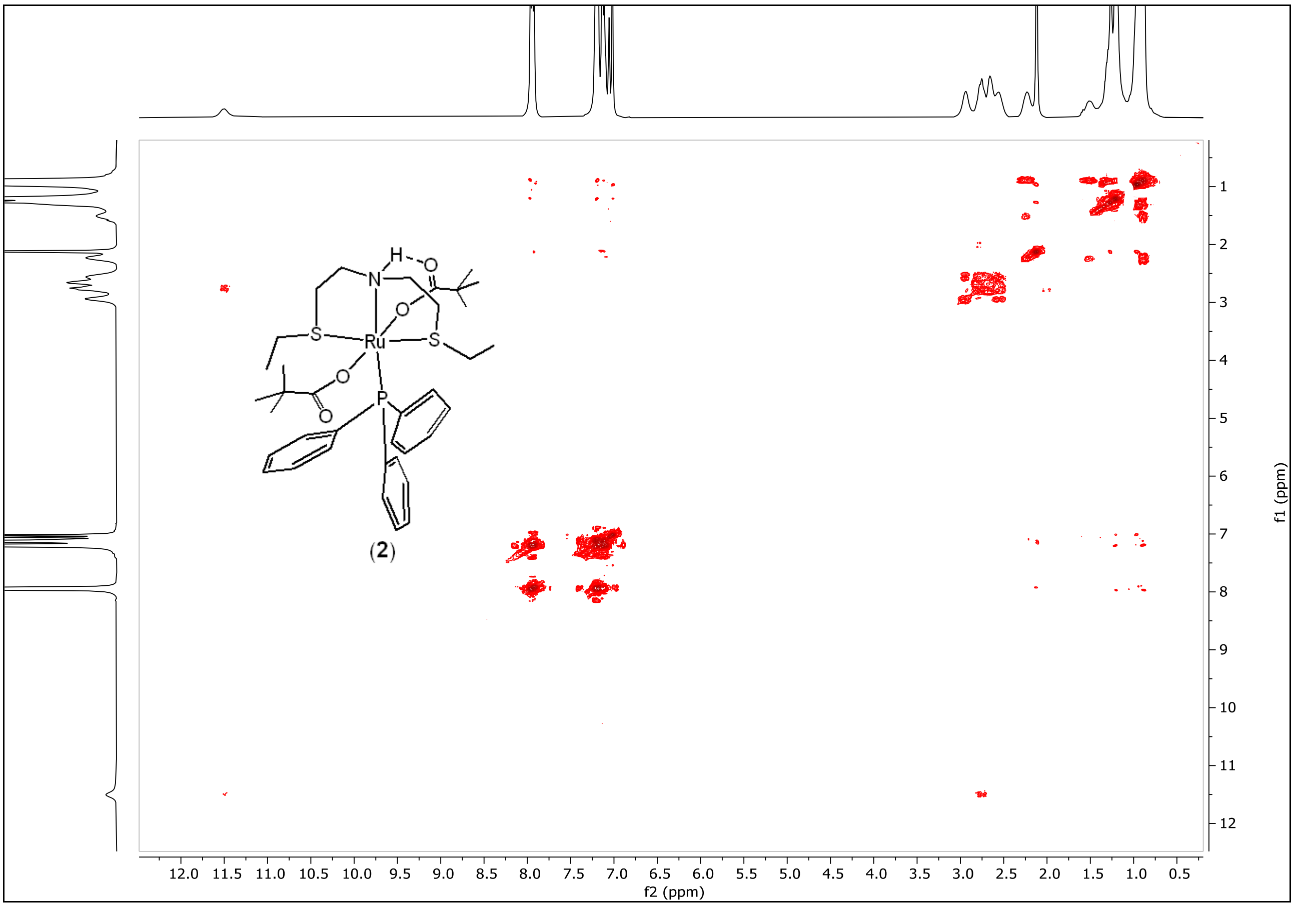

Figure S13. ^{1}H-^{1}H COSY 2D NMR spectrum of *trans*-[Ru(η^1-Piv)$_2$(SNS)(PPh_3)] (**2**) in toluene-d^8 at 25 °C.

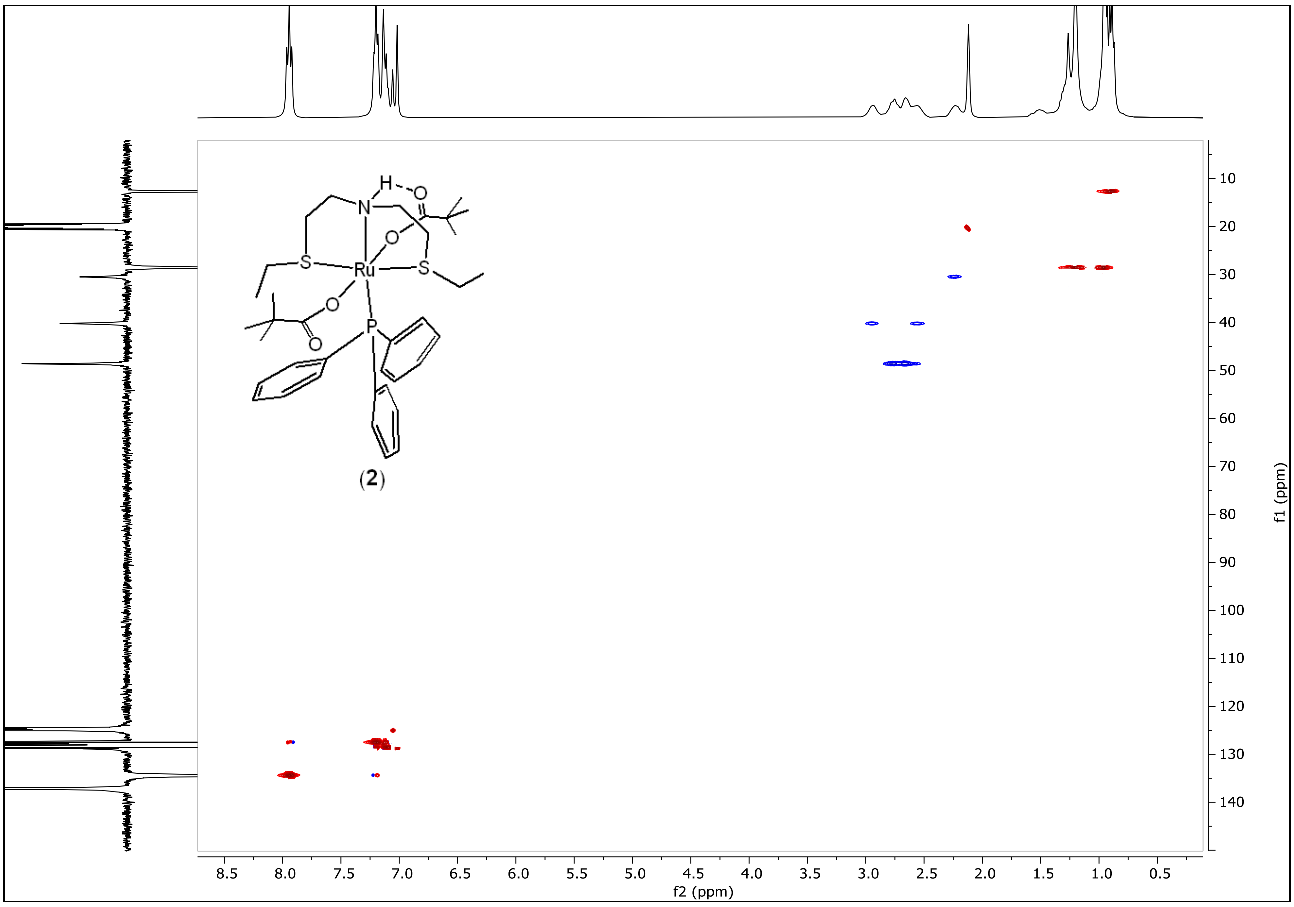

Figure S14. ^{1}H-^{13}C HSQC 2D NMR spectrum *trans*-[Ru(η^1-Piv)$_2$(SNS)(PPh$_3$)] (**2**) in toluene-d^8 at 25 °C.

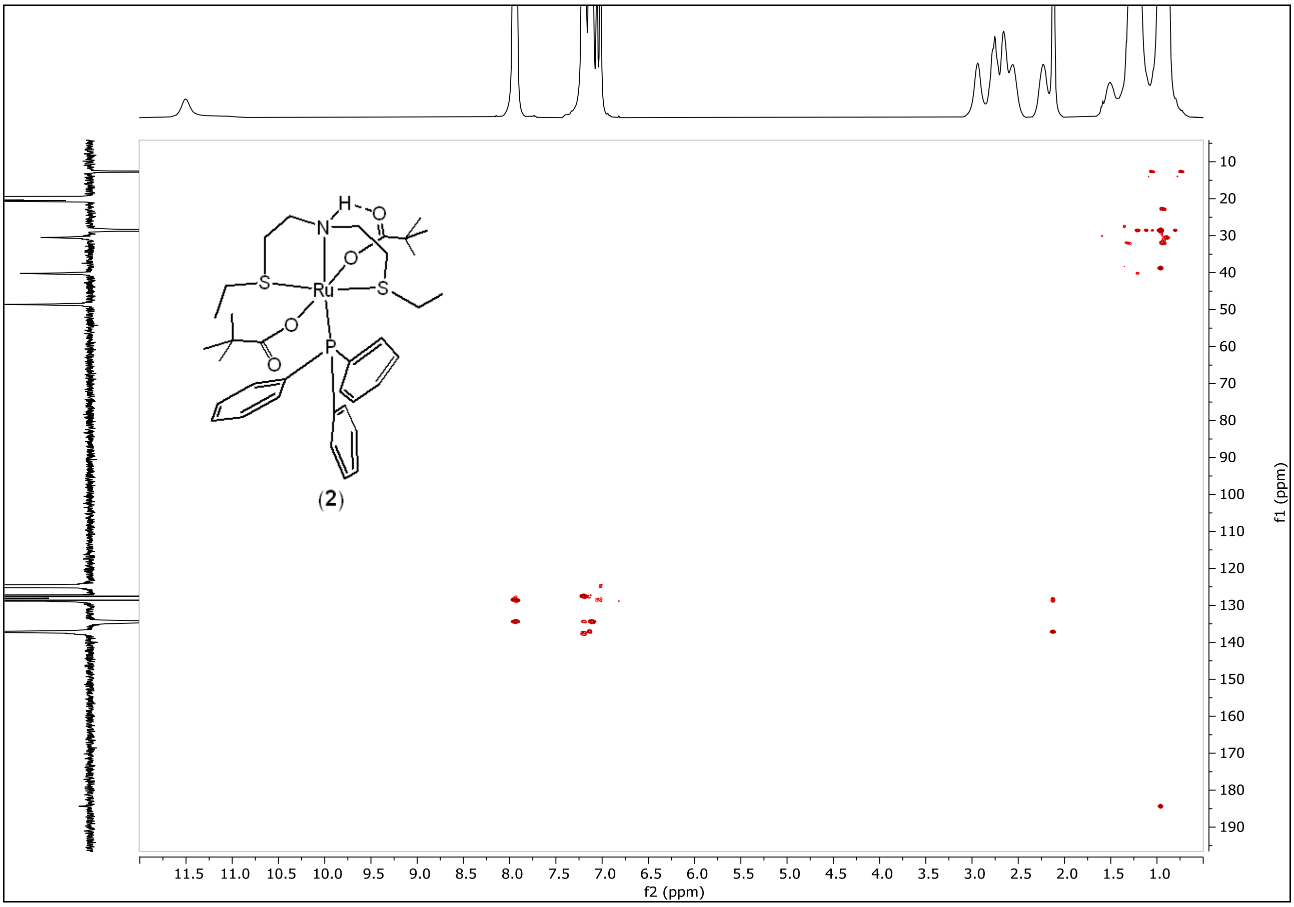

Figure S15. ^{1}H-^{13}C HMBC 2D NMR spectrum of *trans*-[Ru(η^1-Piv)$_2$(SNS)(PPh$_3$)] (**2**) in toluene-d^8 at 25 °C.

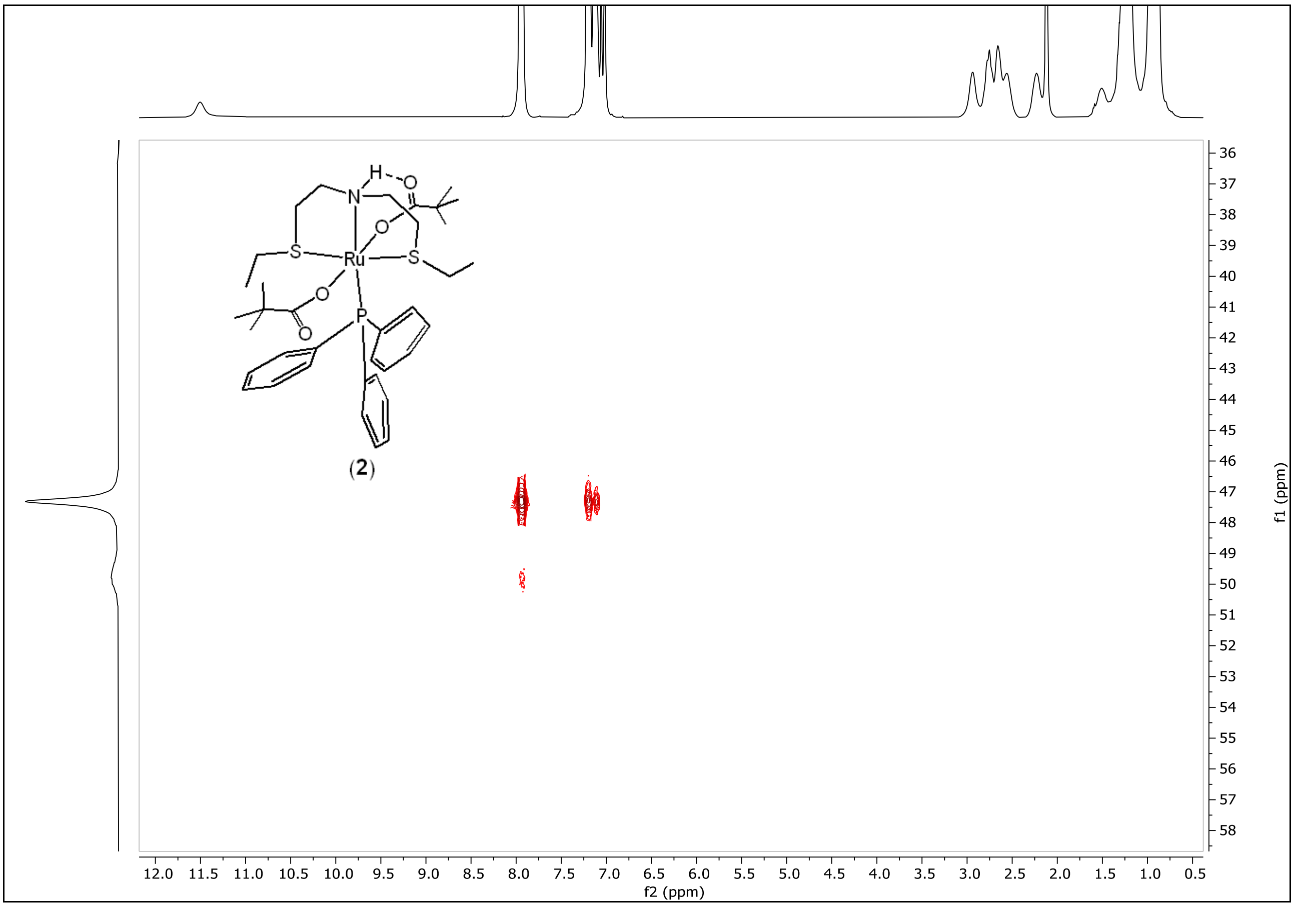

Figure S16. ^{1}H-^{31}P HMBC 2D NMR spectrum of *trans*-[Ru(η^{1}-Piv)$_2$(SNS)(PPh$_3$)] (**2**) in toluene-d^8 at 25 °C

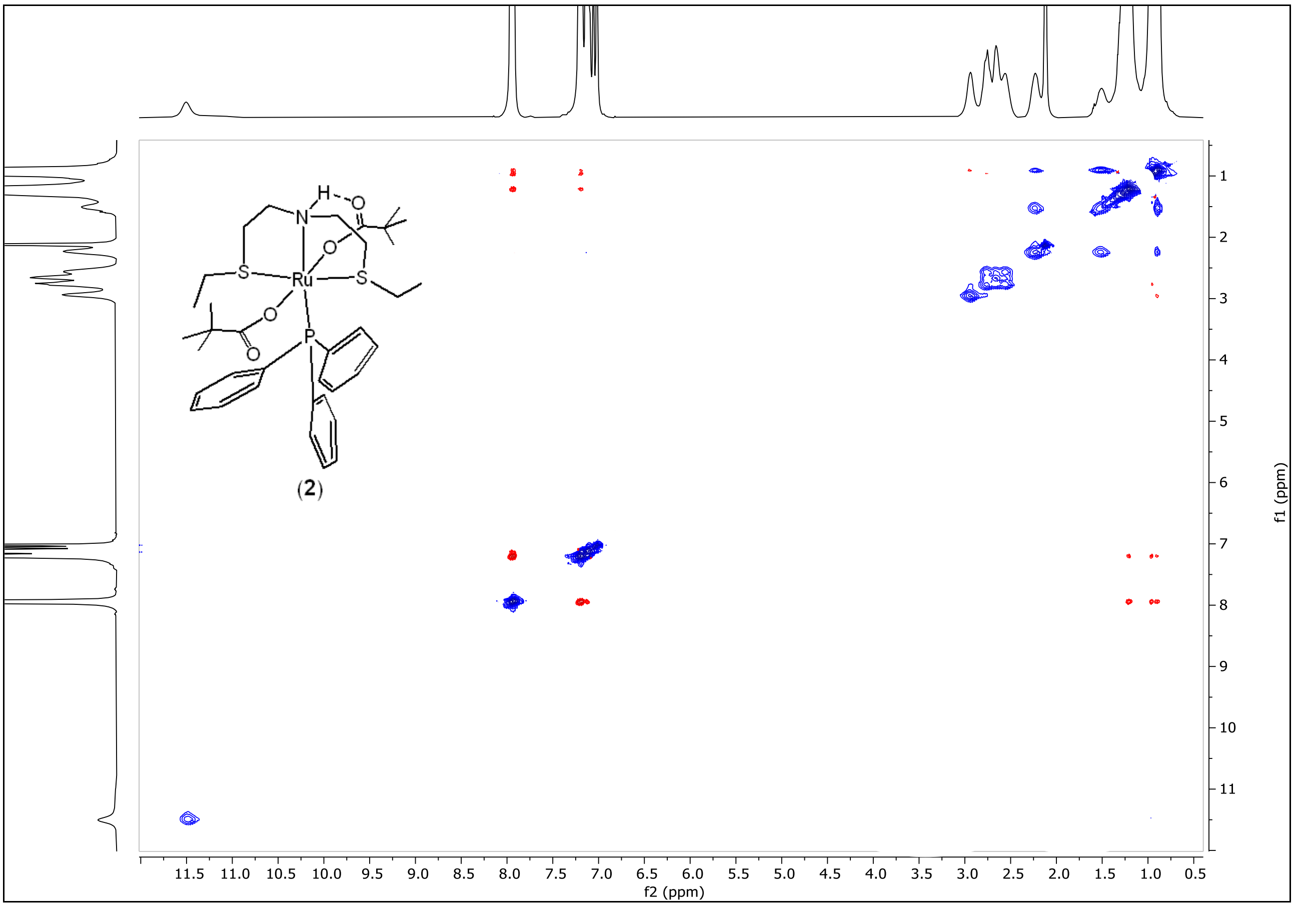

Figure S17. ^{1}H-^{1}H NOESY 2D NMR spectrum of *trans*-[Ru(η^1-Piv)$_2$(SNS)(PPh$_3$)] (**2**) in toluene-d^8 at 25 °C.

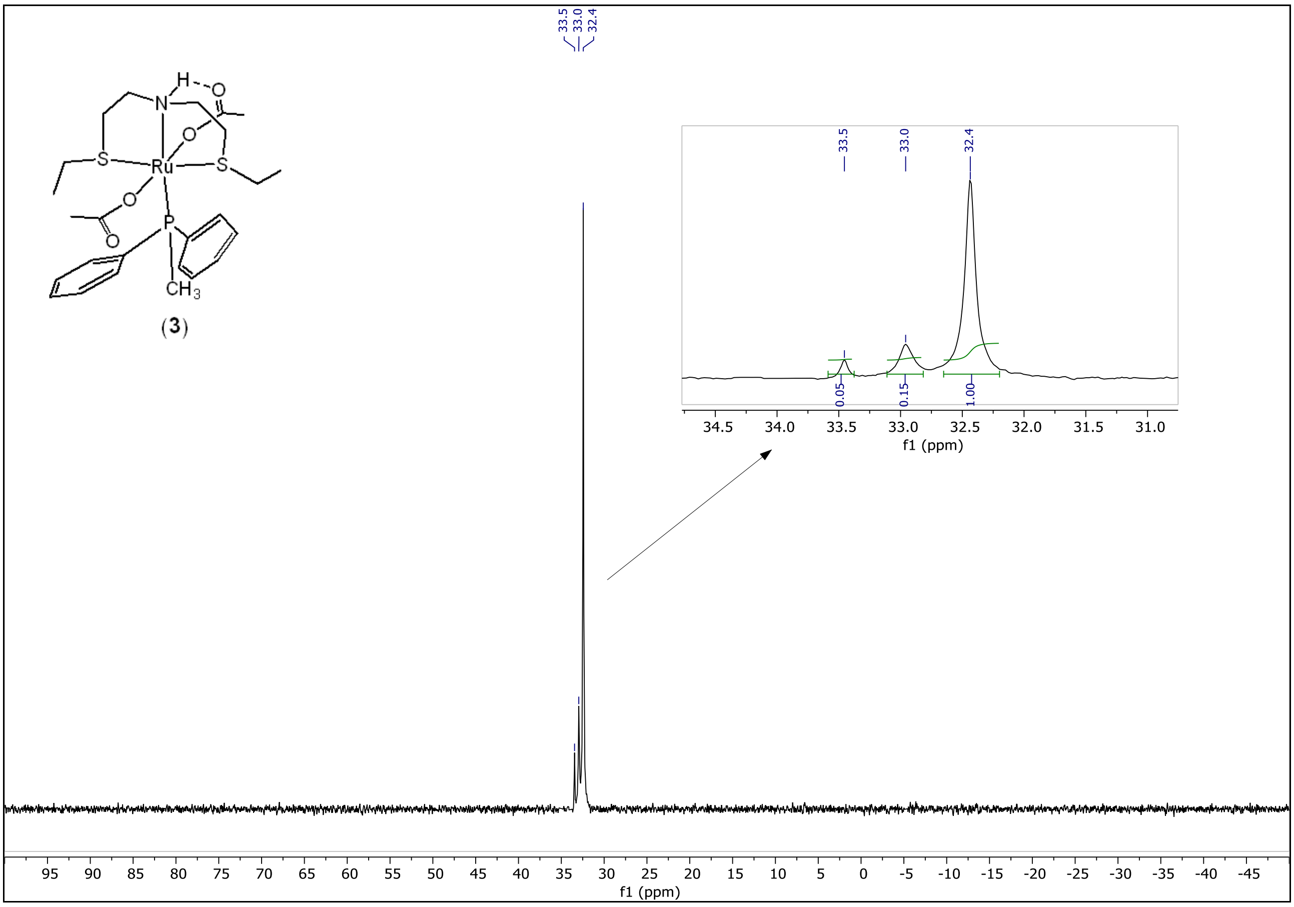

Figure S18. $^{31}P\{^{1}H\}$ NMR spectrum (162.0 MHz) of *trans*-[Ru(η^1-OAc)$_2$(SNS)(PPh$_2$Me)] (**3**) in toluene-d^8 at 25 °C.

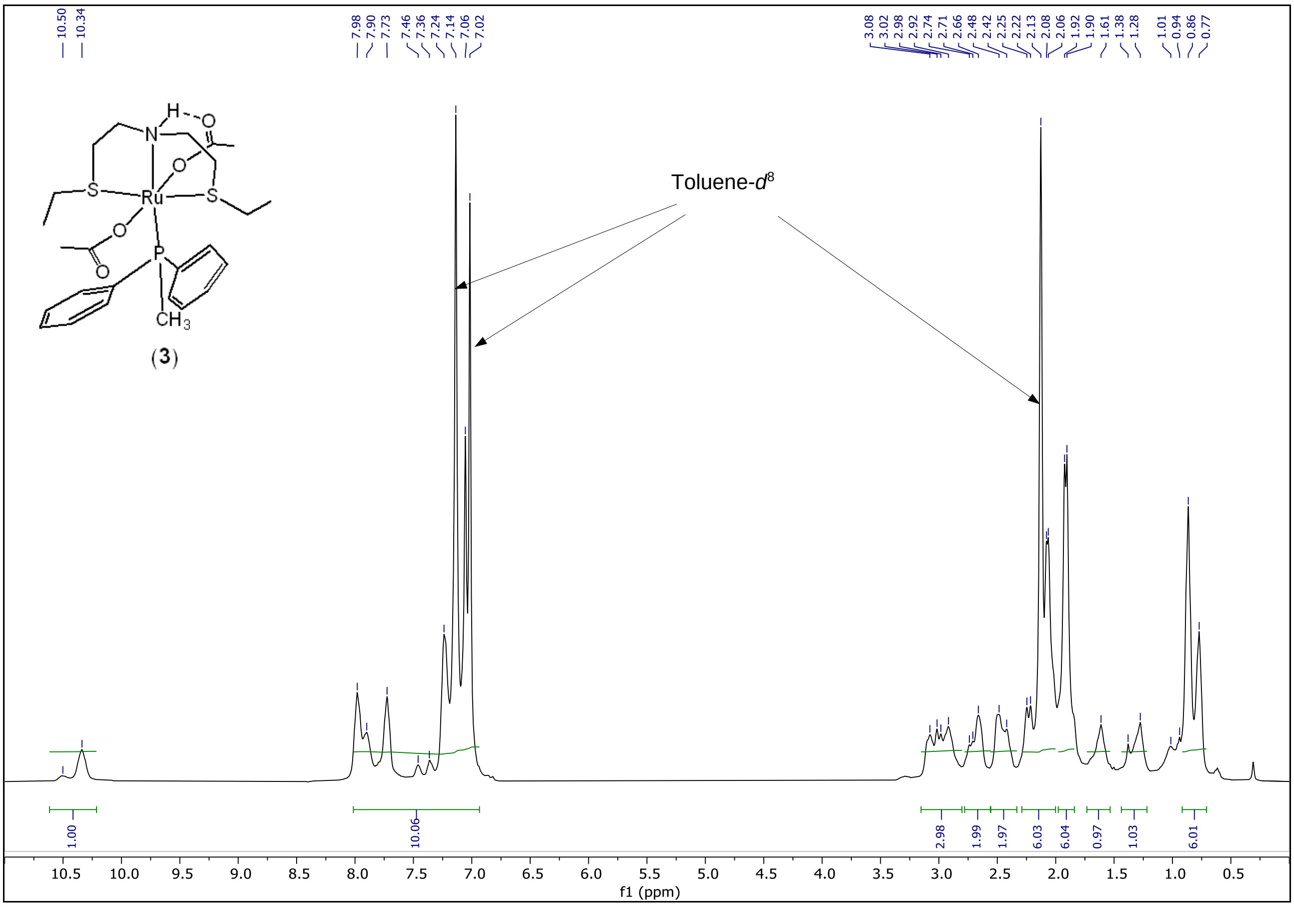

Figure S19. ^{1}H NMR spectrum (400.1 MHz) of [*trans*-[Ru(η^1-OAc)$_2$(SNS)(PPh$_2$Me)] (**3**) in toluene-d^8 at 25 °C.

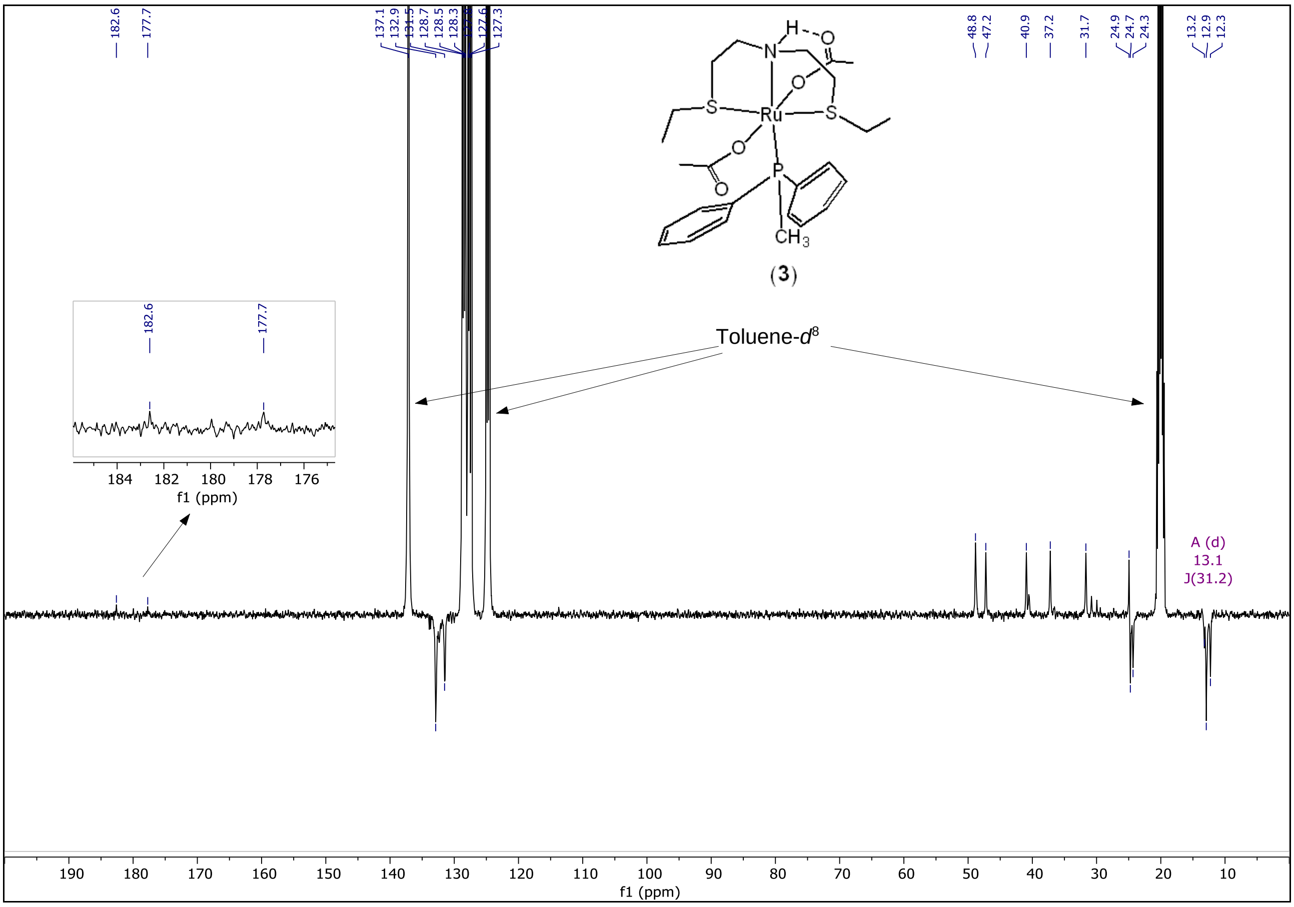

Figure S20. $^{13}C\{^{1}H\}$ DEPTQ NMR spectrum (100.6 MHz) of *trans*-[Ru(η^1-OAc)$_2$(SNS)(PPh$_2$Me)] (**3**) in toluene-d^8 at 25 °C.

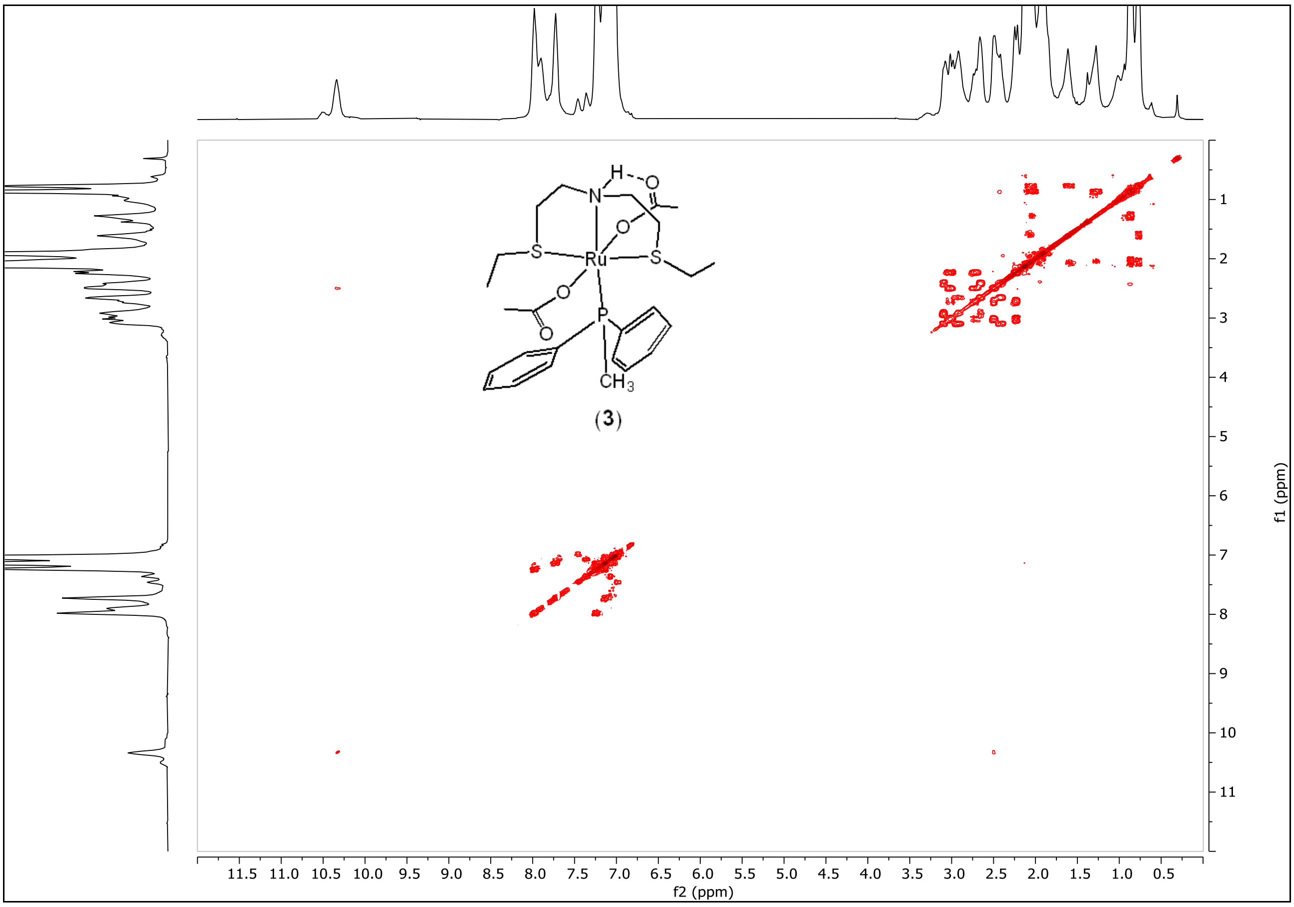

Figure S21. ^{1}H-^{1}H COSY 2D NMR spectrum of *trans*-[Ru(η^1-OAc)$_2$(SNS)(PPh$_2$Me)] (**3**) in toluene-*d*^{8} at 25 °C.

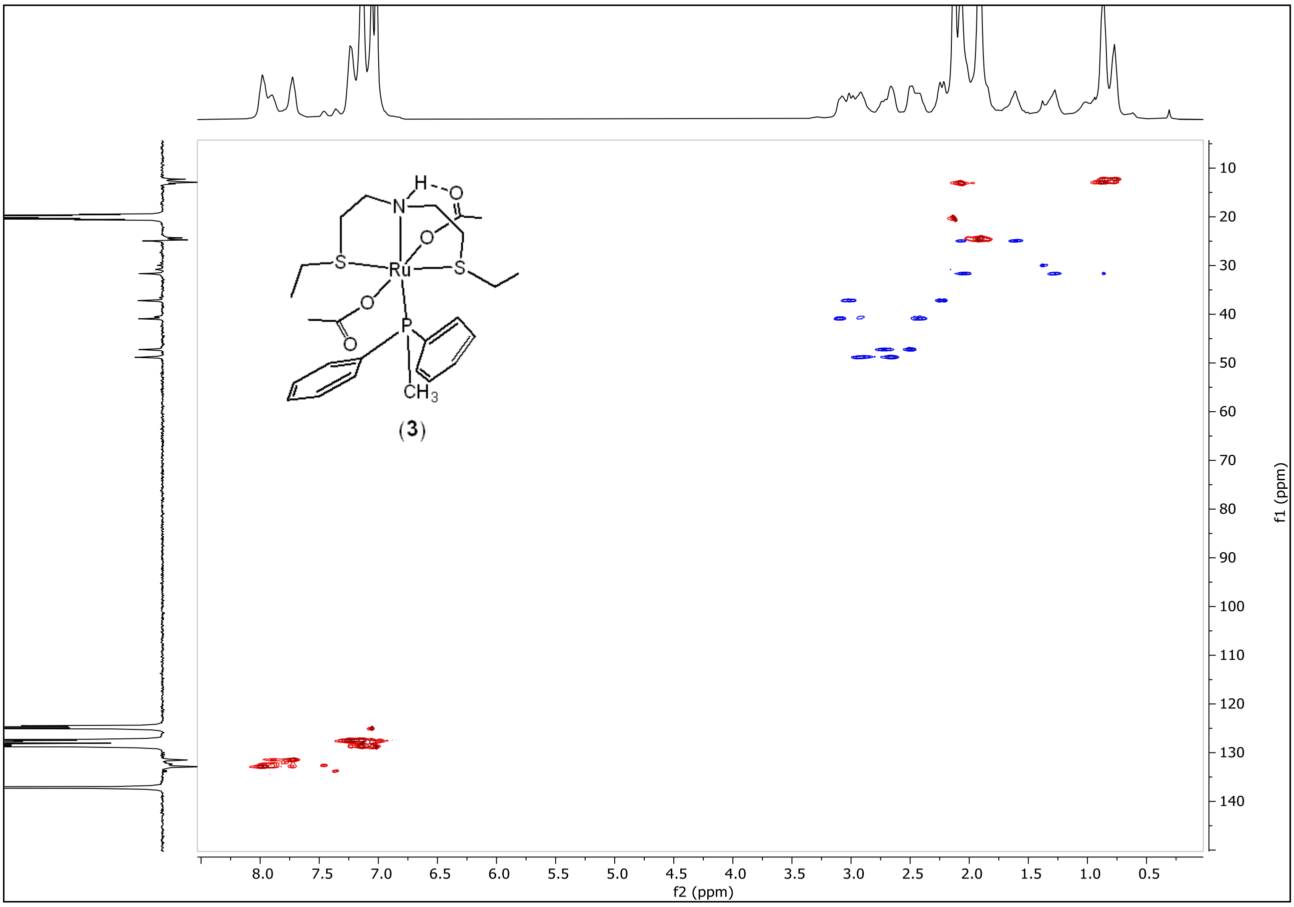

Figure S22. ^{1}H-^{13}C HSQC 2D NMR spectrum of *trans*-[Ru(η^{1}-OAc)$_2$(SNS)(PPh$_2$Me)] (**3**) in toluene-*d*^{8} at 25 °C.

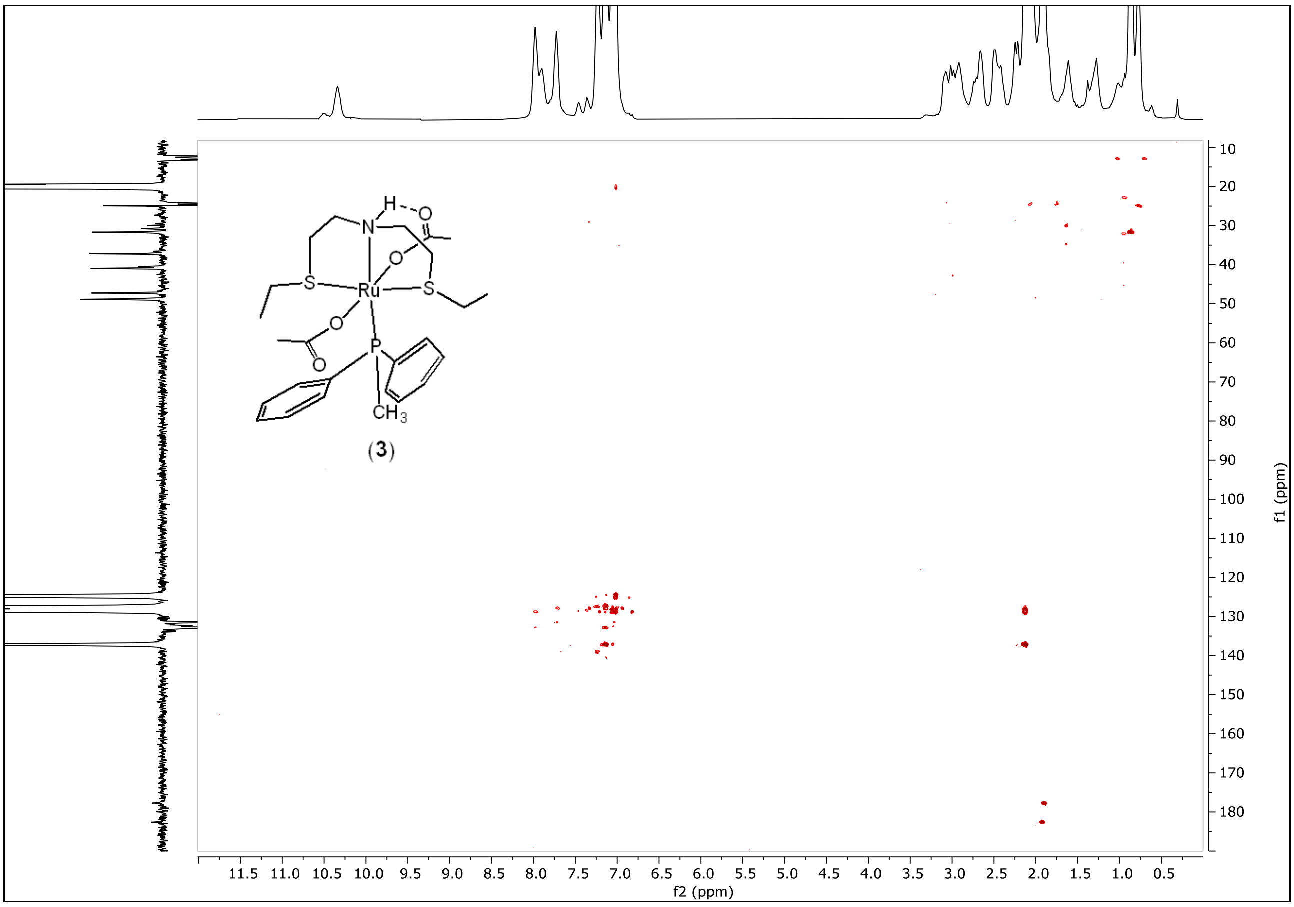

Figure S23. ^{1}H-^{13}C HMBC 2D NMR spectrum of *trans*-[Ru(η^{1}-OAc)$_2$(SNS)(PPh$_2$Me)] (**3**) in toluene-d^{8} at 25 °C.

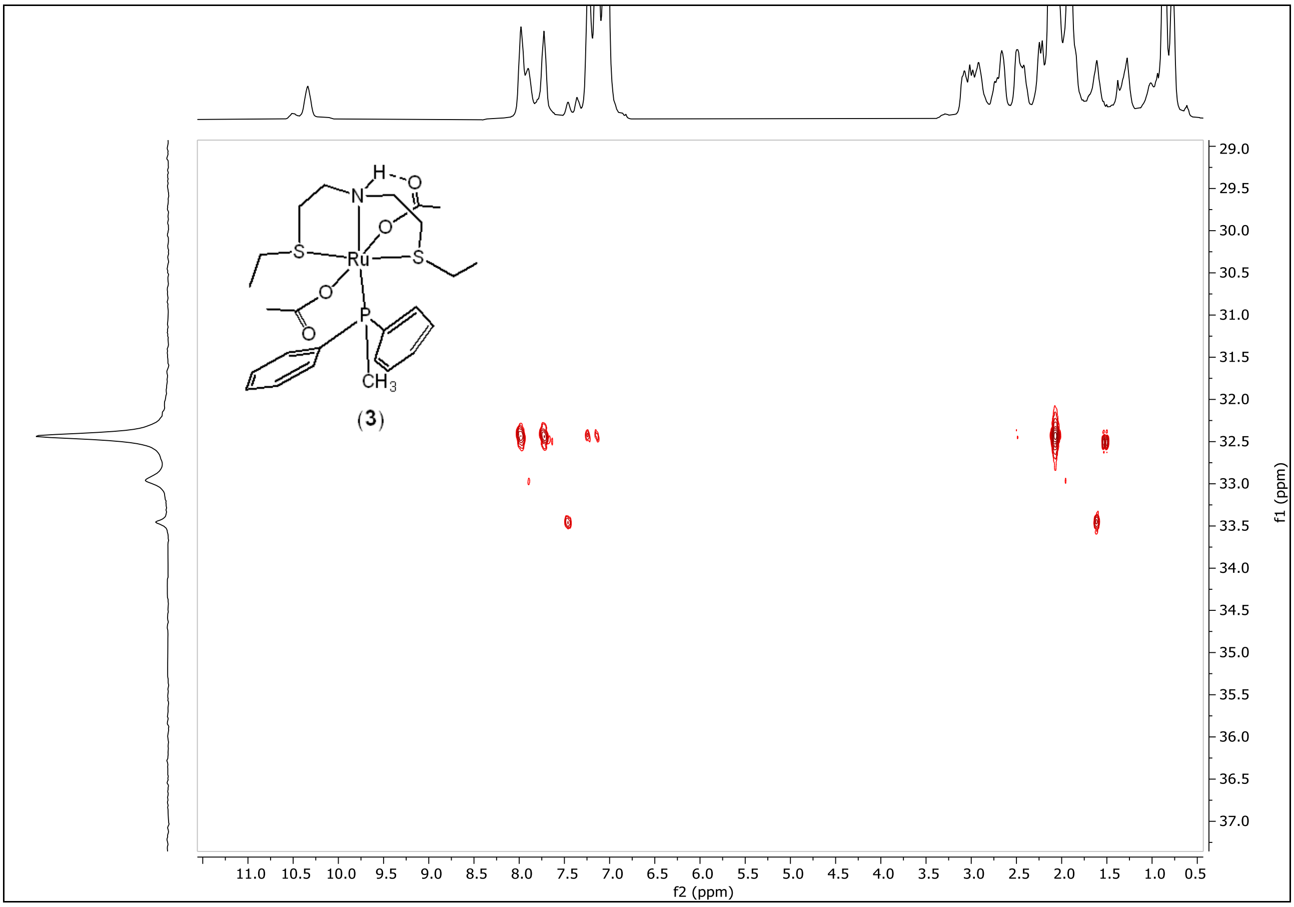

Figure S24. ^{1}H-^{31}P HMBC 2D NMR spectrum of *trans*-[Ru(η^1-OAc)$_2$(SNS)(PPh_2Me)] (**3**) in toluene-d^8 at 25 °C.

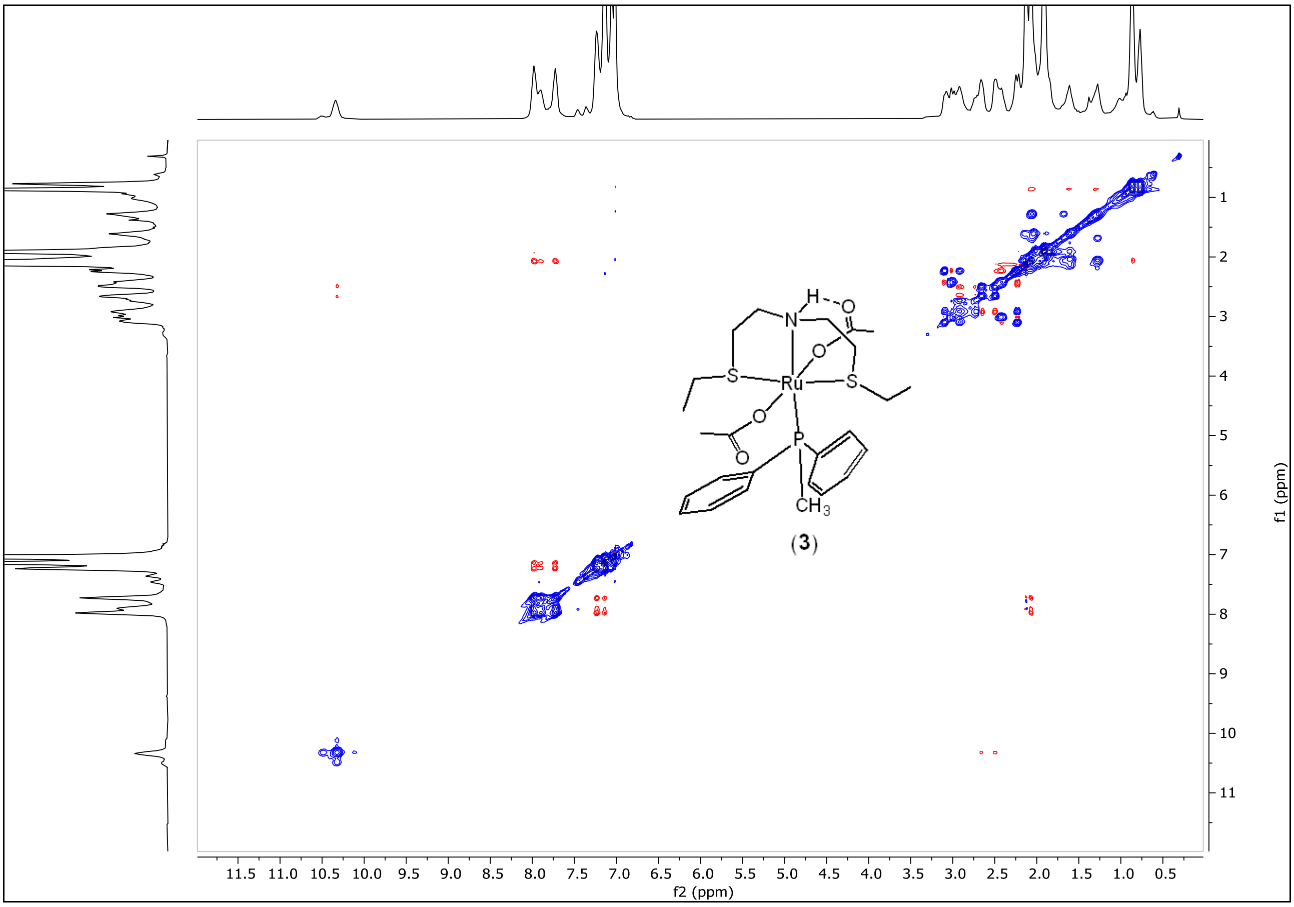

Figure S25. ^{1}H-^{1}H NOESY 2D NMR spectrum of *trans*-[Ru(η^1-OAc)$_2$(SNS)(PPh$_2$Me)] (**3**) in toluene-d^8 at 25 °C.

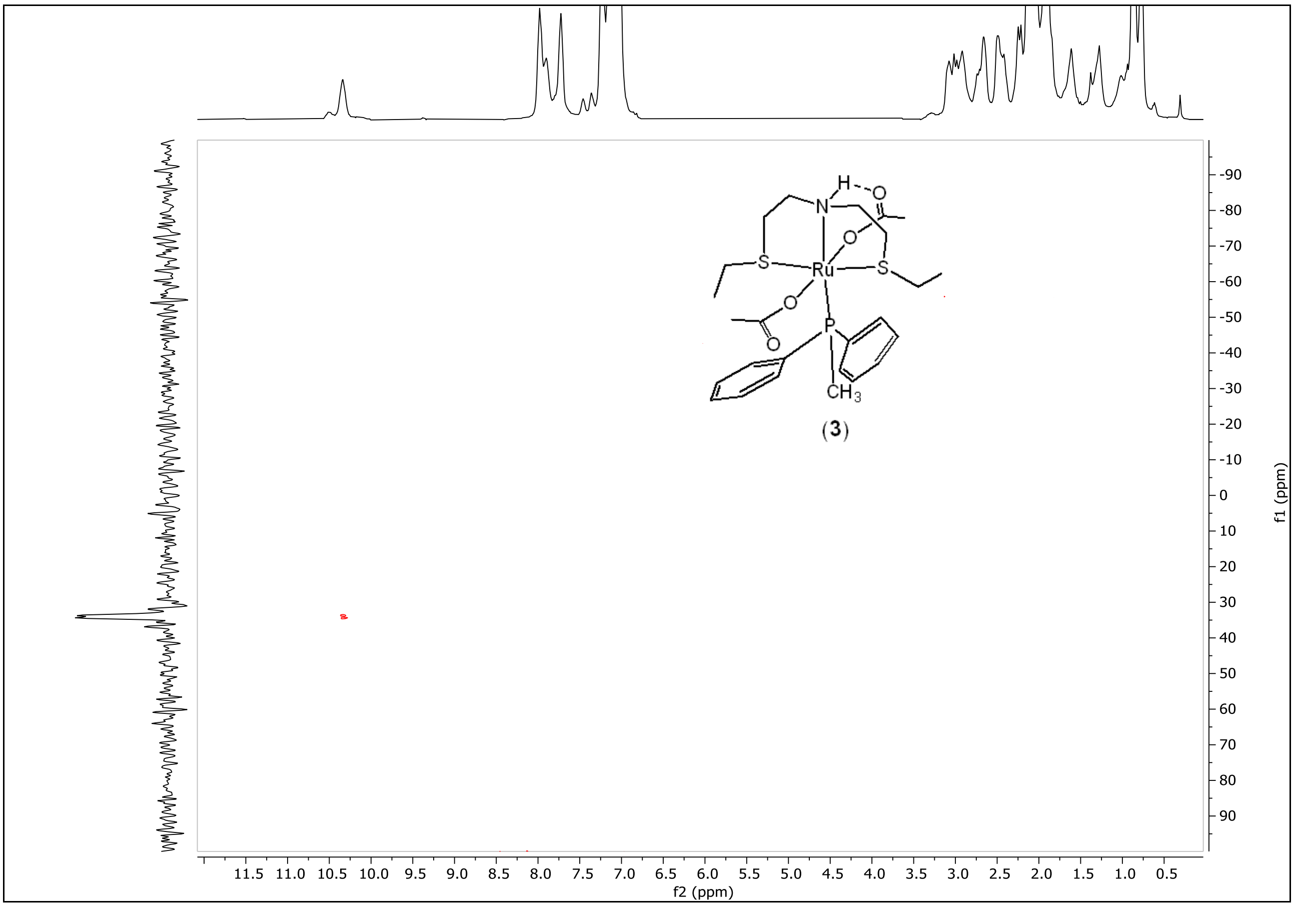

Figure S26. ^{1}H-^{15}N HSQC 2D NMR spectrum of *trans*-[Ru(η^{1}-OAc)$_2$(SNS)(PPh$_2$Me)] (**3**) in toluene-d^8 at 25 °C.

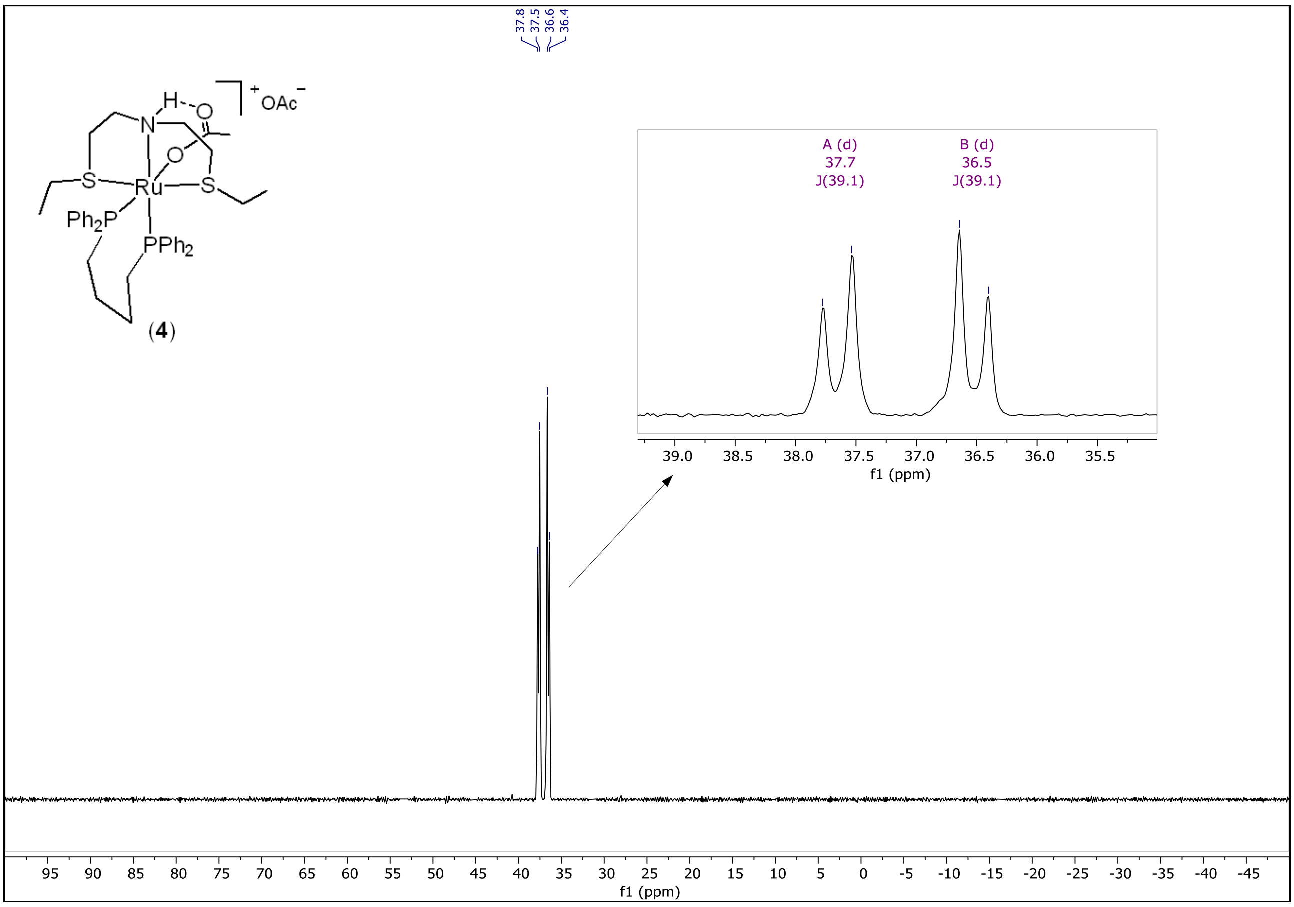

Figure S27. $^{31}P\{^{1}H\}$ NMR spectrum (162.0 MHz) of *cis*-[Ru(η^{1}-OAc)(SNS)(dppb)] (**4**) in toluene-*d*^{8} at 25 °C.

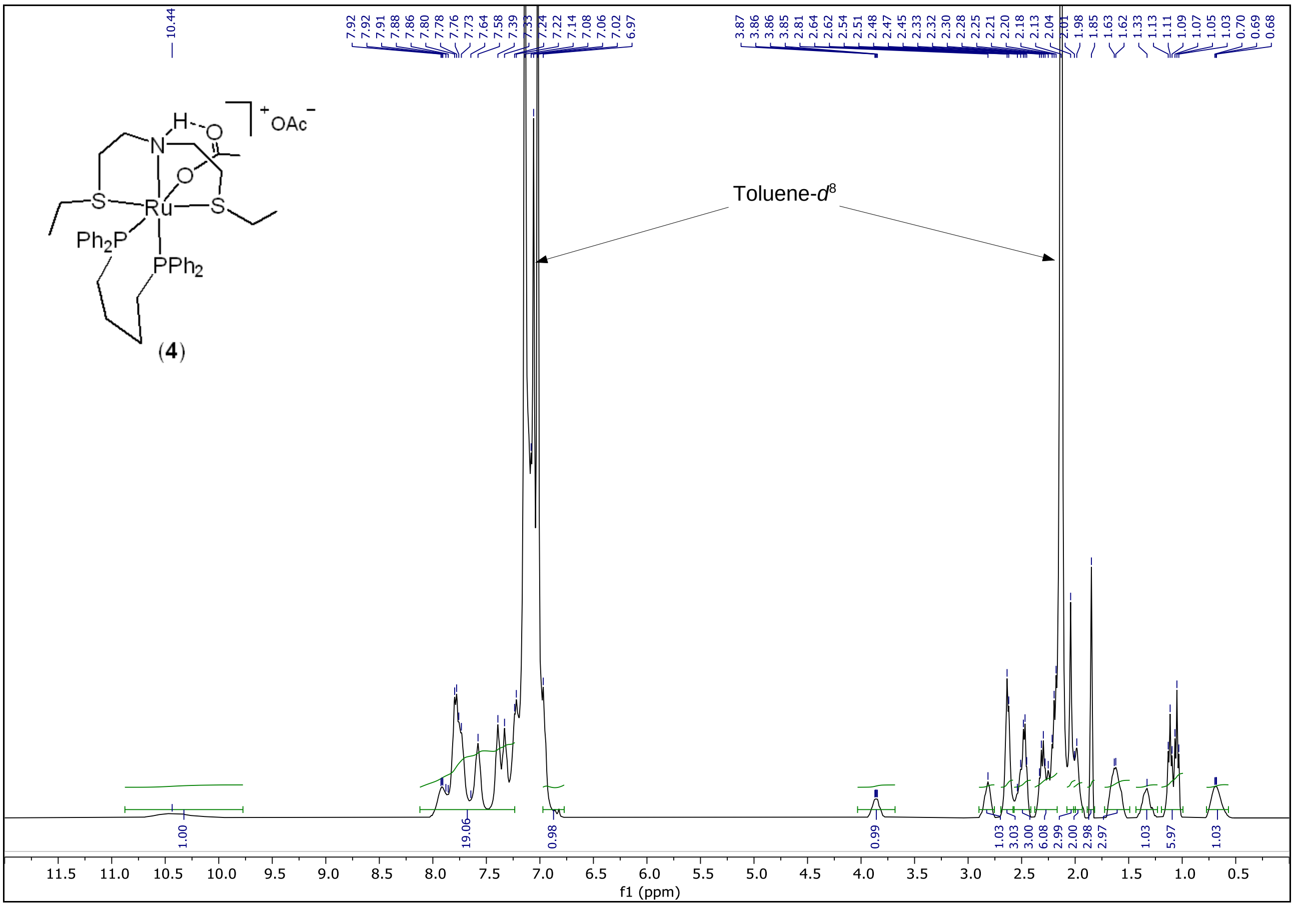

Figure S28. ^{1}H NMR spectrum (400.1 MHz) of *cis*-[Ru(η^1-OAc)(SNS)(dppb)] (**4**) in toluene-d^8 at 25 °C.

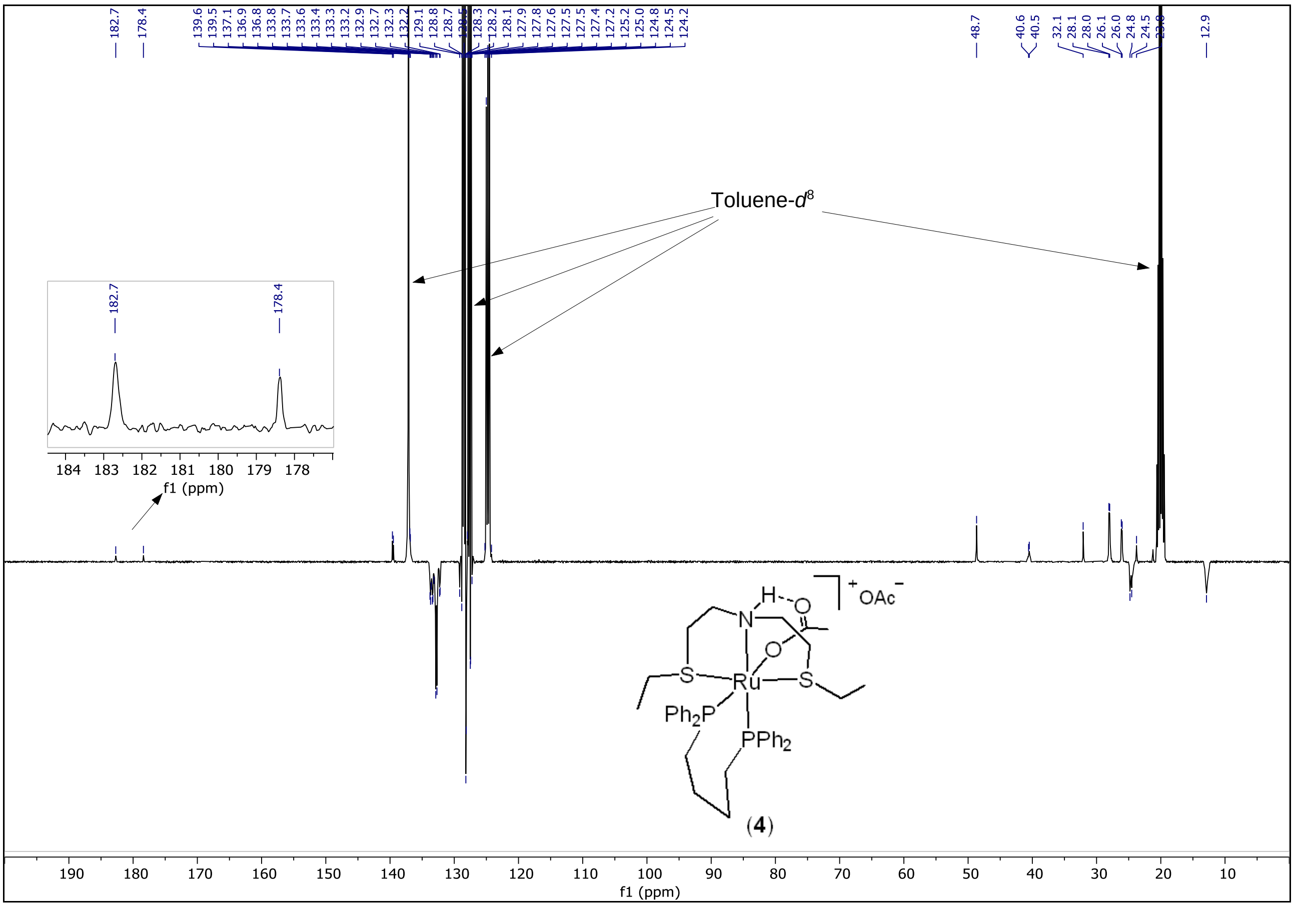

Figure S29. $^{13}C\{^{1}H\}$ DEPTQ NMR spectrum (100.6 MHz) of *cis*-[Ru(η[1]-OAc)(SNS)(dppb)] (**4**) in toluene-*d*[8] at 25 °C.

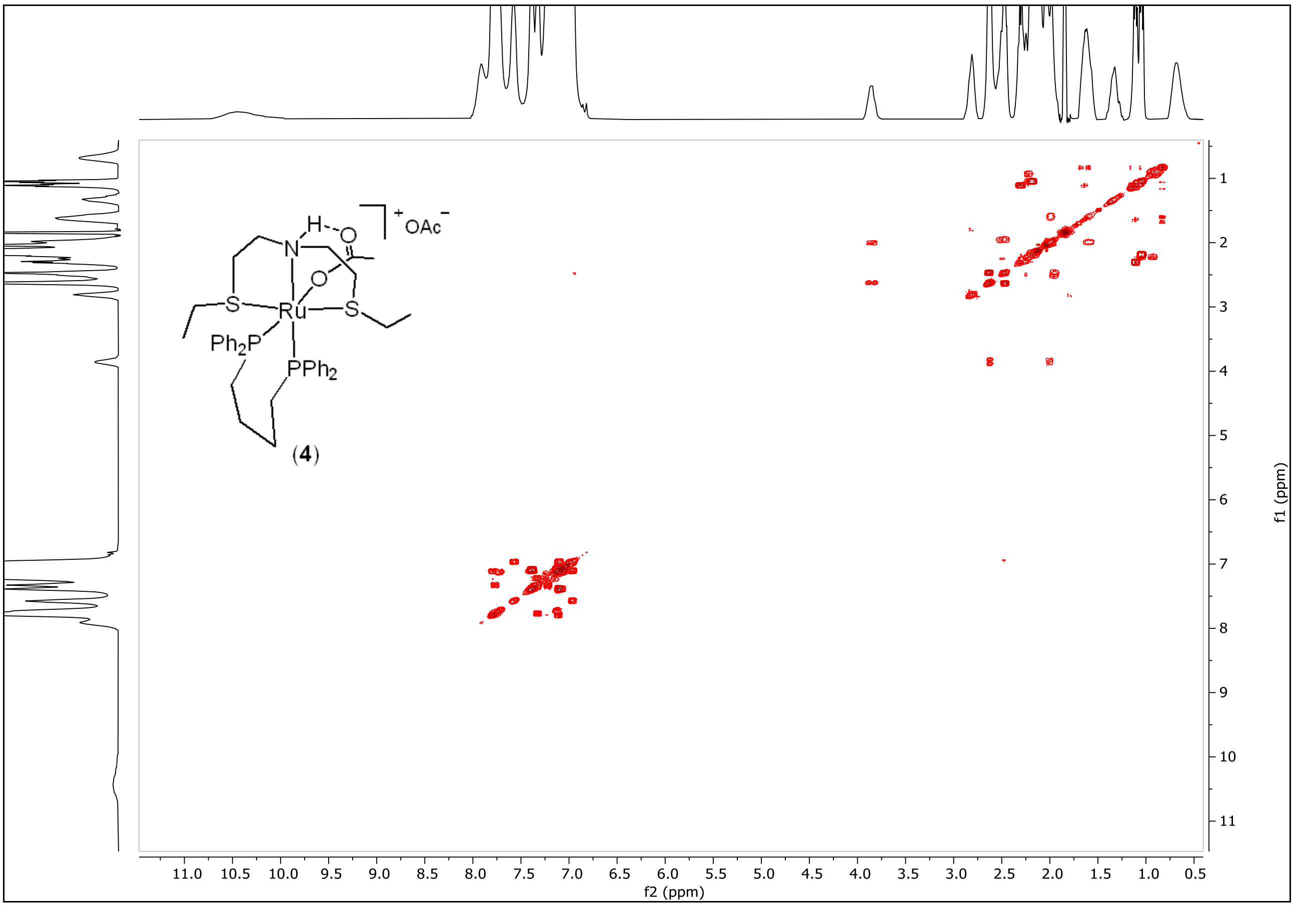

Figure S30. ^{1}H-^{1}H COSY 2D NMR spectrum of *cis*-[Ru(η^1-OAc)(SNS)(dppb)] (**4**) in toluene-d^8 at 25 °C.

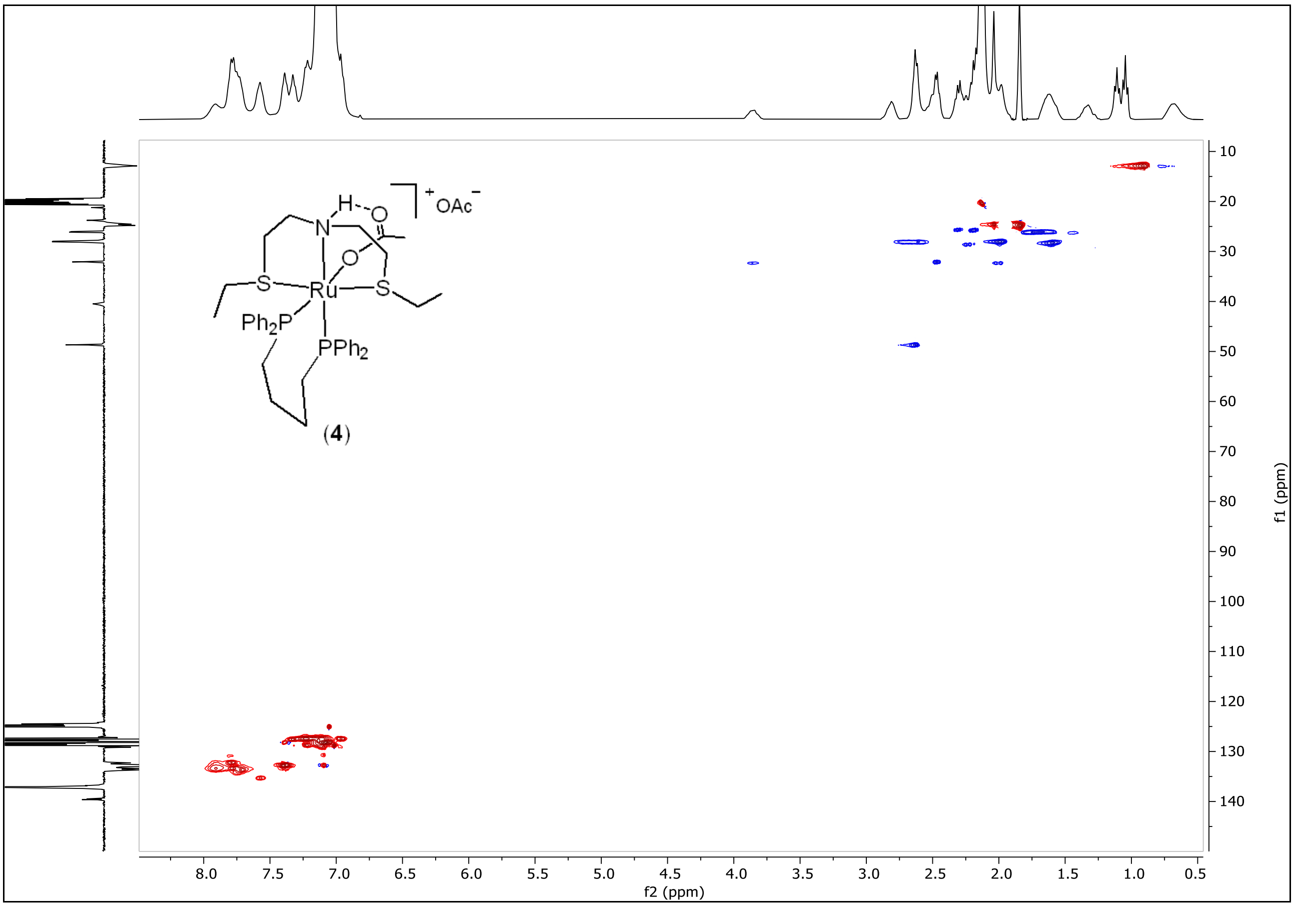

Figure S31. ^{1}H-^{13}C HSQC 2D NMR spectrum of *cis*-[Ru(η^1-OAc)(SNS)(dppb)] (**4**) in toluene-d^8 at 25 °C.

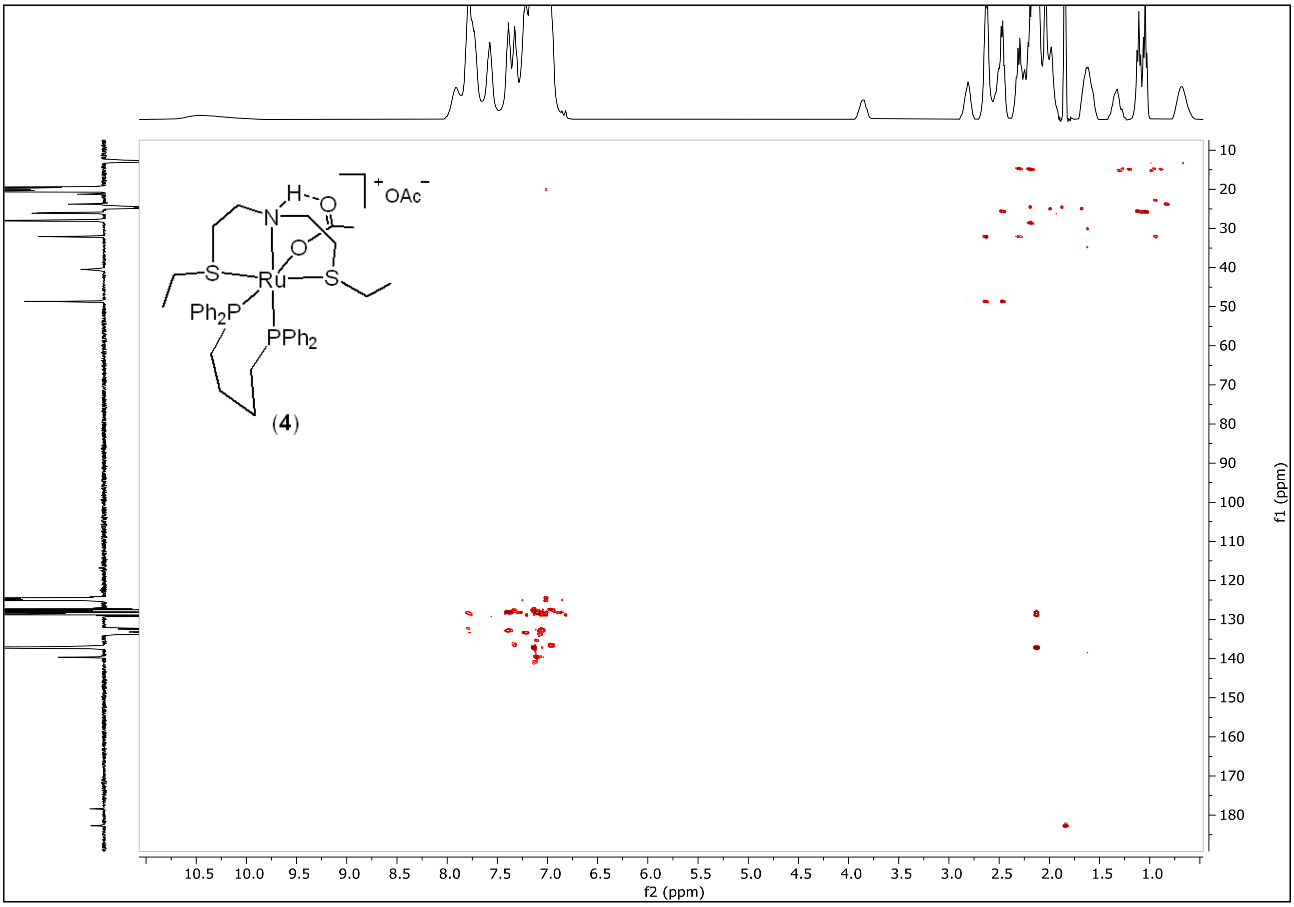

Figure S32. ^{1}H-^{13}C HMBC 2D NMR spectrum of *cis*-[Ru(η^{1}-OAc)(SNS)(dppb)] (**4**) in toluene-d^{8} at 25 °C.

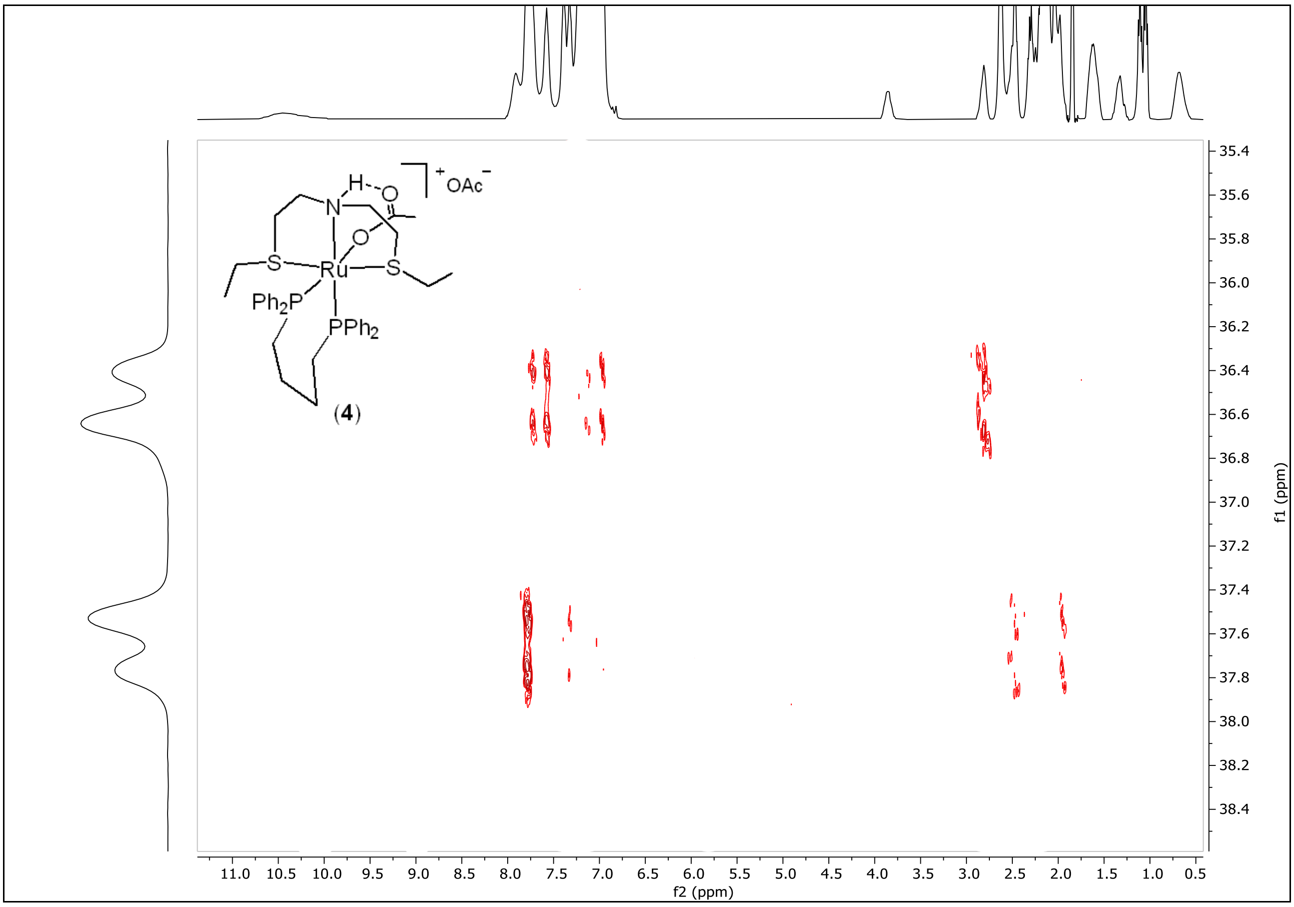

Figure S33. ^{1}H-^{31}P HMBC 2D NMR spectrum of *cis*-[Ru(η^{1}-OAc)(SNS)(dppb)] (**4**) in toluene-d^{8} at 25 °C.

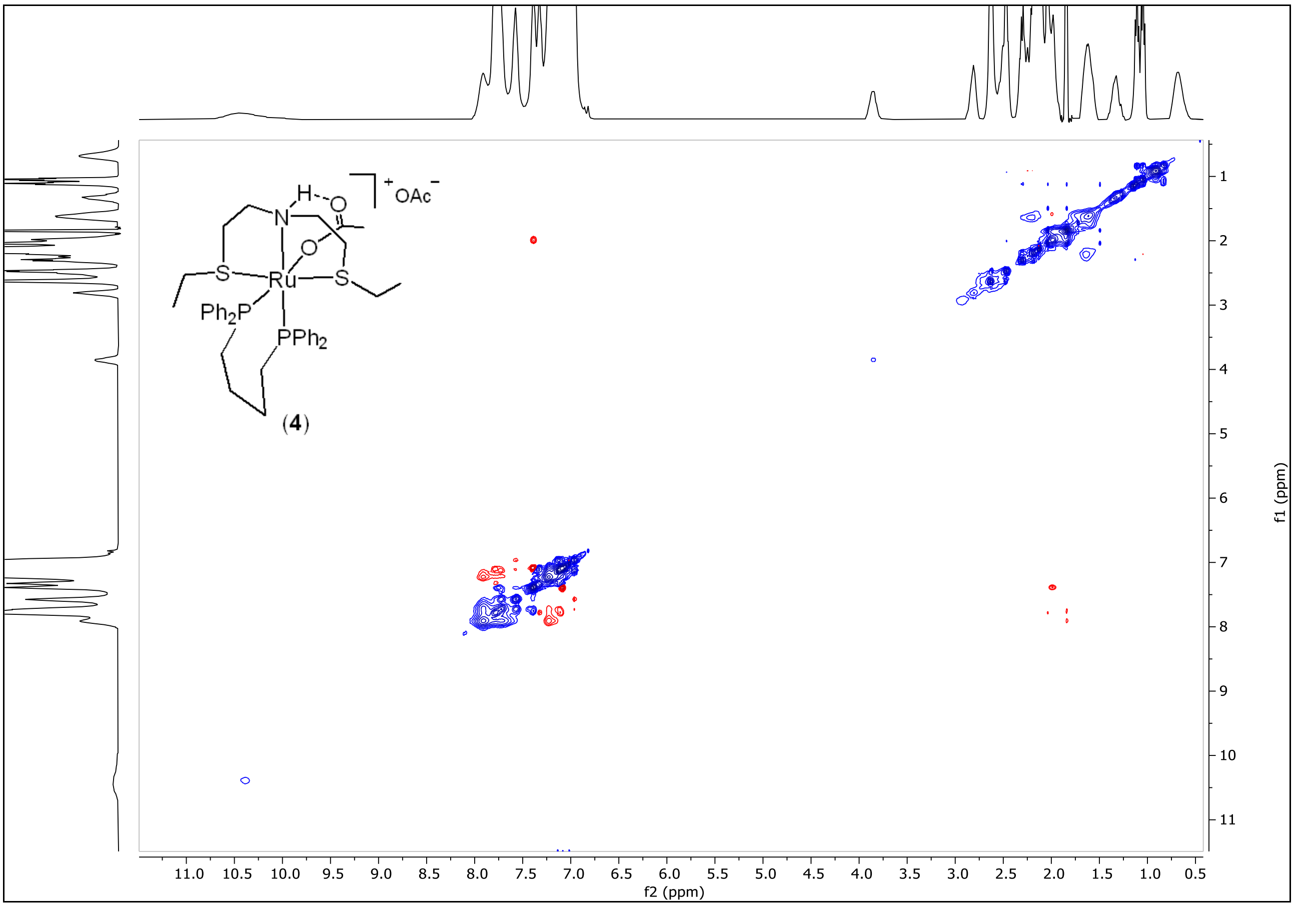

Figure S34. ^{1}H-^{1}H NOESY 2D NMR spectrum of *cis*-[Ru(η[1]-OAc)(SNS)(dppb)] (**4**) in toluene-d^8 at 25 °C.

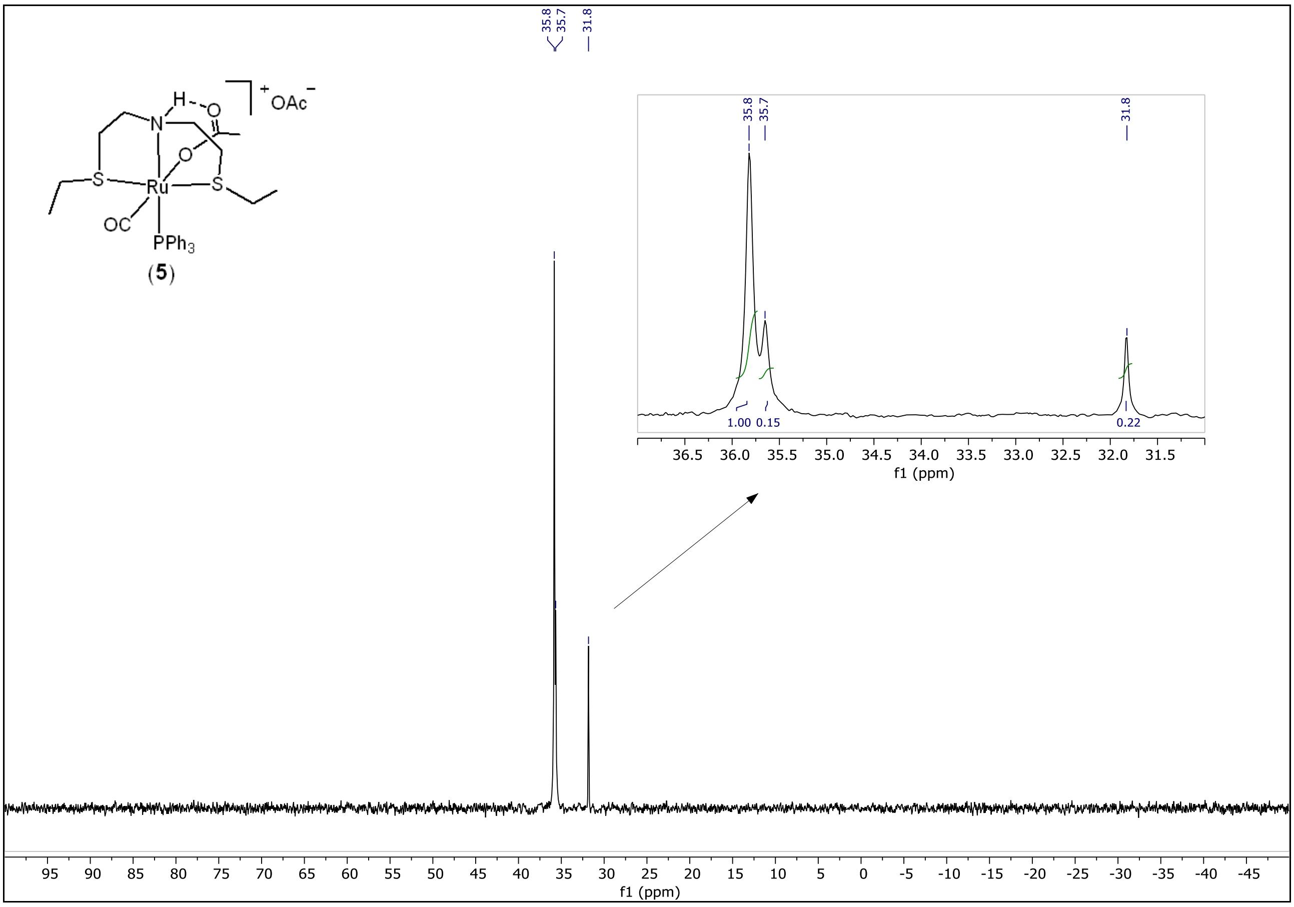

Figure S35. $^{31}P\{^1H\}$ NMR spectrum (162.0 MHz) of [Ru(η^1-OAc)(CO)(SNS)(PPh_3)]OAc (**5**) in CD_3OD at 25 °C.

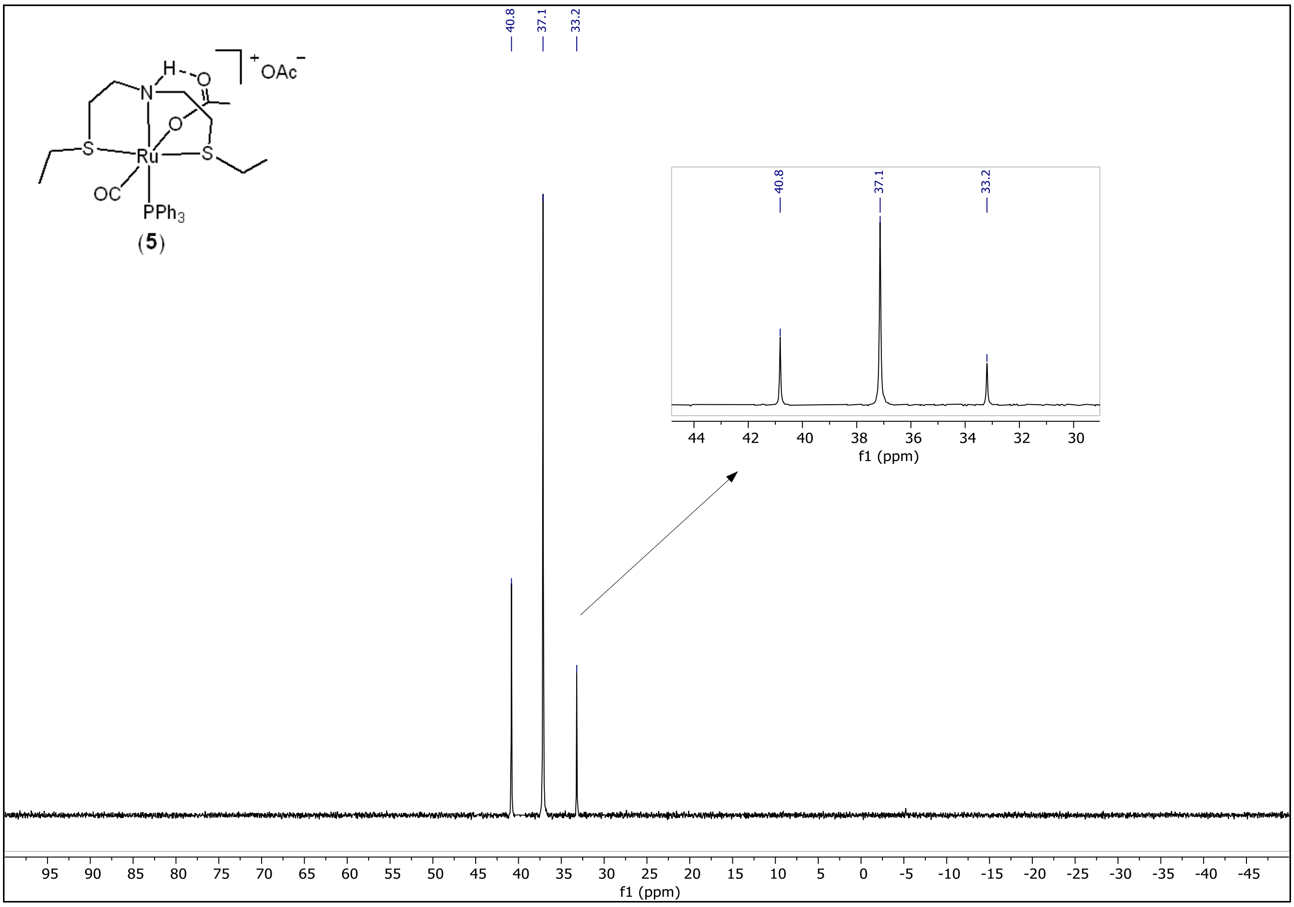

Figure S36. $^{31}P\{^{1}H\}$ NMR spectrum (162.0 MHz) of [Ru(η^1-OAc)(CO)(SNS)(PPh_3)]OAc (**5**) in toluene-d^8 at 25 °C.

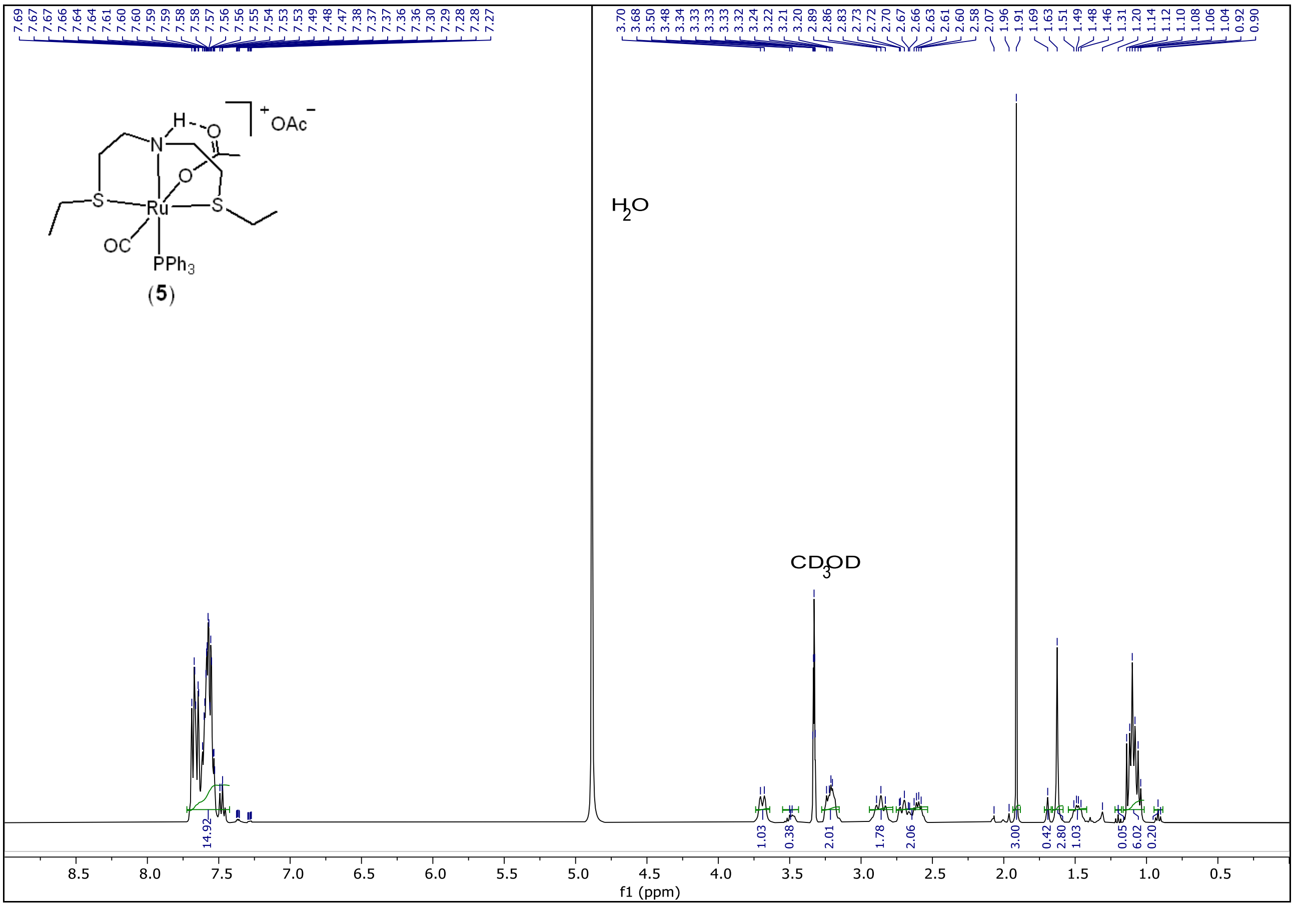

Figure S37. ^{1}H NMR spectrum (400.1 MHz) of [Ru(η^1-OAc)(CO)(SNS)(PPh$_3$)]OAc (**5**) in CD_3OD at 25 °C.

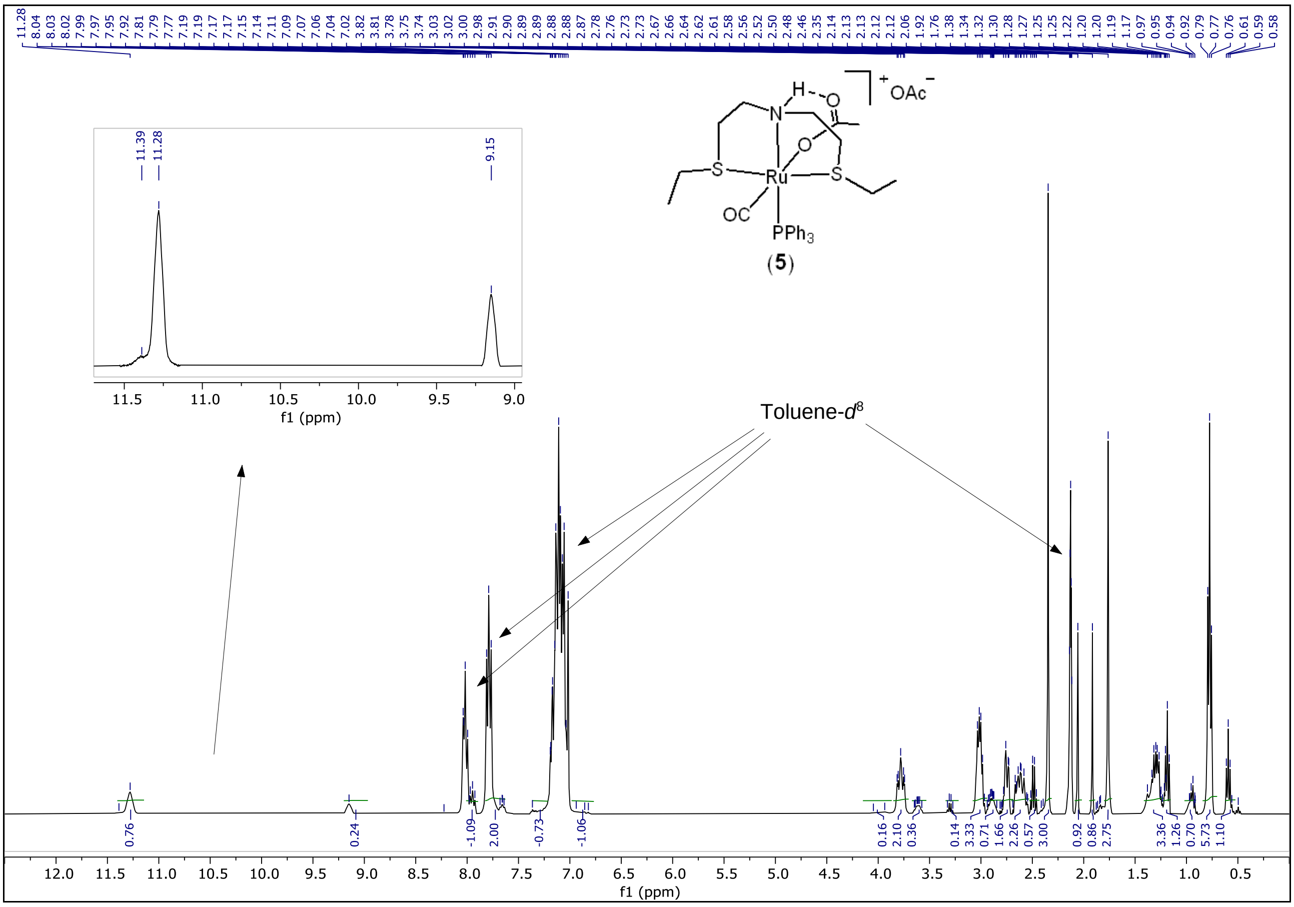

Figure S38. ^{1}H NMR spectrum (400.1 MHz) of [Ru(η^1-OAc)(CO)(SNS)(PPh$_3$)]OAc (**5**) in toluene-d^8 at 25 °C.

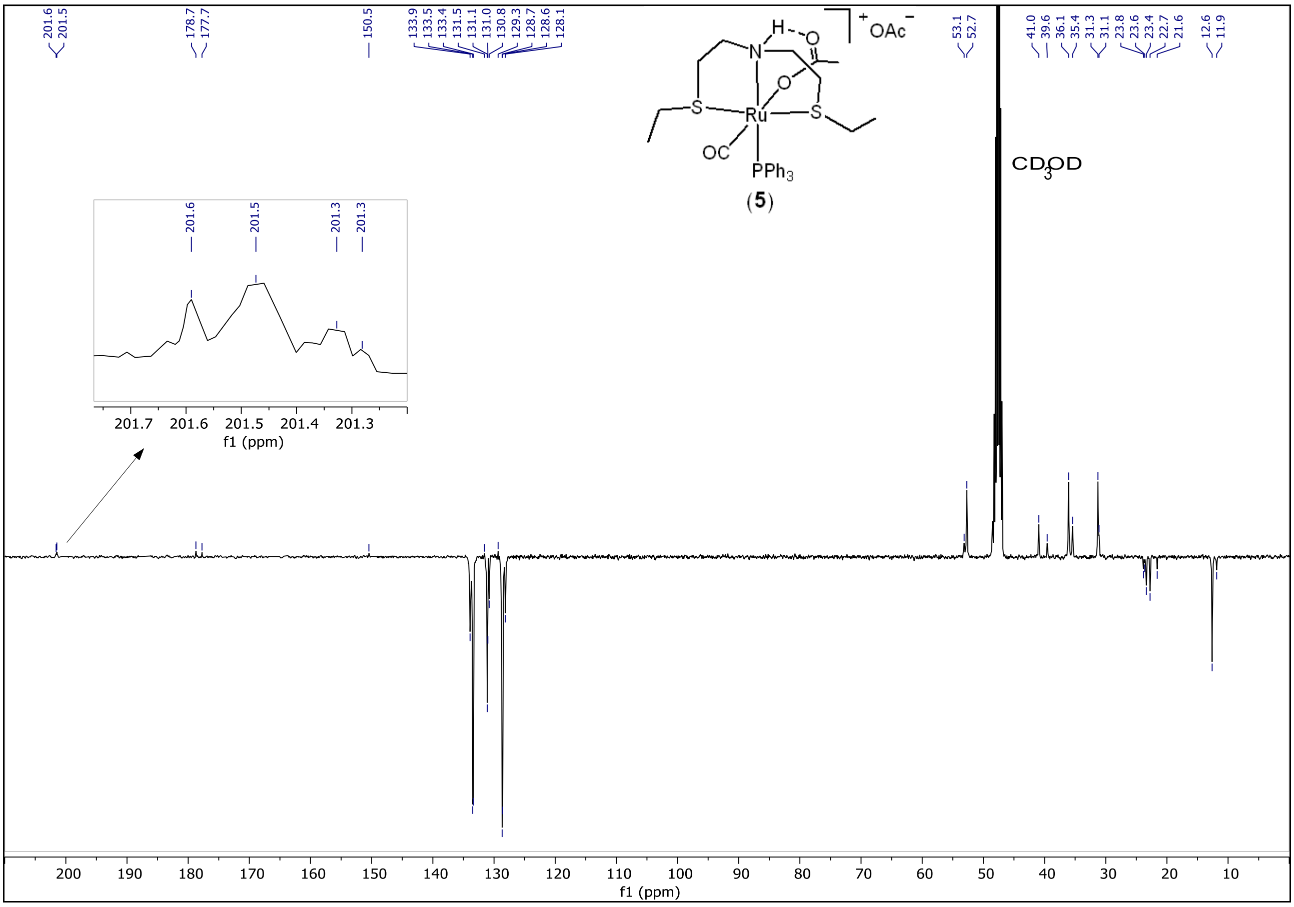

Figure S39. $^{13}C\{^{1}H\}$ DEPTQ NMR spectrum (100.6 MHz) of [Ru(η^{1}-OAc)(CO)(SNS)(PPh_3)]OAc (**5**) in CD_3OD at 25 °C.

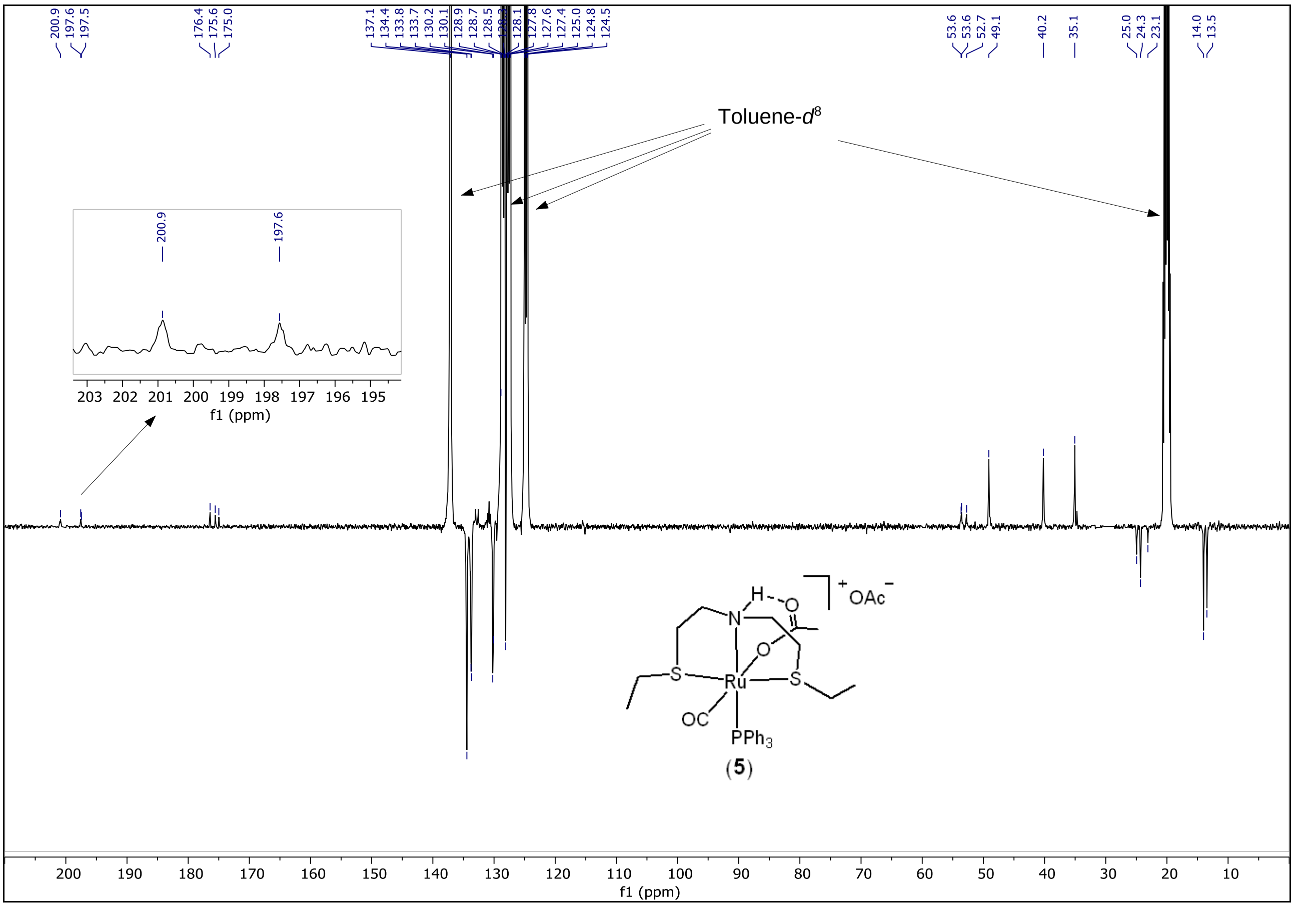

Figure S40. $^{13}C\{^{1}H\}$ DEPTQ NMR spectrum (100.6 MHz) of [Ru(η^1-OAc)(CO)(SNS)(PPh_3)]OAc (**5**) in toluene-d^8 at 25 °C.

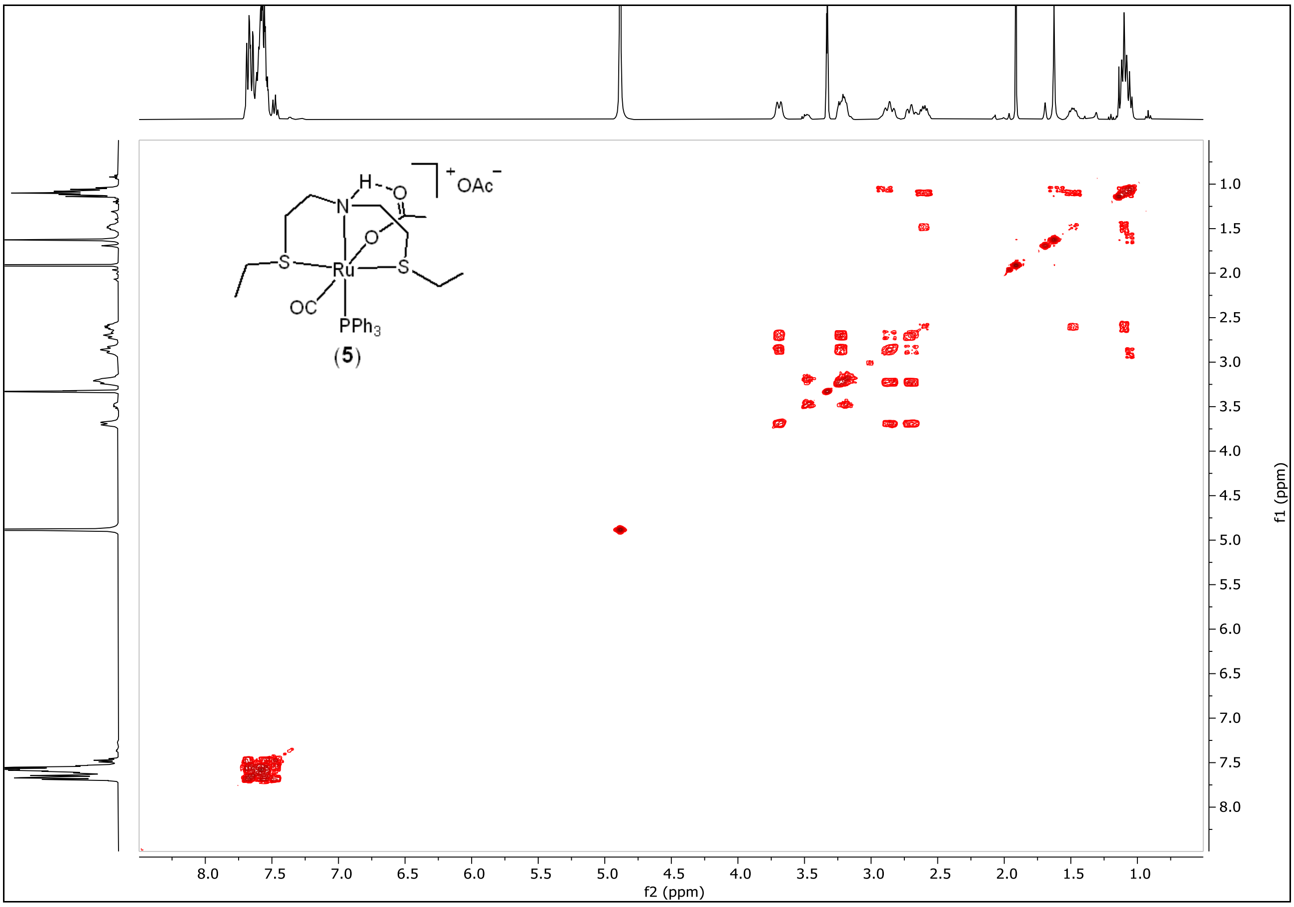

Figure S41. ^{1}H-^{1}H COSY 2D NMR spectrum of [Ru(η^{1}-OAc)(CO)(SNS)(PPh_3)]OAc (**5**) in CD_3OD at 25 °C.

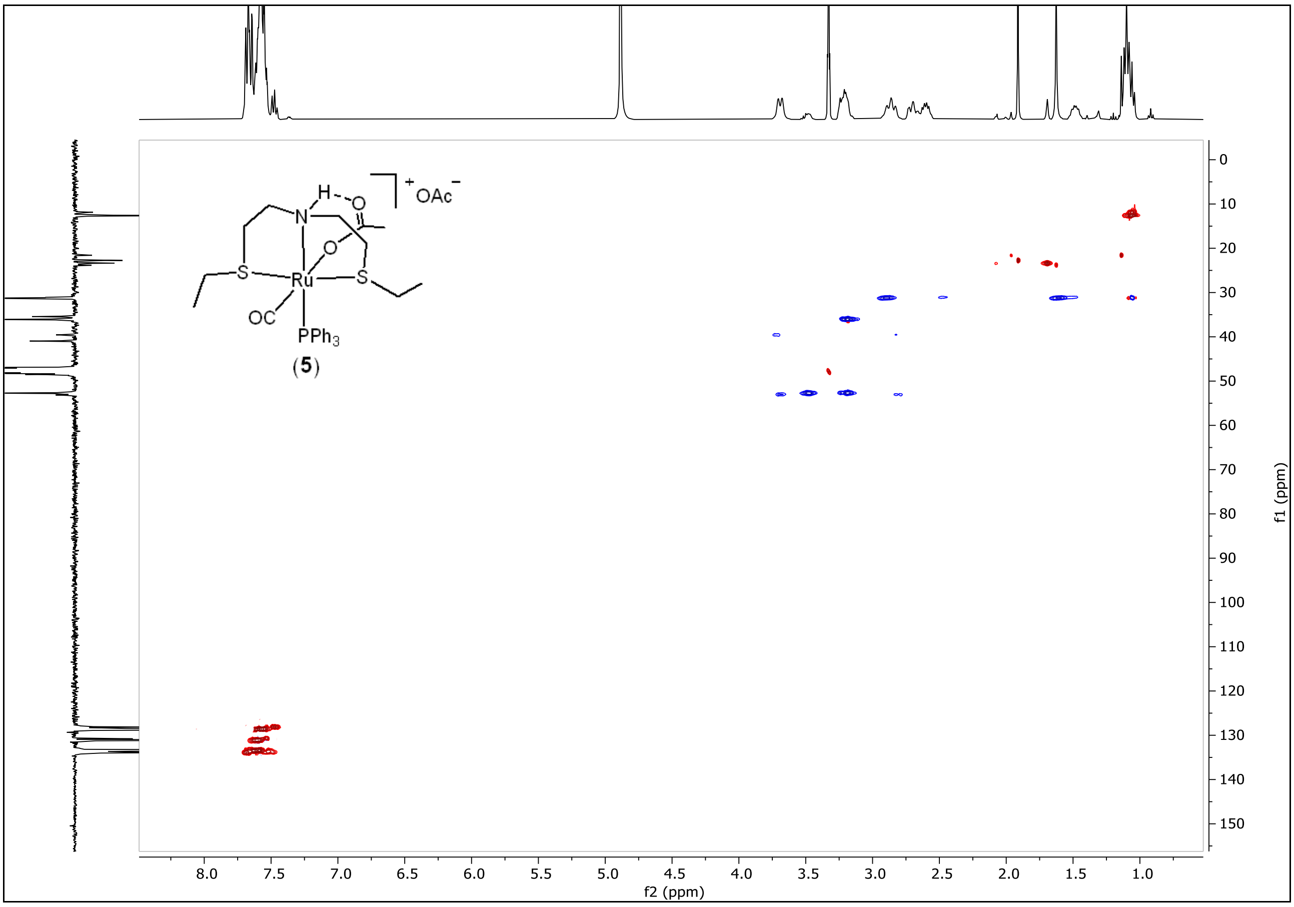

Figure S42. ¹H-¹³C HSQC 2D NMR spectrum of [Ru(η^1-OAc)(CO)(SNS)(PPh_3)]OAc (**5**) in CD_3OD at 25 °C.

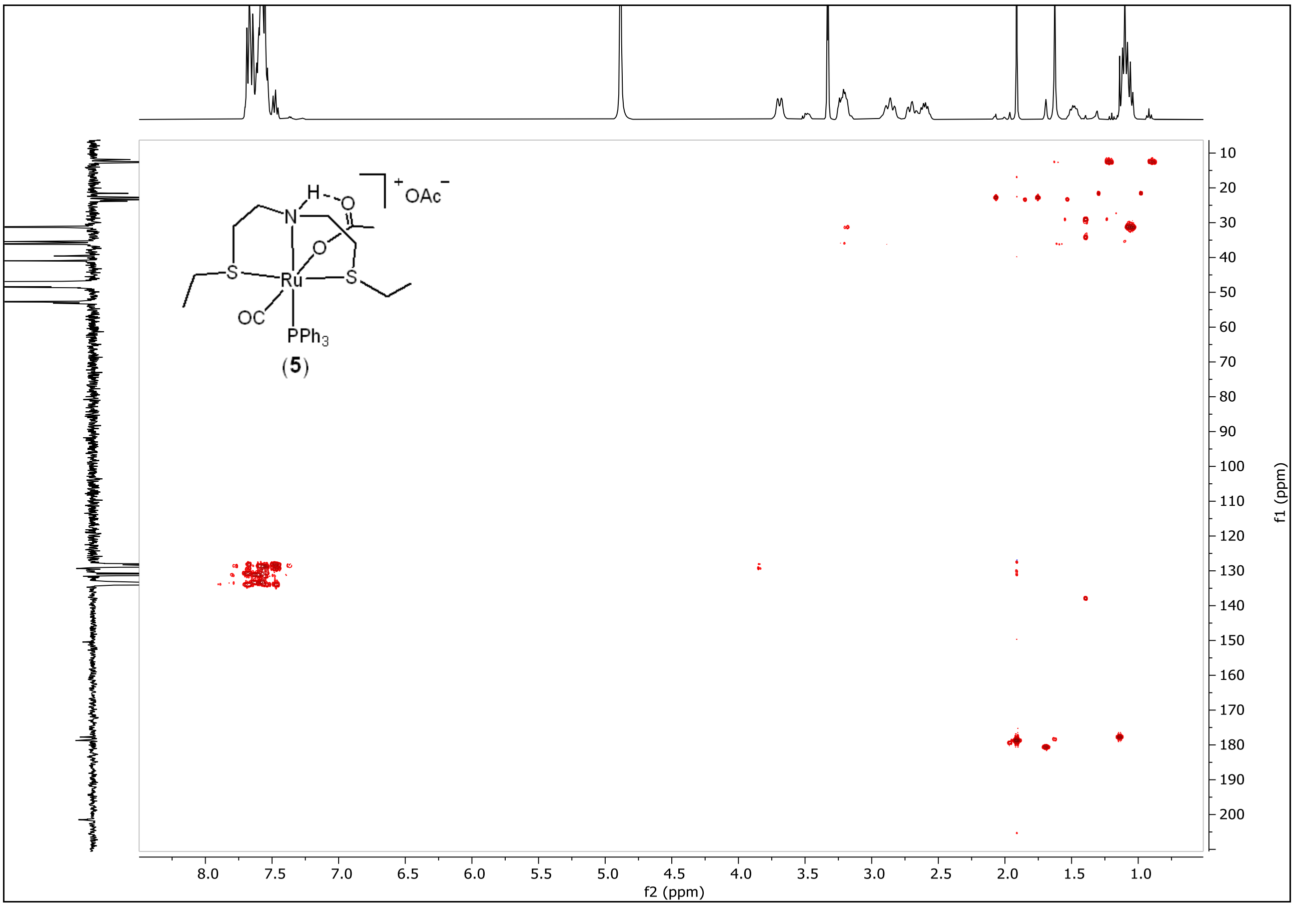

Figure S43. ^{1}H-^{13}C HMBC 2D NMR spectrum of $[Ru(\eta^1\text{-OAc})(CO)(SNS)(PPh_3)]OAc$ (**5**) in CD_3OD at 25 °C.

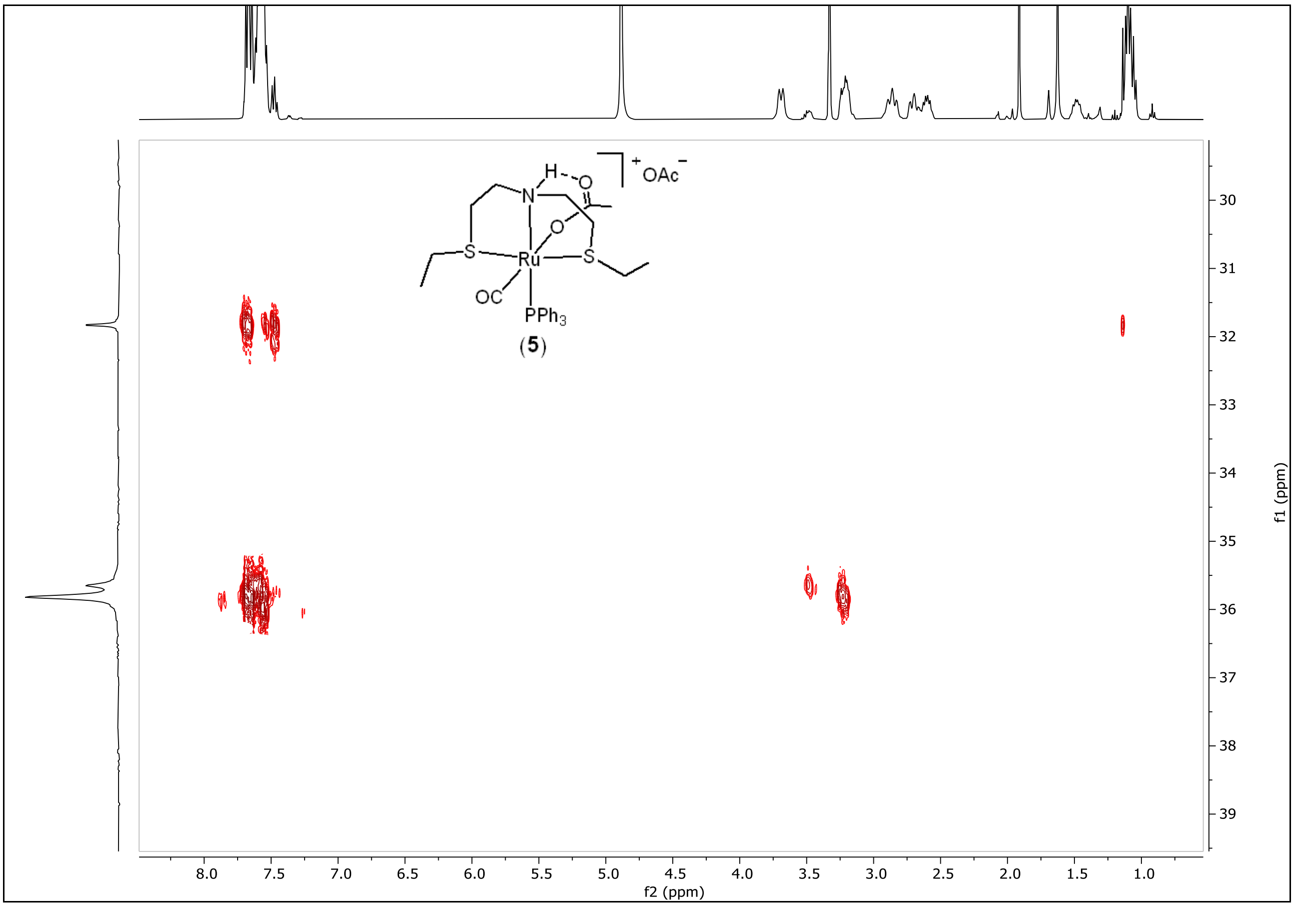

Figure S44. ^{1}H-^{31}P HMBC 2D NMR spectrum of [Ru(η^1-OAc)(CO)(SNS)(PPh_3)]OAc (**5**) in CD_3OD at 25 °C.

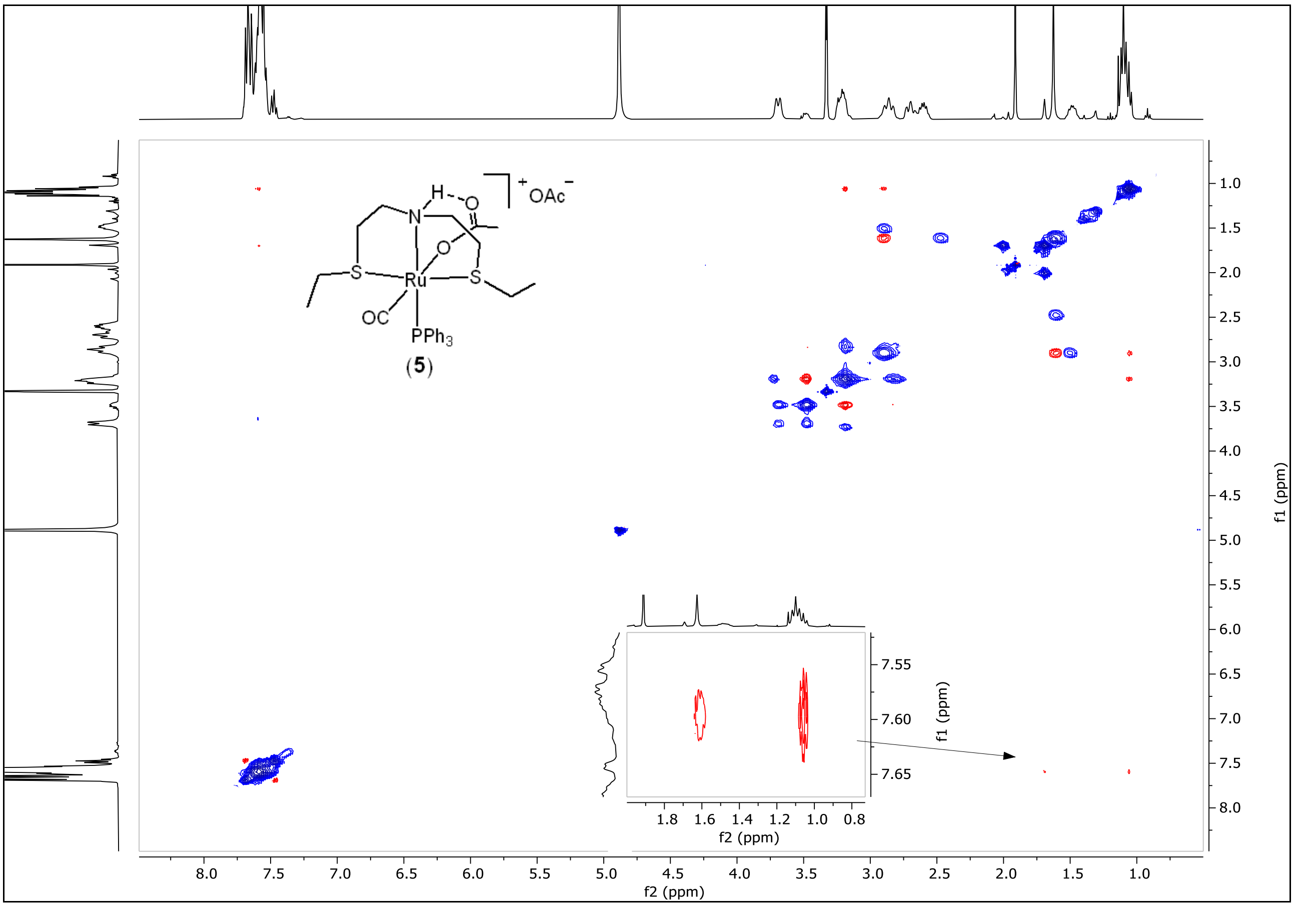

Figure S45. ^{1}H-^{1}H NOESY 2D NMR spectrum of [Ru(η^1-OAc)(CO)(SNS)(PPh_3)]OAc (**5**) in CD_3OD at 25 °C.

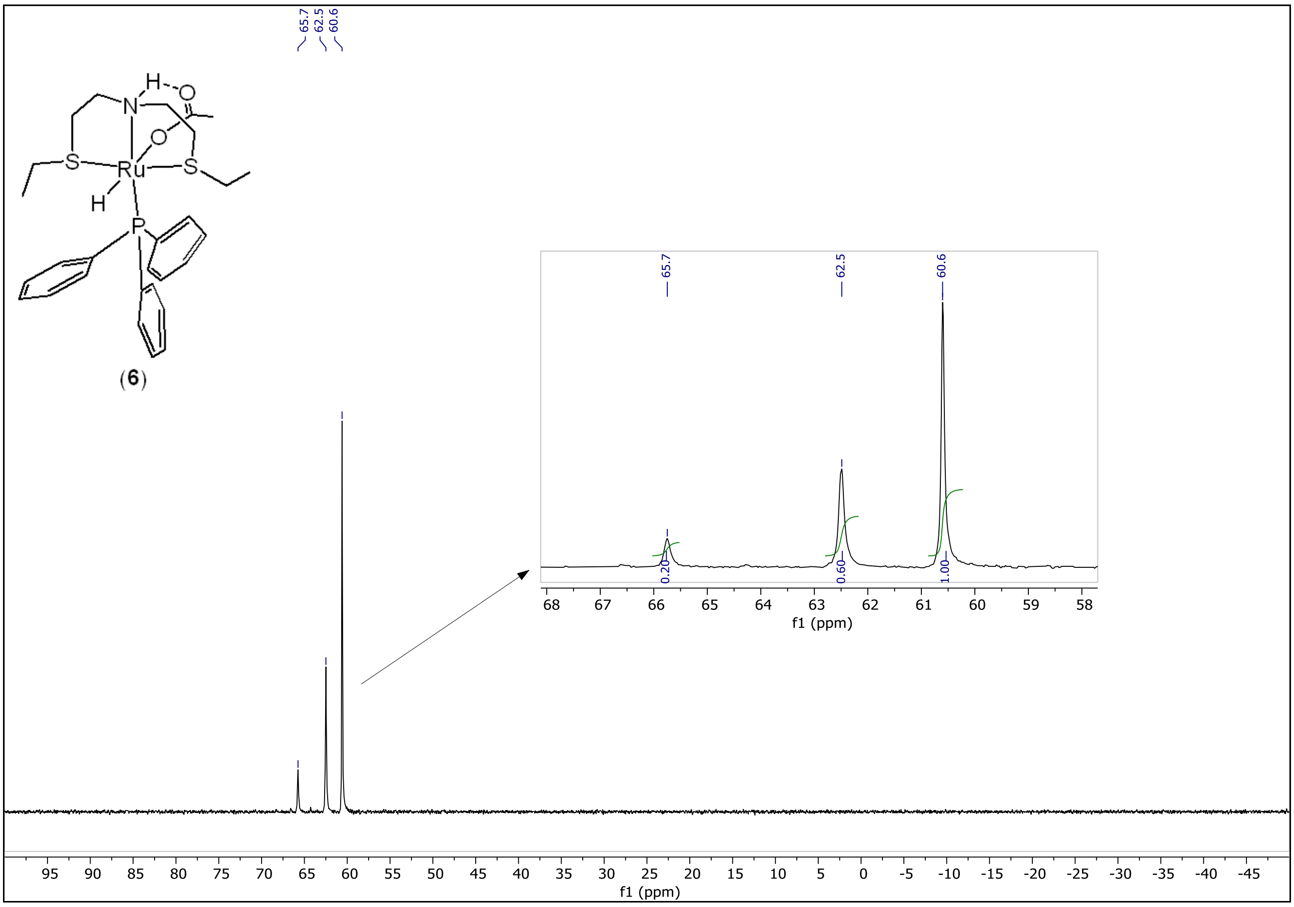

Figure S46. $^{31}P\{^{1}H\}$ NMR spectrum (162.0 MHz) of *trans*-[RuH(η^1-OAc)(SNS)(PPh_3)] (**6**) in toluene-d^8 at 25 °C.

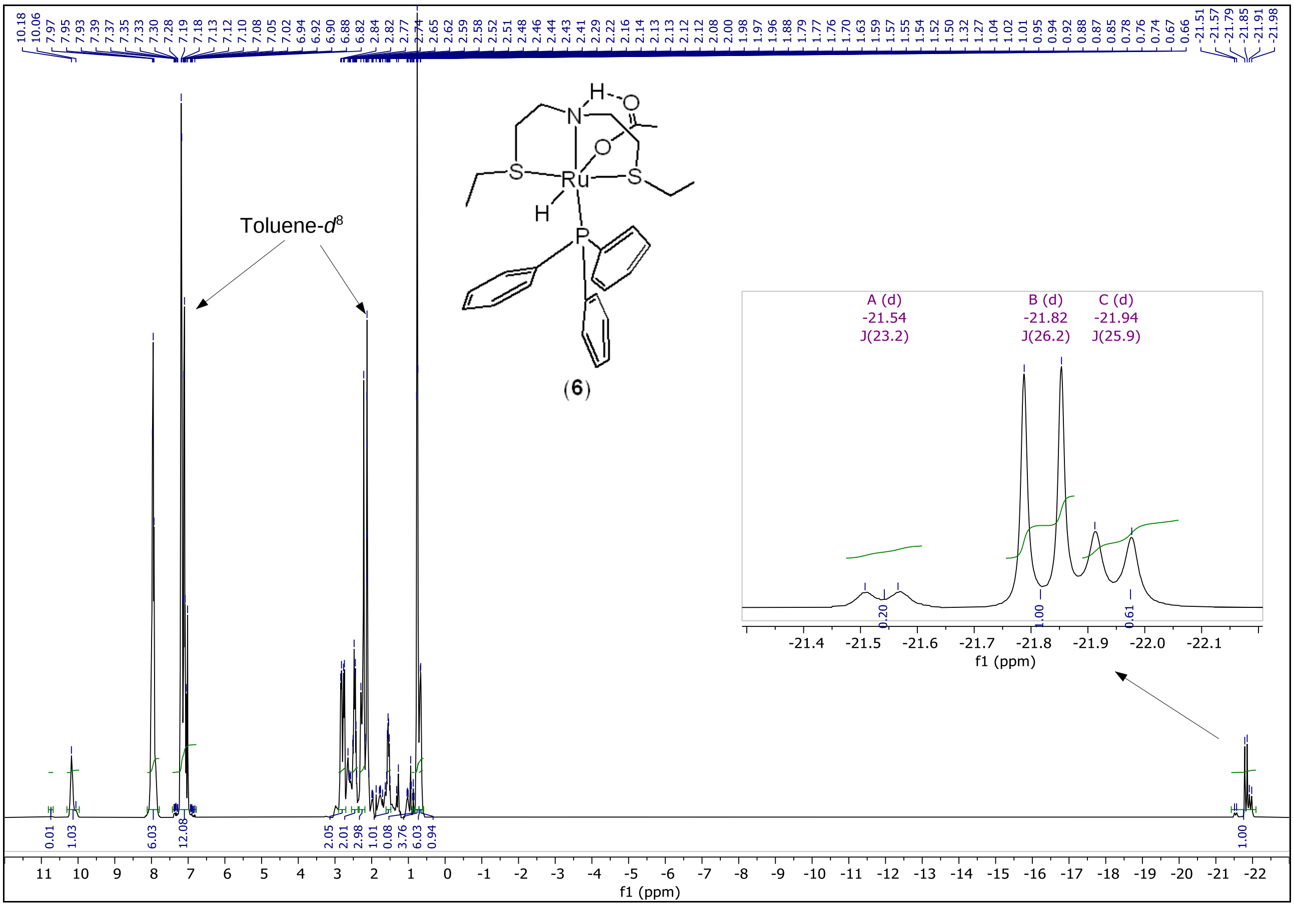

Figure S47. ^{1}H NMR spectrum (400.1 MHz) of *trans*-[RuH(η^1-OAc)(SNS)(PPh_3)] (**6**) in toluene-d^8 at 25 °C.

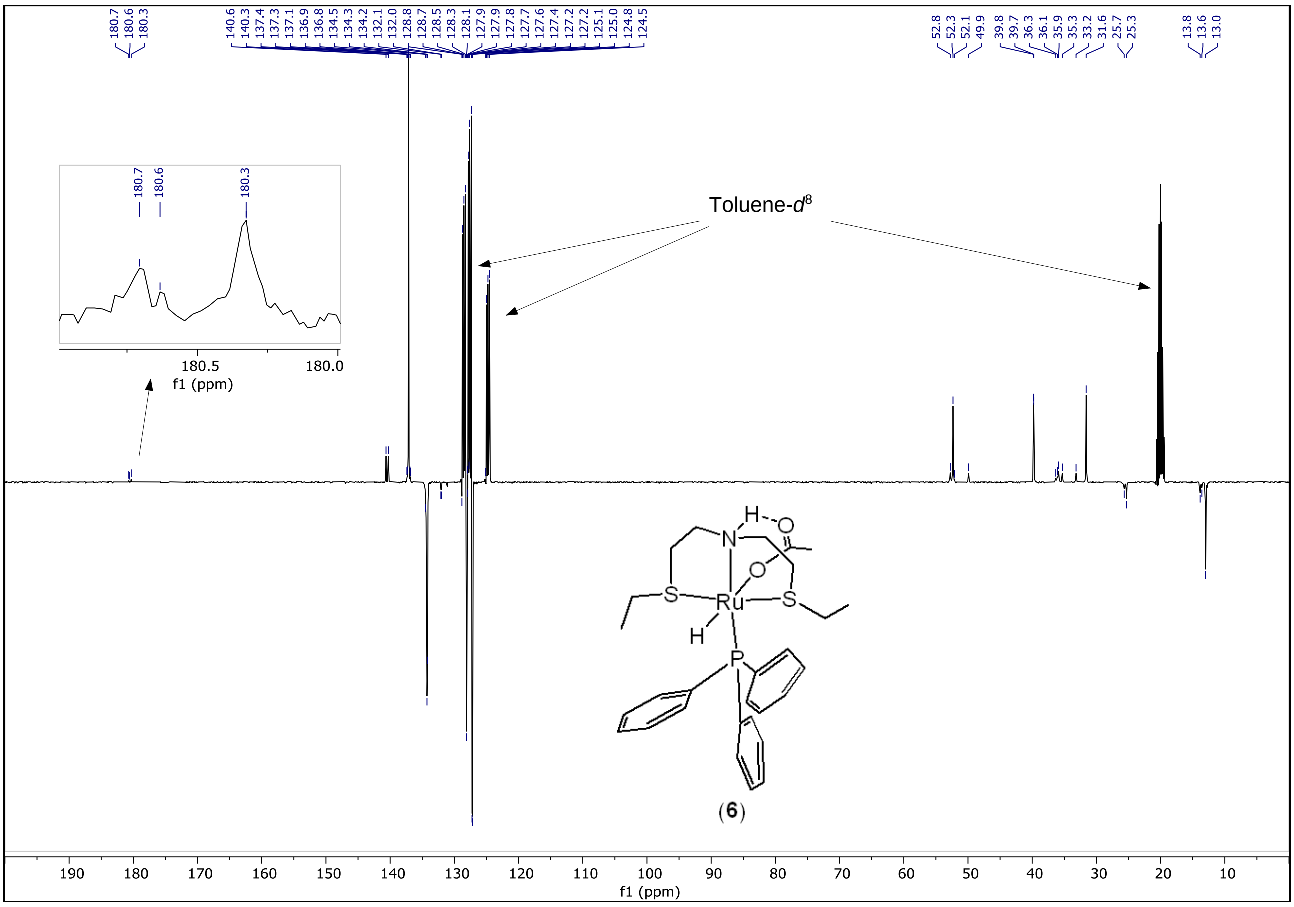

Figure S48. $^{13}C\{^{1}H\}$ DEPTQ NMR spectrum (100.6 MHz) of *trans*-[RuH(η^1-OAc)(SNS)(PPh_3)] (**6**) in toluene-d^8 at 25 °C.

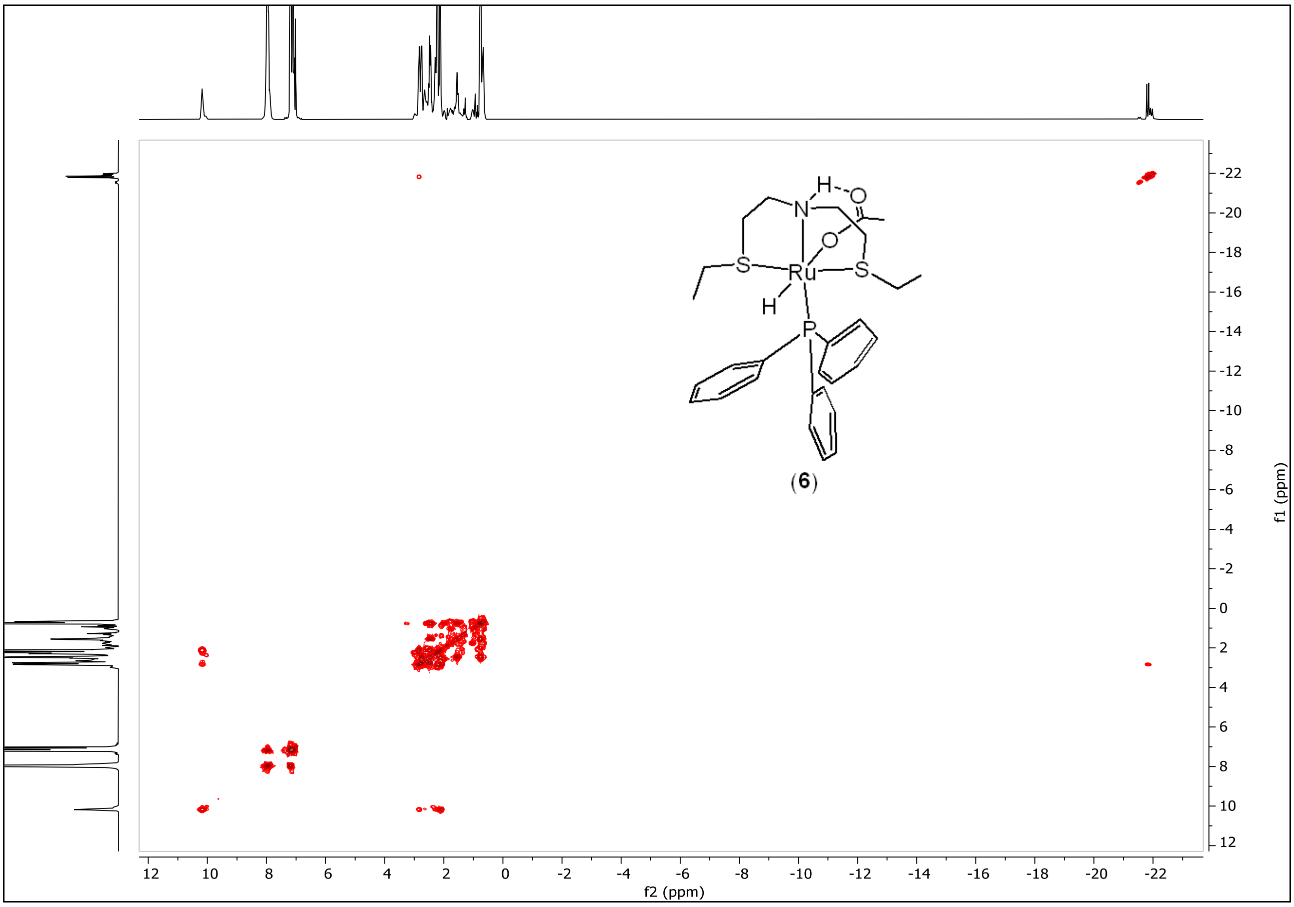

Figure S49. ^{1}H-^{1}H COSY 2D NMR spectrum of *trans*-[RuH(η^{1}-OAc)(SNS)(PPh$_3$)] (**6**) in toluene-d^8 at 25 °C.

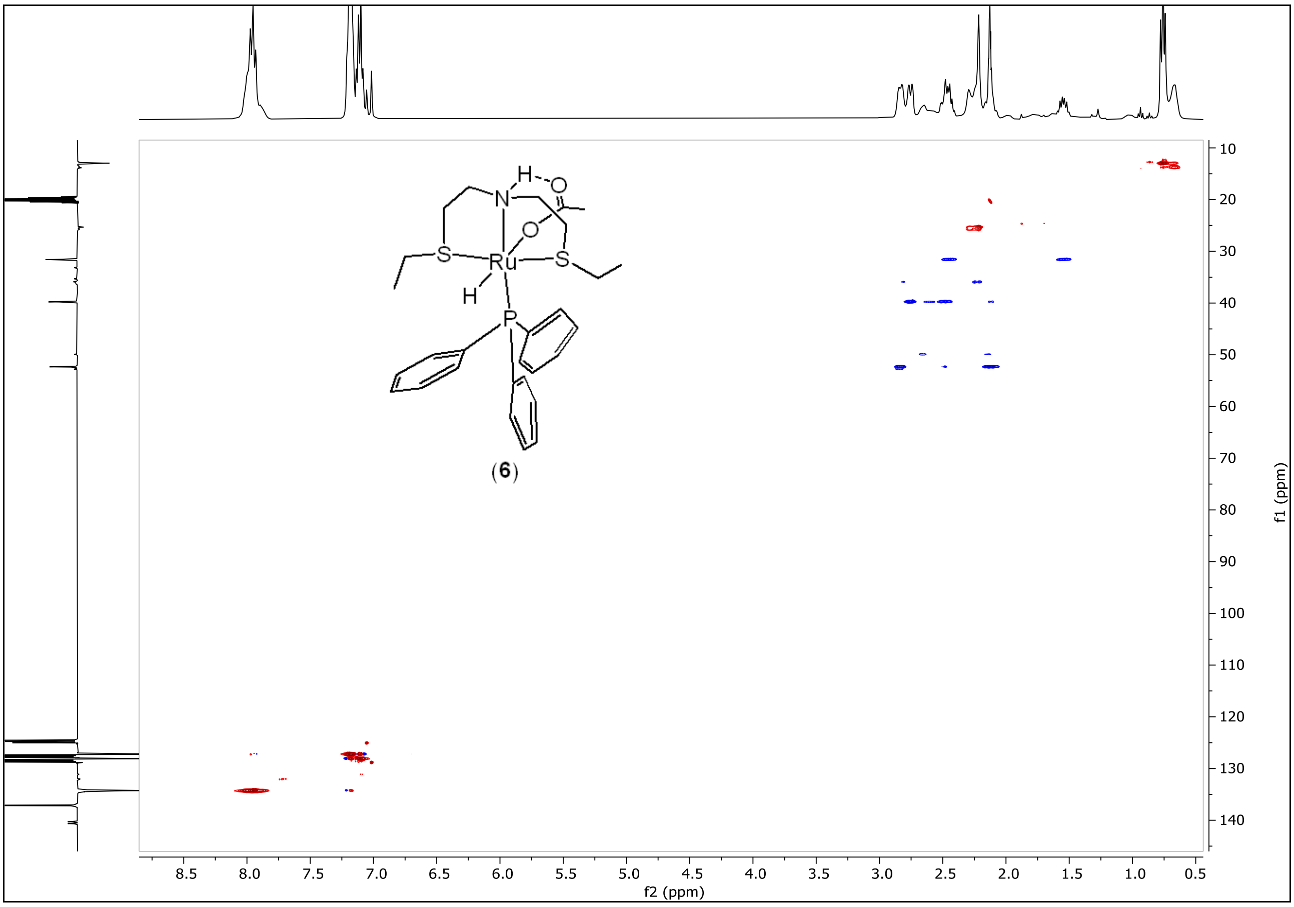

Figure S50. ^{1}H-^{13}C HSQC 2D NMR spectrum of *trans*-[RuH(η^1-OAc)(SNS)(PPh_3)] (**6**) in toluene-d^8 at 25 °C.

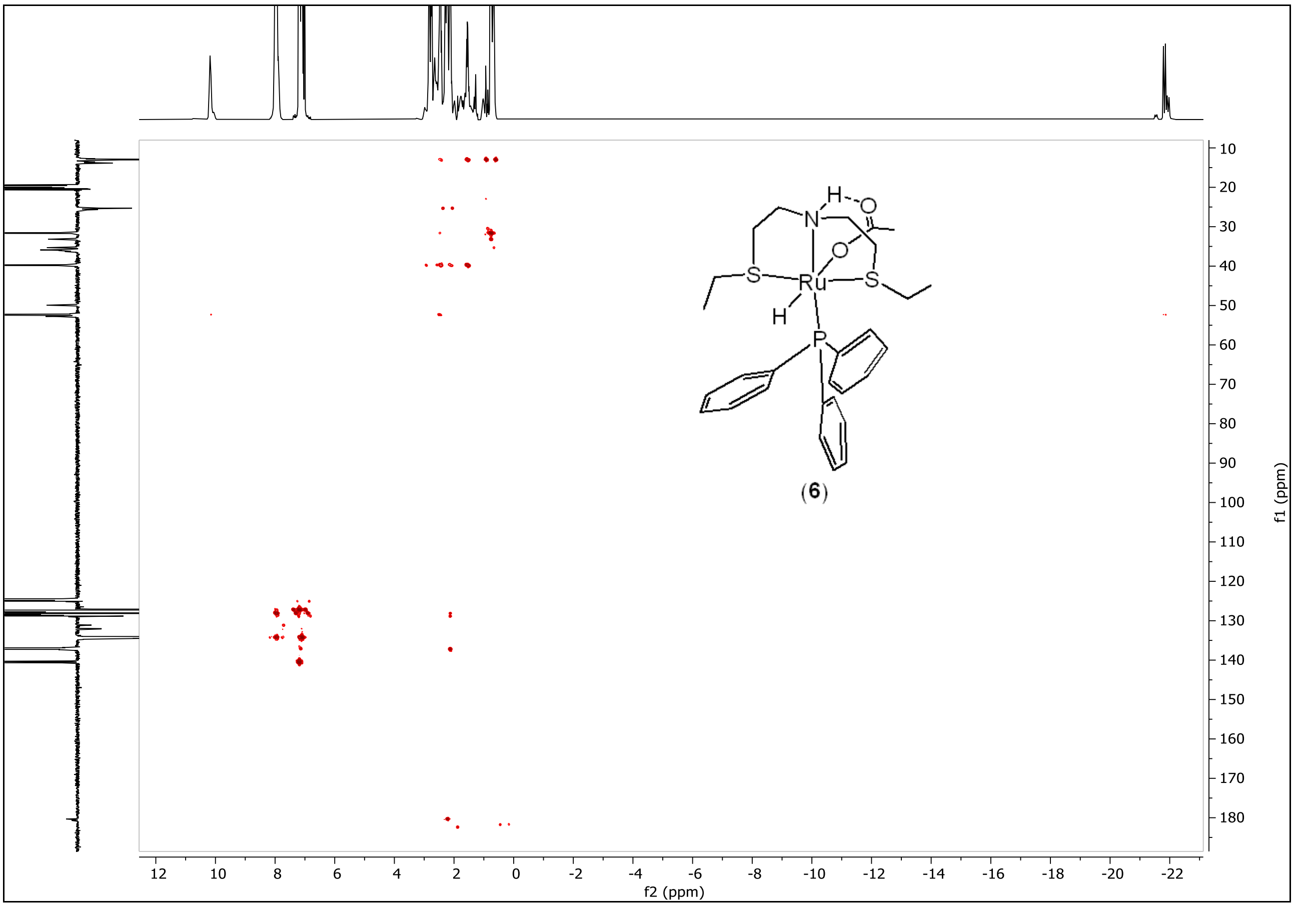

Figure S51. ^{1}H-^{13}C HMBC 2D NMR spectrum of *trans*-[RuH(η^{1}-OAc)(SNS)(PPh$_3$)] (**6**) in toluene-*d*^{8} at 25 °C.

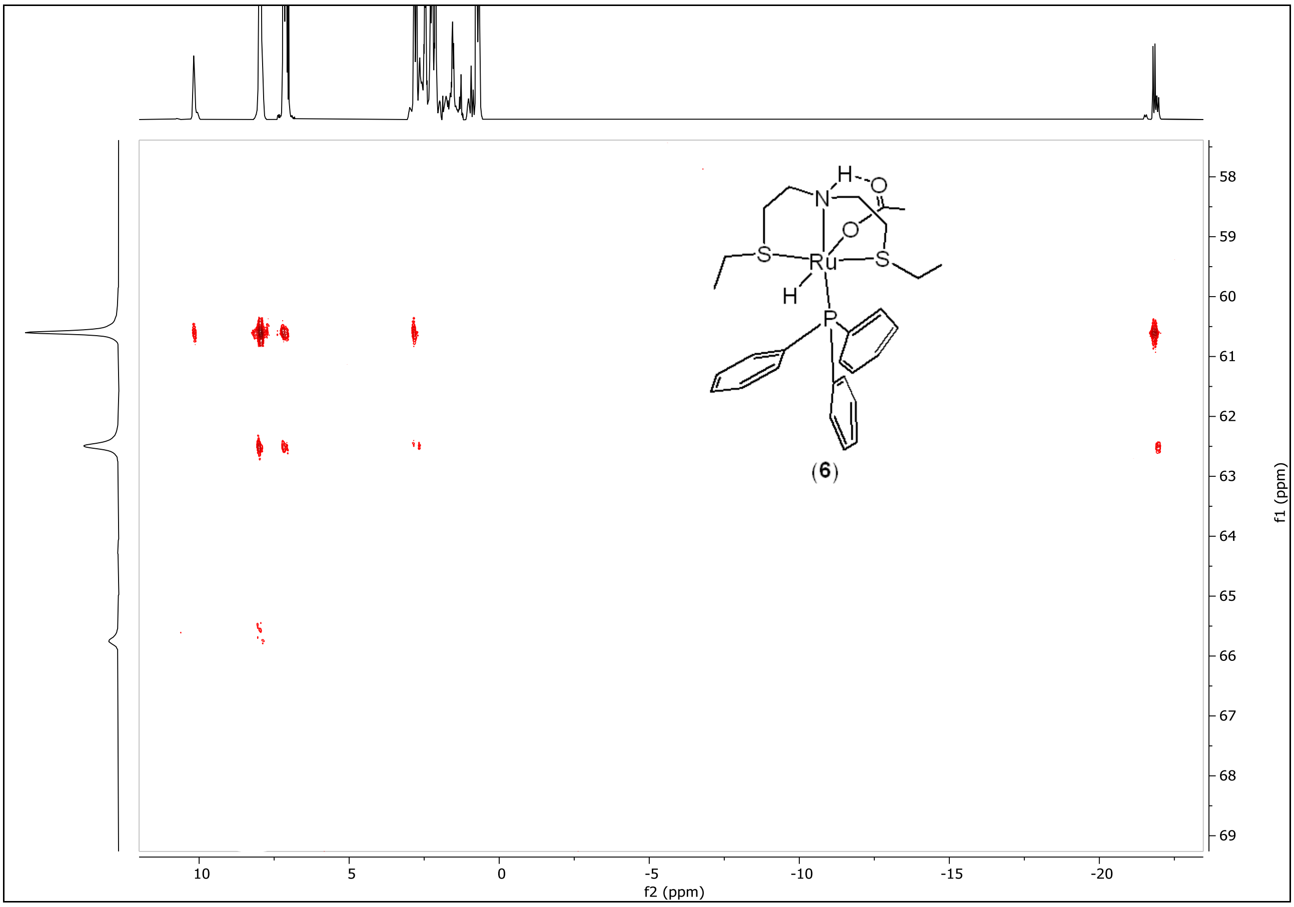

Figure S52. ^{1}H-^{31}P HMBC 2D NMR spectrum of *trans*-[RuH(η^1-OAc)(SNS)(PPh_3)] (**6**) in toluene-d^8 at 25 °C.

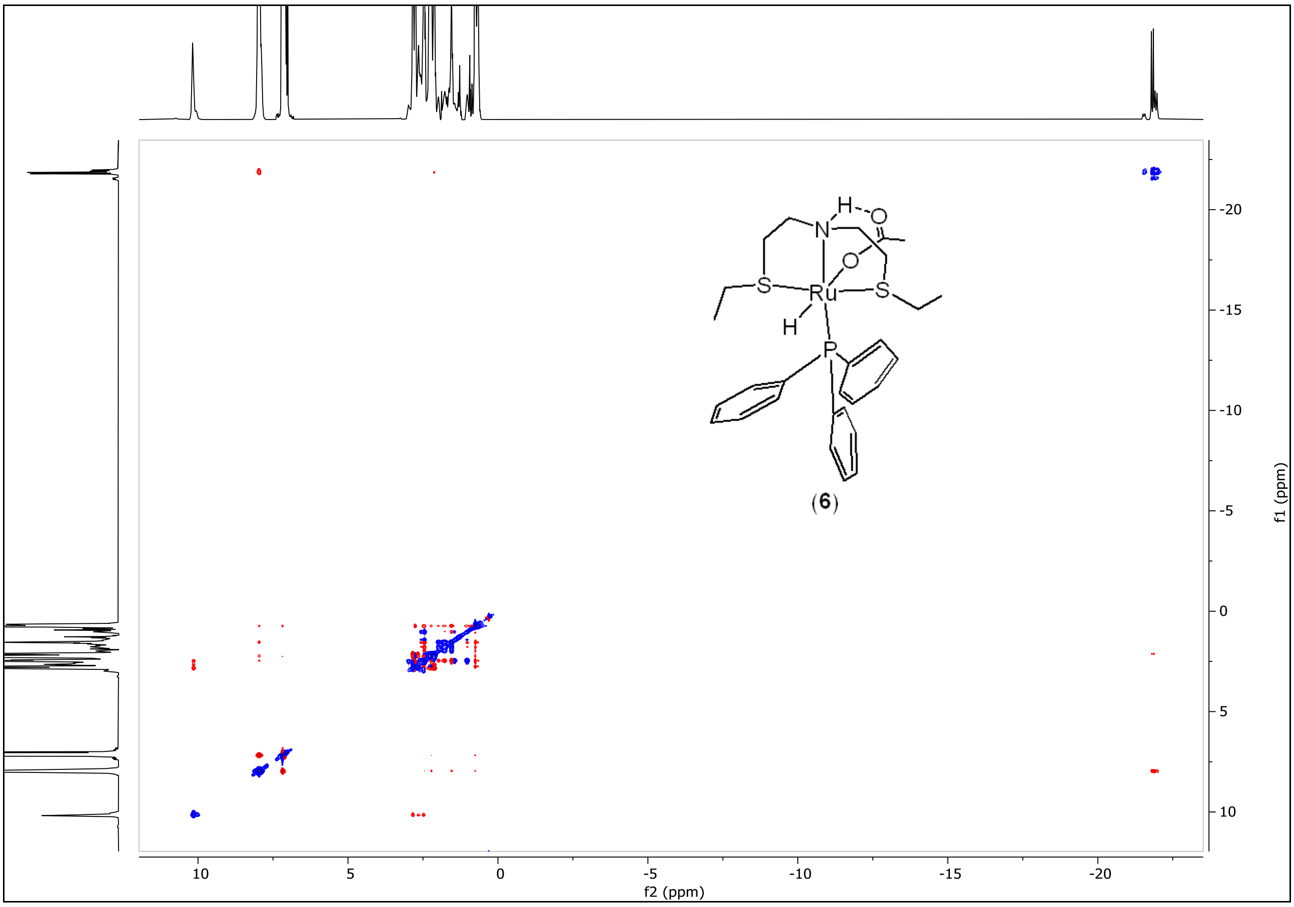

Figure S53. ^{1}H-^{1}H NOESY 2D NMR spectrum of *trans*-[RuH(η^1-OAc)(SNS)(PPh_3)] (**6**) in toluene-d^8 at 25 °C.

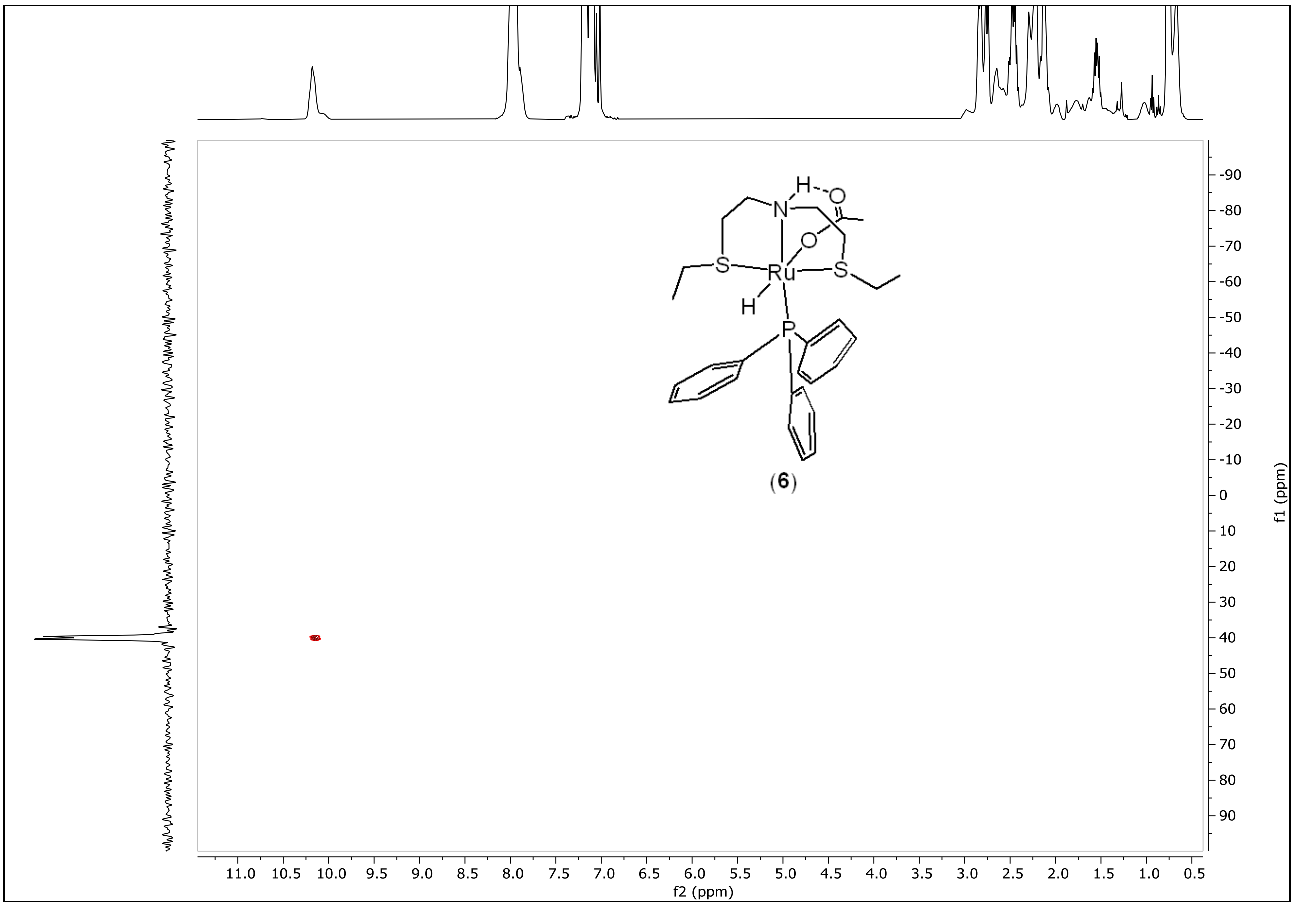

Figure S54. ^{1}H-^{15}N HSQC 2D NMR spectrum of *trans*-[RuH(η^1-OAc)(SNS)(PPh_3)] (**6**) in toluene-d^8 at 25 °C.

Table S1. Further data regarding the catalytic HY of methyl decanoate **a** (2-20 mmol) with complexes **1**-**5** and the *trans*-[$RuCl_2$(SNS)(PPh_3)] derivative with different bases (50 mol%) at 27.5 bar of H_2 in presence or without solvents after 16 h.

Entry	Complex	S/C	Base	Solvent	Temp. [ºC]	Conv.[a] [%]
1	**1**	10000	NaOMe	-	90	33.6
2	**1**	50000	NaOMe	-	90	20.3
3	**2**	10000	NaOMe	-	90	16.5
4	**2**	50000	NaOMe	-	90	20.5
5	**2**	50000	NaOMe	Toluene	40	60.2
6	**3**	10000	NaOMe	-	90	18.1
7	**3**	50000	NaOMe	-	40	31.2
8	**3**	50000	NaOMe	-	90	16.4
9	**4**	10000	NaOMe	-	90	22.9
10	**5**	10000	NaOMe	-	90	14.8
11	**5**	50000	NaOMe	-	40	27.7
12	**5**	50000	NaOMe	-	90	27.6
13	[$RuCl_2$(SNS)(PPh_3)]	10000	NaOMe	-	90	34.2
14	[$RuCl_2$(SNS)(PPh_3)]	50000	NaOMe	-	90	21.5
15	[$RuCl_2$(SNS)(PPh_3)]	50000	NaOMe	Toluene	40	20.8
16	[$RuCl_2$(SNS)(PPh_3)]	100000	NaOEt	-	40	1.7

[a] Determined by GC analyses.

Table S2. Further data regarding the catalytic HY of esters (2-20 mmol) with complexes **1** and *trans*-[$RuCl_2$(SNS)(PPh_3)] and with base (50 mol%) at 27.5 bar of H_2 and in presence and absence of solvent after 16 h.

Entry	Complex	Substrate	S/C	base	Solvent	T [°C]	Conv.[a] [%]
1	[$RuCl_2$(SNS)(PPh_3)]	Ethyl decanoate **b**	50000	NaOEt	Toluene	40	96.1
2	[$RuCl_2$(SNS)(PPh_3)]	Ethyl dodecanoate **c**	44000	NaOEt	-	40	98.3
3	[$RuCl_2$(SNS)(PPh_3)]		100000	NaOEt	-	40	96.2
4	**1**	Ethyl 10-undecenoate **d**	10000	NaOEt	-	30	96.8
5	**1**		100000	NaOEt	-	40	28.5
6	[$RuCl_2$(SNS)(PPh_3)]		10000	NaOEt	-	30	96.1
7	[$RuCl_2$(SNS)(PPh_3)]		10000	NaOEt	-	40	98.6
8	[$RuCl_2$(SNS)(PPh_3)]		50000	NaOEt	-	40	100
9	[$RuCl_2$(SNS)(PPh_3)]		100000	NaOEt	-	40	95.6
10	[$RuCl_2$(SNS)(PPh_3)]	Methyl oleate **e**	25000	NaOMe	-	60	91.8
11	[$RuCl_2$(SNS)(PPh_3)]		50000	NaOMe	-	50	100
12	[$RuCl_2$(SNS)(PPh_3)]	Ethyl oleate **f**	50000	NaOEt	-	40	97.1
13	[$RuCl_2$(SNS)(PPh_3)]	Ethyl benzoate **h**	50000	NaOEt	-	40	97.0

[a] Determined by GC analyses.

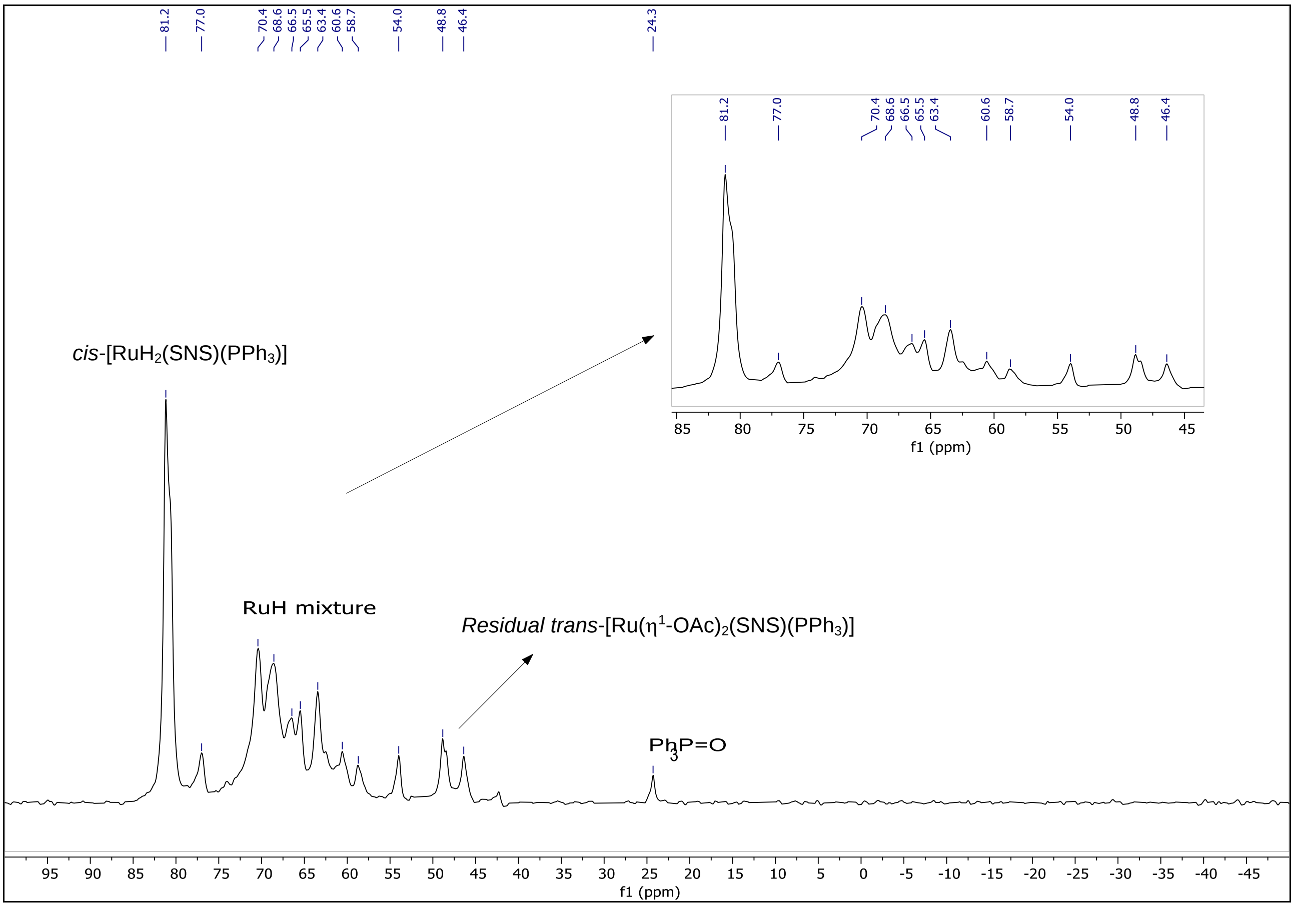

Figure S55. $^{31}P\{^1H\}$ NMR spectrum (162.0 MHz) of the mixture of ruthenium hydrides obtained by reaction of *trans*-[Ru(η^1-OAc)$_2$(SNS)(PPh$_3$)] (**1**) with H_2 (5 bar) and KO^{t}Bu (3 equiv) after heating at 70 °C for 5 h, recorded in toluene-d^8 at 25 °C.

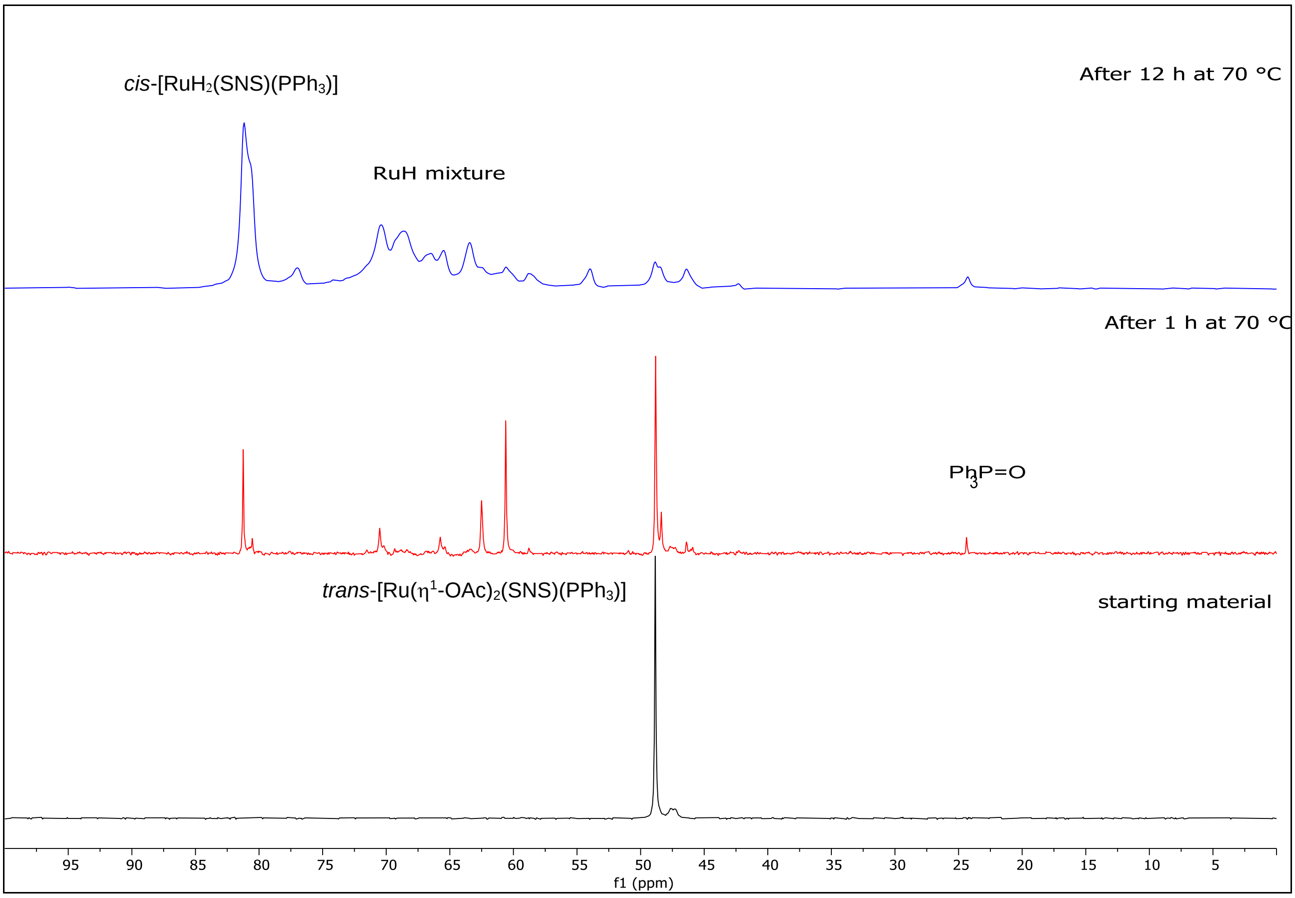

Figure S56. Effect of heating on the formation of the mixture of ruthenium hydrides by reaction of *trans*-[Ru(η^1-OAc)$_2$(SNS)(PPh$_3$)] (**1**) with H_2 (5 bar) and KO^{t}Bu (3 equiv) in the $^{31}P\{^1H\}$ NMR spectra (162.0 MHz), recorded in toluene-d^8 at 25 °C.

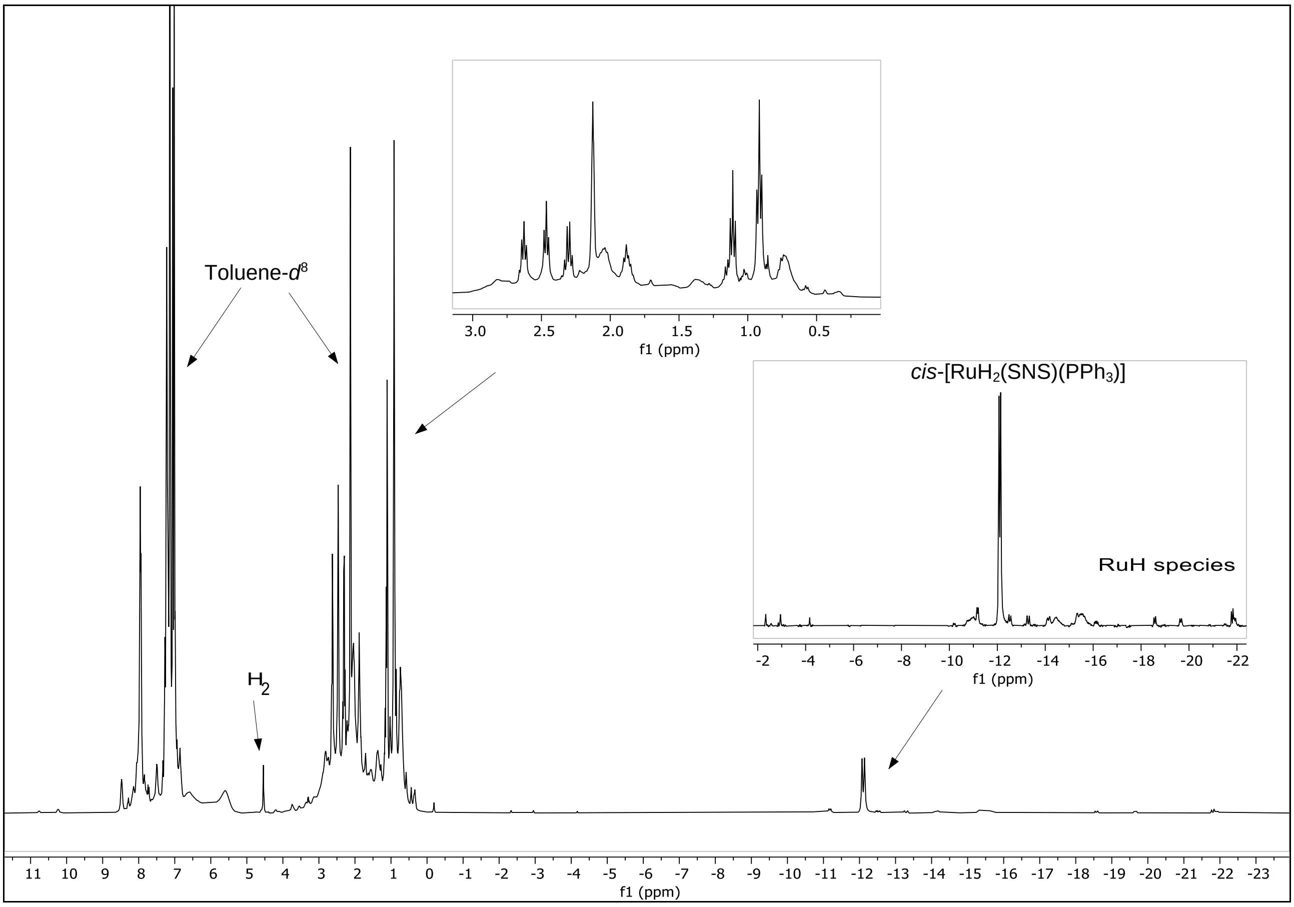

Figure S57. ^{1}H NMR spectrum (400.1 MHz) of the mixture of ruthenium hydrides obtained by reaction of *trans*-[Ru(η^2-OAc)$_2$(SNS)(PPh$_3$)] (**1**) with H_2 (5 bar) and KO^{t}Bu (3 equiv) after heating at 70 °C for 12 h, recorded in toluene-d^8 at 25 °C.

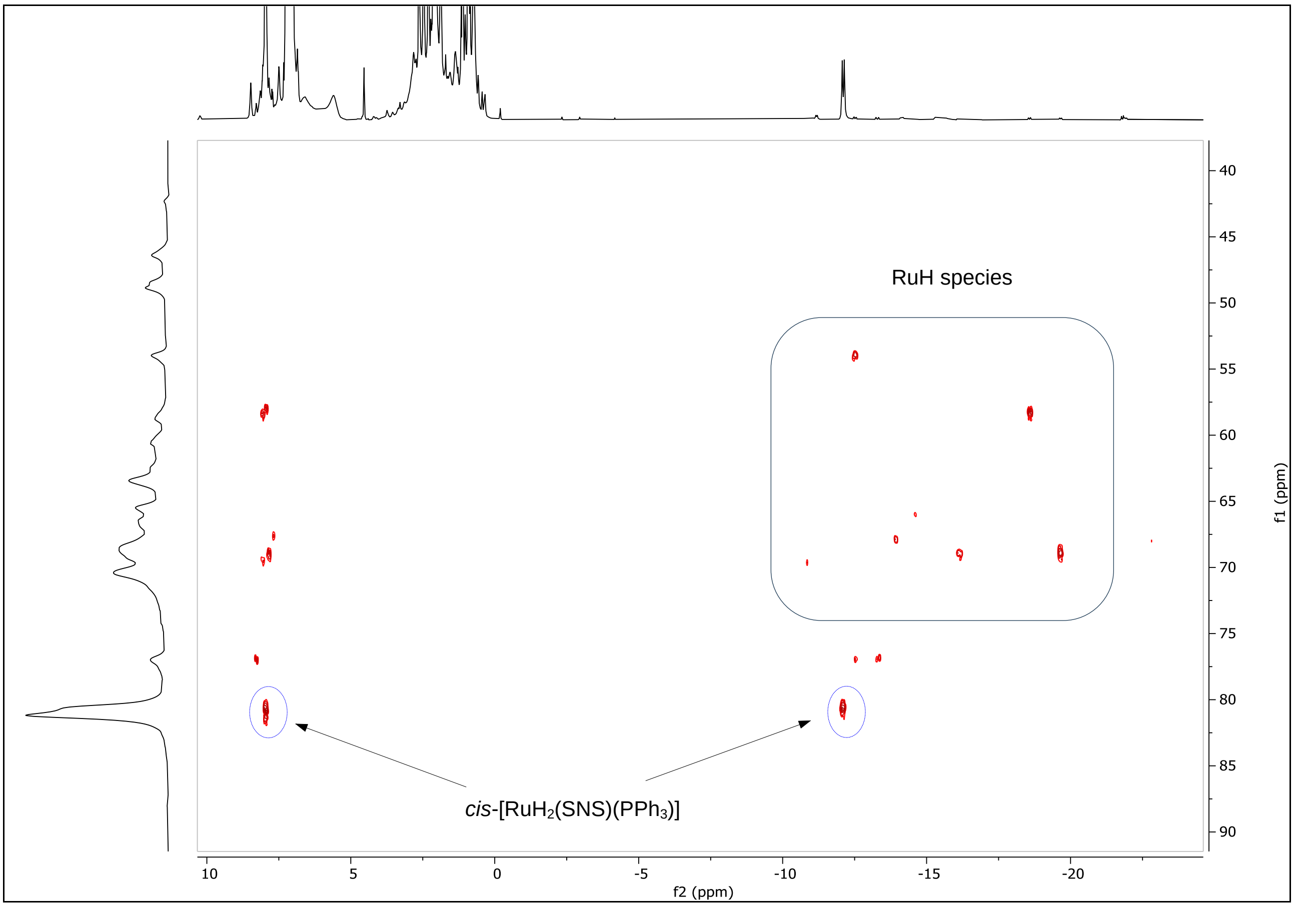

Figure S58. ^{1}H-^{31}P HMBC 2D NMR spectrum of the mixture of ruthenium hydrides obtained by reaction of *trans*-[Ru(η^{1}-OAc)$_2$(SNS)(PPh$_3$)] (**1**) with H_2 (5 bar) and KO^{t}Bu (3 equiv) after heating at 70 °C for 12 h, recorded in toluene-d^8 at 25 °C.

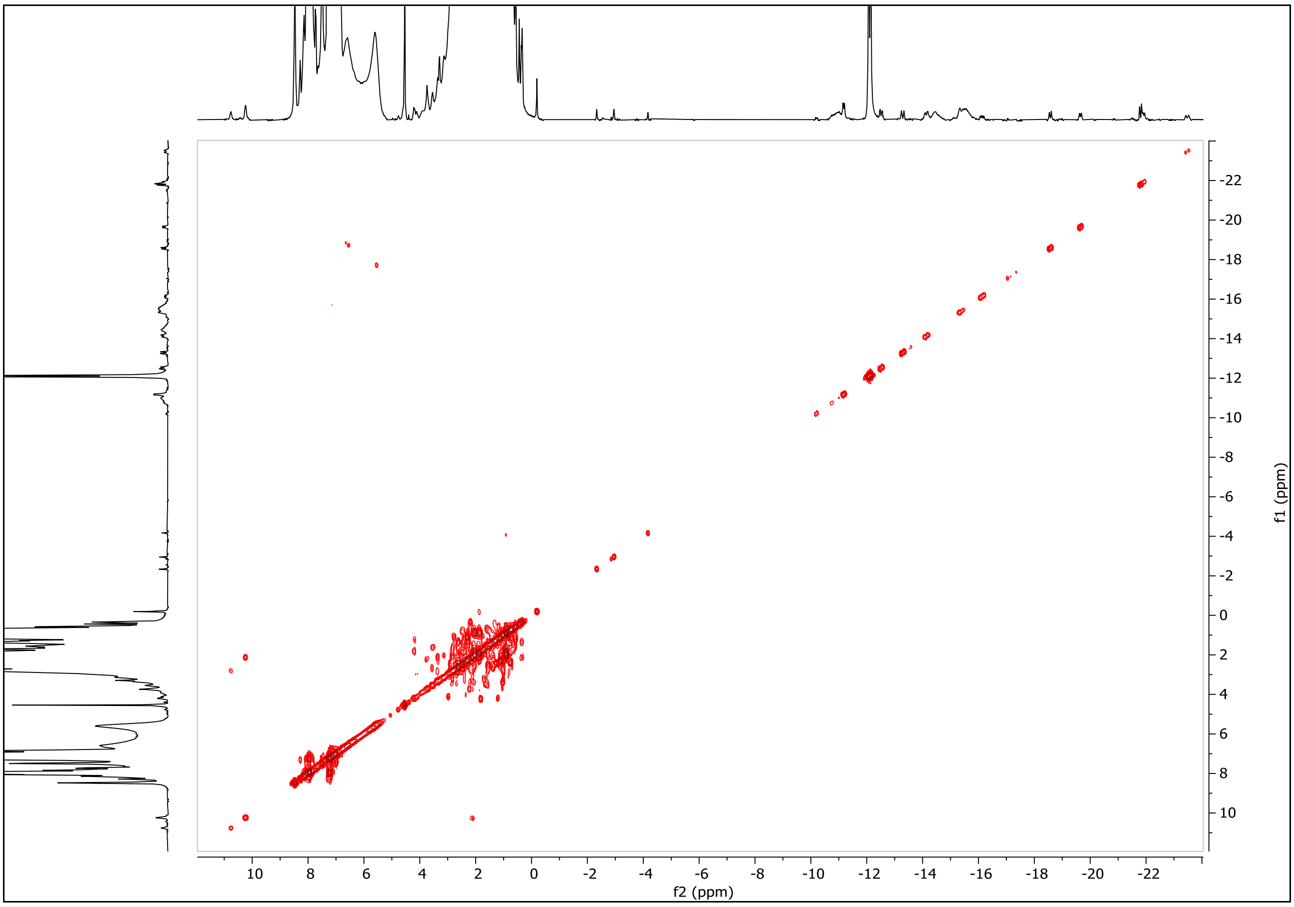

Figure S59. ${}^{1}H$-${}^{1}H$ COSY 2D NMR spectrum of the mixture of ruthenium hydrides obtained by reaction of *trans*-[Ru(η^1-OAc)$_2$(SNS)(PPh$_3$)] (**1**) with H_2 (5 bar) and KO^{t}Bu (3 equiv) after heating at 70 °C for 12 h, recorded in toluene-d^8 at 25 °C.

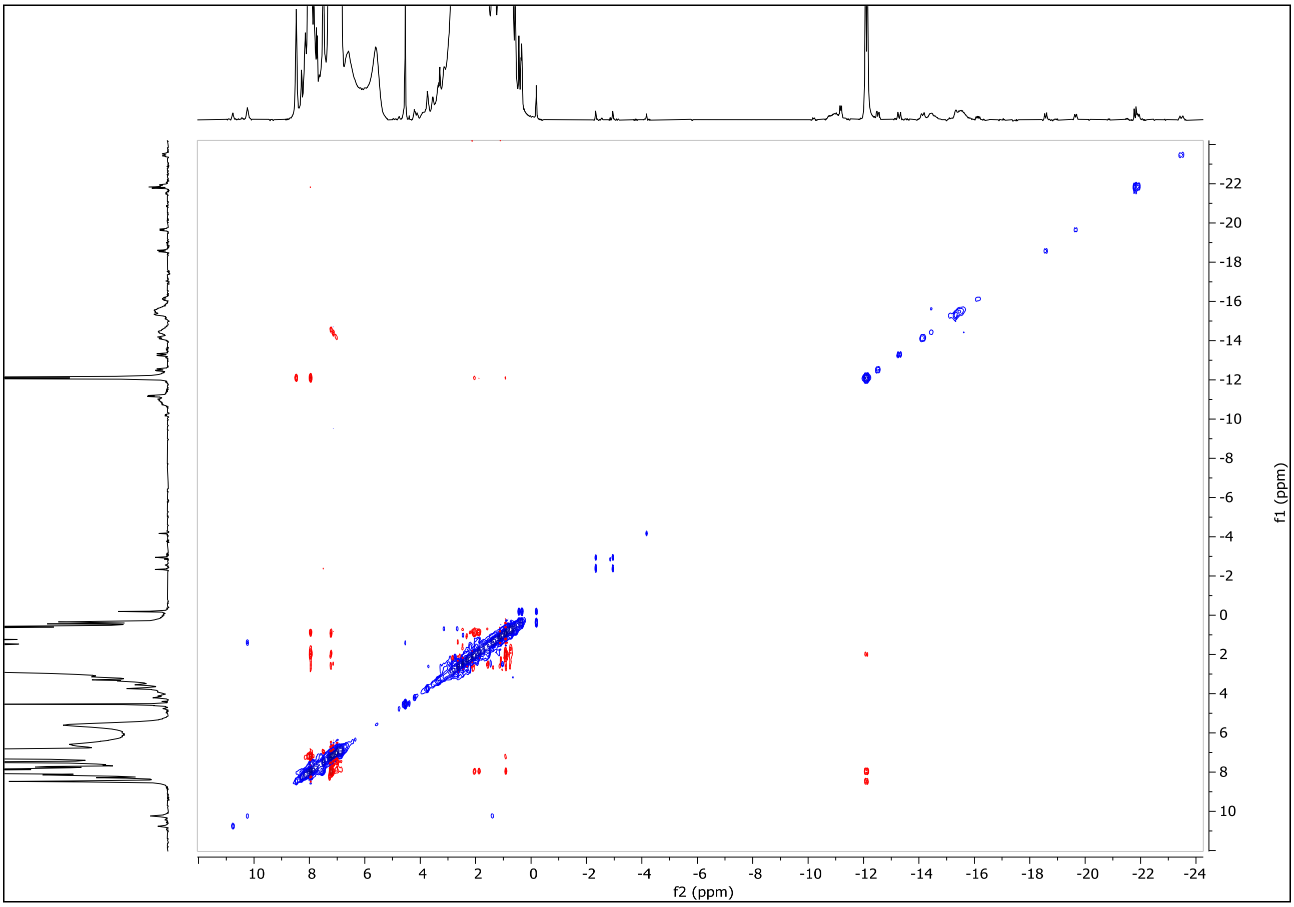

Figure S60. ^{1}H-^{1}H NOESY 2D NMR spectrum of the mixture of ruthenium hydrides obtained by reaction of *trans*-[Ru(η^1-OAc)$_2$(SNS)(PPh$_3$)] (**1**) with H_2 (5 bar) and KO^{t}Bu (3 equiv) after heating at 70 °C for 12 h, recorded in toluene-d^8 at 25 °C.

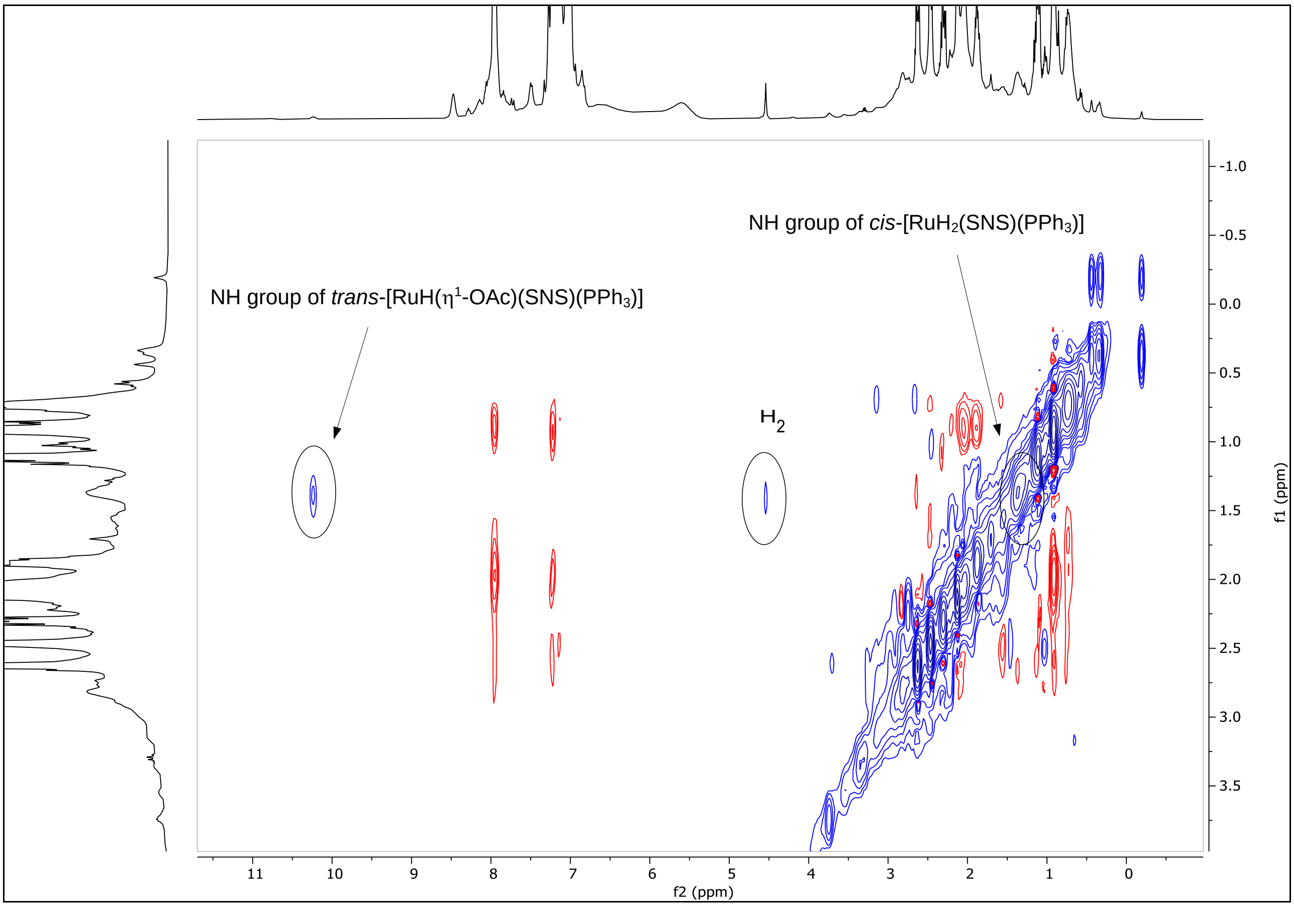

Figure S61. ^{1}H-^{1}H NOESY 2D NMR spectrum of the mixture of ruthenium hydrides obtained by reaction of *trans*-[Ru(η^1-OAc)$_2$(SNS)(PPh$_3$)] (**1**) with H_2 (5 bar) and KO^{t}Bu (3 equiv) after heating at 70 °C for 12 h, recorded in toluene-d^8 at 25 °C (region 0.0-4.0 ppm).

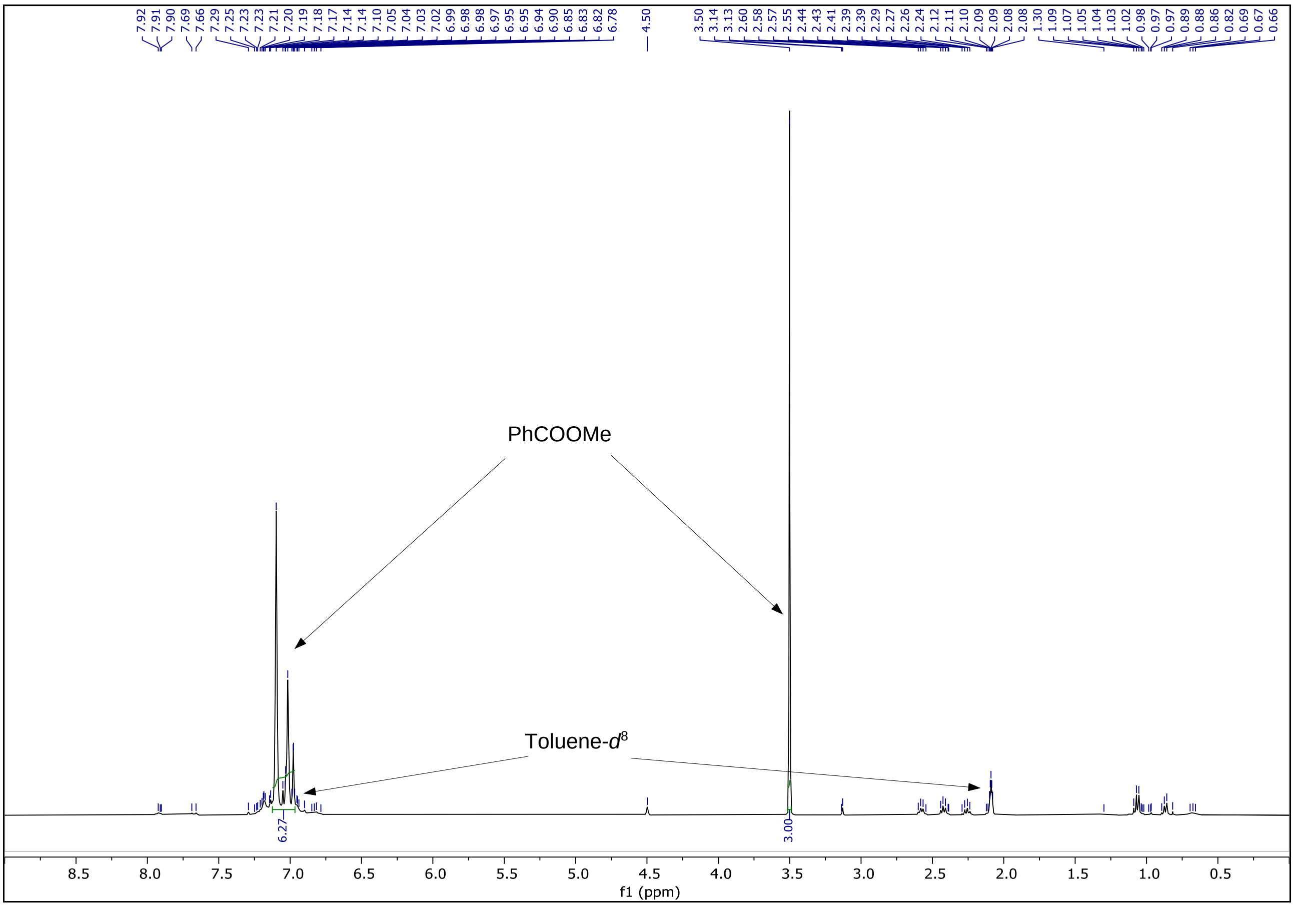

Figure S62. ^1H NMR spectrum (400.1 MHz) after the addition of PhCOOMe (5 equiv) to the mixture of ruthenium hydrides (obtained by reaction of *trans*-[Ru(η^1-OAc)$_2$(SNS)(PPh$_3$)] (**1**) with H_2 and KO^{t}Bu), at RT in H_2 (5 bar) recorded in toluene-d^8 at 25 °C.

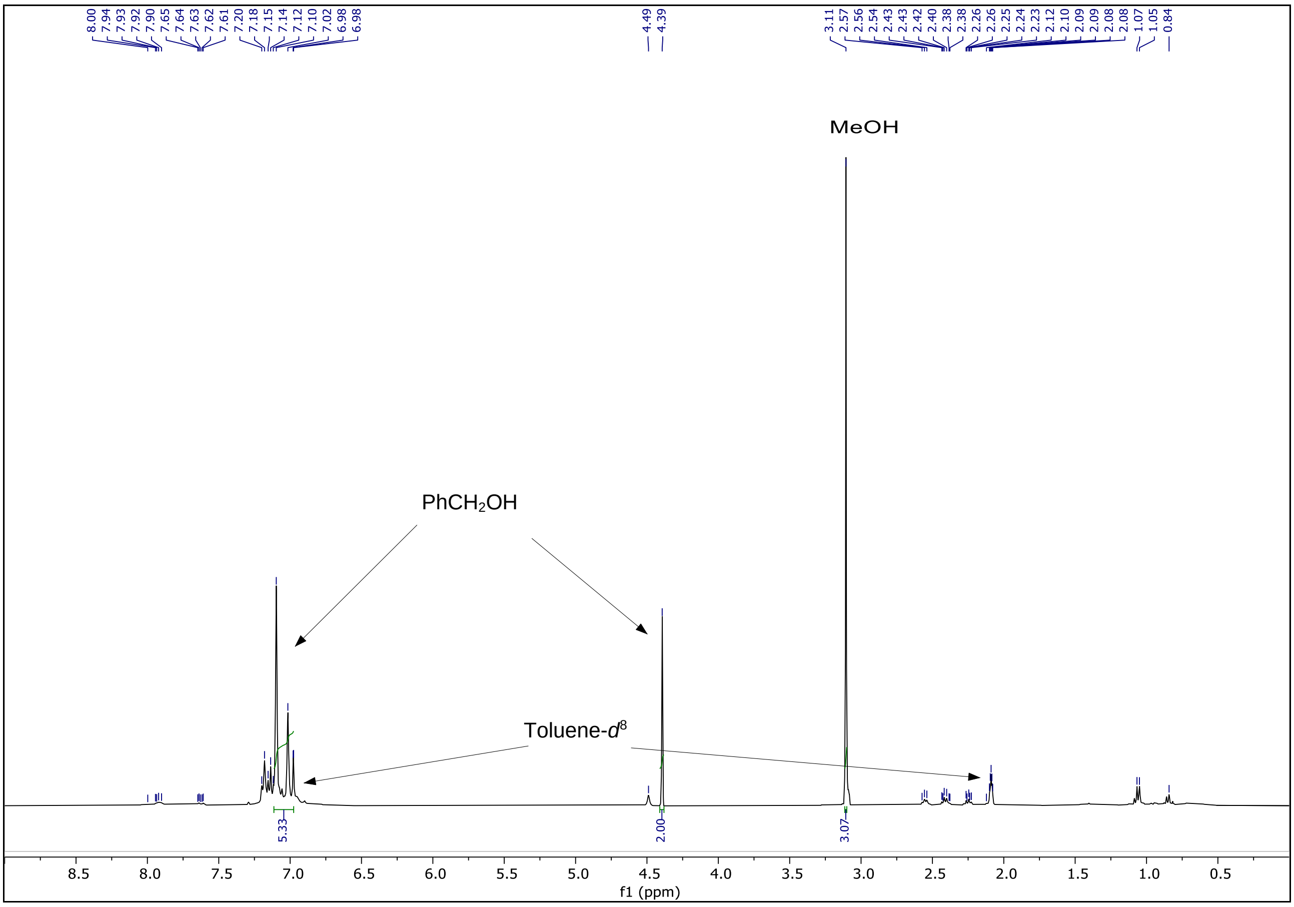

Figure S63. ^{1}H NMR spectrum (400.1 MHz) after the addition of PhCOOMe (5 equiv) to the mixture of ruthenium hydrides (obtained by reaction of *trans*-[Ru(η^1-OAc)$_2$(SNS)(PPh$_3$)] (**1**) with H_2 and KO^{t}Bu), after heating at 70 °C for 5 h recorded in toluene-d^8 at 25 °C.

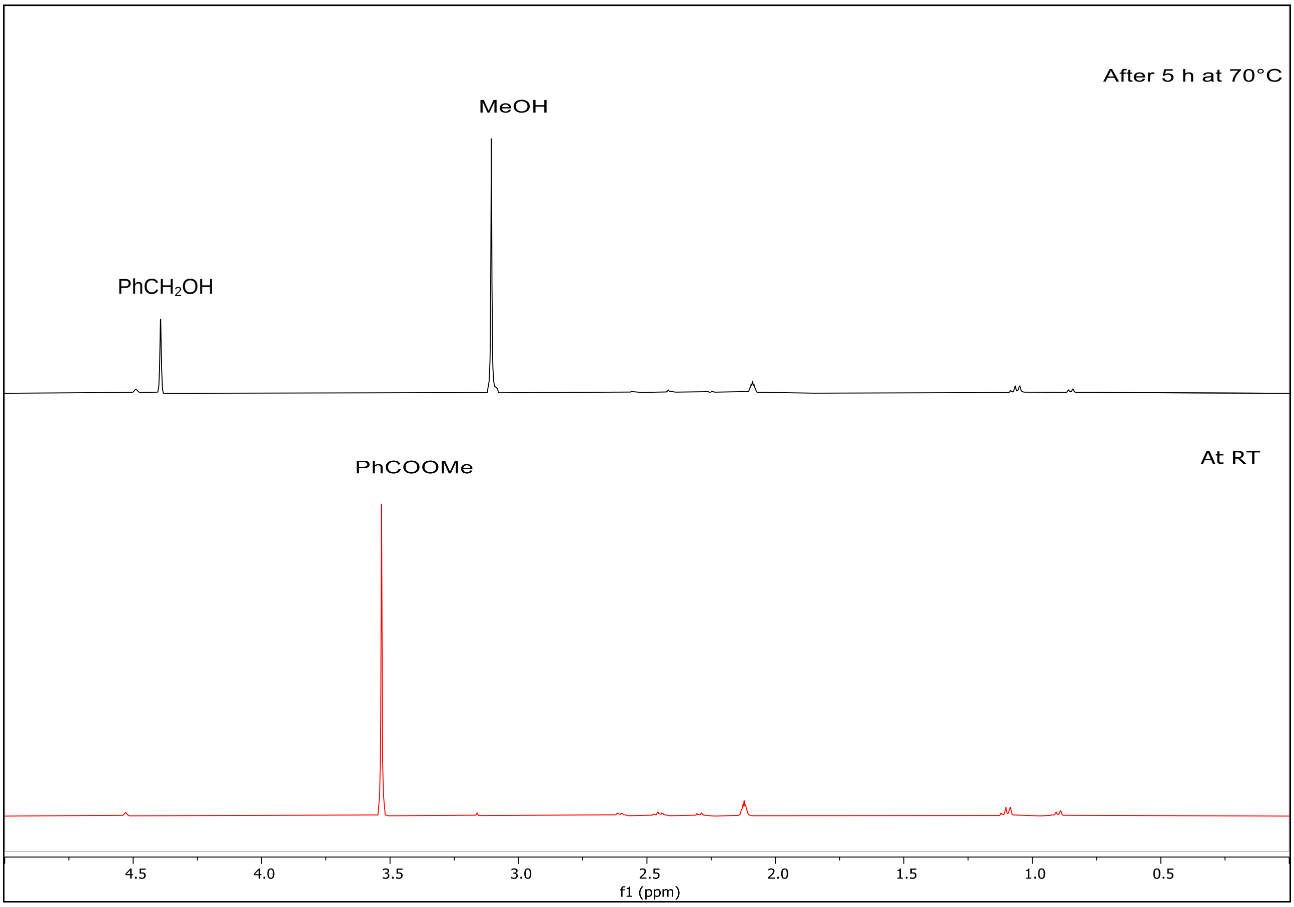

Figure S64. Effect of heating (70 °C for 5 h) on the ^{1}H NMR spectrum (400.1 MHz) of the mixture of ruthenium hydrides (obtained by reaction of *trans*-[Ru(η^{1}-OAc)$_2$(SNS)(PPh$_3$)] (**1**) with H_2 and KO^{t}Bu) after the addition of PhCOOMe (5 equiv) in H_2 (5 bar) recorded in toluene-d^{8} at 25 °C.

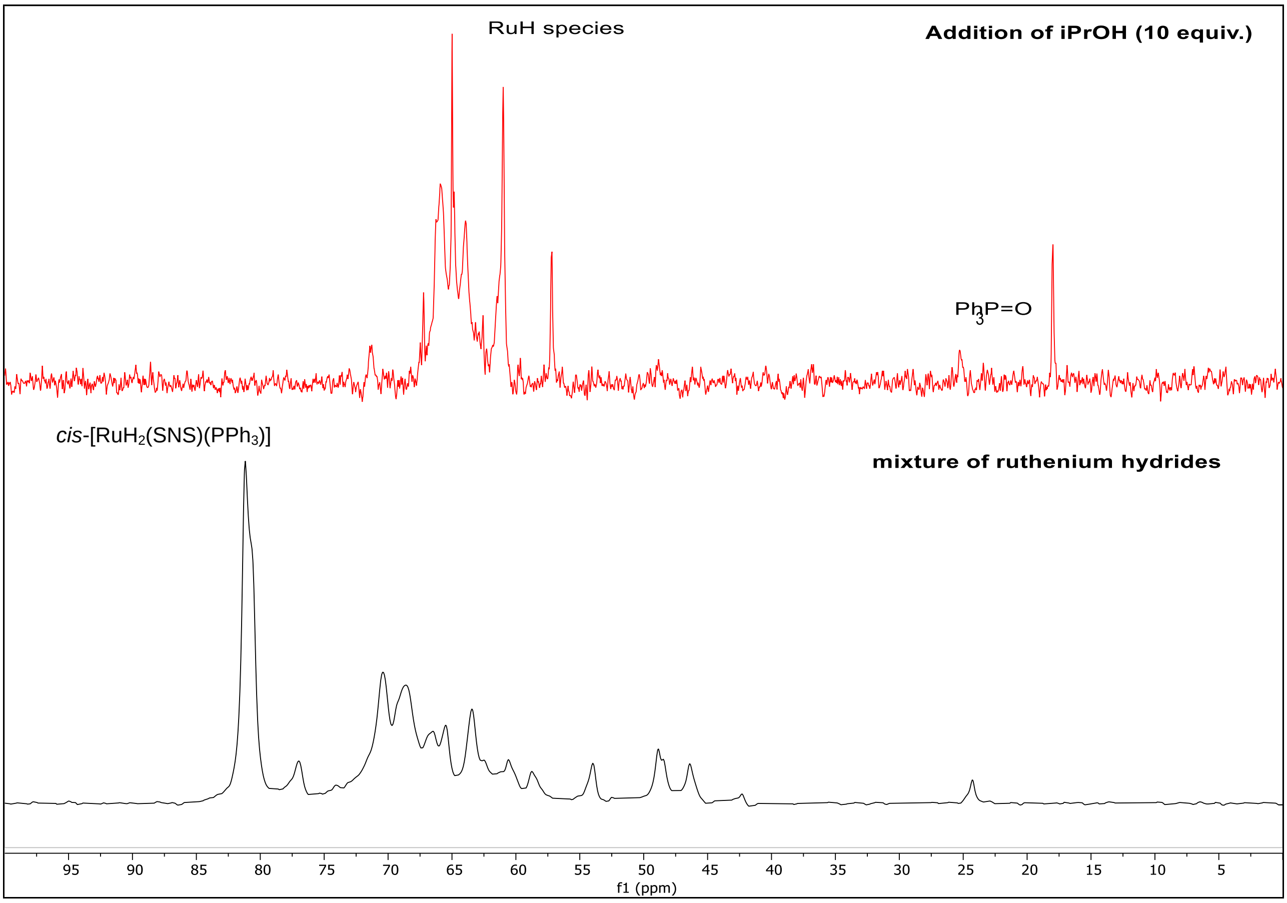

Figure S65. Effect of addition of 2-propanol (10 equiv) to the mixture of ruthenium hydrides (obtained by reaction of *trans*-[Ru(η^1-OAc)$_2$(SNS)(PPh$_3$)] (**1**) with H_2 and KO^{t}Bu) on the ^{31}P{^{1}H} NMR spectra (162.0 MHz), recorded in toluene-d^8 at 25 °C.

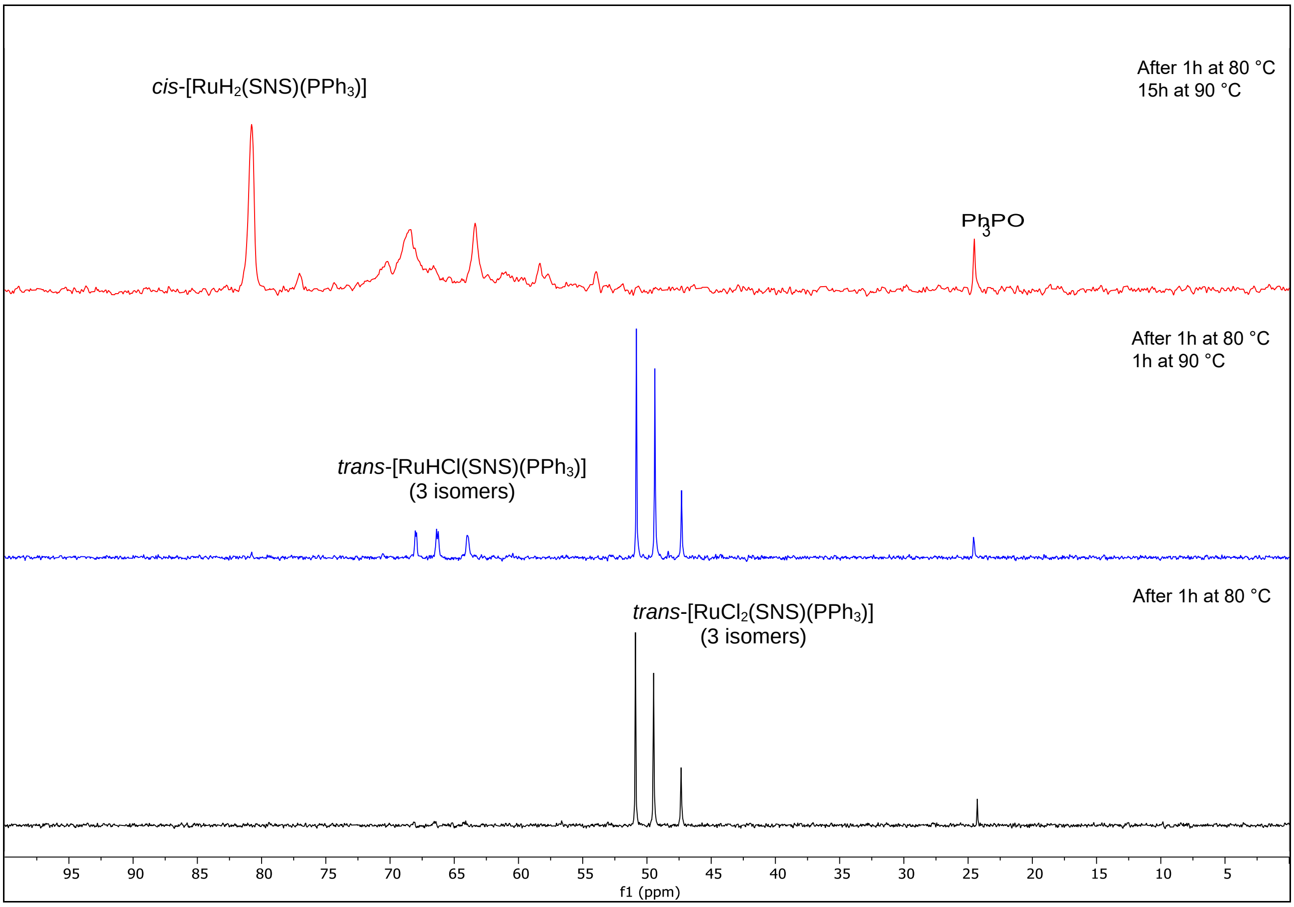

Figure S66. Effect of heating in the formation of the mixture of ruthenium hydrides by reaction of *trans*-[RuCl$_2$(SNS)(PPh$_3$)] with H_2 (5 bar) and KO^{t}Bu (3 equiv) in the ^{31}P{^{1}H} NMR spectra (162.0 MHz), recorded in toluene-d^8 at 25 °C.

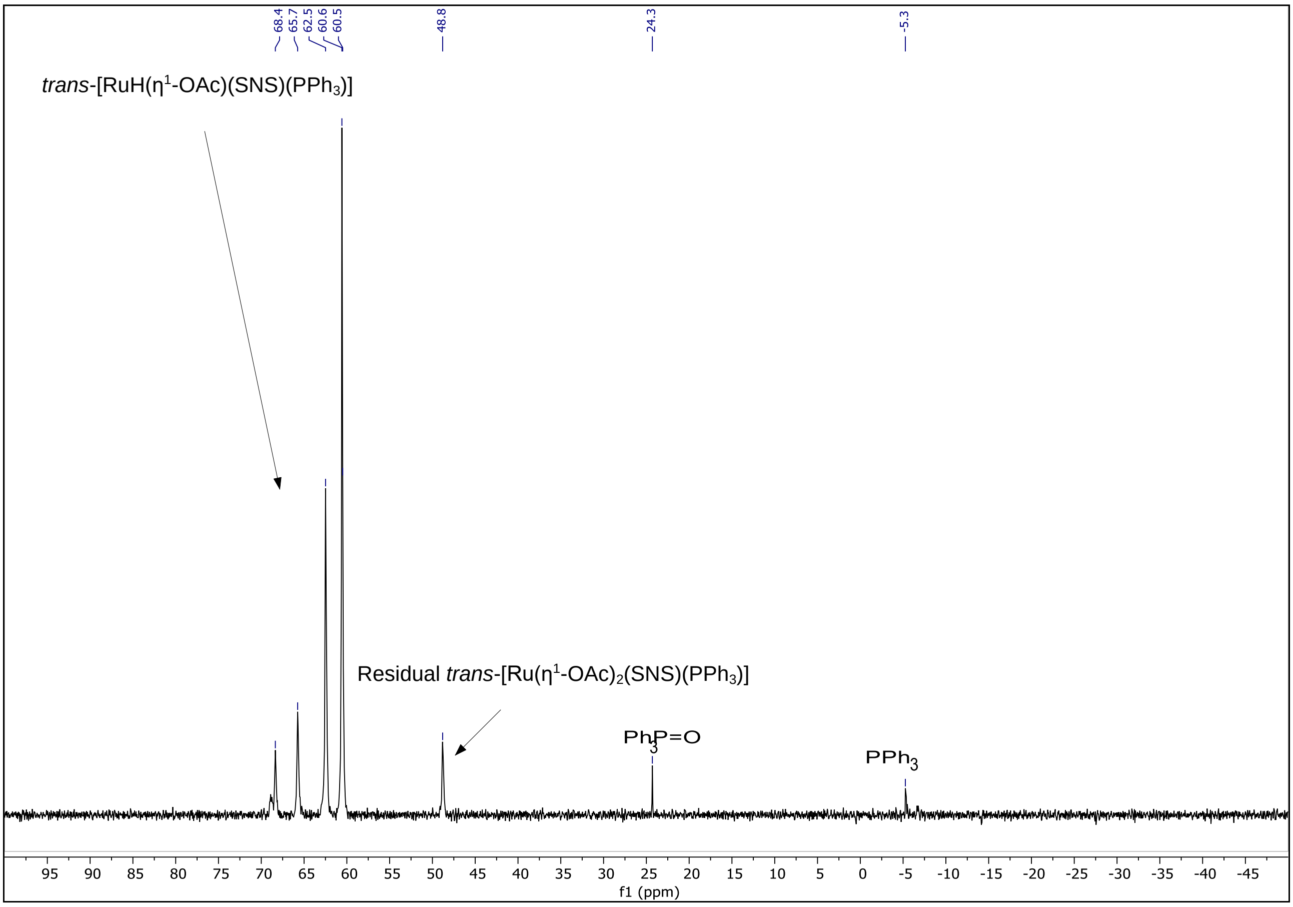

Figure S67. $^{31}P\{^{1}H\}$ NMR spectrum (162.0 MHz) of the mixture of ruthenium monohydrides obtained by reaction of *trans*-[Ru(η^1-OAc)$_2$(SNS)(PPh$_3$)] (**1**) with H_2 (5 bar) and DBU (3 equiv) after heating at 100 °C for 15 days, recorded in toluene-d^8 at 25 °C.

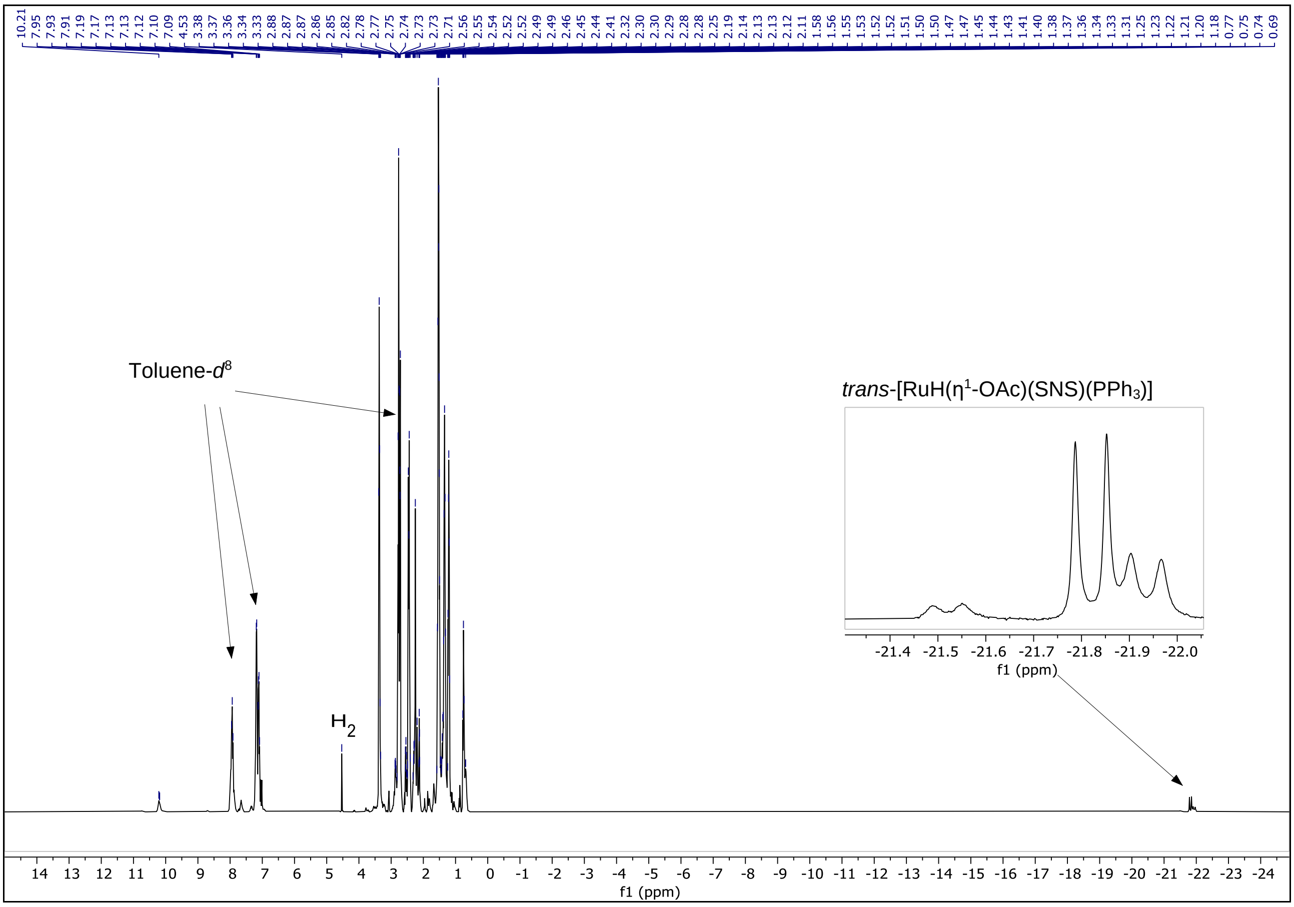

Figure S68. ^{1}H NMR spectrum (400.1 MHz) of the mixture of ruthenium monohydrides obtained by reaction of *trans*-[Ru(η^1-OAc)$_2$(SNS)(PPh$_3$)] (**1**) with H_2 (5 bar) and DBU (3 equiv) after heating at 100 °C for 15 days, recorded in toluene-d^8 at 25 °C.

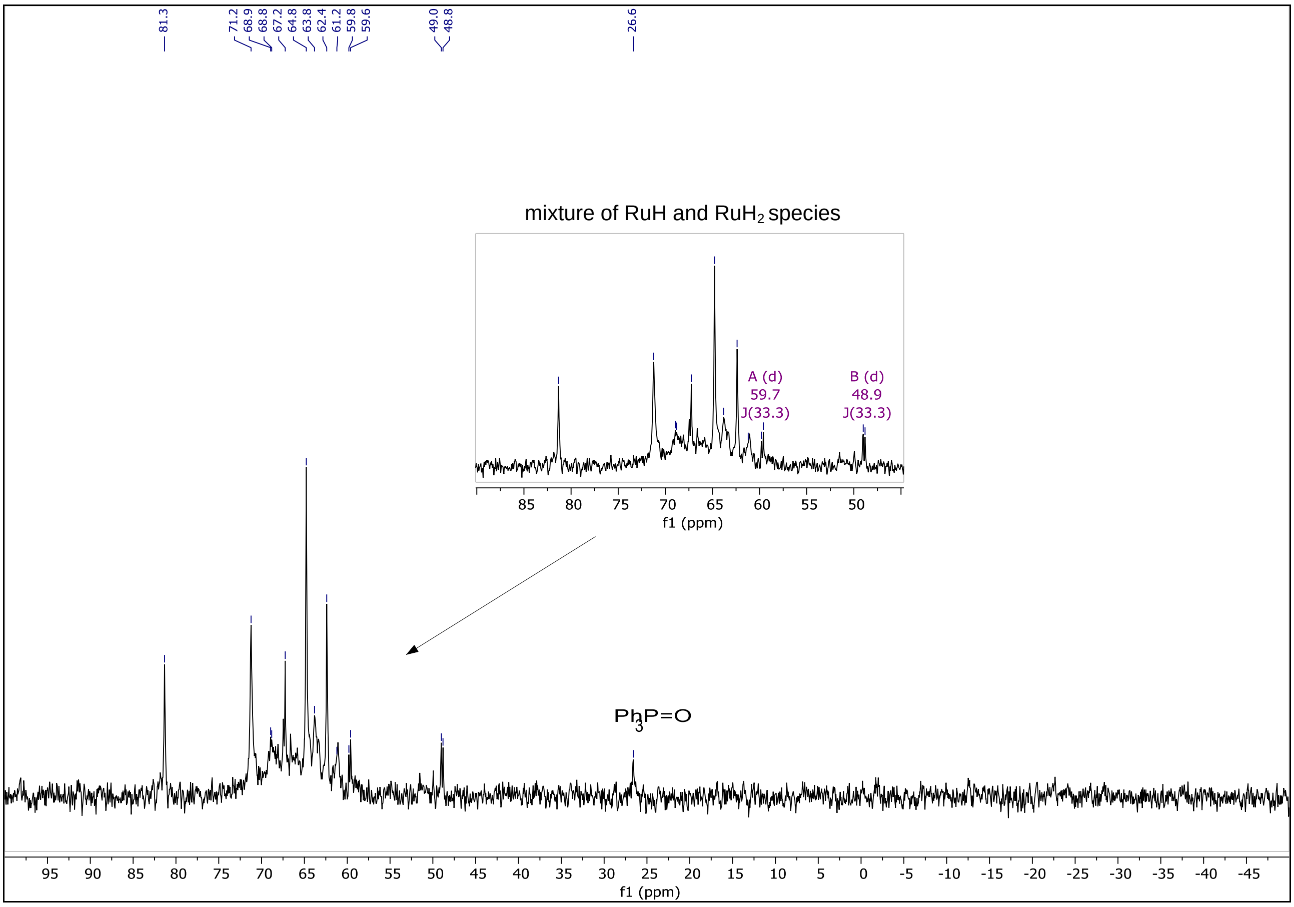

Figure S69. $^{31}P\{^{1}H\}$ NMR spectrum (162.0 MHz) of the mixture of ruthenium hydrides obtained by reaction of *trans*-[Ru(η^1-OAc)$_2$(SNS)(PPh$_3$)] (**1**) with H_2 (5 bar) and DIBAL-H (2 equiv or 1 equiv as dimer) after heating at 70 °C for 4 h, recorded in toluene-d^8 at 25 °C.

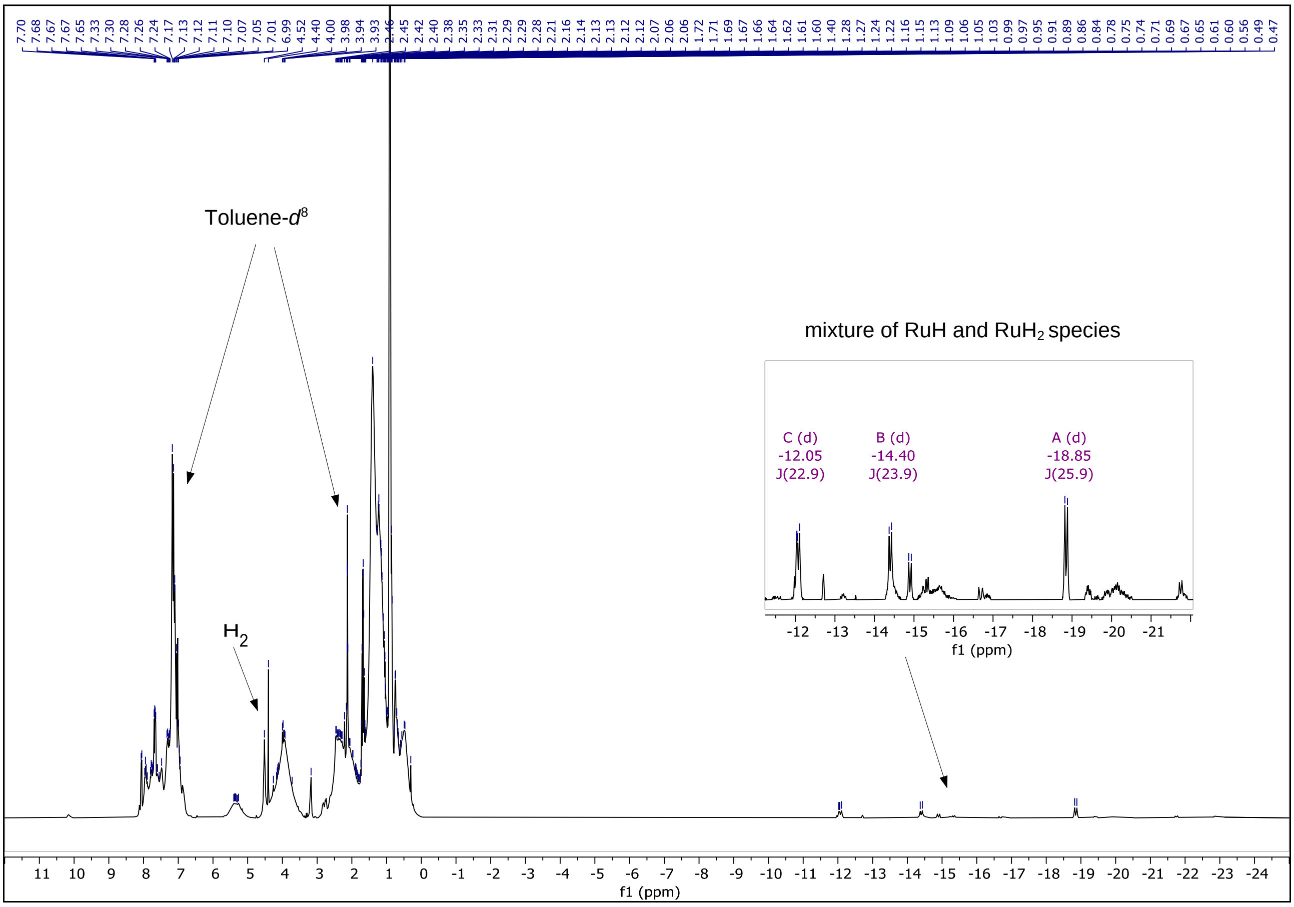

Figure S70. ^{1}HNMR spectrum (400.1 MHz) of the mixture of ruthenium hydrides obtained by reaction of *trans*-[Ru(η^1-OAc)$_2$(SNS)(PPh$_3$)] (**1**) with H_2 (5 bar) and DIBAL-H (2 equiv or 1 equiv as dimer) after heating at 70 °C for 4 h, recorded in toluene-*d*^{8} at 25 °C.

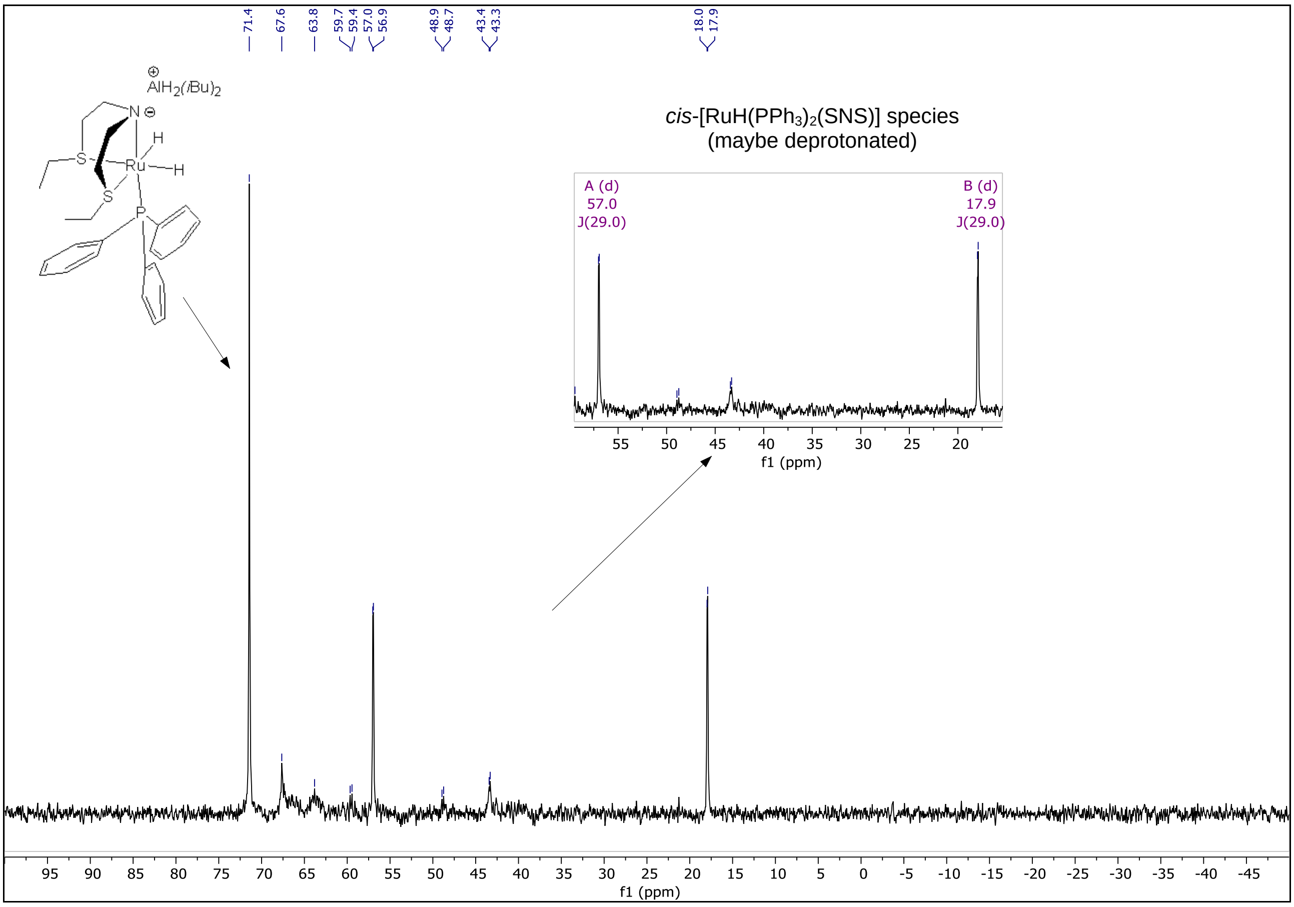

Figure S71. $^{31}P\{^1H\}$ NMR spectrum (162.0 MHz) of the mixture of ruthenium hydrides obtained by reaction of *trans*-[Ru(η^1-OAc)$_2$(SNS)(PPh$_3$)] (**1**) with H_2 (5 bar) and DIBAL-H (6 equiv or 3 equiv as dimer) after heating at 70 °C for 1 h, recorded in toluene-*d*^{8} at 25 °C.

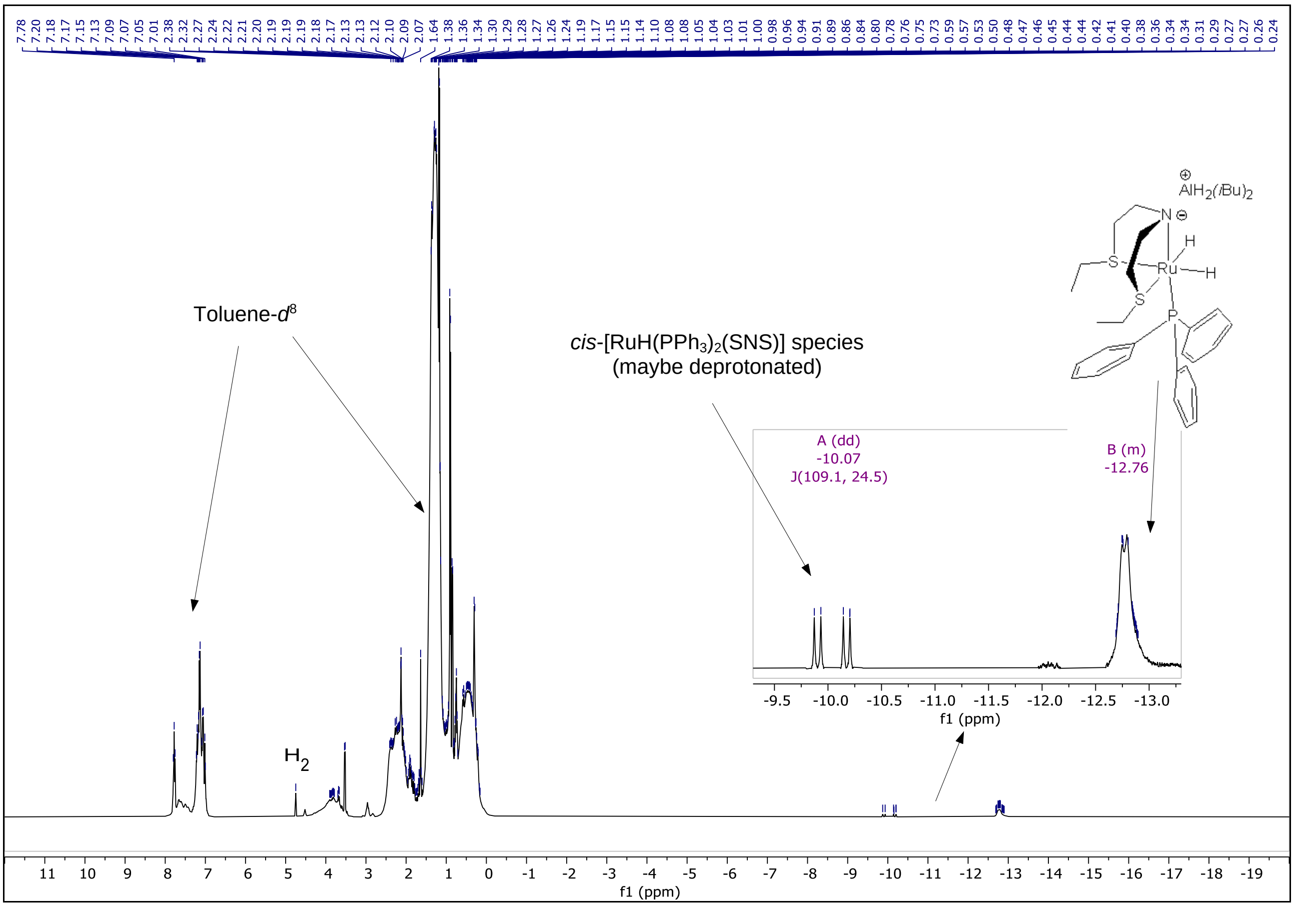

Figure S72. ^{1}HNMR spectrum (400.1 MHz) of the mixture of ruthenium hydrides obtained by reaction of *trans*-[Ru(η^1-OAc)$_2$(SNS)(PPh$_3$)] (**1**) with H_2 (5 bar) and DIBAL-H (6 equiv or 3 equiv as dimer) after heating at 70 °C for 1 h, recorded in toluene-*d*^{8} at 25 °C.

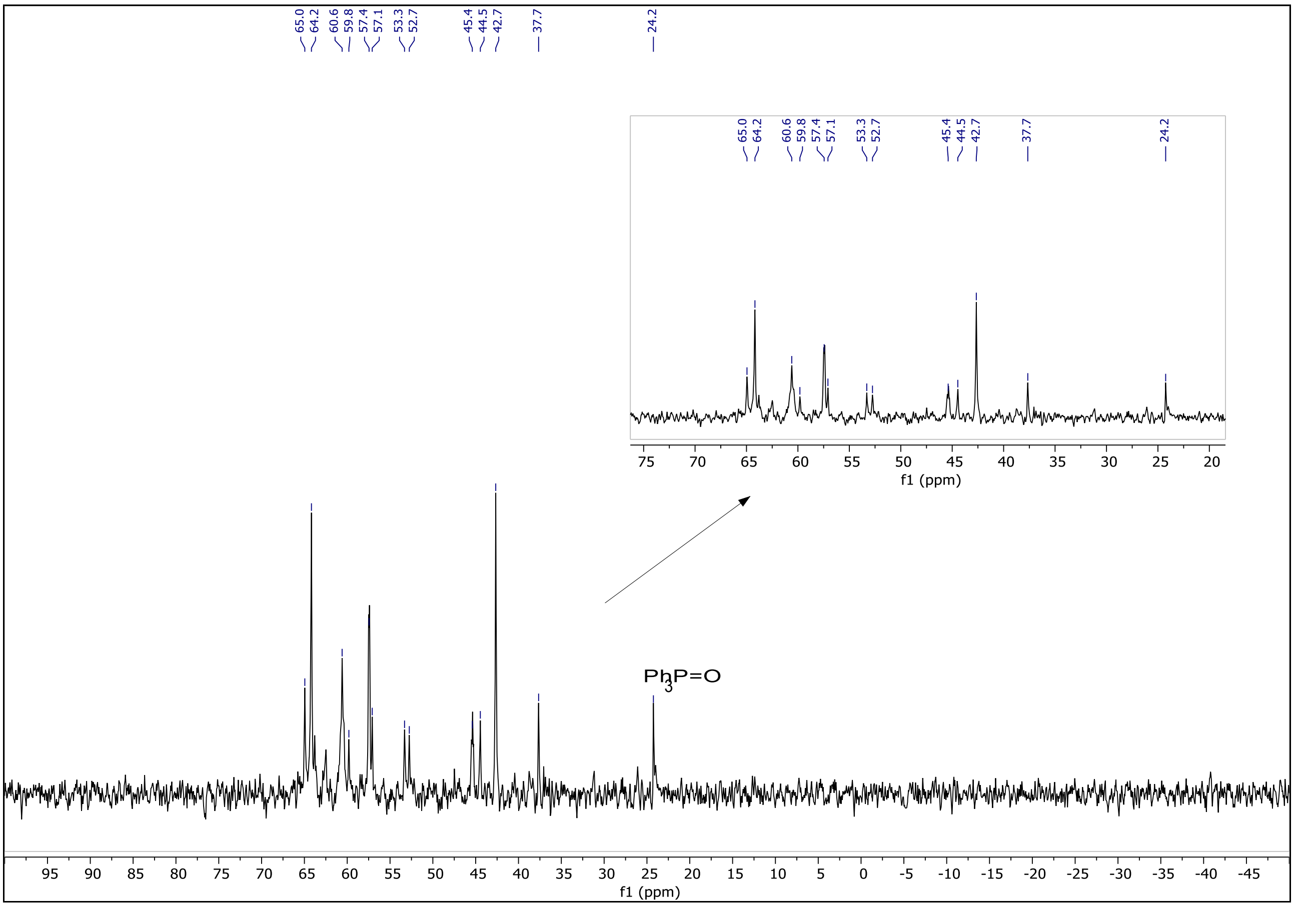

Figure S73. $^{31}P\{^{1}H\}$ NMR spectrum (162.0 MHz) of the mixture of ruthenium hydrides obtained by reaction of [Ru(η^1-OAc)(CO)(SNS)(PPh_3)]OAc (**5**) with H_2 (5 bar) and KO*ᵗ*Bu (3 equiv) after heating at 70 °C for 2 h, recorded in toluene-d^8 at 25 °C.

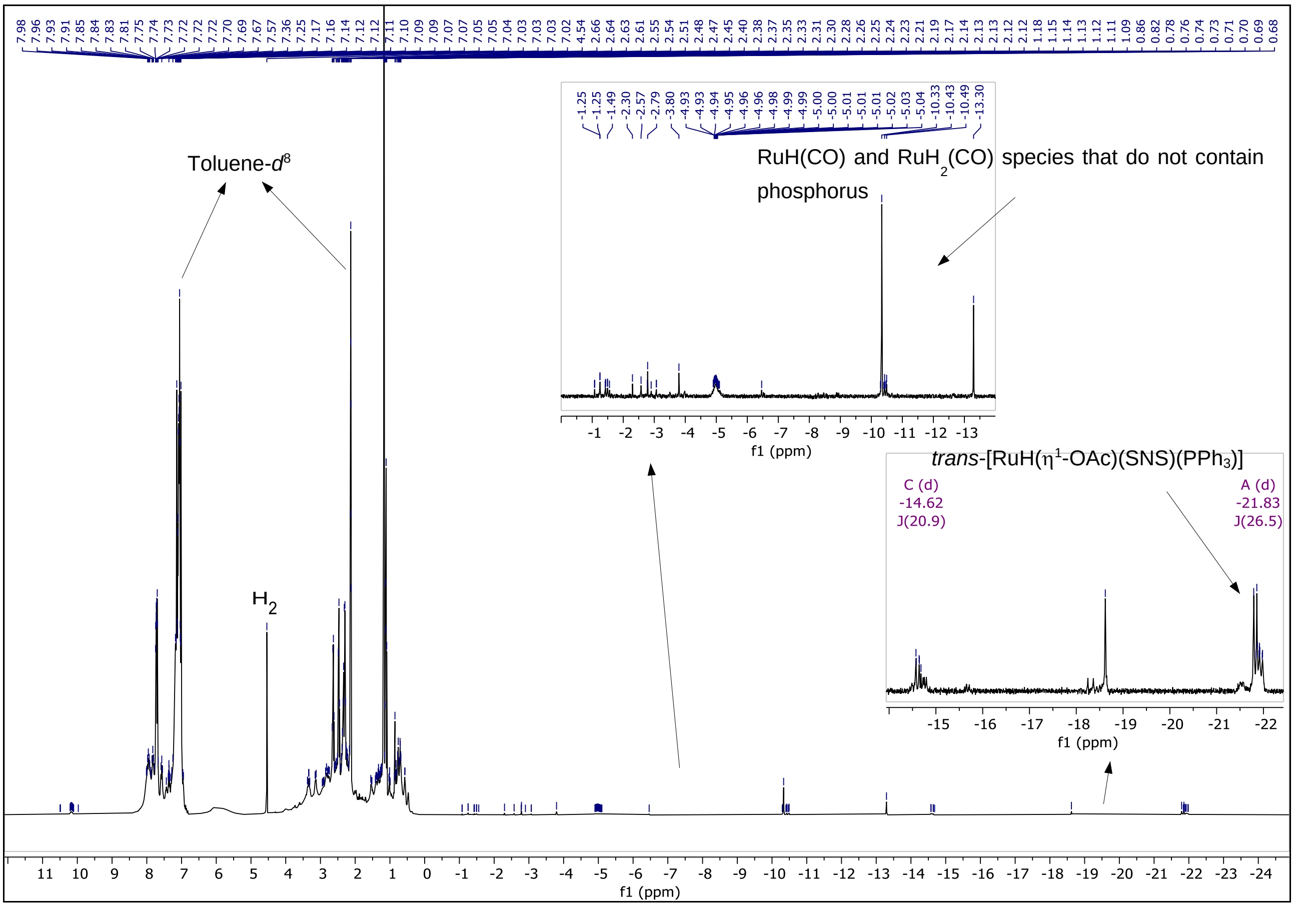

Figure S74. ^{1}HNMR spectrum (400.1 MHz) of the mixture of ruthenium hydrides obtained by reaction of [Ru(η^1-OAc)(CO)(SNS)(PPh_3)]OAc (**5**) with H_2 (5 bar) and KO^{t}Bu (3 equiv) after heating at 70 °C for 2 h, recorded in toluene-d^8 at 25 °C.

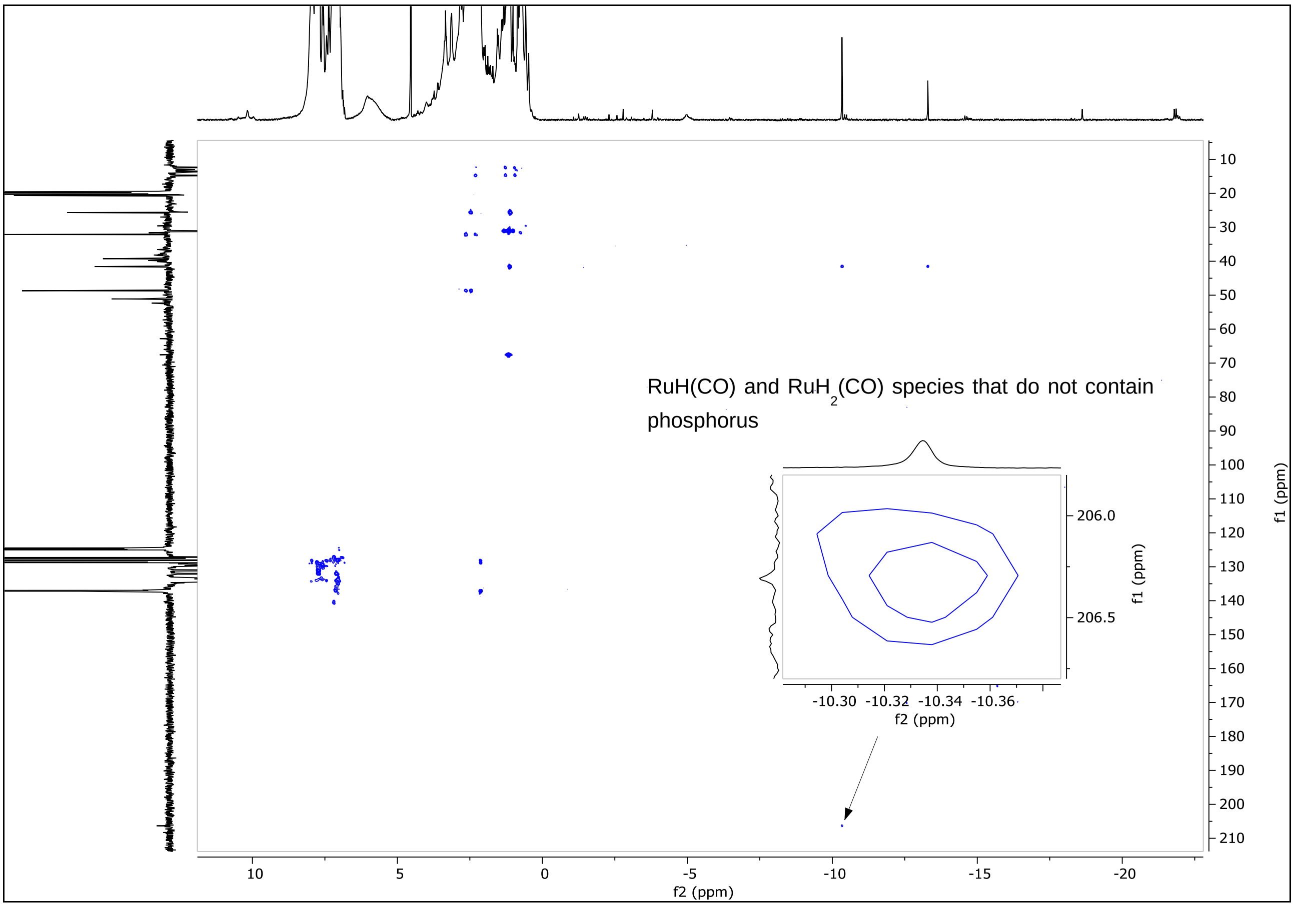

Figure S75. ^{1}H-^{13}C HMBC 2D NMR spectrum of the mixture of ruthenium hydrides obtained by reaction of [Ru(η^1-OAc)(CO)(SNS)(PPh_3)]OAc (**5**) with H_2 (5 bar) and KO^{t}Bu (3 equiv) after heating at 70 °C for 2 h, recorded in toluene-d^8 at 25 °C.

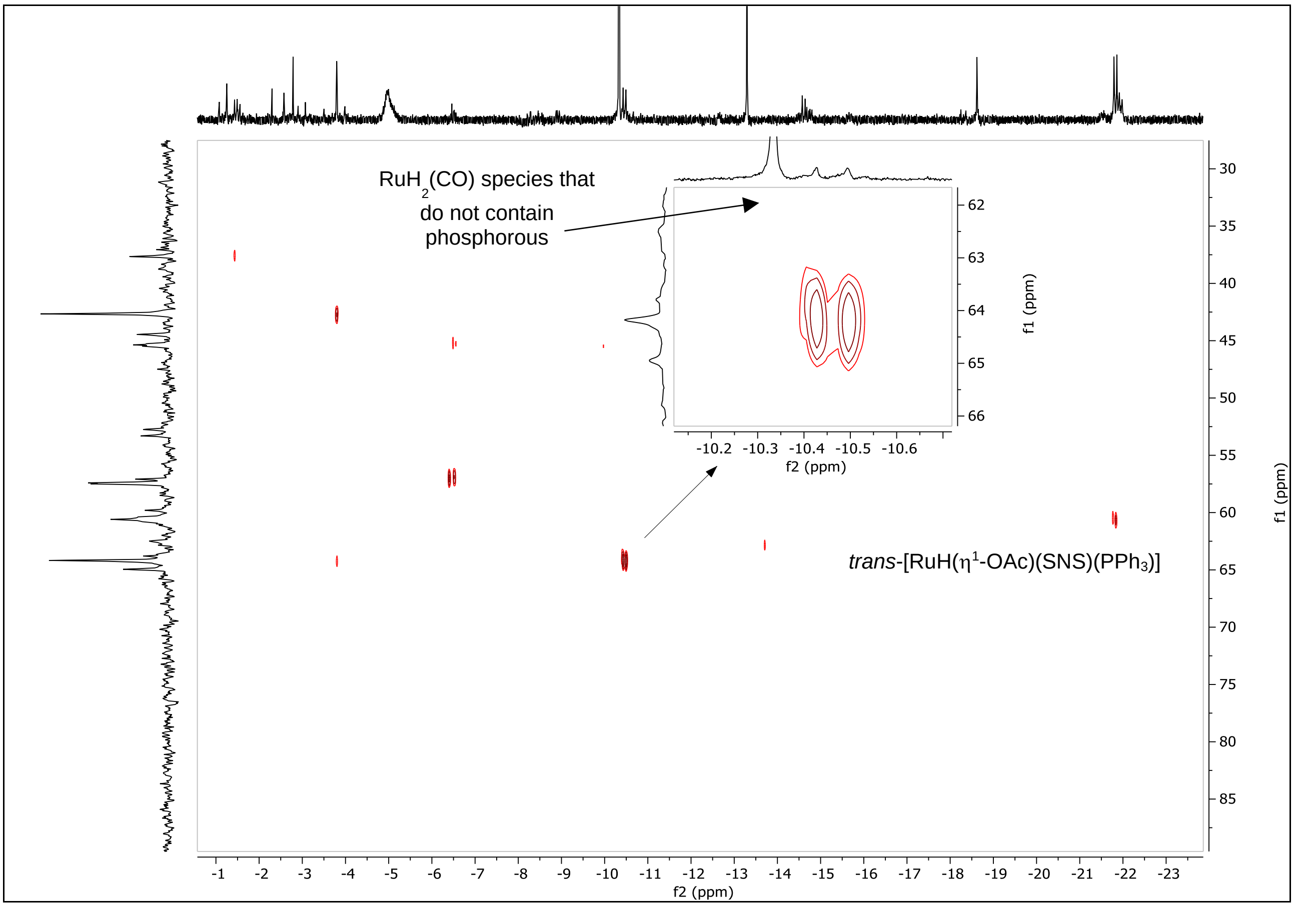

Figure S76. ^{1}H-^{31}P HMBC 2D NMR spectrum of the mixture of ruthenium hydrides obtained by reaction of [Ru(η^1-OAc)(CO)(SNS)(PPh_3)]OAc (**5**) with H_2 (5 bar) and KO^{t}Bu (3 equiv) after heating at 70 °C for 2 h, recorded in toluene-d^8 at 25 °C.

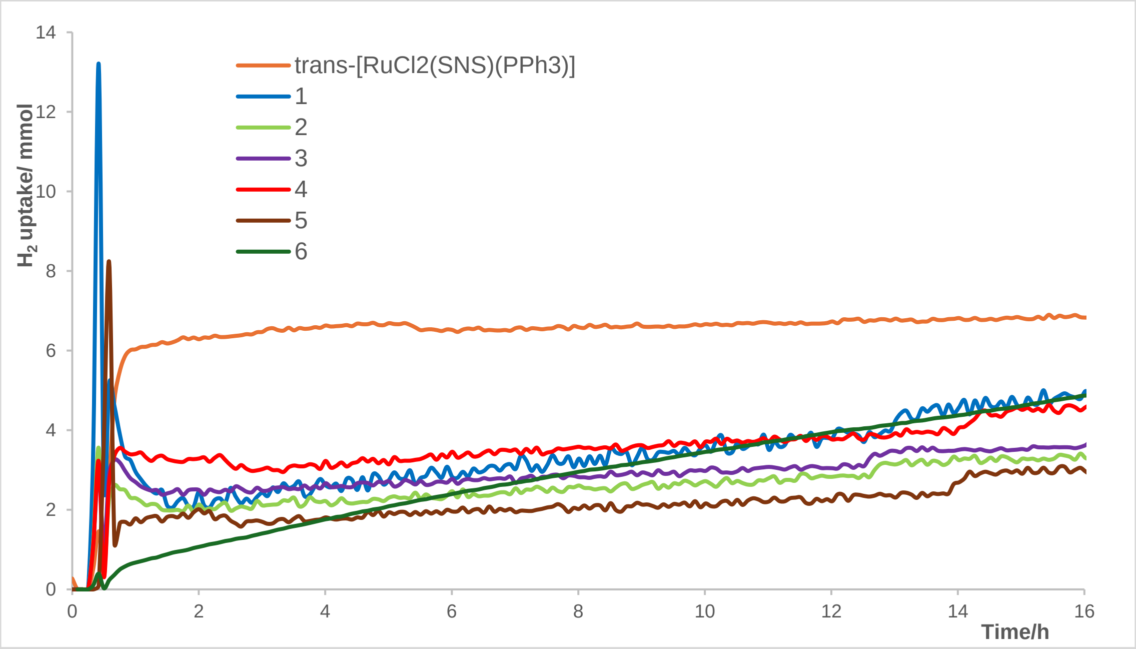

Figure S77. Hydrogen uptake curve of complexes **1-6** and the *trans*-[RuCl$_2$(SNS)(PPh$_3$)] derivative for the solvent-free reduction of methyl decanoate **a** at 90 °C, 27.5 bar of H_2, 50 mol% NaOMe (S/C 10000).

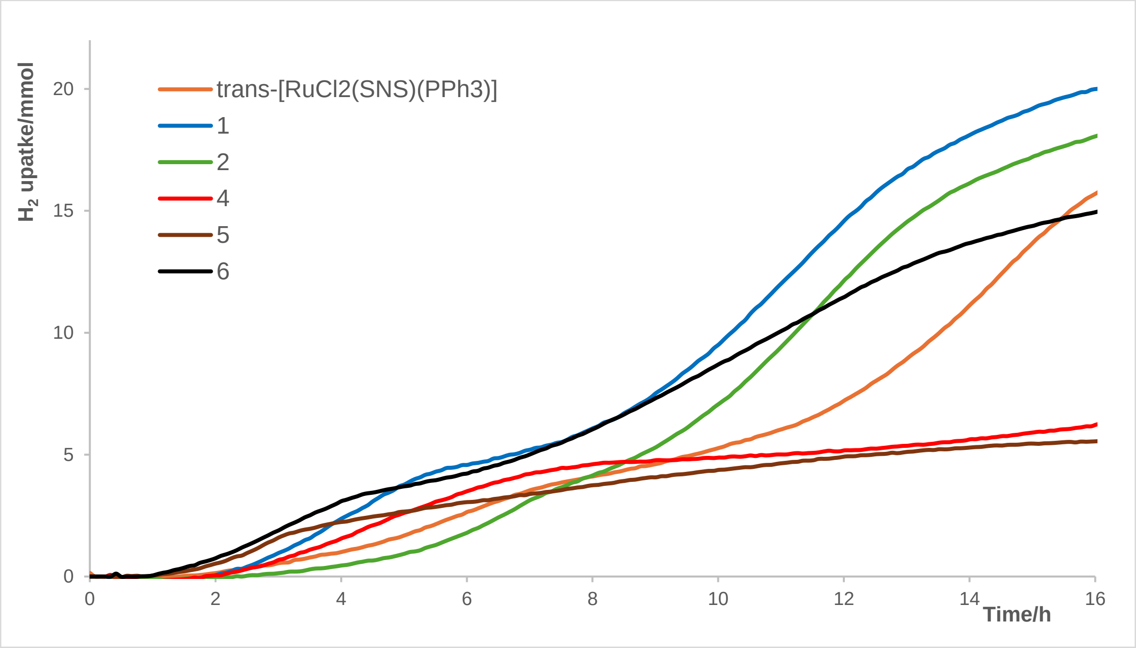

Figure S78. Hydrogen uptake curve of complexes **1**, **2**, **4-6** and the *trans*-[RuCl$_2$(SNS)(PPh$_3$)] derivative for the solvent-free reduction of methyl decanoate **a** at 40 °C, 27.5 bar of H_2, 50 mol% NaOMe (S/C 50000).

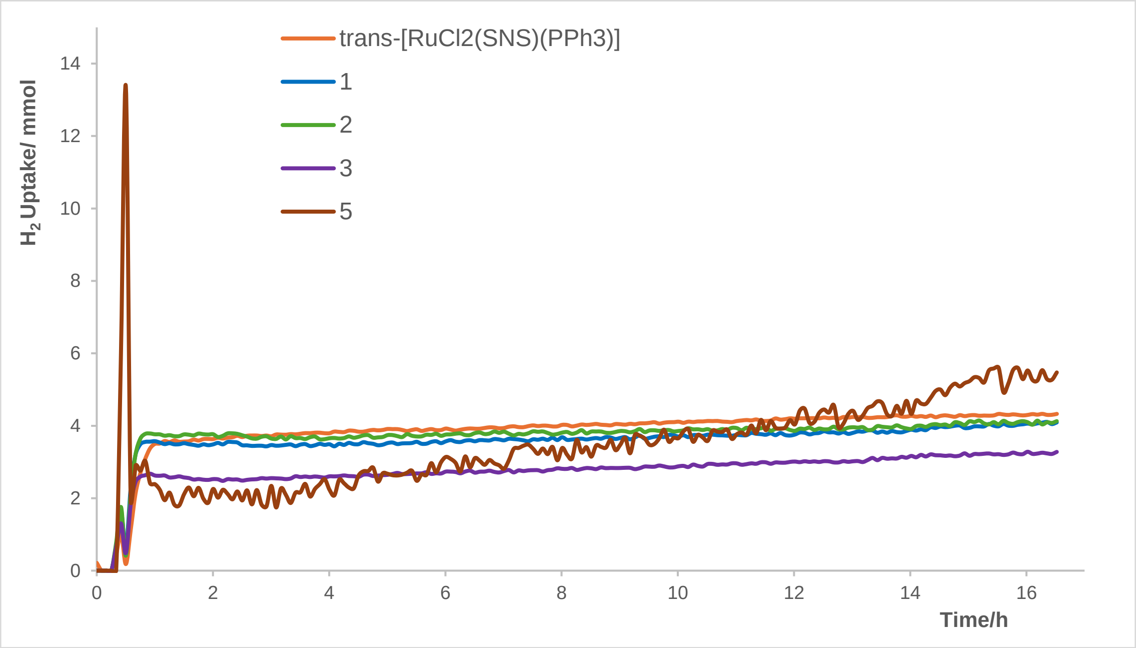

Figure S79. Hydrogen uptake curve of complexes **1-3**, **5** and the *trans*-[RuCl₂(SNS)(PPh₃)] derivative for the solvent-free reduction of methyl decanoate **a** at 90 °C, 27.5 bar of H_2, 50 mol% NaOMe (S/C 50000).

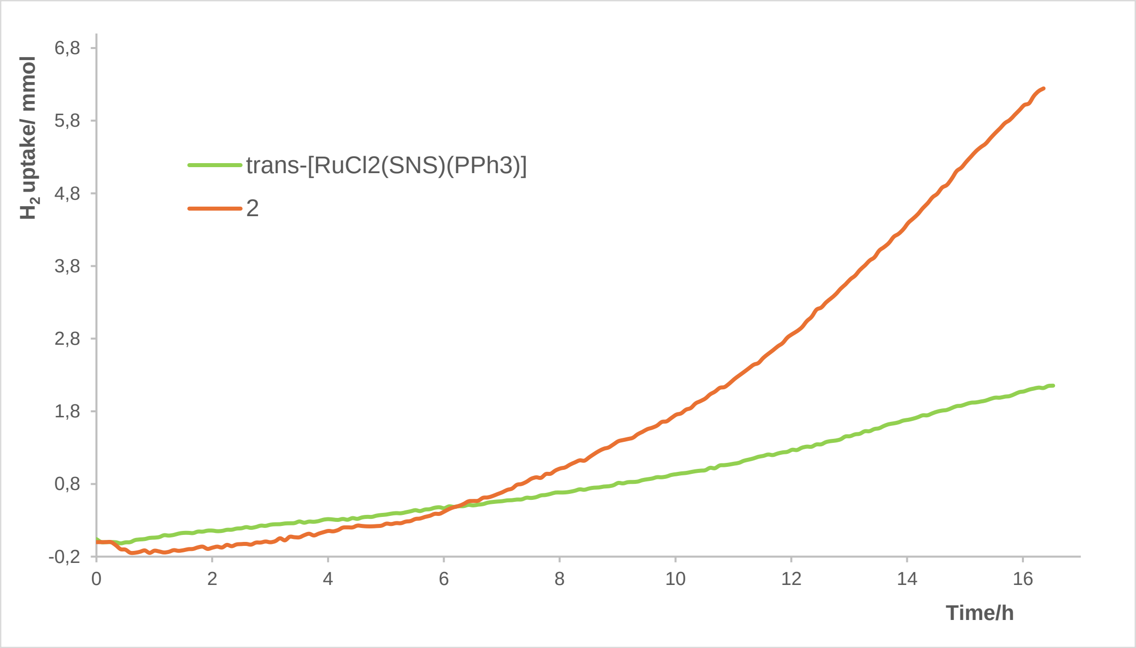

Figure S80. Hydrogen uptake curve of complex **2** and the *trans*-[RuCl₂(SNS)(PPh₃)] derivative for the reduction of methyl decanoate **a** at 40 °C, 27.5 bar of H_2, 50 mol% NaOMe (S/C 50000) and toluene as solvent.

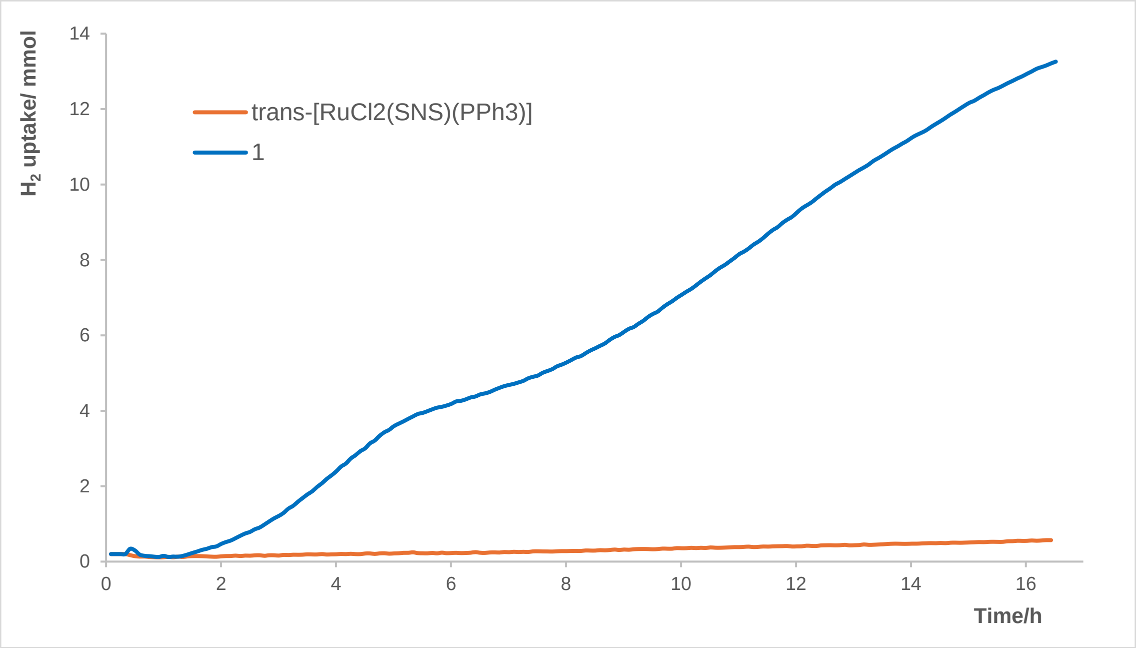

Figure S81. Hydrogen uptake curve of complex **1** and the *trans*-[RuCl$_2$(SNS)(PPh$_3$)] derivative for the solvent-free reduction of methyl decanoate **a** at 40 °C, 27.5 bar of H_2, 50 mol% NaOMe (S/C 100000).

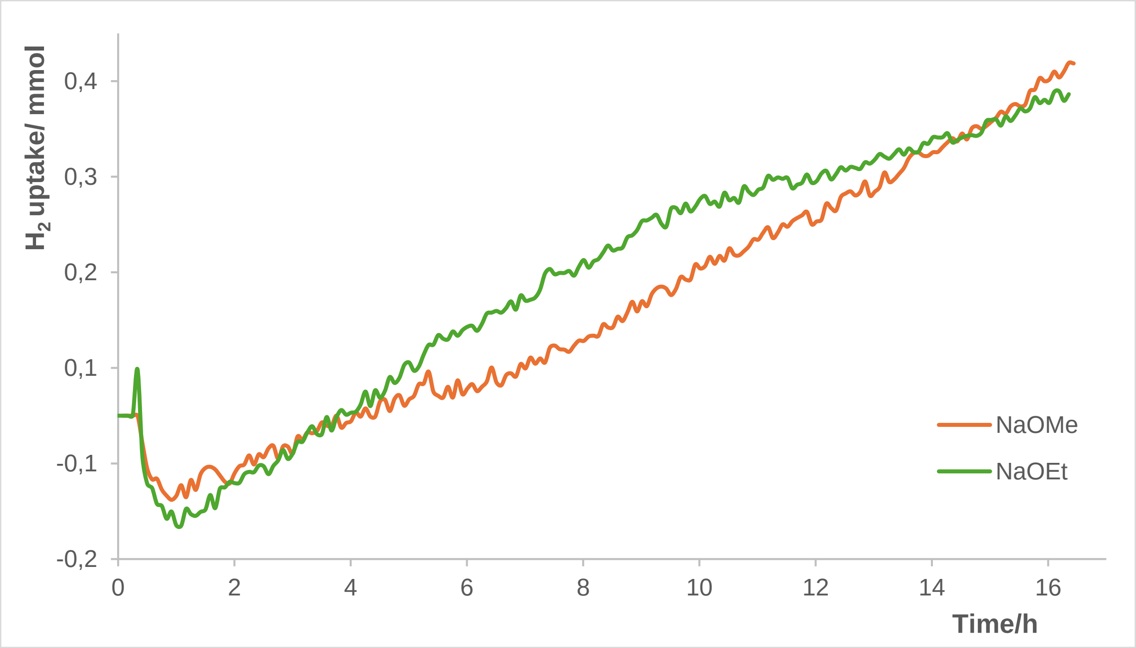

Figure S82. Hydrogen uptake curve of the *trans*-[RuCl$_2$(SNS)(PPh$_3$)] derivative for the solvent-free reduction of methyl decanoate **a** at 40 °C, 27.5 bar of H_2, using different bases at 50 mol% (S/C 100000).

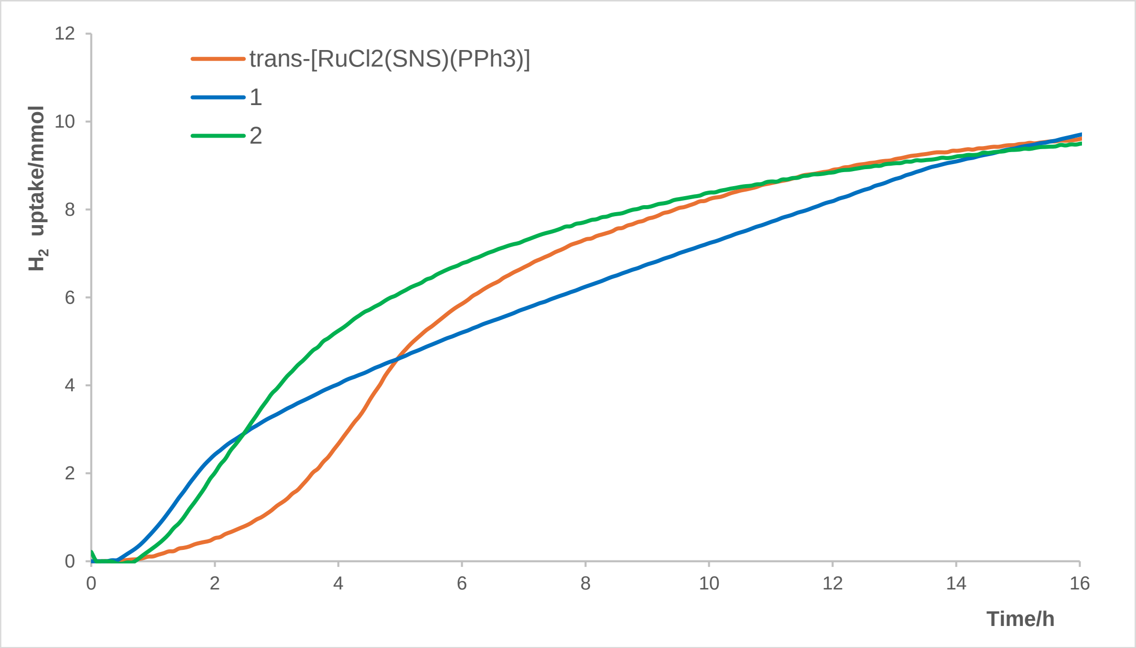

Figure S83. Hydrogen uptake curve of complexes **1**, **2** and the *trans*-[$RuCl_2$(SNS)(PPh_3)] derivative for the solvent-free reduction of ethyl decanoate **b** at 40 °C, 27.5 bar of H_2, 50 mol% NaOEt (S/C 50000).

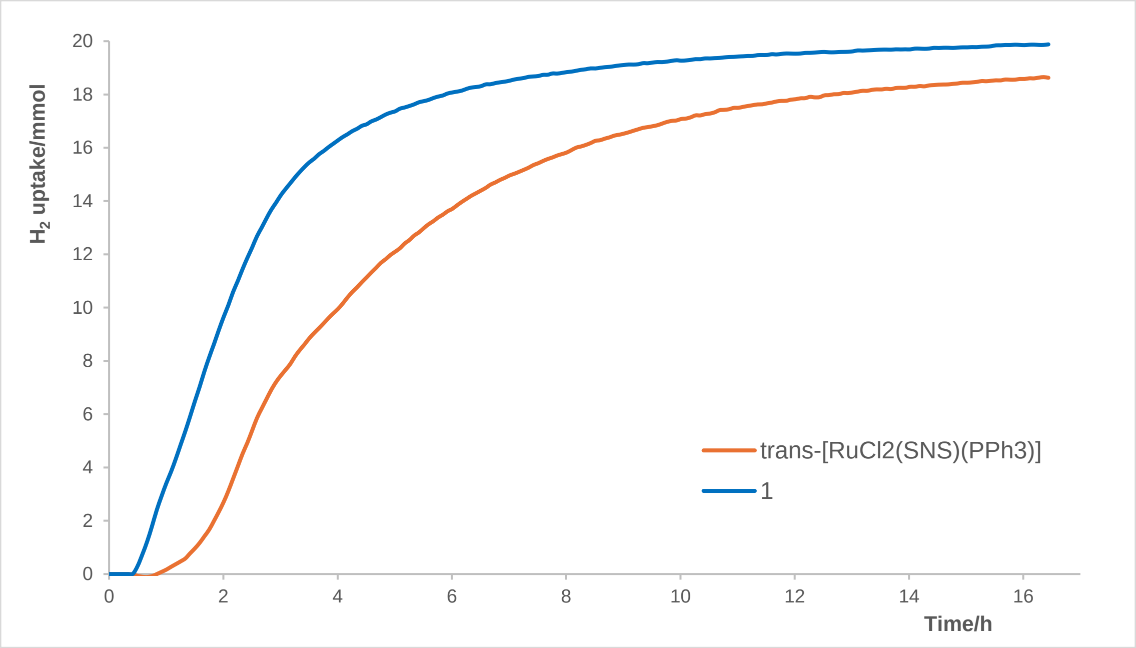

Figure S84. Hydrogen uptake curve of complex **1** and the *trans*-[$RuCl_2$(SNS)(PPh_3)] derivative for the solvent-free reduction of ethyl decanoate **b** at 40 °C, 27.5 bar of H_2, 50 mol% NaOEt (S/C 100000).

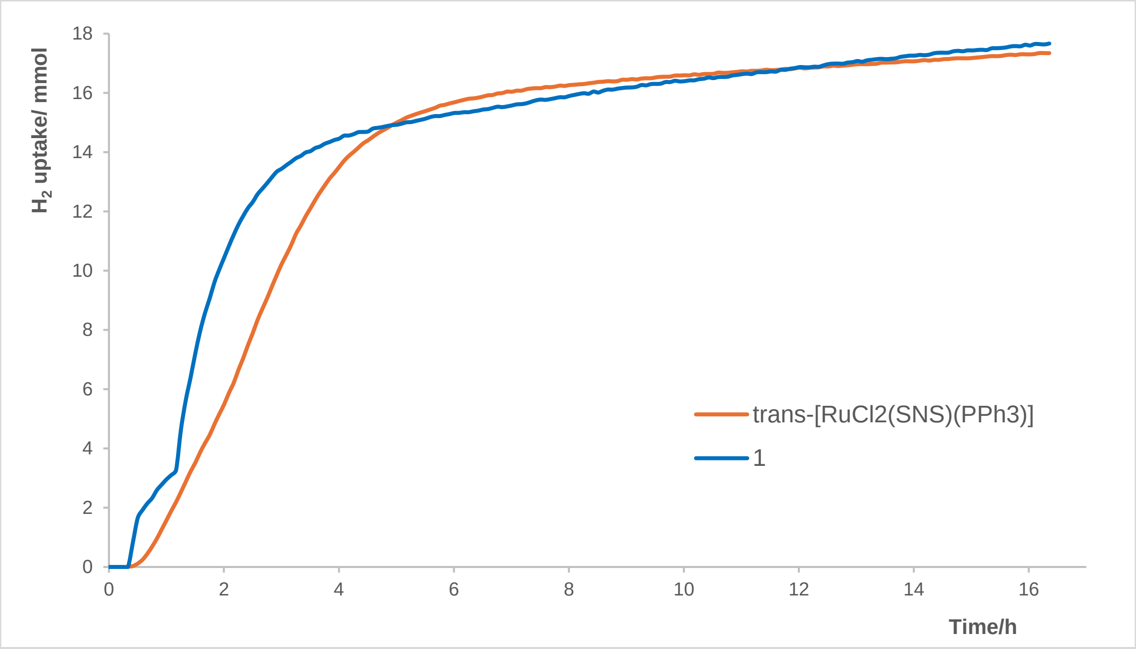

Figure S85. Hydrogen uptake curve of complex **1** and the *trans*-[RuCl$_2$(SNS)(PPh$_3$)] derivative for the solvent-free reduction of ethyl dodecanoate **c** at 40 °C, 27.5 bar of H_2, 50 mol% NaOEt (S/C 44000).

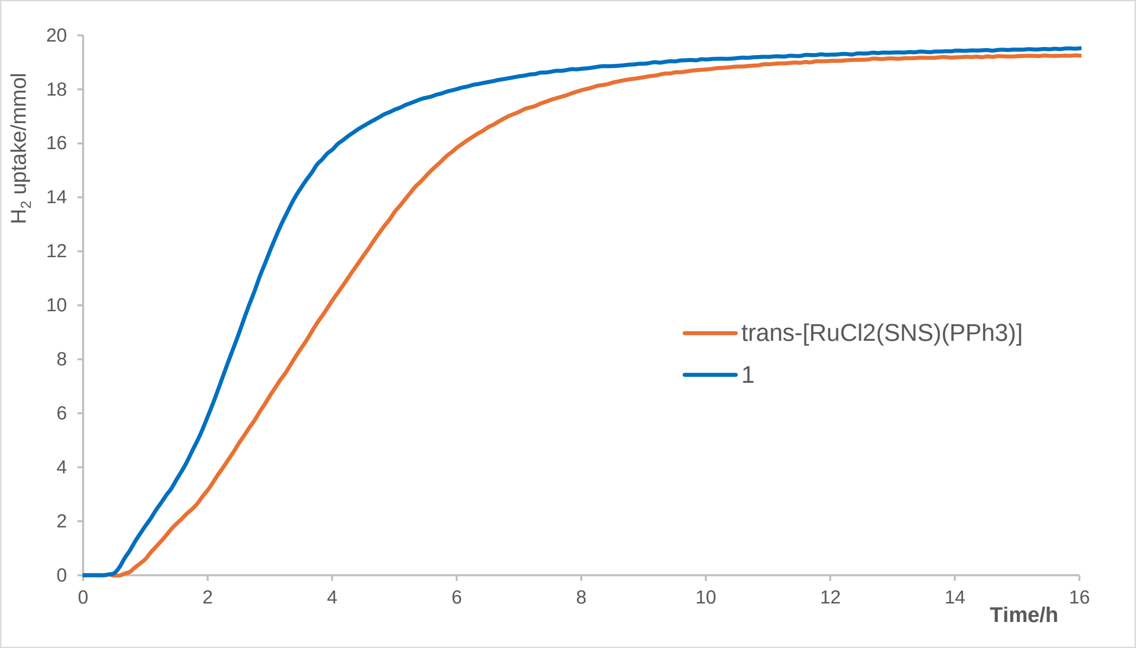

Figure S86. Hydrogen uptake curve of complex **1** and the *trans*-[RuCl$_2$(SNS)(PPh$_3$)] derivative for the solvent-free reduction of ethyl dodecanoate **c** at 40 °C, 27.5 bar of H_2, 50 mol% NaOEt (S/C 100000).

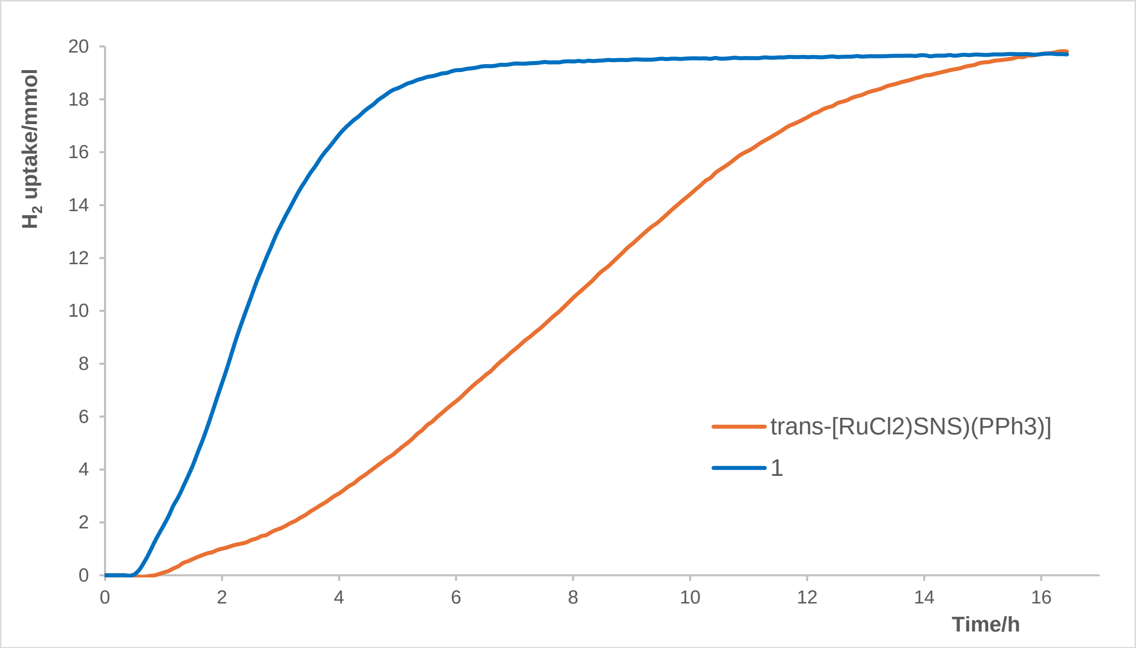

Figure S87. Hydrogen uptake curve of complex **1** and the *trans*-[RuCl$_2$(SNS)(PPh$_3$)] derivative for the solvent-free reduction of ethyl 10-undecenoate **d** at 40 °C, 27.5 bar of H_2, 50 mol% NaOEt (S/C 10000).

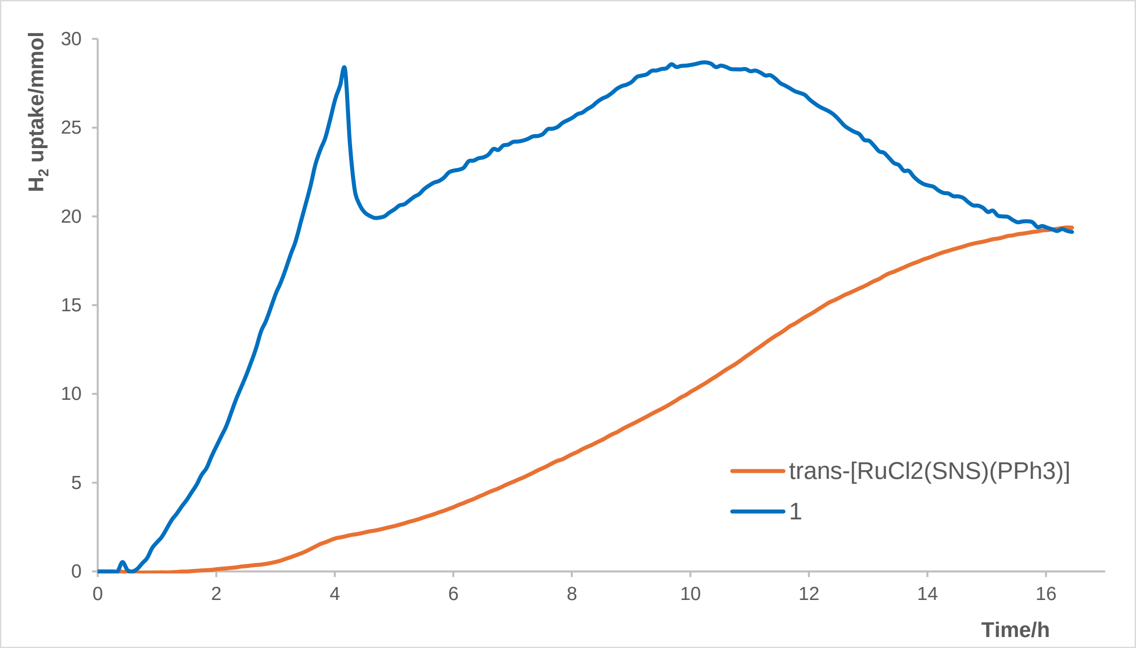

Figure S88. Hydrogen uptake curve of complex **1** and the *trans*-[RuCl$_2$(SNS)(PPh$_3$)] derivative for the solvent-free reduction of ethyl 10-undecenoate **d** at 30 °C, 27.5 bar of H_2, 50 mol% NaOEt (S/C 10000).

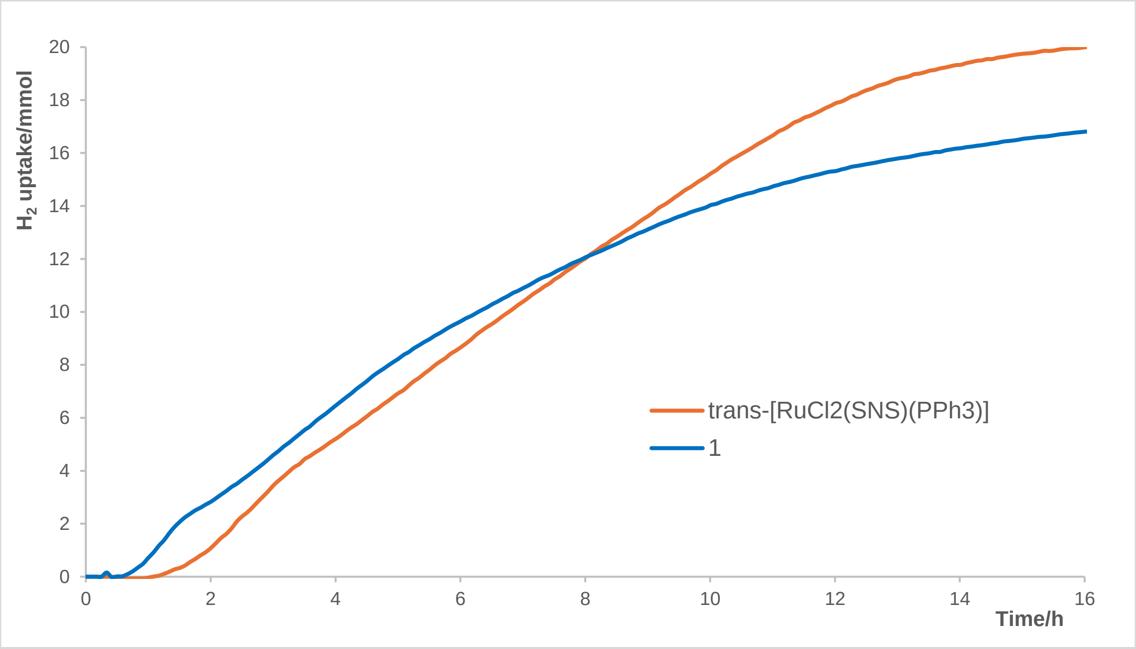

Figure S89. Hydrogen uptake curve of complex **1** and the *trans*-[$RuCl_2$(SNS)(PPh_3)] derivative for the solvent-free reduction of ethyl 10-undecenoate **d** at 40 °C, 27.5 bar of H_2, 50 mol% NaOEt (S/C 50000).

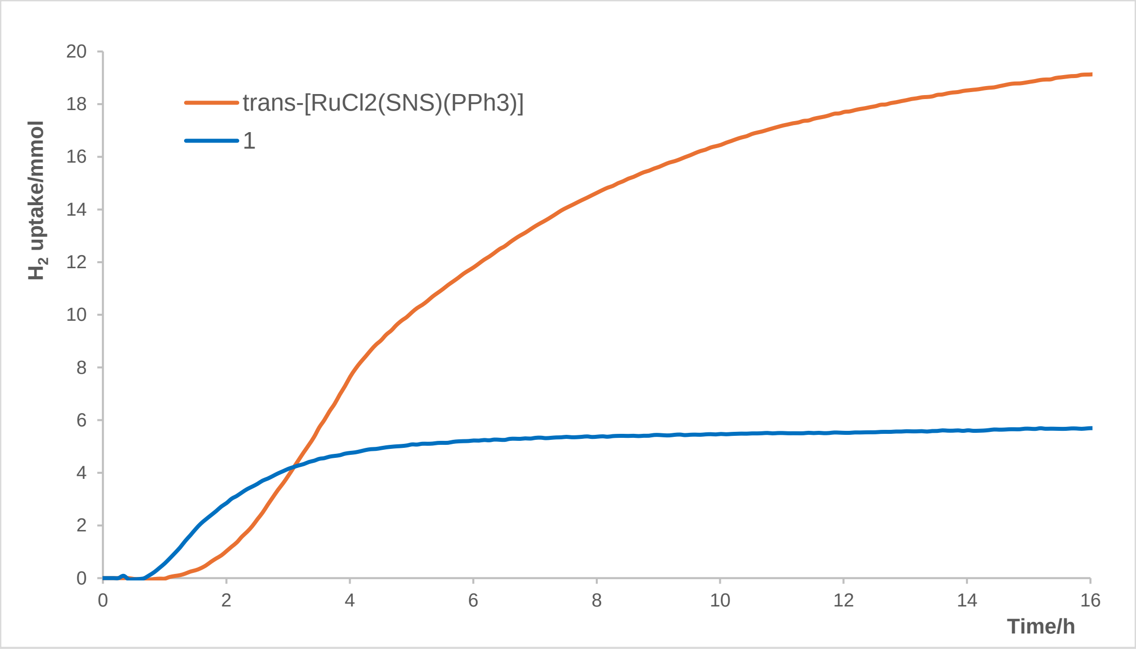

Figure S90. Hydrogen uptake curve of complex **1** and the *trans*-[$RuCl_2$(SNS)(PPh_3)] derivative for the solvent-free reduction of ethyl 10-undecenoate **d** at 40 °C, 27.5 bar of H_2, 50 mol% NaOEt (S/C 100000).

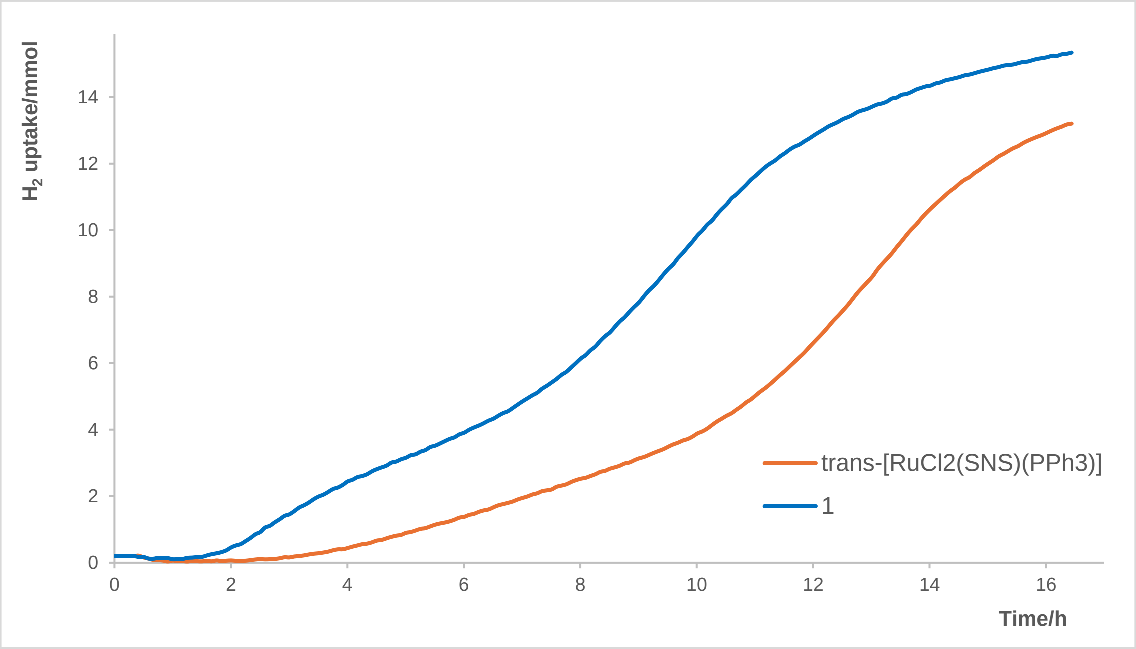

Figure S91. Hydrogen uptake curve of complex **1** and the *trans*-[RuCl$_2$(SNS)(PPh$_3$)] derivative for the solvent-free reduction of methyl oleate **e** at 60 °C, 27.5 bar of H_2, 50 mol% NaOMe (S/C 25000).

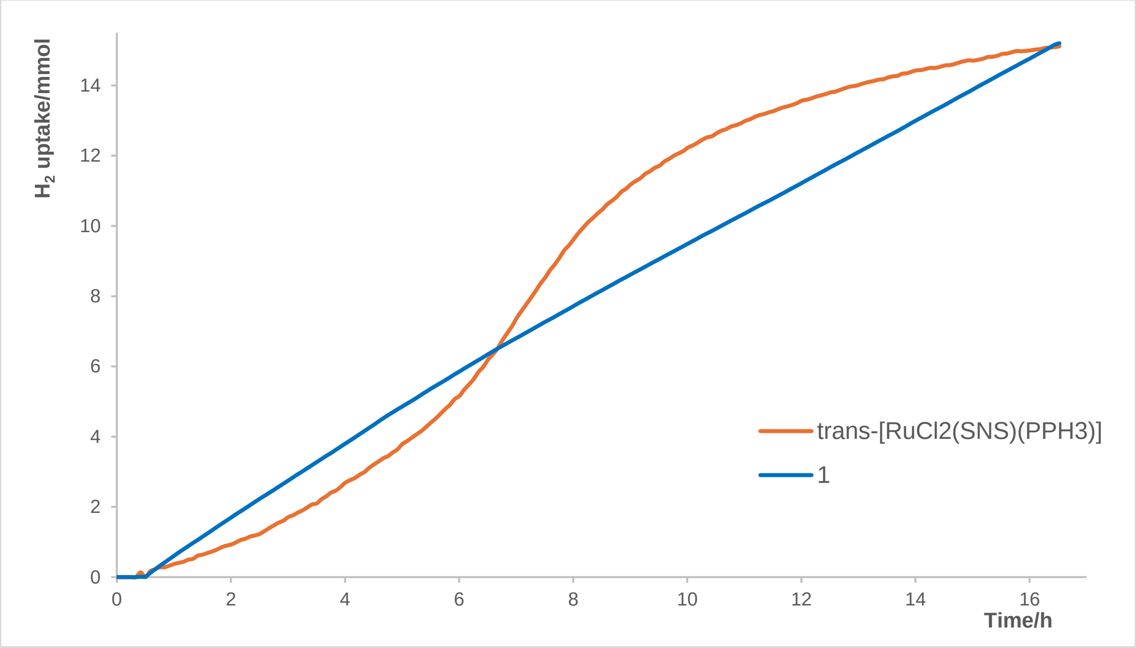

Figure S92. Hydrogen uptake curve of complex **1** and the *trans*-[RuCl$_2$(SNS)(PPh$_3$)] derivative for the solvent-free reduction of methyl oleate **e** at 50 °C, 27.5 bar of H_2, 50 mol% NaOMe (S/C 50000).

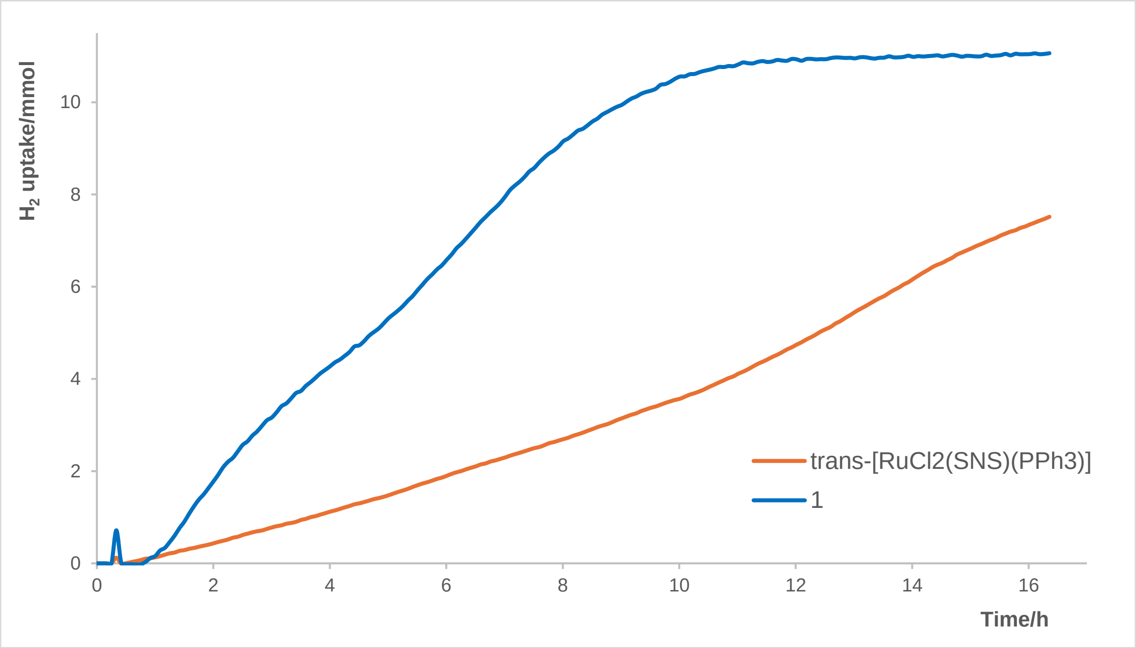

Figure S93. Hydrogen uptake curve of complex **1** and the *trans*-[RuCl$_2$(SNS)(PPh$_3$)] derivative for the solvent-free reduction of methyl oleate **e** at 50 °C, 27.5 bar of H_2, 50 mol% NaOMe (S/C 100000).

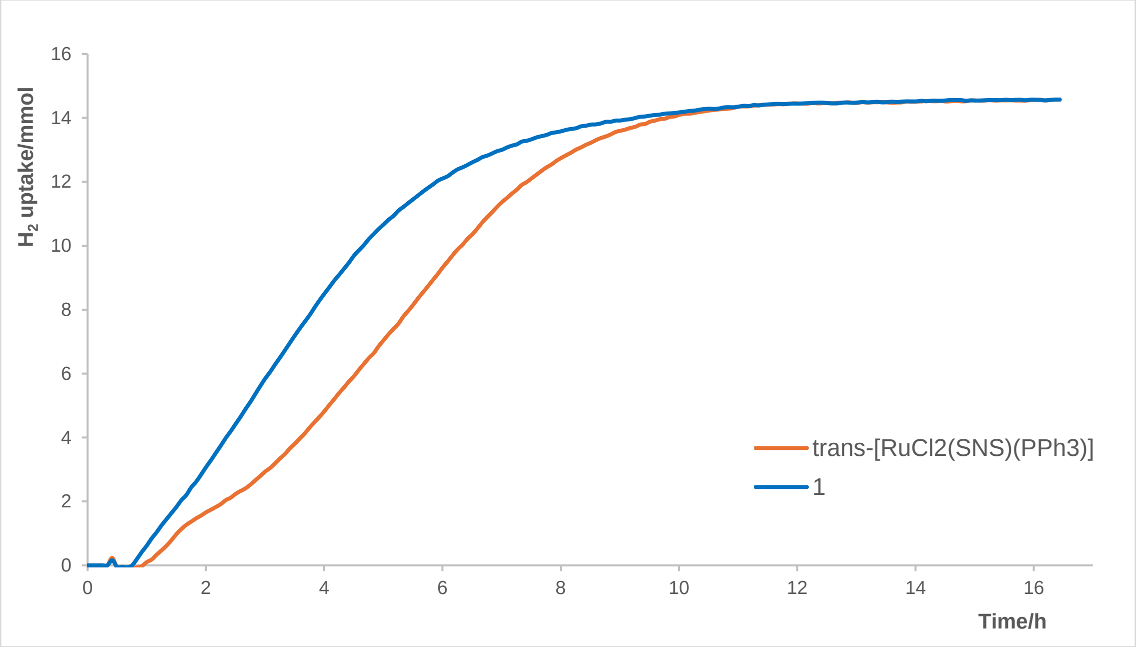

Figure S94. Hydrogen uptake curve of complex **1** and the *trans*-[RuCl$_2$(SNS)(PPh$_3$)] derivative for the solvent-free reduction of ethyl oleate **f** at 40 °C, 27.5 bar of H_2, 50 mol% NaOEt (S/C 50000).

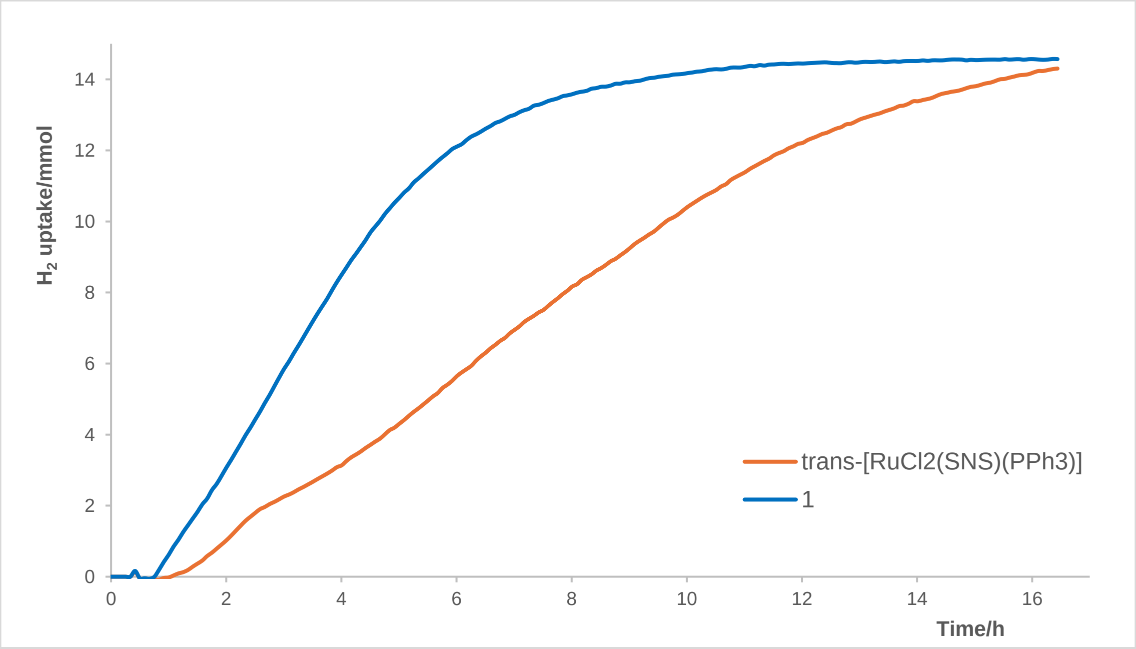

Figure S95. Hydrogen uptake curve of complex **1** and the *trans*-[RuCl$_2$(SNS)(PPh$_3$)] derivative for the solvent-free reduction of ethyl oleate **f** at 40 °C, 27.5 bar of H_2, 50 mol% NaOEt (S/C 100000).

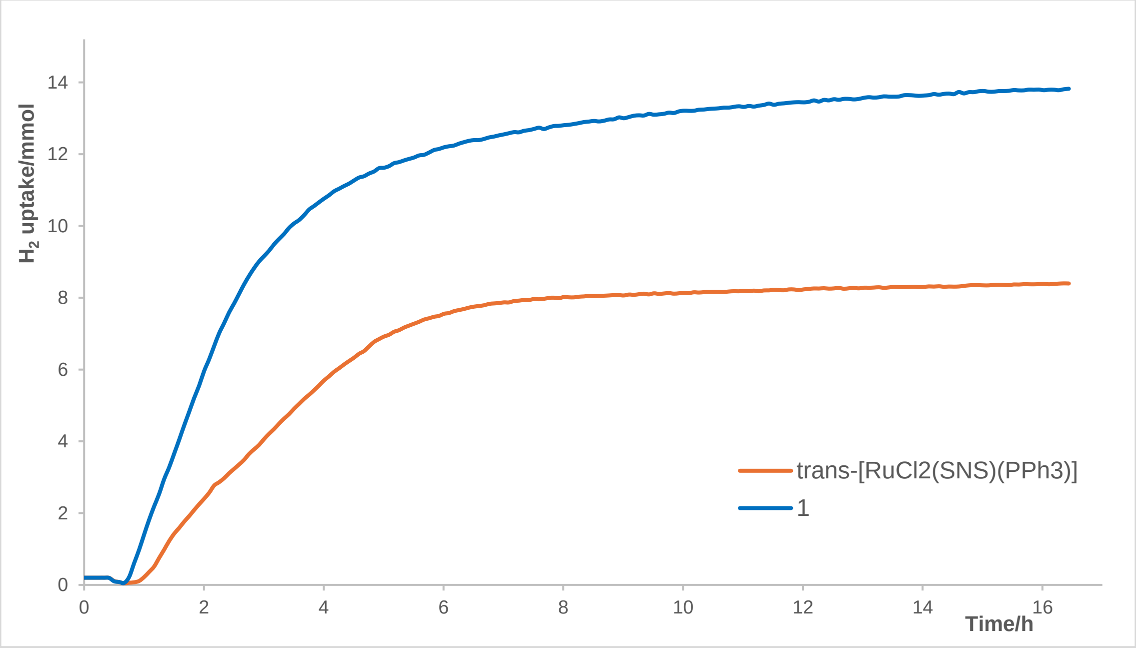

Figure S96. Hydrogen uptake curve of complex **1** and the *trans*-[RuCl$_2$(SNS)(PPh$_3$)] derivative for the solvent-free reduction of ethyl oleate **f** at 50 °C, 27.5 bar of H_2, 50 mol% NaOEt (S/C 100000).

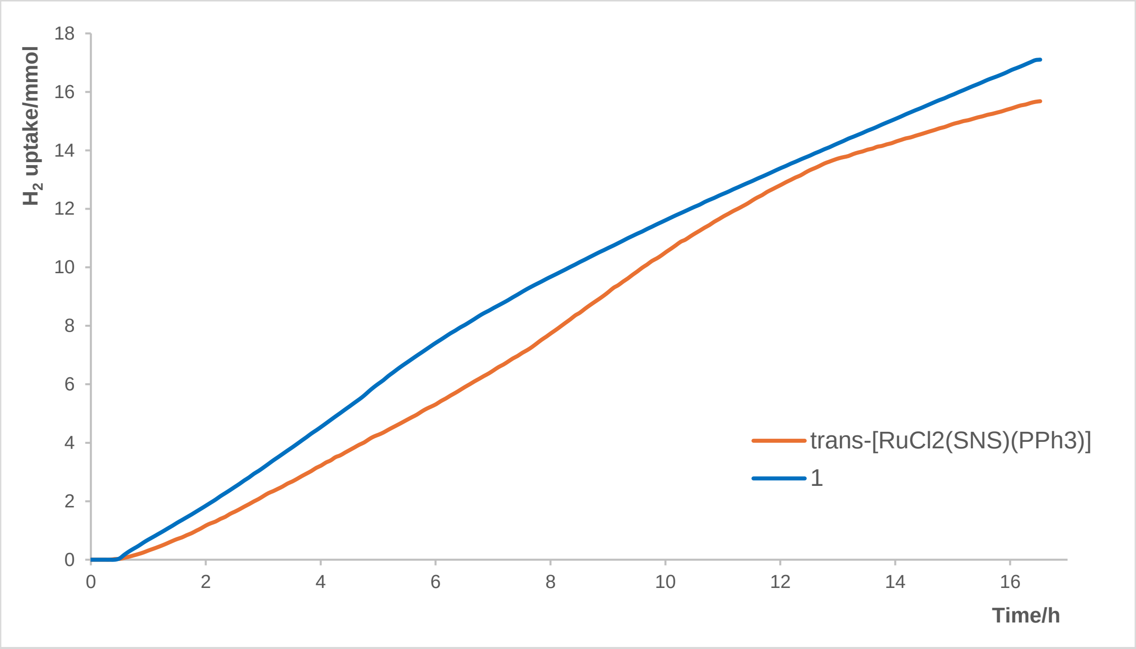

Figure S97. Hydrogen uptake curve of complex **1** and the *trans*-[RuCl$_2$(SNS)(PPh$_3$)] derivative for the solvent-free reduction of methyl benzoate **g** at 40 °C, 27.5 bar of H_2, 50 mol% NaOMe (S/C 100000).

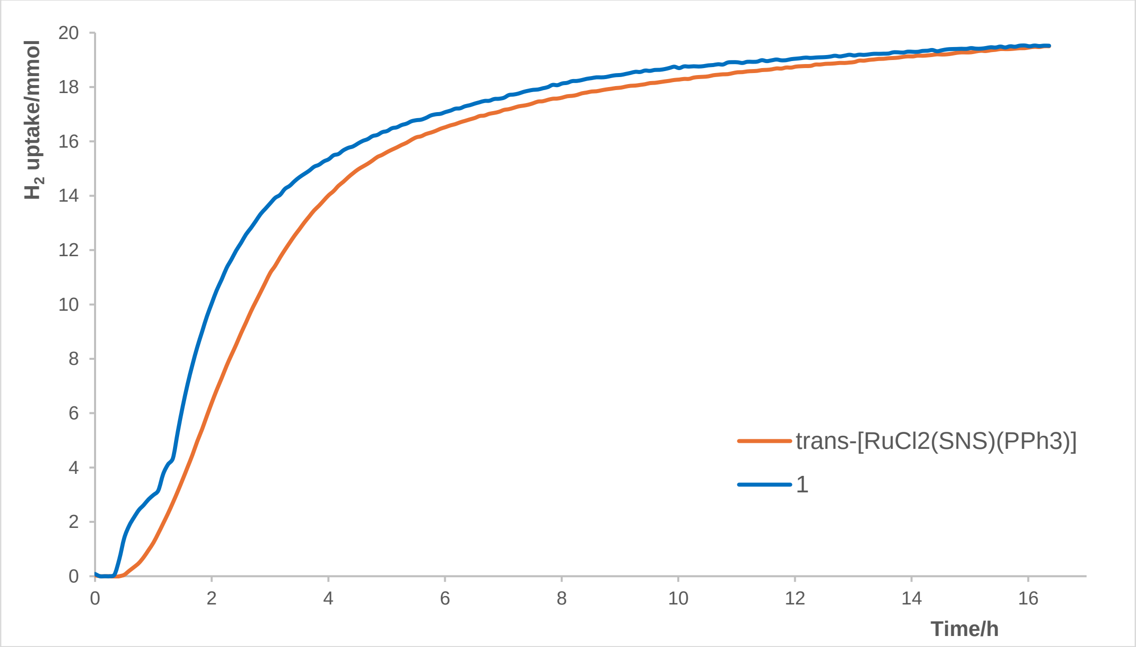

Figure S98. Hydrogen uptake curve of complex **1** and the *trans*-[RuCl$_2$(SNS)(PPh$_3$)] derivative for the solvent-free reduction of ethyl benzoate **h** at 40 °C, 27.5 bar of H_2, 50 mol% NaOEt (S/C 50000).

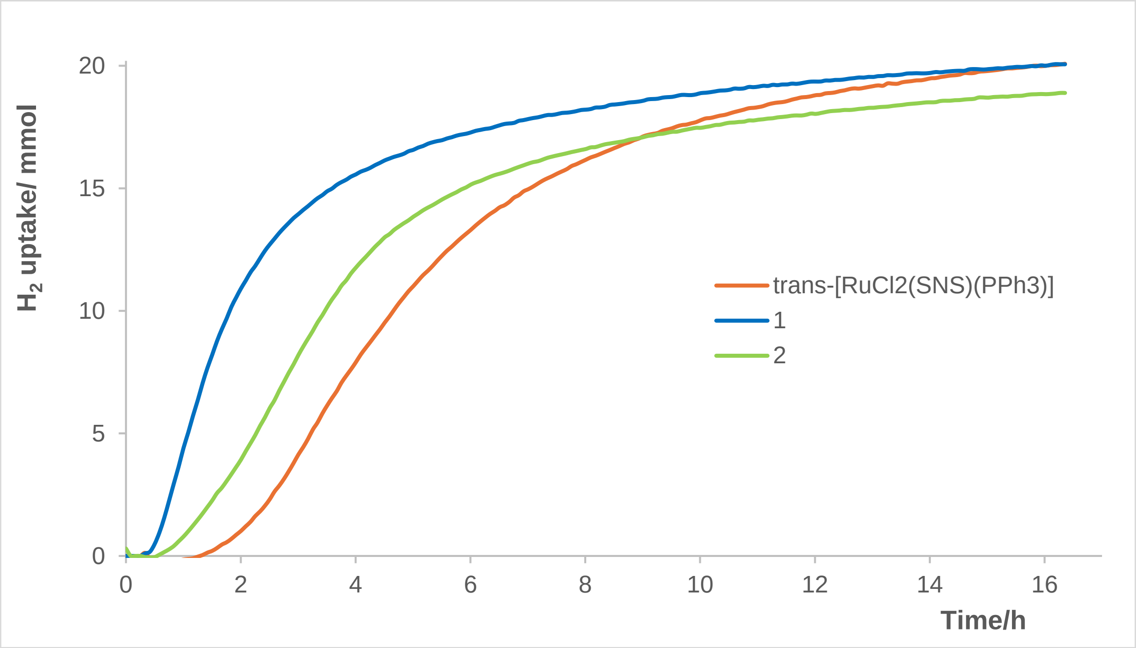

Figure S99. Hydrogen uptake curve of complexes **1**, **2** and the *trans*-[$RuCl_2$(SNS)(PPh_3)] derivative for the solvent-free reduction of ethyl benzoate **h** at 40 °C, 27.5 bar of H_2, 50 mol% NaOEt (S/C 100000).

GC analytical method for methyl and ethyl decanoate hydrogenations

The crude reaction mixtures were quenched with HCl (1M in water) and extracted with MTBE before analysis using achiral gas chromatography to measure reaction conversions. The following analytical method was used.

Instrument: Varian GC 3900 system
Column: Agilent HP-88: 60 m x 0.25 mm x 0.2 μm
Column Oven Temperature Gradient: initial T = 115 °C for 1 min, ramped to 187 °C at 3 °C/min, then to 240 °C at 5 °C/min, which is maintained for other 1.4 min for a total of 37 min of analysis.
Carrier Gas: N_2 at 1.2 mL/min.
Detector: FID (T = 240 °C)
Sample diluent: MTBE
Injection volume: 5.0 μL

Retention time of identified compounds

Compound	Elution Time (min)
Methyl decanoate	8.09
Ethyl decanoate	8.80
1-decanol	10.41
Decyl decanoate	24.01

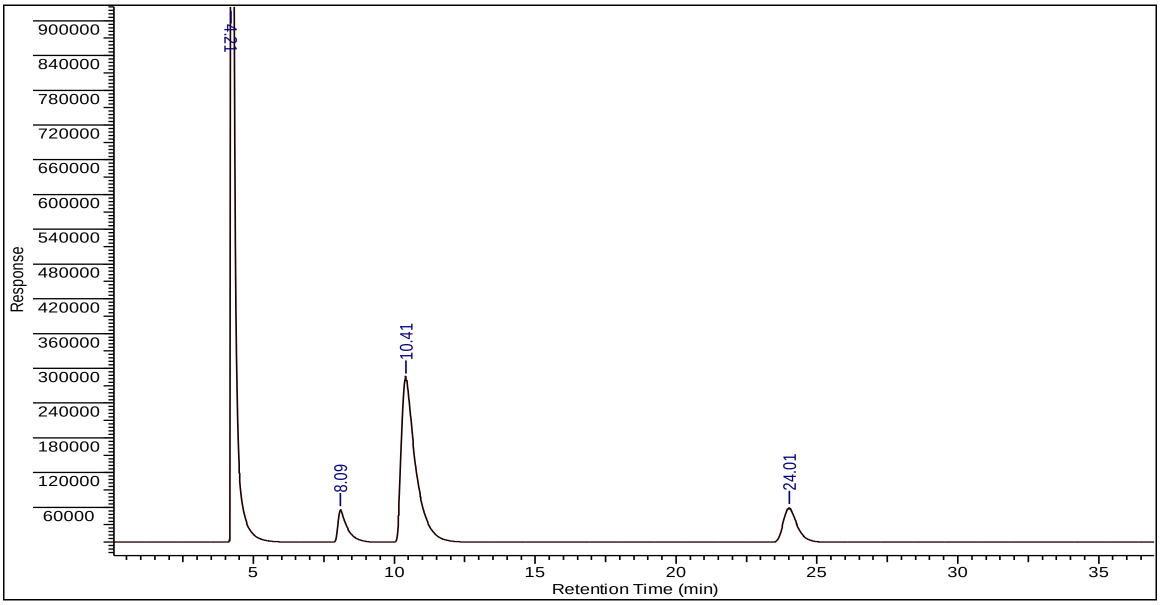

Figure S100. Representative example of GC chromatogram of the catalytic HY of methyl decanoate **a** using RuSNS complexes containing all the species involved in the reaction (methyl decanoate, decyl decanoate, and 1-decanol product).

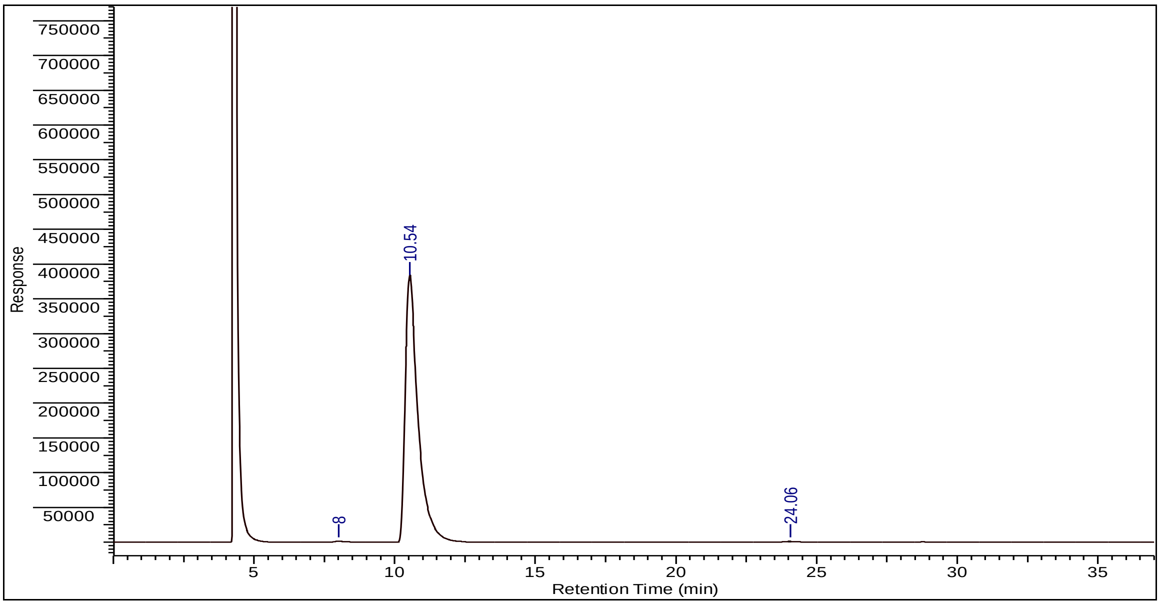

No.	Name	tR	Peak Area	Area Percent	Width
1	methyl decanoate	8.004	38130012.000	0.310	0.758
2	1-decanol	10.539	12206906368.000	99.403	0.685
3	decyl decanoate	24.061	35209988.000	0.287	0.854

Figure S101. GC trace of products of methyl decanoate **a** hydrogenation catalysed by complex **1** (S/C 50000) with 50 mol% NaOEt, 27.5 bar of H_2 and 40 °C, showing full conversion to 1-decanol.

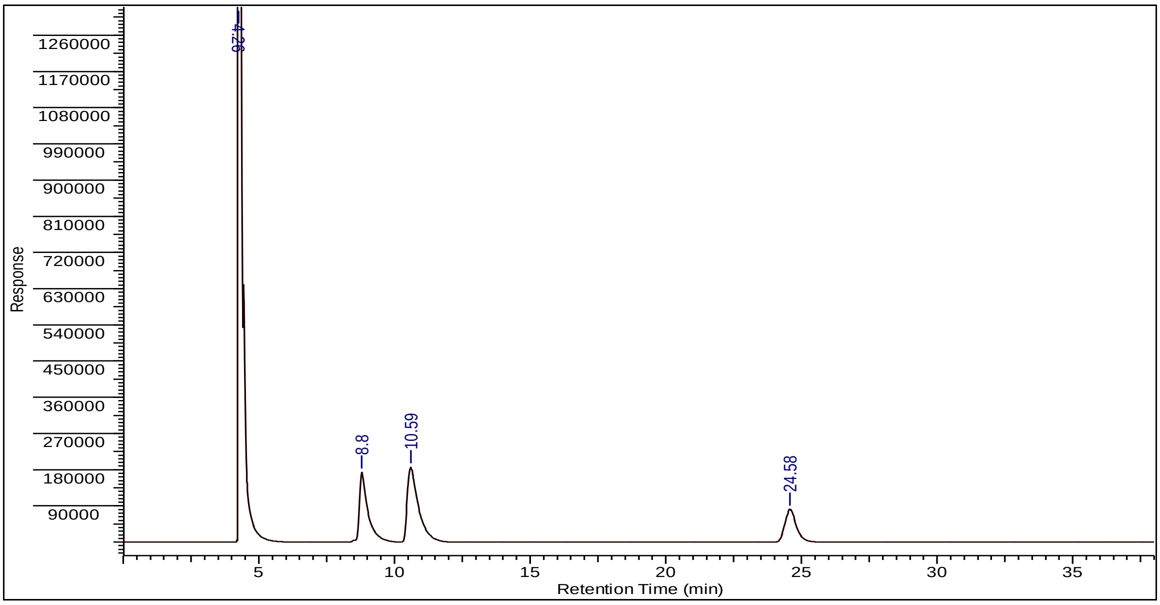

Figure S102. Representative example of GC chromatogram of the catalytic HY of ethyl decanoate **b** using RuSNS complexes containing all the species involved in the reaction (ethyl decanoate, decyl decanoate, and 1-decanol product).

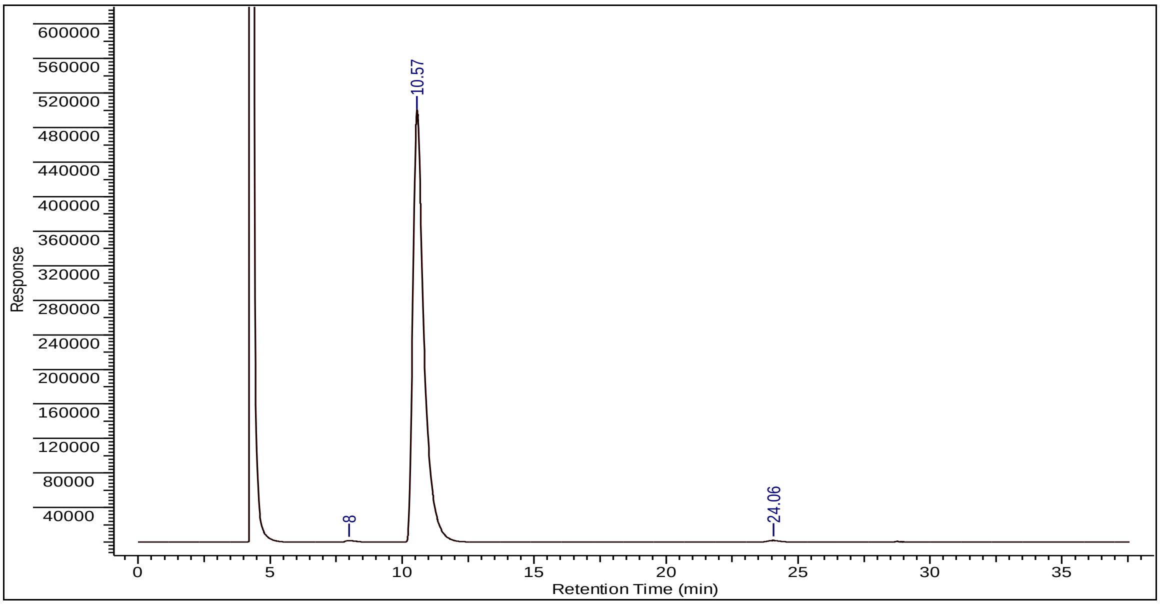

No.	Name	tR	Peak Area	Area Percent	Width
1	ethyl decanoate	7.996	43016648.000	0.293	0.621
2	1-decanol	10.570	14594448384.000	99.312	0.630
3	decyl decanol	24.060	58145940.000	0.396	0.766

Figure S103. GC trace of products of ethyl decanoate **b** hydrogenation catalysed by complex **1** (S/C 50000) with 50 mol% NaOEt, 27.5 bar of H_2 and 40 °C, showing full conversion to 1-decanol.

GC analytical method for ethyl dodecanoate hydrogenations

The crude reaction mixtures were quenched with HCl (1M in water) and extracted with MTBE before analysis using achiral gas chromatography to measure reaction conversions. The following analytical method was used.

Instrument: Varian GC 3900 system

Column: Agilent HP-88: 60 m x 0.25 mm x 0.2 μm

Column Oven Temperature Gradient: initial T = 115 °C for 1 min, ramped to 187 °C at 3 °C/min, then to 240 °C at 5 °C/min, which is maintained for other 1.4 min for a total of 37 min of analysis.

Carrier Gas: N_2 at 1.2 mL/min.

Detector: FID (T = 240 °C)

Sample diluent: MTBE

Injection volume: 5.0 μL

Retention time of identified compounds

Compound	Elution Time (min)
Ethyl dodecanoate	8.50
1-dodecanol	11.91

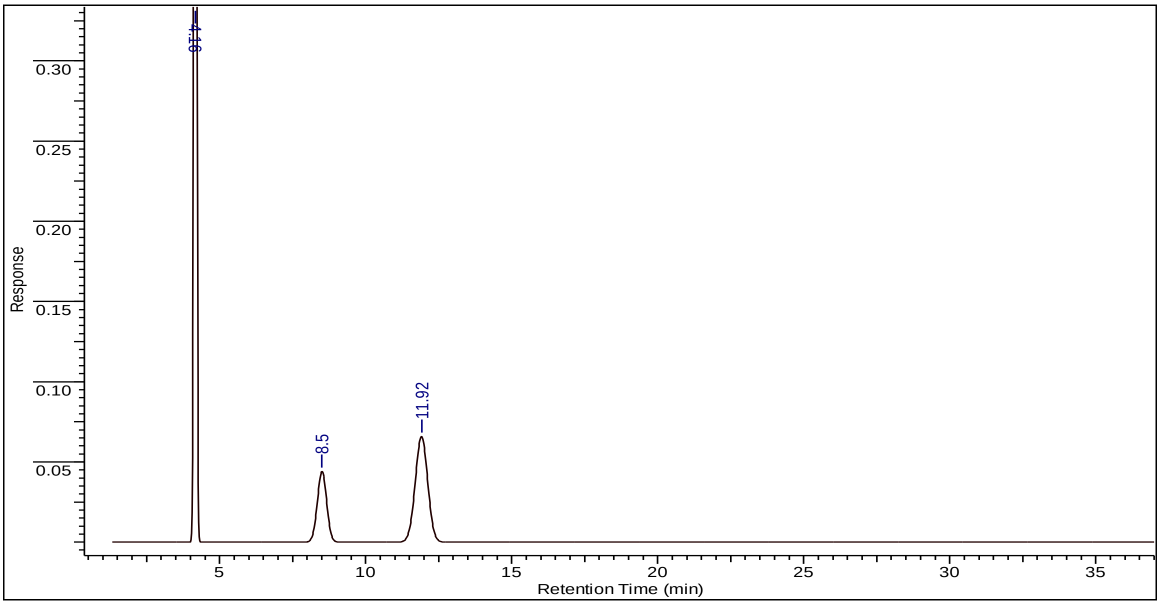

Figure S104. Representative example of GC chromatogram of the catalytic HY of ethyl dodecanoate **c** using RuSNS complexes containing all the species involved in the reaction (ethyl dodecanoate and 1-dodecanol product).

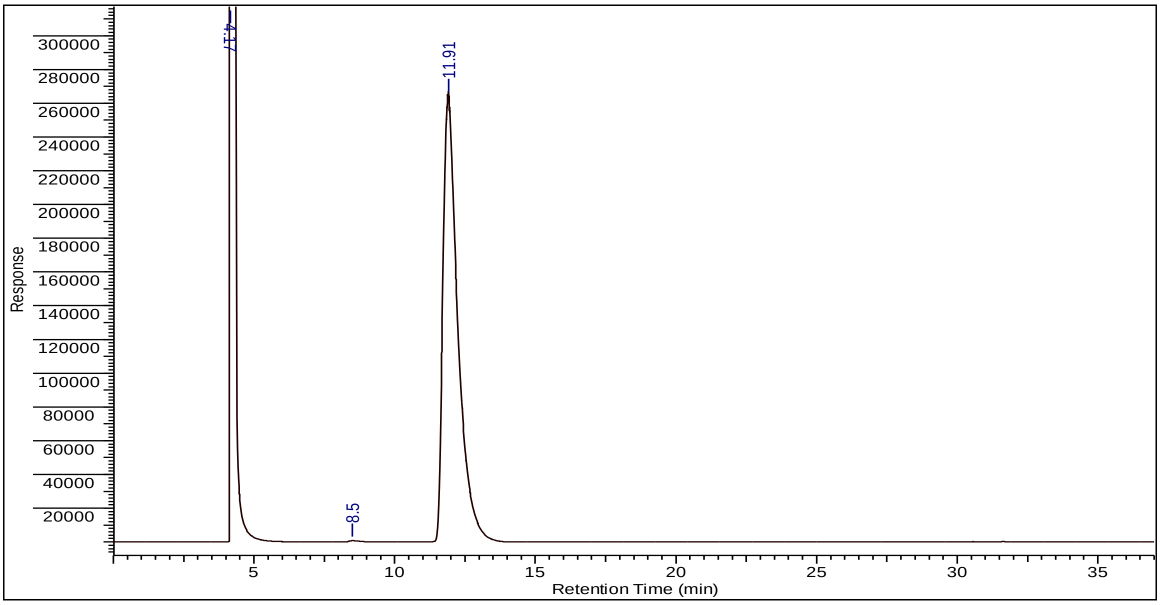

Figure S105. GC trace of products of ethyl dodecanoate **c** hydrogenation catalysed by complex **1** (S/C 50000) with 50 mol% NaOEt, 27.5 bar of H_2 and 40 °C, showing full conversion to 1-dodecanol.

GC analytical method for ethyl 10-undecenoate hydrogenations

The crude reaction mixtures were quenched with HCl (1M in water) and extracted with MTBE before analysis using achiral gas chromatography to measure reaction conversions. The following analytical method was used.

Instrument: Varian GC 3900 system

Column: Agilent HP-88: 60 m x 0.25 mm x 0.2 μm

Column Oven Temperature Gradient: initial T = 115 °C for 1 min, ramped to 187 °C at 3 °C/min, then to 240 °C at 5 °C/min, which is maintained for other 1.4 min for a total of 37 min of analysis.

Carrier Gas: N_2 at 1.2 mL/min.

Detector: FID (T = 240 °C)

Sample diluent: MTBE

Injection volume: 5.0 μL

Retention time of identified compounds

Compound	Elution Time (min)
Ethyl 10-undecenoate	11.01
1-undecanol	12.50
10-undecen-1-ol	14.37

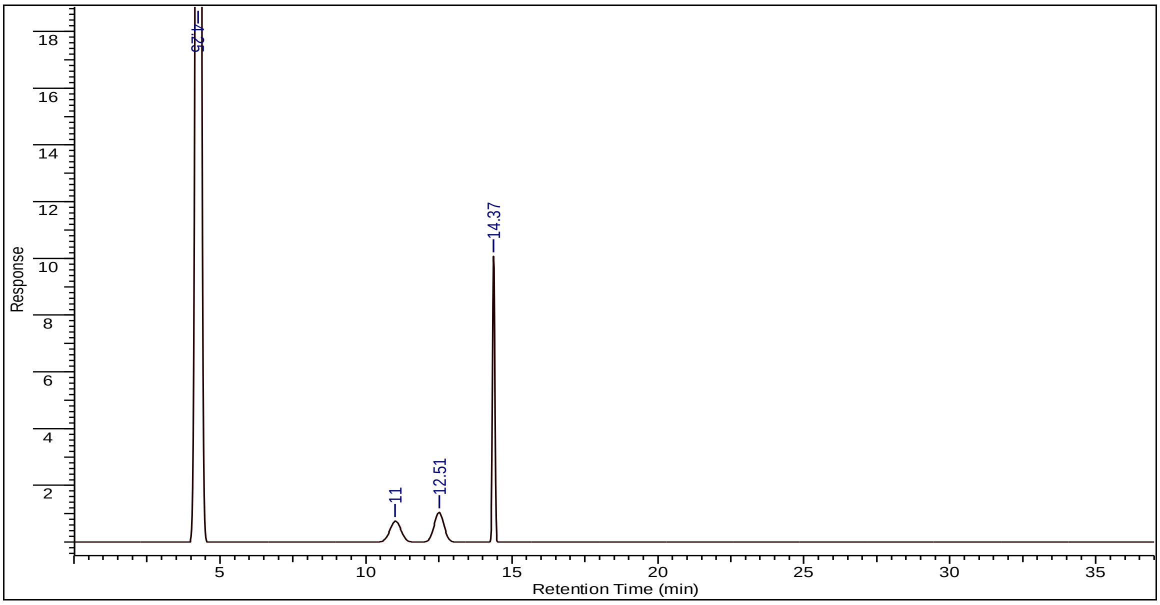

Figure S106. Representative example of GC chromatogram of the catalytic HY of ethyl 10-undecenoate **d** using RuSNS complexes containing all the species involved in the reaction (ethyl 10-undecenoate, 1-undecanol and 10-undecen-1-ol).

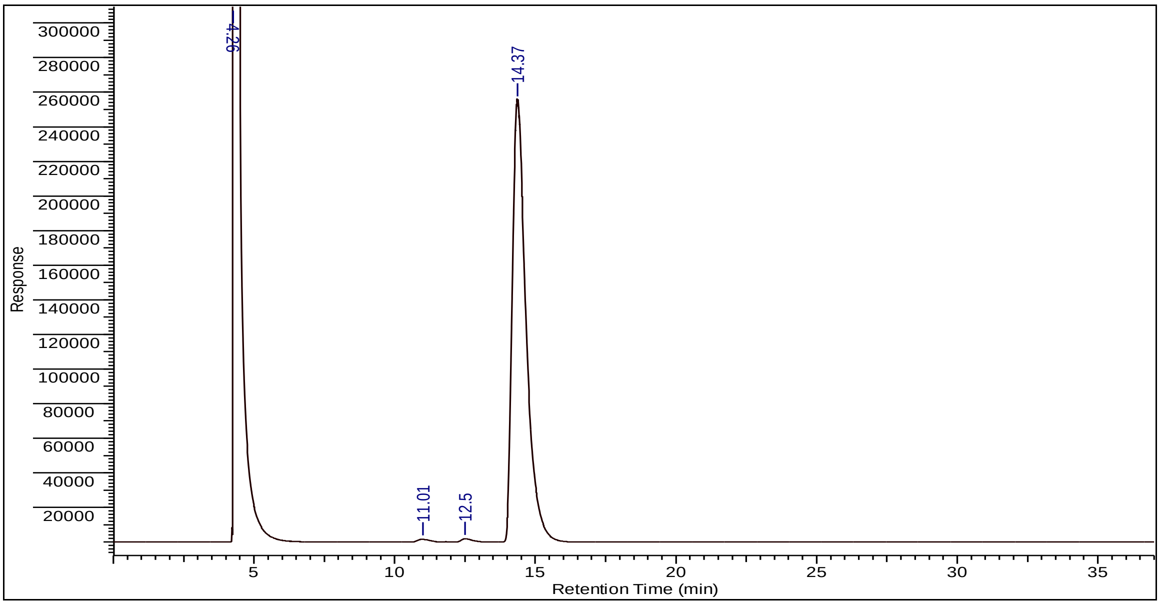

No.	Name	tR	Peak Area	Area Percent	Width
1	ethyl 10-undecenoate	11.008	44027932.000	0.498	0.720
2	1-undecanol	12.501	49338472.000	0.558	0.634
3	10-undecen-1-ol	14.371	8751792128.000	98.944	0.765

Figure S107. GC trace of products of ethyl 10-undecenoate **d** hydrogenation catalysed by complex **1** (S/C 10000) with 50 mol% NaOEt, 27.5 bar of H_2 and 40 °C, showing full conversion to 10-undecen-1-ol.

GC analytical method for methyl and ethyl oleate hydrogenations

The crude reaction mixtures were quenched with HCl (1M in water) and extracted with MTBE before analysis using achiral gas chromatography to measure reaction conversions. The following analytical method was used.

Instrument: Varian GC 3900 system

Column: Agilent HP-88: 60 m x 0.25 mm x 0.2 μm

Column Oven Temperature Gradient: initial T = 115 °C for 1 min, ramped to 187 °C at 3 °C/min, then to 240 °C at 5 °C/min, which is maintained for other 19.4 min for a total of 55 min of analysis.

Carrier Gas: N_2 at 1.2 mL/min.

Detector: FID (T = 240 °C)

Sample diluent: MTBE

Injection volume: 1.0 μL

Retention time of identified compounds

Compound	Elution Time (min)
Methyl Palmitate (C16:0)	19.66
Palmityl Alcohol (C16:0)	22.93
Methyl Stearate (C18:0)	21.15
Stearyl Alcohol (C18:0)	25.05
Methyl Oleate (C18:1)	25.17
Oleyl Alcohol (C18:1 Z)	28.19
Palmitoleyl Alcohol (C16:1 Z)	27.08
Methyl linoleate (C18:2)	26.69
Linoleyl Alcohol (C18:2)	29.54

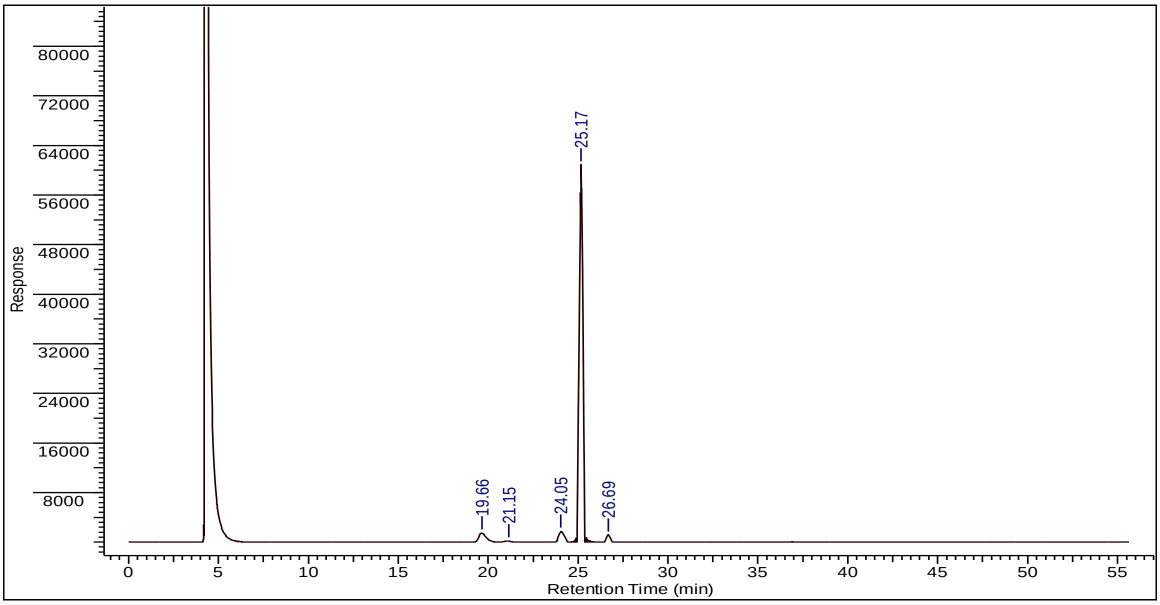

No.	Name	tR	Peak Area	Area Percent	Width
1	Methyl Palmitate (C16:0)	19.658	529337824.000	7.128	0.672
2	Methyl Stearate (C18:0)	21.153	666021.000	1.506	0.583
3	Methyl palmitolate (C16:1)	24.046	123567334.00	4.223	0.527
4	Methyl Oleate (C18:1)	25.174	8432724480.000	84.096	0.739
5	Methyl linoleate (C18:2)	26.695	2326733312.000	3.047	0.629

Figure S108. Representative GC trace of starting material reference for technical grade methyl oleate **e**, showing composition (%) as a ratio of peak areas.

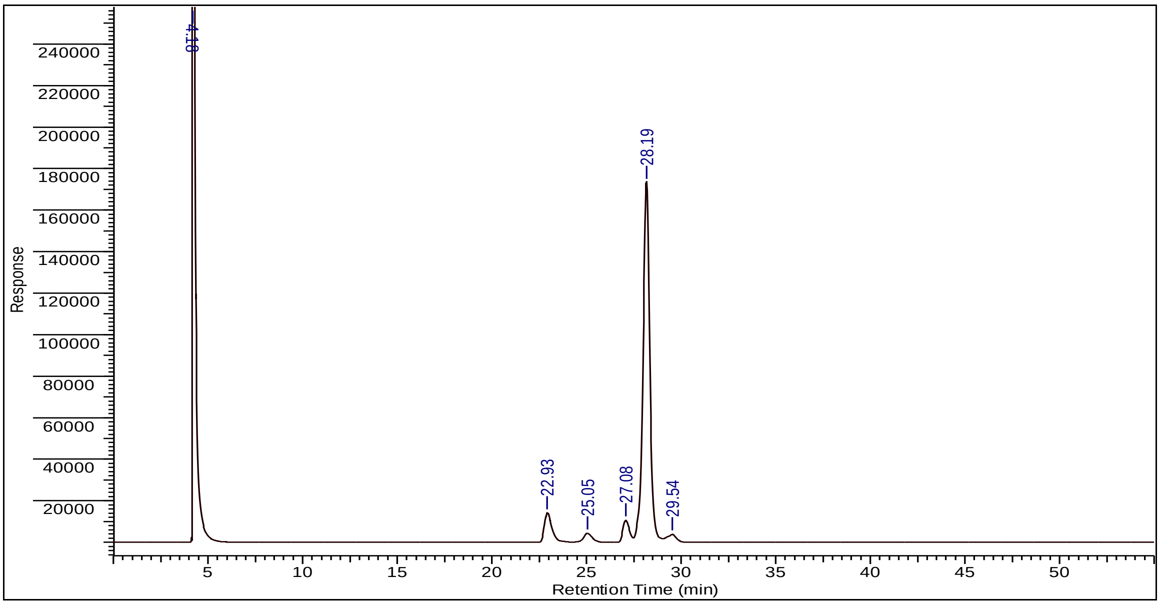

No.	Name	tR	Peak Area	Area Percent	Width
1	Palmityl Alcohol (C16:0)	22.931	393023840.000	7.497	0.632
2	Stearyl Alcohol (C18:0)	25.049	137728368.000	1.627	0.729
3	Palmitoleyl Alcohol (C16:1 (Z))	27.080	251530144.000	4.798	0.576
4	Oleyl Alcohol (C18:1 Z)	28.187	4305246720.000	83.119	0.550
5	Linoleyl Alcohol (C18:2)	29.537	155155232.000	2.959	0.945

Figure S109. Representative GC trace of hydrogenation products of methyl oleate **e** catalysed by complex **1** at 27.5 bar H_2 and 50 °C with 50 mol% NaOMe (S/C 25000).

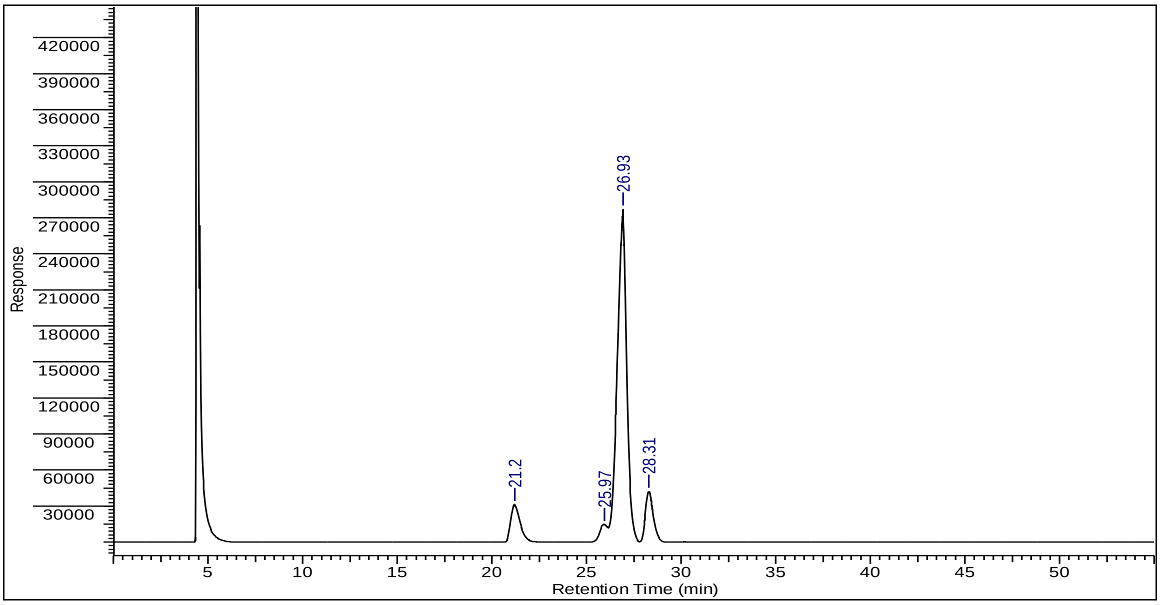

No.	Name	tR	Peak Area	Area Percent	Width
1	ethyl palmitate	21.200	1077972480.	6.245	0.804
2	ethyl palmitoleate	25.965	387381376.0	1.179	0.870
3	ethyl oleate	26.933	9497743360.	78.935	0.776
4	ethyl linoleate	28.306	1223611904.	12.041	0.671

Figure S110. GC trace of starting material reference for technical grade ethyl oleate **f**, showing composition (%) as a ratio of peak areas.

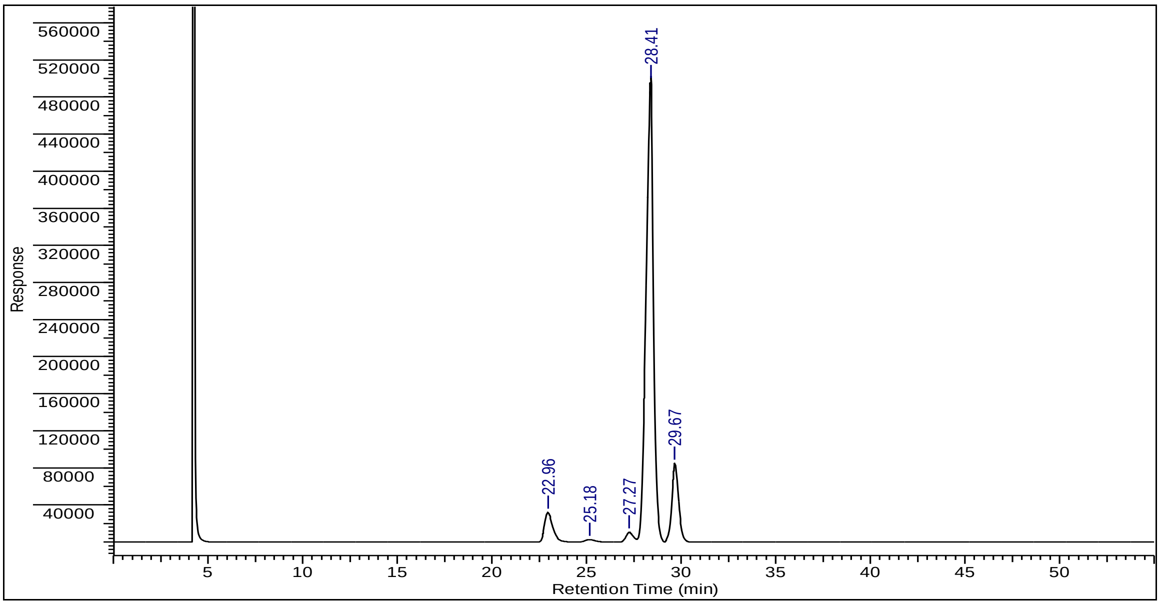

No.	Name	tR	Peak Area	Area Percent	Width
1	Palmityl alcohol (C16:0)	22.963	965901376.0	5.680	0.699
2	Stearyl alcohol (C18:0)	25.175	93709280.0	0.551	0.859
3	Palmitoleyl alcohol (C16:1 (Z))	27.265	249935680.0	1.470	0.652
4	Oleyl alcohol (C18:1 (Z))	28.409	1590685696.	79.919	0.618
5	Linoleyl alcohol (C18:2 (Z))	29.667	2105272320.	12.380	0.567

Figure S111. GC trace of hydrogenation products of ethyl oleate **f** catalyzed by complex **1** at 27.5 bar H_2 and 50 °C with 50 mol% NaOEt (S/C 50000).

GC analytical method for methyl and ethyl benzoate hydrogenations

The crude reaction mixtures were quenched with HCl (1M in water) and extracted with MTBE before analysis using achiral gas chromatography to measure reaction conversions. The following analytical method was used.

Instrument: Varian GC 3900 system

Column: Agilent HP-88: 60 m x 0.25 mm x 0.2 μm

Column Oven Temperature Gradient: initial T = 115 °C for 1 min, ramped to 187 °C at 3 °C/min, then to 240 °C at 5 °C/min, which is maintained for other 1.4 min for a total of 37 min of analysis.

Carrier Gas: N_2 at 1.2 mL/min.

Detector: FID (T = 240 °C)

Sample diluent: MTBE

Injection volume: 1.0 μL

Retention time of identified compounds

Compound	Elution Time (min)
Methyl benzoate	6.75
Ethyl benzoate	7.02
Benzyl alcohol	9.38
Benzyl benzoate	19.79

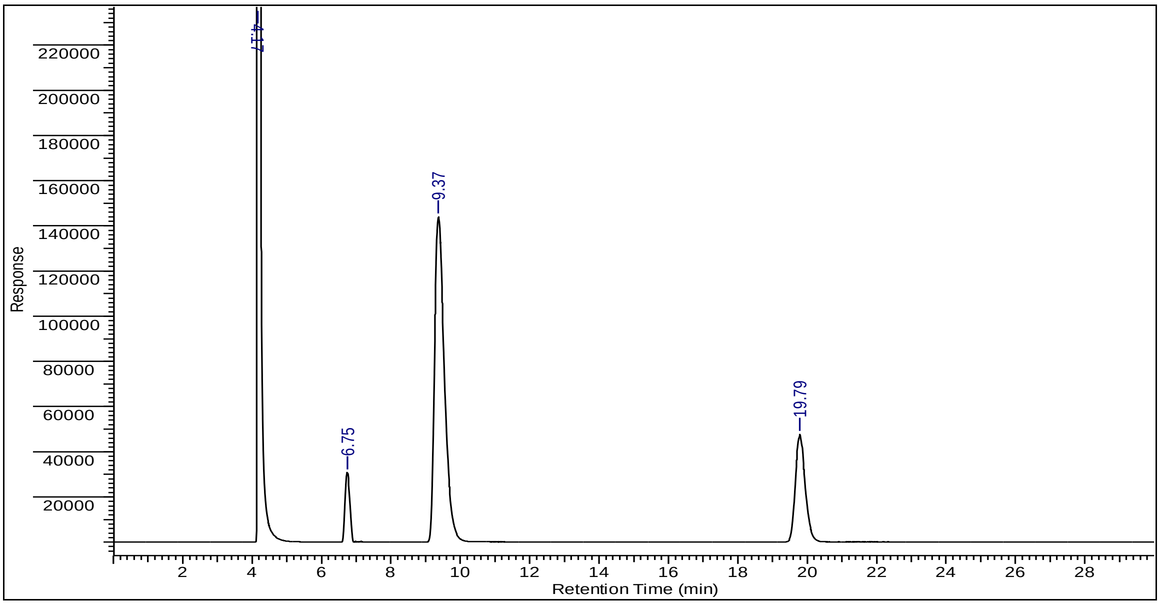

Figure S112. Representative example of GC chromatogram of the catalytic HY of methyl benzoate **g** using RuSNS complexes containing all the species involved in the reaction (methyl benzoate, benzyl alcohol and benzyl benzoate).

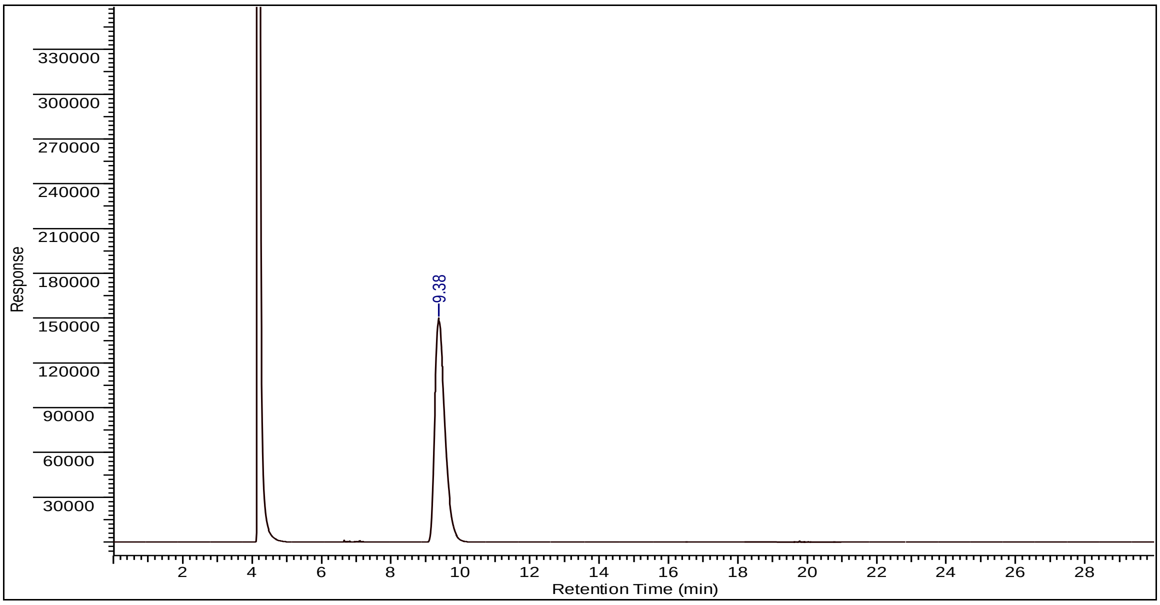

No.	Name	tR	Peak Area	Area Percent	Width
1	benzyl alcohol	9.380	3044316928.000	100.000	0.472

Figure S113. GC trace of products of methyl benzoate **g** hydrogenation catalysed by complex **1** (S/C 10000) with 50 mol% NaOEt, 27.5 bar of H_2 and 40 °C, showing full conversion to benzyl alcohol.

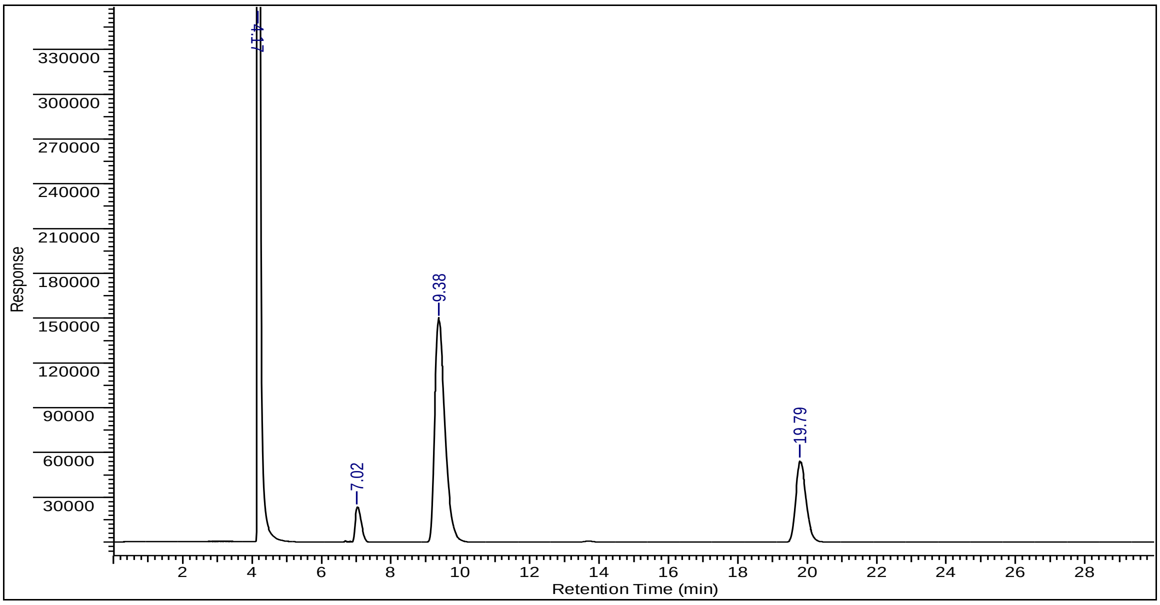

Figure S114. Representative example of GC chromatogram of the catalytic HY of ethyl benzoate **h** using RuSNS complexes containing all the species involved in the reaction (ethyl benzoate, benzyl alcohol and benzyl benzoate).

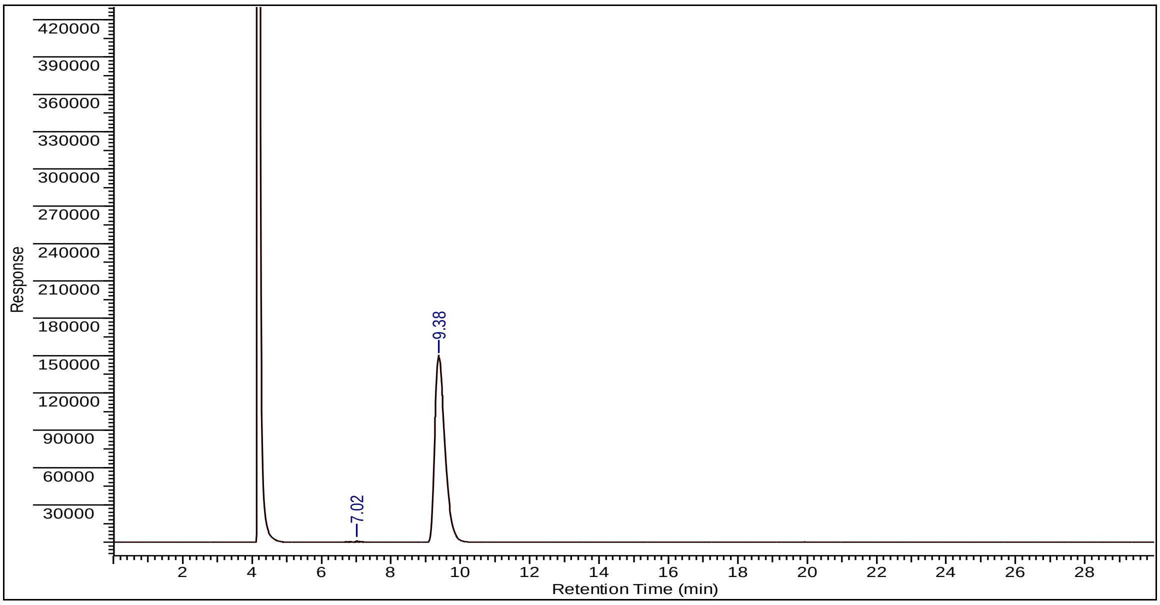

No.	Name	tR	Peak Area	Area Percent	Width
1	etil benzoato	7.022	4482973.500	0.146	0.062
2	benzyl alcohol	9.380	3059767808.000	99.854	0.472

Figure S115. GC trace of products of ethyl benzoate **h** hydrogenation catalysed by complex **1** (S/C 100000) with 50 mol% NaOEt, 27.5 bar of H_2 and 40 °C, showing full conversion to benzyl alcohol.

Synthesis of 1-decanol by large scale hydrogenation of ethyl decanoate

The hydrogenation of ethyl decanoate on a larger scale (107.6 mmol, 21.55 g, 25 mL) was performed in a 50 mL Parr autoclave reactor. The substrate has been added to the vessel, followed by NaOEt (53.79 mmol, 3.66 g) and the catalyst **1** (1.45 mg, 0.0216 µmol) from a 5.84 mg/mL solution in EtOH (250 µL, 1 vol%). The S/C molar ratio was 50000/1, whereas the base concentration was 50 mol% with respect to the substrate. A Parr High Pressure Burette system (650 mL) was connected to the reactor with a high-pressure hose, fitted with valves so that it can be sealed off from both the hydrogen supply and the autoclave. The line was then purged several times with nitrogen, three times with hydrogen, followed by slowly filling the Parr reactor to the desired pressure (28-29 bar) with H_2, and disconnected to the hydrogen source. The reaction was then set to stir at 1300 rpm, and the reactor slowly heated to 45 °C using a model 4838 Parr Temperature Controller. The reaction mixture was stirred at this temperature for 16 h. At the end of the reaction, the heating was switched off, the reactor was cooled at room temperature and the excess hydrogen was vented and the product purged with nitrogen five times. The final waxy compound was treated with 1.0 M HCl (50 mL), extracted with MTBE (40 mL) and dried with anhydrous Na_2SO_4. The product was isolated and separated from the remaining catalyst by carrying out a short filtration over a plug of silica gel and evaporation of the volatiles, leading 1-decanol as a colorless oil in 95% yield (16.16 g) that was analyzed by NMR and GC (99% purity).[1]

^{1}H-NMR (400.1 MHz, $CDCl_3$, 25 °C): δ = 3.56 (t, ^3J(H,H) = 6.7 Hz, 2H; CH_2OH), 1.63-1.43 (m, 3H; CH_2CH_2OH and OH), 1.33-1.13 (m, 14H; internal CH_2), 0.81 ppm (t, ^3J(H,H) = 6.8 Hz, 3H; CH_3). $^{13}C\{^1H\}$ NMR (100.6 MHz, $CDCl_3$, 25 °C): δ = 63.0 (s; CH_2OH), 32.8 (s; CH_2CH_2OH), 31.9 (s; CH_2), 29.6 (s; CH_2), 29.5 (s; CH_2), 29.4 (s; CH_2), 29.3 (s; CH_2), 25.7 (s; CH_2), 22.7 (s; CH_2CH_3), 14.1 ppm (s; CH_3).

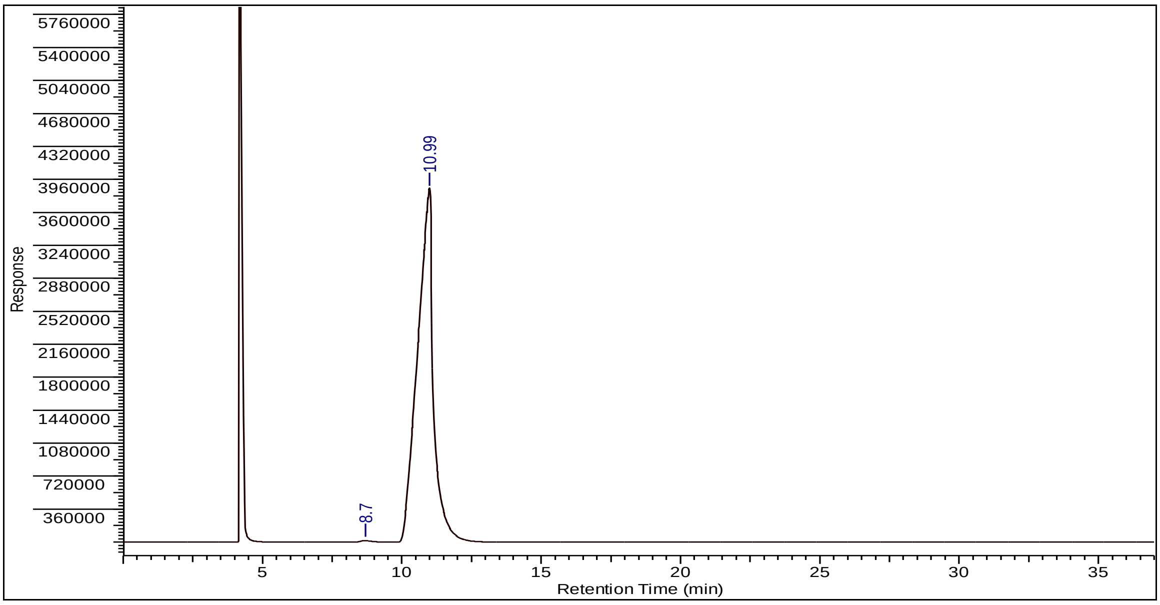

No.	Name	tR	Peak Area	Area Percent	Width
1	ethyl decanoate	8.697	413743328.000	0.266	0.642
2	1-decanol	10.987	155280965632.000	99.734	0.847

Figure S116. GC trace of products of ethyl decanoate **b** hydrogenation in multigram scale (25 mL, 21.55 g, 107.58 mmol), promoted by complex **1** at S/C 50000 (T = 45 °C, pH_2 = 28 bar, 18 h) with NaOEt 50 mol% in neat conditions.

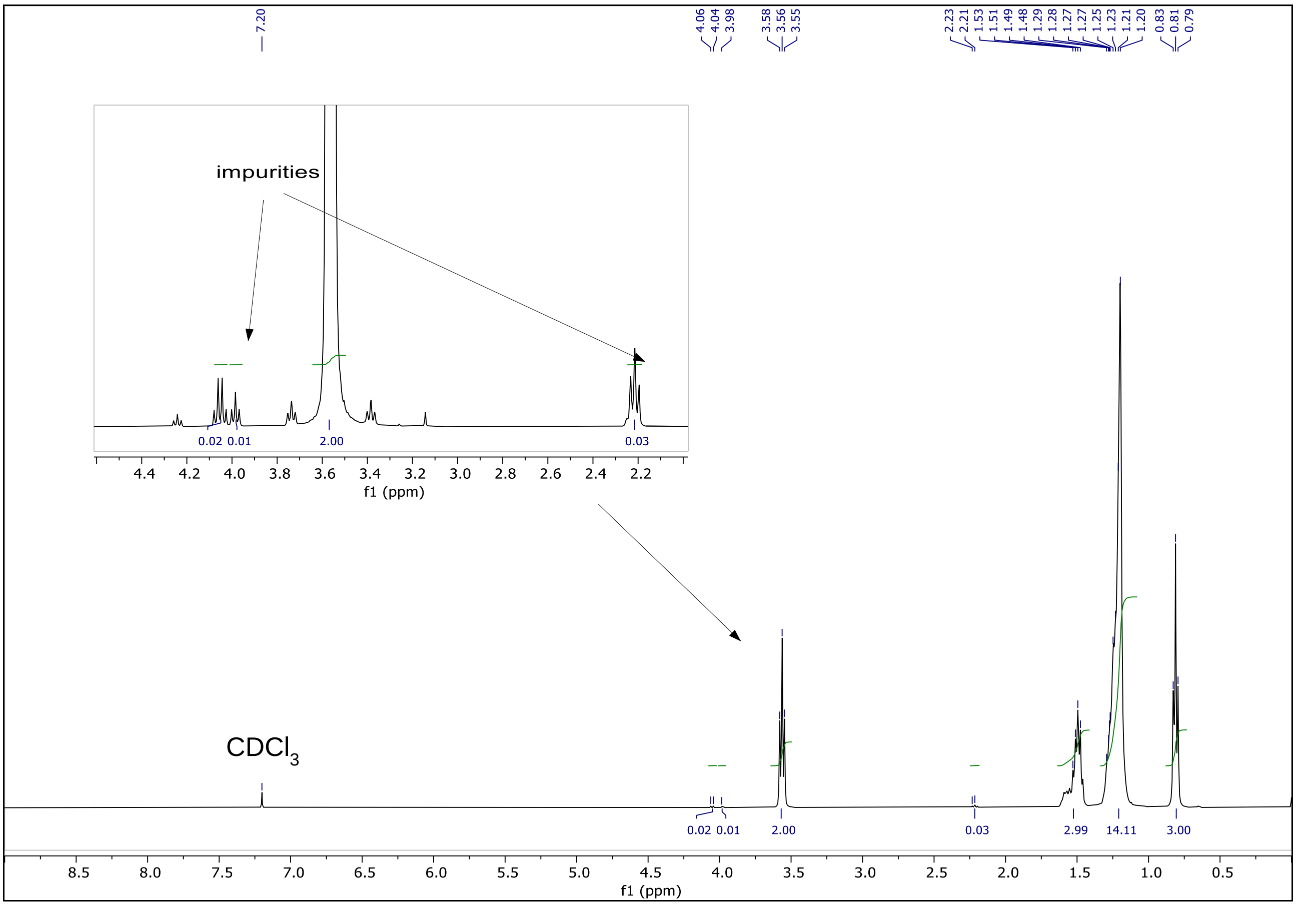

Figure S117. ^{1}H NMR spectrum (400.1 MHz) in $CDCl_3$ of 1-decanol obtained by catalytic hydrogenation of ethyl decanoate **b** in multigram scale (25 mL, 21.55 g, 107.58 mmol), promoted by complex **1** at S/C 50000 (T = 45 °C, pH_2 = 28 bar, 18 h) with NaOEt 50 mol% in neat conditions.

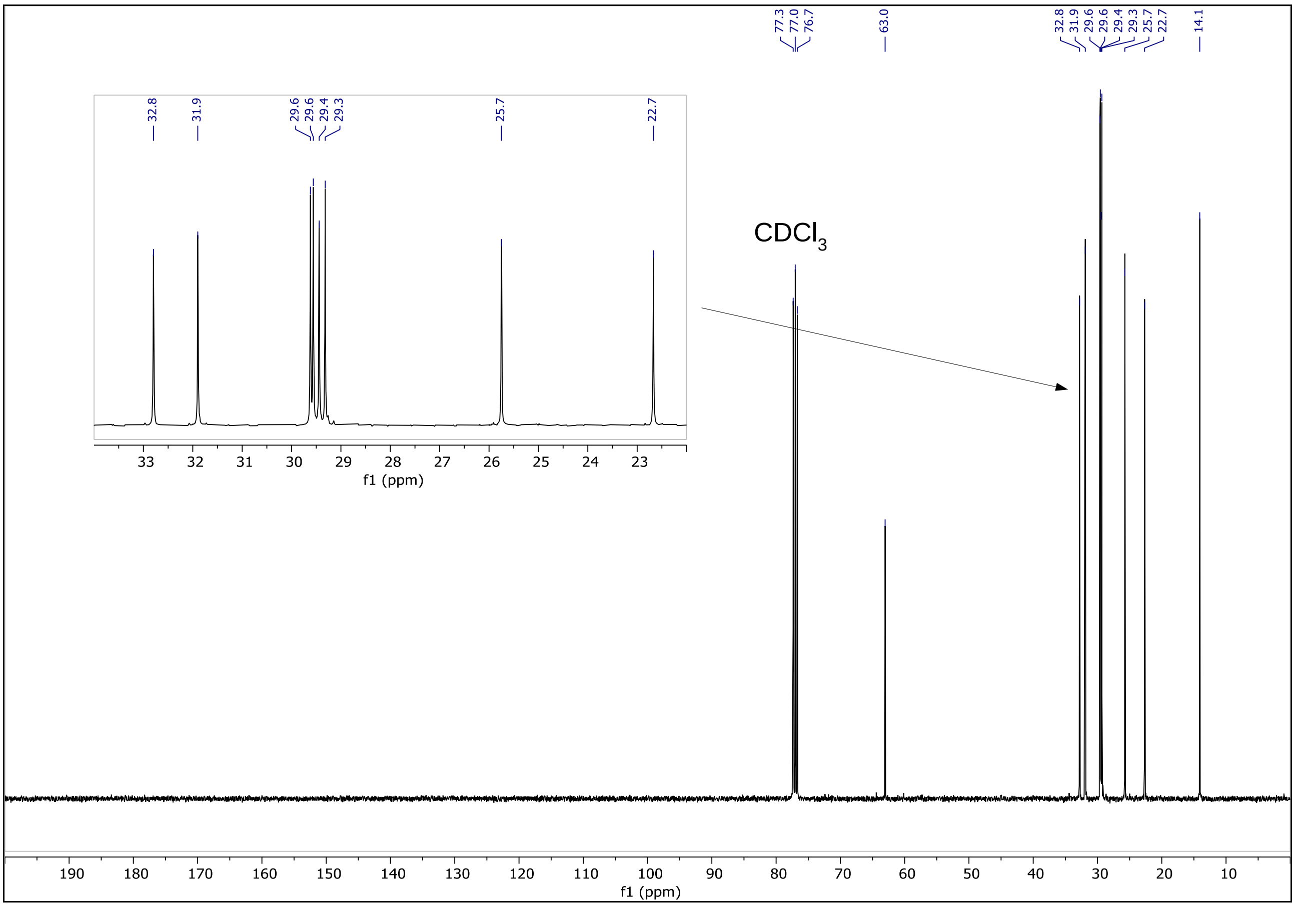

Figure S118. $^{13}C\{^{1}H\}$ NMR spectrum (100.6 MHz) in $CDCl_3$ of 1-decanol obtained by catalytic hydrogenation of ethyl decanoate **b** in multigram scale (25 mL, 21.55 g, 107.58 mmol), promoted by complex **1** at S/C 50000 (T = 45 °C, pH_2 = 28 bar, 18 h) with NaOEt 50 mol% in neat conditions.

Synthesis of oleyl alcohol by large scale hydrogenation of ethyl oleate

Oleyl alcohol was prepared on a larger scale by following the procedure used for the synthesis of 1-decanol, with ethyl oleate (69.40 mmol, 21.50 g, 25 mL) in place of ethyl decanoate in a 50 mL Parr autoclave reactor, employing the catalyst **1** (0.94 mg, 1.39 µmol) from a 3.75 mg/mL solution in EtOH (250 µL, 1 vol%). The S/C molar ratio was 50000/1, a similar result was also obtained with an S/C ratio of 100000/1 using the appropriate amounts of catalyst **1**. The stirring rate of the reaction mixture has been increased at 1400 rpm, and the reactor slowly heated to 45 °C. The hydrogen pressure was 28 bar and the reaction time was 16 h. After work-up, in the same conditions previously described for 1-decanol, oleyl alcohol was recovered in 92% yield (17.14 g) and analyzed by NMR and GC (99.9% purity). The final product contains oleyl alcohol (>78%) and other alcohols (linoleyl alcohol, palmityl alcohol, etc.) because the starting material was of technical grade and other esters were present together with ethyl oleate.[1-4]

^{1}H-NMR (400.1 MHz, $CDCl_3$, 25 °C): δ = 5.52-5.20 (m, 2H; CH=CH unsaturated alcohols), 3.57 (t, 2H, ^{3}J(H,H) = 6.8 Hz; C*H*$_2$OH), 2.70 (t, ^{3}J(H,H) = 6.3 Hz; CH=CH-C*H*$_2$-CH=CH linoleyl alcohol), 2.02-1.87 (m, 4H; CH_2), 1.49 (q, *J*(H,H) = 7.3 Hz, 2H; HOCH$_2$-C*H*$_2$-), 1.34-1.13 (m, 21H; CH_2), 0.81 ppm (t, ^{3}J(H,H) = 7.0 Hz, 3H; CH$_2$C*H*$_3$).

^{13}C{^{1}H} NMR (100.6 MHz, $CDCl_3$, 25 °C): δ = 130.2 (s; CH=CH linoleyl alcohol), 130.1 (s; CH=CH linoleyl alcohol), 130.0 (s; CH=CH oleyl alcohol), 129.8 (s; CH=CH oleyl and palmitoleyl alcohol), 129.7 (s; CH=CH palmitoleyl alcohol), 128.0 (s; CH=CH linoleyl alcohol), 127.9 (s; CH=CH linoleyl alcohol), 63.1 (s; *C*H$_2$OH oleyl alcohol), 63.0 (s; *C*H$_2$OH linoleyl alcohol), 32.8 (s; CH_2), 32.6 (s; CH_2), 31.9 (s; CH_2 oleyl alcohol), 31.5 (s; CH_2), 31.4 (s; CH_2), 29.8 (s; CH_2 oleyl alcohol), 29.7 (s; CH_2 oleyl alcohol), 29.7 (s; CH_2), 29.6 (s; CH_2), 29.5 (s; CH_2 oleyl alcohol), 29.5 (s; CH_2 oleyl alcohol), 29.4 (s; CH_2), 29.4 (s; CH_2 oleyl alcohol), 29.3 (s; CH_2), 29.3 (s; CH_2 oleyl alcohol), 29.2 (s; CH_2 oleyl alcohol), 29.2 (s; CH_2), 29.1 (s; CH_2), 27.2 (s; CH_2 oleyl alcohol), 27.2 (s; CH_2 oleyl alcohol), 25.8 (s; CH_2 oleyl alcohol), 25.6 (s; CH_2), 22.7 (s; CH_2 oleyl alcohol), 22.6 (s; CH_2), 14.1 (s; CH_3 oleyl alcohol), 14.1 ppm (s; CH_3).

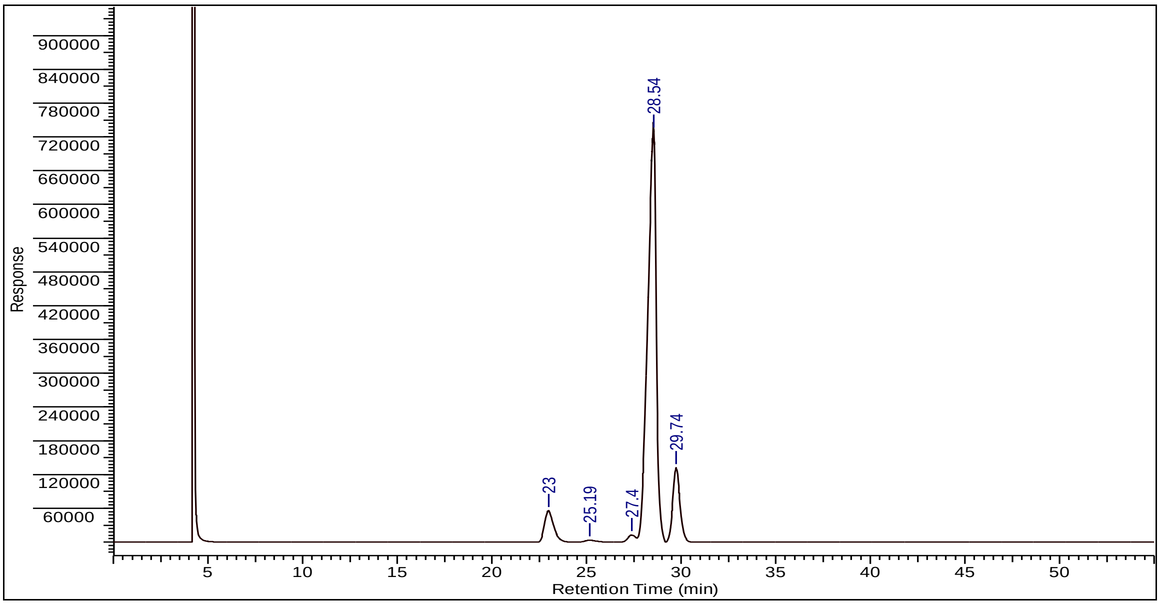

No.	Name	tR	Peak Area	Area Percent	Width
1	Palmityl alcohol (C16:0)	22.99	1880247936.000	6.473	0.772
2	Stearyl alcohol (C18:0)	25.18	134703424.000	0.464	0.974
3	Palmitoleyl alcohol (C16:1 ((Z))	27.40	325492448.000	1.121	0.709
4	Oleyl alcohol (C18:1 (Z))	28.54	23182536704.000	79.812	0.724
5	Linoleyl alcohol (C18:2 (Z))	29.73	3523532800.000	12.131	0.612

Figure S119. GC trace of products of ethyl oleate **f** hydrogenation in multigram scale (25 mL, 21.50 g, 69.40 mmol), promoted by complex **1** at S/C 50000 (T = 45 °C, pH_2 = 28 bar, 18 h) with NaOEt 50 mol% in neat conditions.

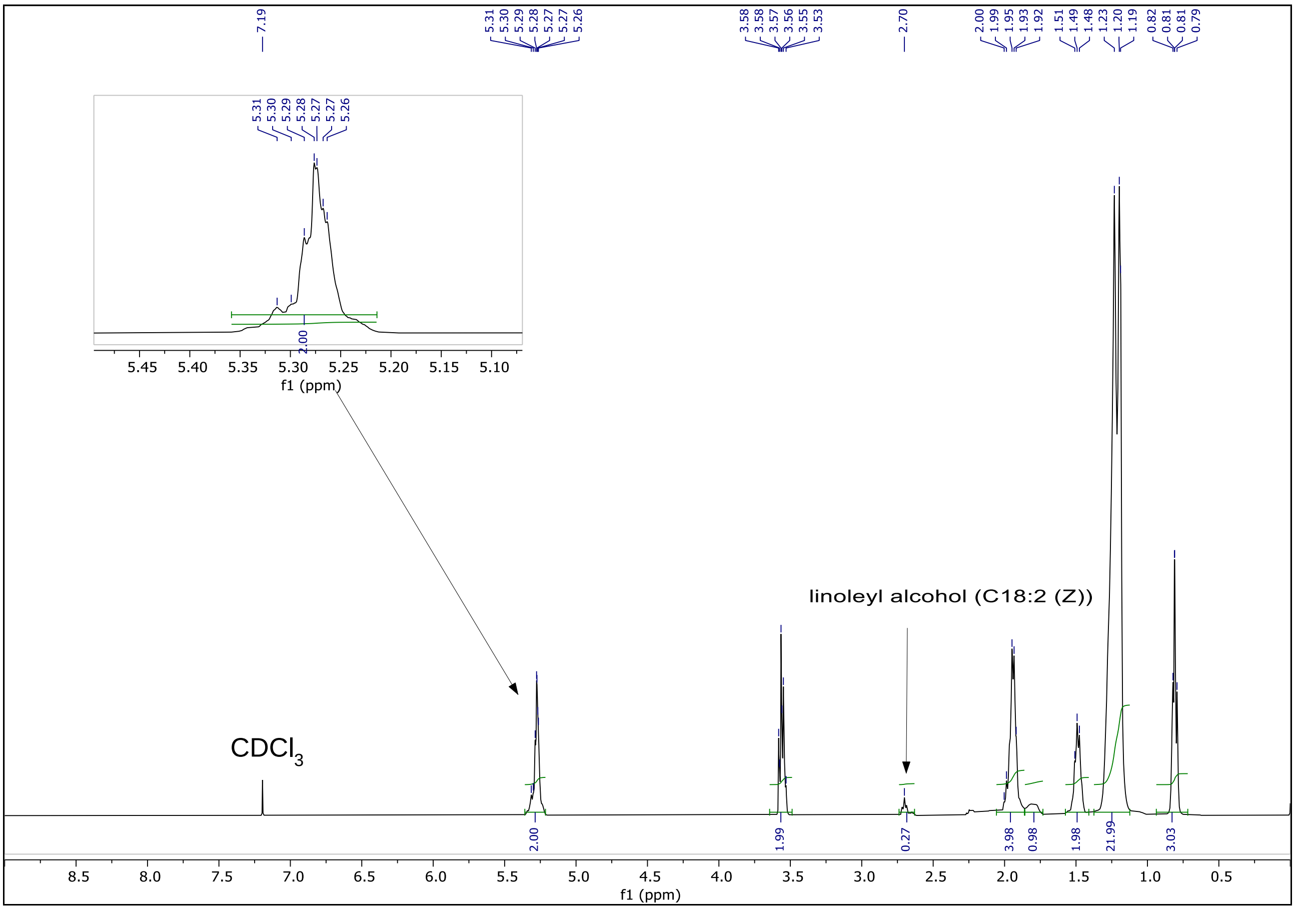

Figure S120. ^{1}H NMR spectrum (400.1 MHz) in $CDCl_3$ of ethyl oleate **f** hydrogenation products in multigram scale (25 mL, 21.50 g, 69.40 mmol), promoted by complex **1** at S/C 50000 (T = 45 °C, pH_2 = 28 bar, 18 h) with NaOEt 50 mol% in neat conditions.

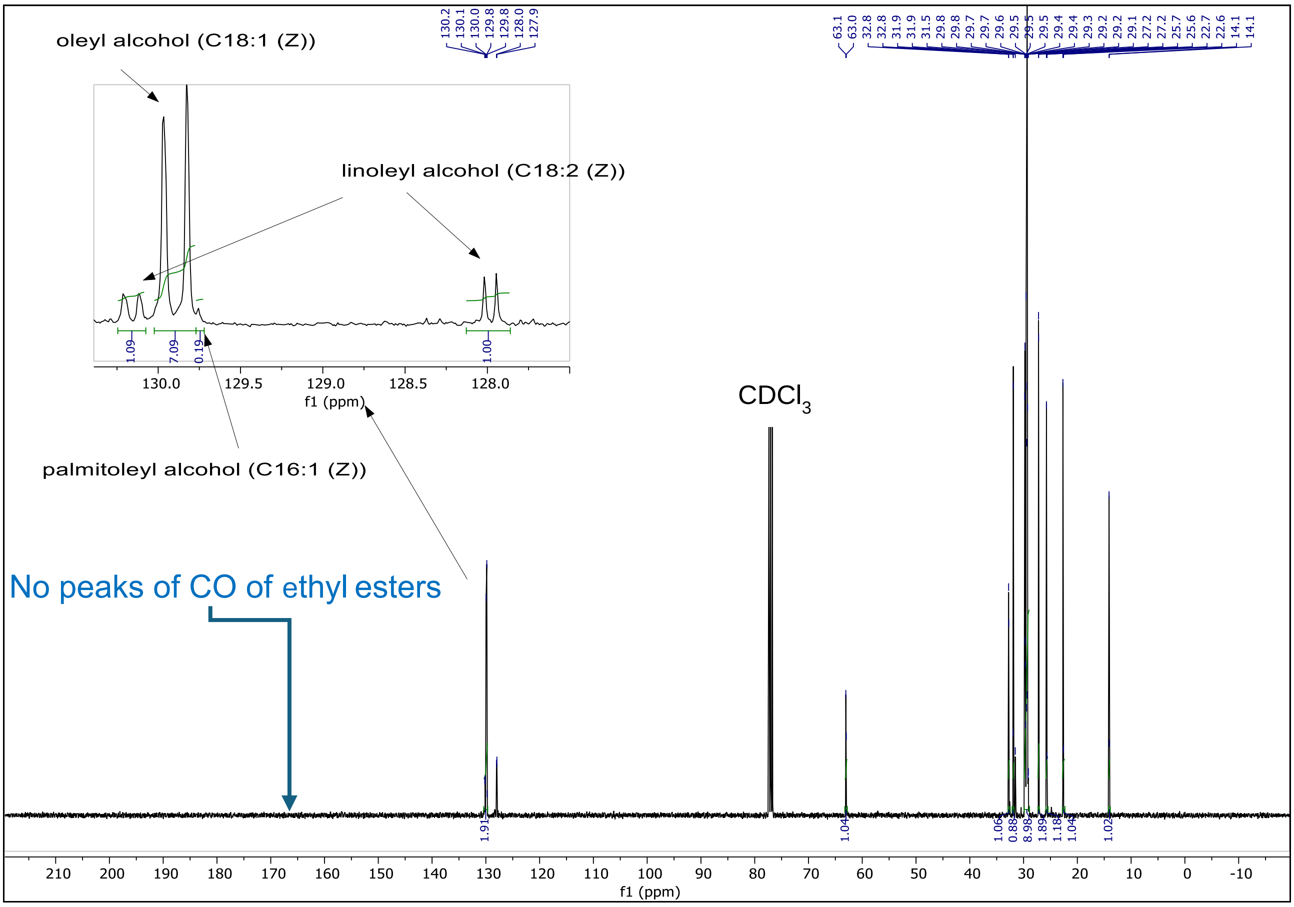

Figure S121. $^{13}C\{^1H\}$ NMR spectrum (100.6 MHz) in $CDCl_3$ of ethyl oleate **f** hydrogenation products in multigram scale (25 mL, 21.50 g, 69.40 mmol), promoted by complex **1** at S/C 50000 (T = 45 °C, pH_2 = 28 bar, 18 h) with NaOEt 50 mol% in neat conditions.

The $^{13}C\{^1H\}$ NMR spectrum of this mixture is compatible with the following composition of polyunsaturated alcohols:

- 78-80% oleyl alcohol (C18:1 (Z))
- 12-13% linoleyl alcohol (C18:2 (Z))
- 0.5-1.0% palmitoleyl alcohol (C16:1 (Z))

Single Crystal X-Ray Structure Determination of Compounds 1 (CCDC 2449278) and 3 (CCDC 2449279)

General Data

The X-ray intensity data were collected at 100 K on a Bruker D8 VENTURE three-angle diffractometer with either a TXS rotating anode or with an IMS microsource with MoK_{α} radiation (λ = 0.71073 Å) using the APEX4 software.[5] The diffractometer was equipped with a Helios optic monochromator, a Bruker PHOTON II detector, and an Oxford Cryostream low temperature device. Measurements were performed on single crystals coated with perfluorinated ether. The crystals were fixed on top of a Kapton micro sampler and frozen under a stream of cold nitrogen. A matrix scan was used to determine the initial lattice parameters. All data were integrated with the Bruker SAINT V8.40B software package using a narrow-frame algorithm and the reflections were corrected for Lorentz and polarization effects, scan speed, and background.[6] Data were corrected for absorption effects including odd and even ordered spherical harmonics by the multi-scan method (SADABS 2016/2).[7] Space group assignment was based upon systematic absences, E statistics, and successful refinement of the structure.

The structure was solved by direct methods using SHELXT and refined by full-matrix least-squares methods against F^2 by minimizing $\Sigma w(F_o^2 - F_c^2)^2$ using SHELXL in conjunction with SHELXLE.[8-10] All non-hydrogen atoms were refined with anisotropic displacement parameters. Hydrogen atoms were refined isotropically on calculated positions using a riding model with their U_{iso} values constrained to 1.5 times the U_{eq} of their pivot atoms for terminal sp^3 carbon atoms and a C–H distance of 0.98 Å. Non-methyl hydrogen atoms were refined using a riding model with methylene, aromatic, and other C–H distances of 0.99 Å, 0.95 Å, and 1.00 Å, respectively, and U_{iso} values constrained to 1.2 times the U_{eq} of their pivot atoms.

Neutral atom scattering factors for all atoms and anomalous dispersion corrections for the non-hydrogen atoms were taken from International Tables for Crystallography.[11] Supplementary crystallographic data reported in this paper have been deposited with the Cambridge Crystallographic Data Centre (CCDC: 2449278 and 2449279) and can be obtained free of charge from The Cambridge Crystallographic Data Centre via www.ccdc.cam.ac.uk/structures.[12] This report and the CIF file were generated using FinalCif.[13]

Single Crystal X-Ray Structure Determination of Compound 1 (CCDC 2449278)

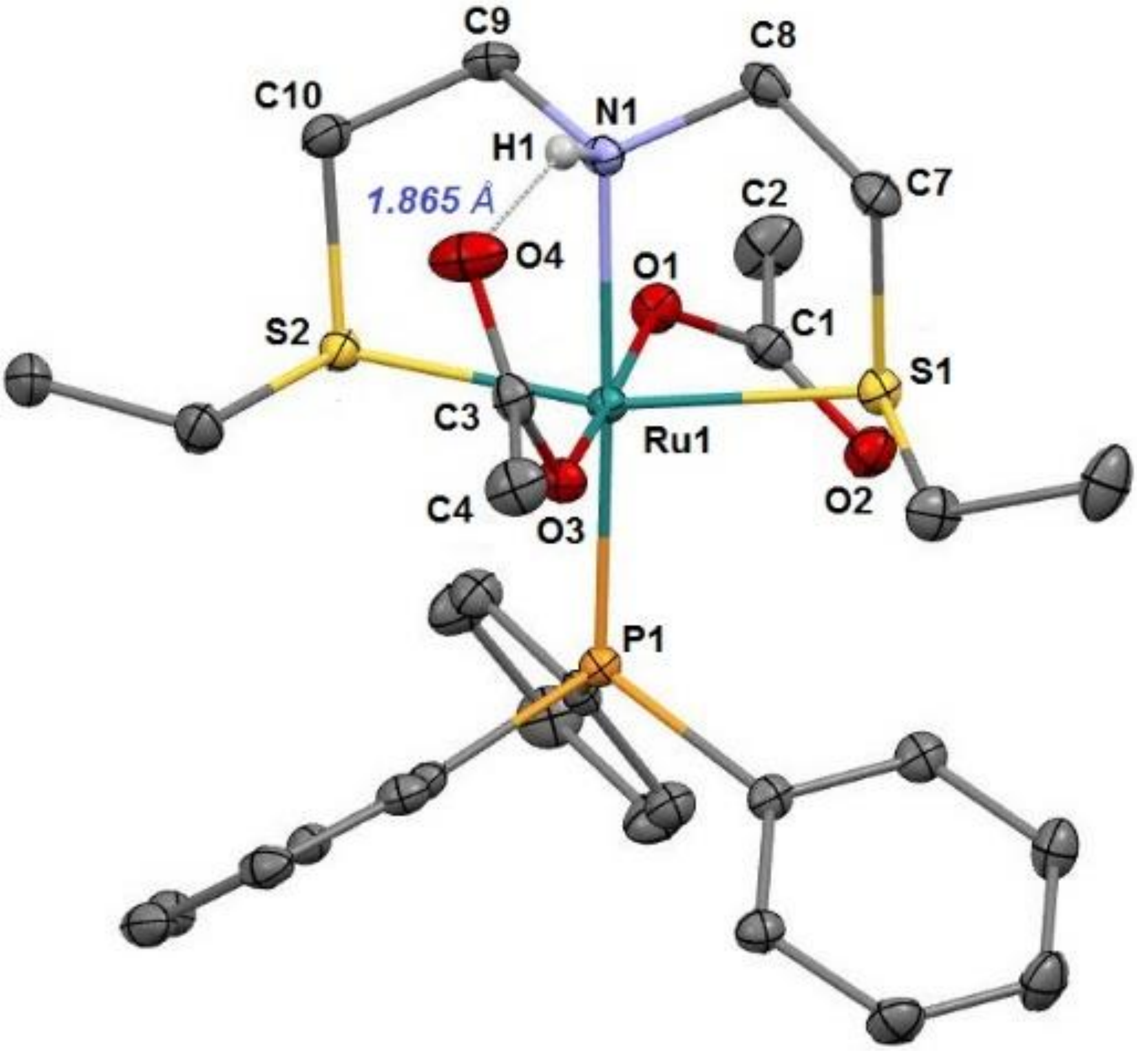

Figure S122. ORTEP representation of compound **1** in the solid state (CCDC 2449278). Ellipsoids are drawn at the 50% probability level. Hydrogen atoms (except for the amino proton) are omitted for clarity. The dotted line indicates a possible intramolecular hydrogen bond. Selected bond lengths [Å] and angles [°]: Ru1–O1 2.101(2), Ru1–O3 2.123(2), Ru1–N1 2.154(2), Ru1–P1 2.2975(8), Ru1–S1 2.3419(8), Ru1–S2 2.3440(8), S1-C7 1.838(3), S2-C10 1.838(3), N1–C8 1.473(4), N1–C9 1.482(4), O1-Ru1-O3 174.77(8), O1-Ru1-N1 83.22(9), O3-Ru1-N1 91.66(9), O1-Ru1-P1 95.07(6), O3-Ru1-P1 90.10(6), N1-Ru1-P1 176.87(7), O1-Ru1-S1 90.44(6), O3-Ru1-S1 88.02(6), N1-Ru1-S1 84.36(7), P1-Ru1-S1 98.29(3), O1-Ru1-S2 83.75(6), O3-Ru1-S2 96.64(6), N1-Ru1-S2 82.42(7), P1-Ru1-S2 94.80(3), S1-Ru1-S2 166.10(3), C8-N1-C9 112.2(2).

Single Crystal X-Ray Structure Determination of Compound 1 (CCDC 2449278)

Detailed Crystallographic Data.

Diffractometer operator:	J. Zuber
Scanspeed	5-15 s per frame
dx	38 mm
Frames:	3797 measured in 13 data sets
phi-scans with delta phi	0.5
omega-scans with delta omega	0.5
shutterless mode	

Crystal Data:

[$C_{30}H_{40}NO_4PRuS_2$]	ρ_{calc} = 1.481 g cm^{-3}
M_r = 674.79	Mo$K\alpha$ radiation, λ = 0.71073 Å
Orthorhombic	Cell parameters from 168611 reflections
space group: $Pna2_1$ (33)	2θ = 4.76–52.75° (0.80 Å)
a = 18.8398(10) Å	μ = 0.745 mm^{-1}
b = 9.5943(6) Å	T = 100 K
c = 16.7445(11) Å	Block-shaped crystal, yellow
V = 3026.6(3) Å^3	0.082 × 0.099 × 0.112 mm^3
α = β = γ = 90°	
Z = 4	
F(000) = 1400	

Data collection:

Bruker D8 Venture diffractometer	6174 independent reflections
Radiation source: IMS microsource	6033 reflections with $I > 2\sigma(I)$

Helios optic monochromator	R_{int} = 0.0426
	$2\theta_{max}$ = 52.75°, $2\theta_{min}$ = 4.76°
phi– and ω–rotation scans	h = −23 23
Absorption correction: multi-scan *SADABS* 2016/2, Bruker	k = −11 11
T_{min} =0.7036, T_{max} = 0.7461	l = −20 20
168611 measured reflections	

Data refinement:

Refinement method	Full-matrix least-squares on F2
Refinement program	SHELXL-2018/3 (Sheldrick, 2018)
Structure solution technique	direct methods
Structure solution program	SHELXT 2018/2 (Sheldrick, 2018)
Function minimized	$\Sigma w(Fo^2 - Fc^2)^2$
Data / restraints / parameters	6174 / 106 / 356
Final R indices	R1 = 0.0185, wR2 = 0.0409
	all data R1 = 0.0201, wR2 = 0.0427
Weighting scheme	$w = 1/[\sigma^2(Fo^2) + 0.0115P]$<br>where $P = (Fo^2 + 2Fc^2)/3$
Goodness-of-fit on F^2	1.232
Largest diff. peak and hole	0.49 and -0.35 eÅ^{-3}

Single Crystal X-Ray Structure Determination of Compound 1 (CCDC 2449279)

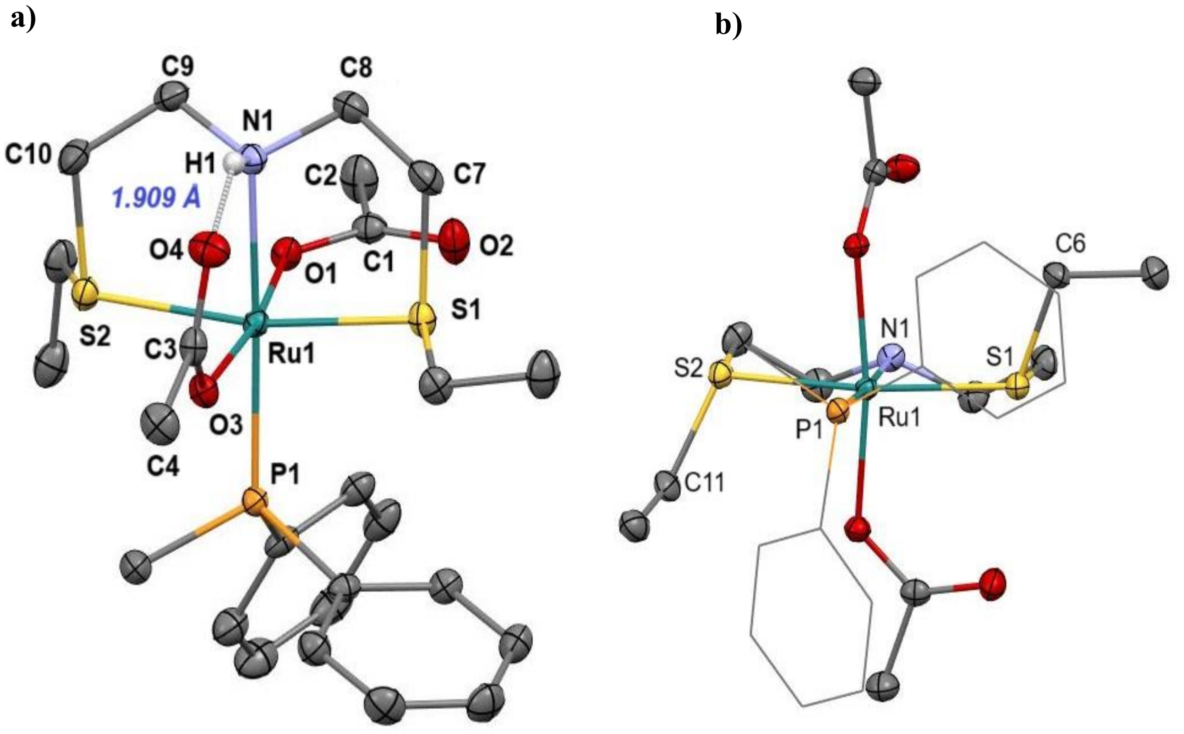

Figure S123. **a**) ORTEP representation of compound **3** in the solid state (CCDC 2449279). Ellipsoids are drawn at the 50% probability level. Hydrogen atoms (except for the amino proton) are omitted for clarity. The dotted line indicates a possible intramolecular hydrogen bond. **b**) Different orientation of the molecular structure of complex **3**, in this representation hydrogen atoms are omitted, and phosphine methyl and phenyl groups are simplified as wireframes for clarity. Selected bond lengths [Å] and angles [°]: Ru1–O1 2.1049(13), Ru1–O3 2.1173(13), Ru1–N1 2.1473(15), Ru1–P1 2.2804(5), Ru1–S1 2.3457(5), Ru1–S2 2.3527(5), S1-C7 1.846(2), S2-C10 1.829(2), N1–C8 1.469(2), N1–C9 1.474(3), O1-Ru1-O3 171.88(5), O1-Ru1-N1 85.93(6), O3-Ru1-N1 93.65(6), O1-Ru1-P1 97.72(4), O3-Ru1-P1 82.56(4), N1-Ru1-P1 176.14(5), O1-Ru1-S1 95.81(4), O3-Ru1-S1 92.21(4), N1-Ru1-S1 83.96(4), P1-Ru1-S1 96.879(18), O1-Ru1-S2 86.75(4), O3-Ru1-S2 85.14(4), N1-Ru1-S2 84.02(4), P1-Ru1-S2 94.897(18), S1-Ru1-S2 167.492(18), C8-N1-C9 113.72(15).

Single Crystal X-Ray Structure Determination of Compound 3 (CCDC 2449279)

Detailed Crystallographic Data.

Diffractometer operator:	J. Zuber
Scanspeed	1-3 s per frame
dx	45 mm
Frames:	4132 measured in 14 data sets
phi-scans with delta phi	0.5
omega-scans with delta omega	0.5
shutterless mode	

Crystal Data:

[$C_{25}H_{38}NO_4PRuS_2$]	ρ_{calc} = 1.512 g cm^{-3}
M_r = 612.72	Mo*K*α radiation, λ = 0.71073 Å
Monoclinic	Cell parameters from 146879 reflections
Space group: $P2_1/n$ (14)	2θ = 4.88-52.88° (0.80 Å)
a = 8.4607(6) Å	μ = 0.828 mm^{-1}
b = 19.4203(16) Å	T = 100 K
c = 16.4398(12) Å	Plate-shaped crystal, brown
α = 90°	0.047 × 0.060 × 0.108 mm^3
β = 94.629(3)°	
γ = 90°	
V = 2692.4(4) Å^3	
Z = 4	
F(000) = 1272	

Data collection:

Bruker D8 Venture diffractometer	5539 independent reflections
Radiation source: TXS rotating anode	4848 reflections with $I > 2\sigma(I)$
Helios optic monochromator	R_{int} = 0.0806
	$2\theta_{max}$ = 52.88°, $2\theta_{min}$ = 4.88°
phi– and ω–rotation scans	h = −10 10
Absorption correction: multi-scan *SADABS* 2016/2, Bruker	k = −24 24
T_{min} =0.7143, T_{max} = 0.7454	l = −20 20
146879 measured reflections	

Data refinement:

Refinement method	Full-matrix least-squares on F2
Refinement program	SHELXL-2018/3 (Sheldrick, 2018)
Structure solution technique	direct methods
Structure solution program	SHELXT 2018/2 (Sheldrick, 2018)
Function minimized	$\Sigma w(Fo^2 - Fc^2)^2$
Data / restraints / parameters	5539 / 123 / 312
Final R indices	R1 = 0.0228, wR2 = 0.0521
	all data R1 = 0.0294, wR2 = 0.0556
Weighting scheme	$w = 1/[\sigma^2(Fo^2) + 0.0232]$ where $P = (Fo^2 + 2Fc^2)/3$
Goodness-of-fit on F^2	1.056
Largest diff. peak and hole	0.53 and -0.34 eÅ^{-3}

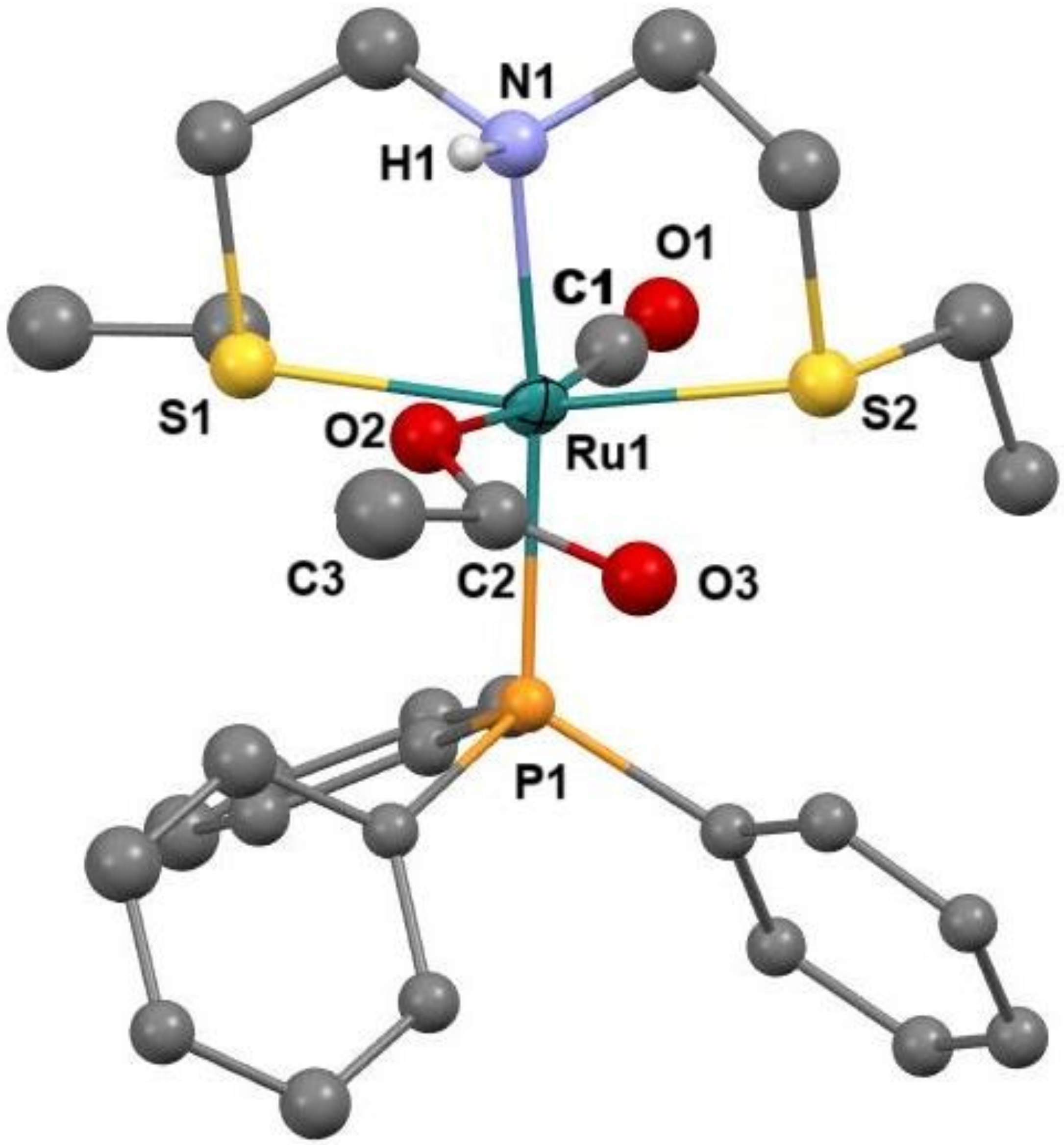

Figure S124. ORTEP representation of a poor-quality crystal of the cationic compound **5** in the solid state. Hydrogen atoms (except for the amino proton) are omitted for clarity. This figure has been inserted only in support of the structural data obtained from solution NMR studies on the compound, because the limited quality of the crystal and the present disorder do not allow the complete determination of the structure of the cationic complex and its counterion.

References

[1] S. Chakraborty, A. Das, S. K. Mandal, *Chem. Commun.*, **2021**, *57*, 12671-12674; doi: 10.1039/D1CC05614J

[2] J. Larsson, A. E. Leung, Christian Lang, B. Wu, M. Wahlgren, T. Nylander, S. Ulvenlund, A. Sanchez-Fernandez, *J. Colloid Interface Sci.*, **2021**, 585, 178-183; doi: 10.1016/j.jcis.2020.11.063

[3] H. Bruns, L. Ziesche, N. K. Taniwal, L. Wolter, T. Brinkhoff, J. Herrmann, R. Müller, S. Schulz, *Beilstein J. Org. Chem.* **2018,** *14,* 2964-2973; doi: 10.3762/bjoc.14.276

[4] K. Isozaki, H. Matsuda, R. Agata, J. Jeon, B. Wu, F. Pincella, M. Ikenaga, Y. Tachibana, Y. Ohta, M. Nakamura, *RSC Sustain.* **2024**, 2, 1358-1362; doi: 10.1039/D3SU00446E

[5] *APEX4 Suite of Crystallographic Software, Version 2021-10.0,* Bruker AXS Inc., Madison, Wisconsin, USA, **2021**.

[6] Bruker, *SAINT, V8.40B*, Bruker AXS Inc., Madison, Wisconsin, USA.

[7] L. Krause, R. Herbst-Irmer, G. M. Sheldrick, D. Stalke, *J. Appl. Cryst.* **2015**, *48*, 3–10, doi:10.1107/S1600576714022985.

[8] G. M. Sheldrick, *Acta Cryst.* **2015**, *A71*, 3–8, doi:10.1107/S2053273314026370.

[9] G. M. Sheldrick, *Acta Cryst.* **2015**, *C71*, 3–8, doi:10.1107/S2053229614024218.

[10] C. B. Huebschle, G. M. Sheldrick, B. Dittrich, *J. Appl. Cryst.* **2011**, *44*, 1281–1284, doi:10.1107/S0021889811043202.

[11] Ed. E. Prince, *International Tables for Crystallography Volume C, Mathematical, Physical and Chemical Tables*, International Union of Crystallography, Chester, England, **2006**, 500–502; 219–222; 193–199.

[12] C. R. Groom, I. J. Bruno, M. P. Lightfoot, S. C. Ward, *Acta Cryst.* **2016**, *B72*, 171–179, doi:10.1107/S2052520616003954.

[13] D. Kratzert, *FinalCif*, *V144*, https://dkratzert.de/finalcif.html.
